# Secosteroid–2-Pyrazoline Hybrids: Design, Synthesis, Biological Evaluation and Development of Therapeutic Combinations Against ERα-Positive Breast Cancer Cells

**DOI:** 10.3390/biomedicines13123057

**Published:** 2025-12-11

**Authors:** Alexey I. Ilovaisky, Alexander M. Scherbakov, Fedor B. Bogdanov, Dumitru Miciurov, Elena I. Chernoburova, Valentina M. Merkulova, Eugene I. Bozhenko, Andrey S. Dmitrenok, Diana I. Salnikova, Danila V. Sorokin, Alvina I. Khamidullina, Mikhail A. Krasil’nikov, Igor V. Zavarzin, Alexander O. Terent’ev

**Affiliations:** 1 N. D. Zelinsky Institute of Organic Chemistry, Russian Academy of Sciences, Leninsky Prospekt 47, 119991 Moscow, Russia; dimon.dimon284@gmail.com (D.M.); chernoburova@mail.ru (E.I.C.); merk68@mail.ru (V.M.M.); dr_bozhenko@mail.ru (E.I.B.); dmt@ioc.ac.ru (A.S.D.); d.salnikova@ronc.ru (D.I.S.); alvina@genebiology.ru (A.I.K.); igorzavarzin@yandex.ru (I.V.Z.); 2 N.N. Blokhin National Medical Research Center of Oncology, Kashirskoye Shosse 24, 115522 Moscow, Russia; a.sherbakov@ronc.ru (A.M.S.); f.bogdanov.f@yandex.ru (F.B.B.); d.sorokin@ronc.ru (D.V.S.); krasilnikovm1@yandex.ru (M.A.K.); 3 Gause Institute of New Antibiotics, Bol’shaya Pirogovskaya Ulitsa 11, 119021 Moscow, Russia; 4 Institute of Gene Biology, Russian Academy of Sciences, Ulitsa Vavilova 34/5, 119334 Moscow, Russia

**Keywords:** secosteroids, pyrazolines, hybrid molecules, breast cancer, antiproliferative activity, Bcl-2, MCF-7, T47D, therapeutic combinations

## Abstract

**Background/Objectives:** Breast cancer remains one of the most prevalent and life-threatening malignancies worldwide. This study describes the design and biological evaluation of a series of secosteroid–2-pyrazoline hybrids as novel antitumor agents against ERα-positive breast cancer cell lines MCF-7 and T47D. **Methods**: A simple and efficient method for synthesizing secosteroid–2-pyrazoline hybrids was developed starting from 13α-hydroxy-3-methoxy-13,17-secoestra-1,3,5(10)-triene-17-oic acid hydrazide and 1,3-diketones. The resulting secosteroid derivatives were evaluated against hormone-dependent MCF-7 and T47D breast cancer cells. Furthermore, the selectivity and effects of three lead compounds on signaling pathways in MCF-7 cells were examined. Flow cytometry was used to assess the cell-cycle distribution of MCF-7 cells treated with the lead compound. **Results**: Among the synthesized hybrids, compounds **3f**, **3j**, and **3k** exhibited potent antiproliferative activity with IC_50_ values of 0.2–0.5 μM against breast cancer cells, while demonstrating very low cytotoxicity towards normal cells (IC_50_ > 25 μM), indicating a favorable safety profile. The antitumor activity of lead compound **3j** was additionally investigated in combination with standard chemotherapeutics, docetaxel and doxorubicin, yielding synergistic effects. The lead compounds showed a dual mechanism of action by inhibiting S6 kinase and promoting Bcl-2 phosphorylation at 0.9 μM, without significantly affecting hormonal breast cancer targets such as ERα, GREB1, and AR. Compound **3j** induced apoptosis accompanied by a reduction of the G1/G0 phase in MCF-7 cells. **Conclusions**: These findings highlight secosteroid–2-pyrazoline hybrids as promising candidates for the development of next-generation breast cancer therapeutics targeting apoptosis and S6K signaling pathways.

## 1. Introduction

Cancer remains one of the most significant challenges to modern healthcare. According to multiple World Health Organization (WHO) reports, oncological diseases consistently rank second among the leading causes of global mortality—surpassed only by cardiovascular disorders [1]. Recent years have shown a sharp increase in cancer incidence, and by 2045 the number of newly diagnosed cases is projected to exceed 32.6 million annually, which is 1.6-fold higher than in 2022 [2]. While lung cancer remains the most frequently diagnosed malignancy in both sexes combined, breast cancer ranks first among women, with 2.3 million new cases in 2022 [3]. Notably, 37.6% of breast cancer diagnoses occur in North America and Europe, likely due to more advanced diagnostic capabilities and widespread implementation of early screening programs in high-income countries [4]. Despite considerable achievements, breast cancer treatment outcomes are often limited by tumor heterogeneity, diverse morphological features, and variability in clinical course. The WHO classification dividing breast tumors into invasive ductal and invasive lobular carcinoma is frequently insufficient for treatment selection. Therefore, assessment of estrogen (ERα), progesterone (PR), HER2 receptor status, and proliferation index Ki-67 is essential for precise tumor stratification [5,6]. Based on receptor expression, breast cancer is categorized into several biological subtypes, among which luminal A and B, characterized by ERα/PR-positivity, are the most common [7]. Hormonal factors—including early menarche, late menopause [8], nulliparity, absence of breastfeeding, and prolonged hormone-replacement therapy [9,10]—significantly increase the risk of hormone-dependent breast cancer development [11]. Estrogen binding to its receptor activates estrogen-response elements regulating tumor growth-related genes. In addition, estrogen can directly modulate signaling cascades governing proliferation, survival, apoptosis resistance, and progesterone signaling [12,13]. Modern endocrine therapy is based on suppression of estrogen function via several mechanisms: aromatase or steroid sulfatase inhibition [14,15], selective estrogen-receptor modulators (SERMs) [16], and estrogen-receptor α degraders (SERDs) that induce partial or complete receptor loss [17]. However, treatment efficacy is often impaired by drug resistance, low tissue selectivity, endocrine side effects, and systemic toxicity [18,19]. Consequently, the discovery of new antitumor agents suitable for chemo- and hormone-therapy remains a priority in medicinal chemistry and molecular oncology.

Natural compounds containing steroid and terpenoid scaffolds represent an invaluable source of anticancer agents due to their favorable pharmacological features and structural diversity [20,21]. Among them, lupane-type triterpenoids (betulin, betulinic acid) demonstrate selective cytotoxicity toward tumor cells with minimal effect on normal tissues [22,23]. Betulinic acid induces apoptosis via mitochondrial permeabilization and cytochrome C release independent of p53 or death-receptor signaling [24,25,26], enabling it to overcome resistance in tumors overexpressing anti-apoptotic Bcl-2 proteins [27,28]. Its broad anticancer activity includes neuroblastoma, glioblastoma, ovarian, colorectal, and breast cancers, and leukemia [29,30,31], although poor solubility (~0.5 μg/mL) and low oral bioavailability restrict clinical application. These limitations highlight the value of rational structural modification in drug development.

Steroid-derived anticancer agents possess numerous advantages including good membrane permeability, improved bioavailability, and reduced systemic toxicity due to structural similarity to endogenous hormones [32]. One of the most promising strategies for modifying steroids is ring opening to form secosteroids [33]. D-secoestrane derivatives exhibit strong antiproliferative activity without estrogenic properties [34,35,36,37,38,39,40,41,42], making them highly attractive for hormone-dependent breast cancer treatment [36,37].

Molecular hybridization—integration of multiple pharmacophores into a single structure—is a productive strategy for multitarget drug design [43,44]. Steroid–heterocycle hybridization often increases therapeutic potency, reduces toxicity, and helps overcome drug resistance [45,46,47,48].

**Figure 1:**
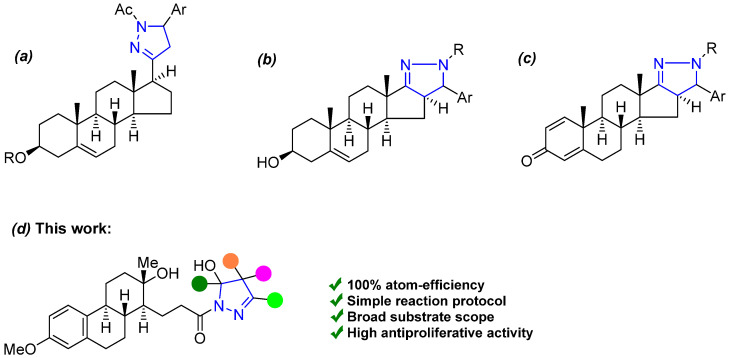
Steroid derivatives with good anticancer activity containing a 2-pyrazoline moiety.

Among nitrogen heterocycles, 2-pyrazoline is a particularly valuable scaffold for anticancer agent development [49,50,51,52,53,54,55]. Pyrazoline-based hybrids exhibit a diverse mode of action, including VEGFR-2 and EGFR inhibition, tubulin disruption, and topoisomerase II inhibition, collectively suppressing cell division, DNA replication, and migration [56,57,58,59,60,61]. Steroid–pyrazoline hybrids have been much less studied, although several 17-substituted analogs have demonstrated significant cytotoxicity against various human cancer cell lines. (Figure 1a). A series of 3β-hydroxyandrost-5-enes with 1-acetyl-5-aryl-2-pyrazoline moiety in the 17 position possessed significant cytotoxic activity against a panel of human cancer cell lines (HT-29, HCT-15, 502713, HOP-62, A-545, MCF-7, SF-295) [62]. Some of these and similar compounds showed antiproliferative effects towards HeLa, MCF-7, and A431 cancer cells comparable to cisplatin [63], although androsta-1,4-dien-3-ones with 1-acetyl-5-aryl-2-pyrazoline fragment showed only moderate activity against NCI-H460, HeLa, and HepG2 cancer cells [64]. The 17-substituted steroidal glycoside–2-pyrazolines were also characterized by growth inhibition of A-549, SF-295, HCT-15, and OVCAR-3 cells comparable or superior to cisplatin [65]. Another approach to the steroid–pyrazoline hybrid compounds is based on fused structures. The androst-5-ene and androst-4,6-diene derivatives with D-ring-fused 5-arylpyrazolines with promising activity against HeLa and MCF-7 cells, acting as potent aromatase and quinone reductase-2 inhibitors, were reported by Amr Ael and co-authors [66,67] (Figure 1b,c). On the contrary, D-ring-fused tigogenin pyrazolines [68] and 2′-substituted-5α-cholestano [5,7-cd]pyrazolines [69] have demonstrated only weak inhibitory effect against different human cancer cell lines.

To date, no secosteroid–pyrazoline hybrids have been reported, despite their structural and biochemical potential making them attractive candidates for development as new anticancer agents.

Recently, we reported an approach to novel secosteroids with high cytotoxicity and good selectivity towards MCF-7 and multidrug-resistant NCI/ADR-RES cells based on the ring D opening of 13α-/13β-17a-oxa-17a-homoestra-1,3,5(10)-trien-17-ones by hydrazine hydrate, followed by subsequent modifications of 13α-hydroxy-3-methoxy-13,17-secoestra-1,3,5(10)-trien-17-oic acid hydrazide [70,71,72,73]. The present work is aimed at the synthesis of secosteroid–2-pyrazoline hybrids (Figure 1d). These novel derivatives of 13α-hydroxy-3-methoxy-13,17-secoestra-1,3,5(10)-trien-17-oic acid were evaluated against hormone-dependent MCF-7 and T47D breast cancer cell lines. Furthermore, the selectivity and effects of the three lead compounds on signaling pathways in MCF-7 cells were analyzed.

## 2. Materials and Methods

### 2.1. General Information

NMR spectra were obtained using AVANCE II 300, DRX500, and AVANCE II 600 spectrometers (Bruker BioSpin GmbH, Rheinstetten, Germany) at ambient temperature. Chemical shifts (δ) are given in ppm relative to the corresponding solvent signals (^1^H: DMSO-*d*_6_, δ = 2.50 ppm; ^13^C: DMSO-*d*_6_, δ = 39.50 ppm). Coupling constants (J) are given in Hertz. High-resolution mass spectra were recorded on a Bruker microTOF II instrument equipped with an electrospray ionization (ESI) source. Measurements were carried out in positive ion mode (MS+) using an interface capillary voltage of 4500 V and a scan range of *m*/*z* 50–3000. External calibration was performed with a HCOONa solution in MeCN. Samples dissolved in MeCN were introduced via direct syringe injection at a flow rate of 3 mL/min. Melting points are uncorrected and were determined using a Boetius capillary apparatus. Thin-layer chromatography (TLC) was performed on silica gel 60 F254 plates and spots were visualized using UV light (254 nm) and by treatment with Ce(SO_4_)_2_/H_2_SO_4_ solution. Column chromatography was carried out on silica gel 60 A (0.060–0.200 mm) (Acros Organics, Geel, Belgium). Steroidal hydrazide **1** was prepared using the published procedure [70]. Pentane-2,4-dione **2a**, heptane-3,5-dione **2o**, benzoylacetone **2q**, and 1,1,1-trifluoropentane-2,4-dione **2t** were commercially available and were used without purification. Other substituted 1,3-dicarbonyl compounds, **2b** [74], **2c** [75], **2d** [74], **2e** [76], **2f** [77], **2g** [78], **2h** [79], **2i** [80], **2j** [81], **2k** [82], **2l** [79], **2m** [83], **2n** [84], **2p** [79], **2r** [85], and **2s** [86], were synthesized according to the literature. All reactions were performed under air.

### 2.2. Synthetic Procedure for Compounds **3a**–**t**

1,3-Diketones **2a**–**t** (30–80 mg, 0.30 mmol) were added to a solution of hydrazide **1** (100 mg, 0.30 mmol) in EtOH (5 mL), and the reaction mixture was stirred under reflux for 8 h (TLC control). Then the solvent was evaporated under reduced pressure using a rotary evaporator (15–20 mmHg). The pure secosteroid–2-pyrazoline hybrid compounds **3a**–**t** were isolated by column chromatography on silica gel (eluent 0–20% EtOAc (EA) in petroleum ether (PE)).

1-{3-[(1*S*,2*S*,4a*S*,10a*R*)-2-Hydroxy-7-methoxy-2-methyl-1,2,3,4,4a,9,10,10a-octahydrophenanthren-1-yl]propanoyl}-3,5-dimethyl-4,5-dihydro-1*H*-pyrazol-5-ol (**3a**). Yield 74%. White solid, mp 71–73 °C, R_f_ = 0.5 (PE:EA = 1:2). ^1^H NMR (600 MHz, DMSO-*d*_6_) *δ* 0.98 (s, 3H, H-18), 1.08–1.20 (m, 3H, H-8, H-11, H-14), 1.23–1.31 (m, 1H, H-7), 1.38–1.47 (m, 1H, H-15), 1.55 (td, *J* = 12.2, 2.8 Hz, 1H, H-12α), 1.74 (dt, *J* = 12.2, 2.8 Hz, 1H, H-12β), 1.748 and 1.749 (both s, total 3H, C*H*_3_-C5′), 1.85–1.95 (m, 1H, H-15), 1.93 (s, 3H, C*H*_3_-C3′), 2.13–2.19 (m, 1H, H-7), 2.20–2.26 (m, 1H, H-9), 2.28–2.34 (m, 1H, H-11), 2.51–2.65 (m, 2H, H-16), 2.71–2.83 (m, 3H, H-6, H-4′), 2.87 (d, *J* = 18.7 Hz, 1H, H-4′), 3.68 (s, 3H, OMe), 4.29 (s, 0.5H, C13-O*H*), 4.32 (s, 0.5H, C13-O*H*), 6.21 (s, 1H, C5′-O*H*), 6.60 (d, *J* = 2.8 Hz, 1H, H-4), 6.67 (dd, *J* = 8.7, 2.8 Hz, 1H, H-2), 7.17 (d, *J* = 8.7 Hz, 1H, H-1). ^13^C NMR (150 MHz, DMSO-*d*_6_) *δ* 15.86 (*C*H_3_-C3′), 21.04 (C-18), 22.70 and 22.77 (C-15), 26.24 (*C*H_3_-C5′), 26.88 (C-7), 28.30 (C-11), 30.04 (C-6), 36.56 and 36.60 (C-16), 42.37 (C-12), 42.85 (C-9), 43.37 (C-8), 51.64 (C-14), 52.04 (C-4′), 54.85 (OMe), 71.55 (C-13), 90.06 and 90.08 (C-5′), 111.66 (C-2), 113.02 (C-4), 126.48 (C-1), 131.96 (C-10), 137.37 (C-5), 153.71 (C-3′), 157.04 (C-3), 171.00 and 171.04 (C-17). HRMS (ESI) *m*/*z* [*M* + Na]^+^ calcd for [C_24_H_34_N_2_O_4_Na]^+^: 437.2411, found: 437.2415.

1-{3-[(1*S*,2*S*,4a*S*,10a*R*)-2-Hydroxy-7-methoxy-2-methyl-1,2,3,4,4a,9,10,10a-octahydrophenanthren-1-yl]propanoyl}-3,4,5-trimethyl-4,5-dihydro-1*H*-pyrazol-5-ol (**3b**). Yield 67%. White solid, mp 71–72 °C, R_f_ = 0.5 (PE:EA = 1:1). ^1^H NMR (300 MHz, DMSO-*d*_6_) *δ* 0.98 (s, 3H, H-18), 1.03 (d, *J* = 7.2 Hz, 3H, C*H*_3_-C4′), 1.07–1.33 (m, 4H, H-7, H-8, H-11, H-14), 1.35–1.48 (m, 1H, H-15), 1.49–1.62 (m, 1H, H-12), 1.69–1.78 (m, 1H, H-12), 1.71 (s, 3H, CH_3_, C*H*_3_-C5′), 1.84–1.95 (m, 1H, H-15), 1.89 (s, 3H, CH_3_, C*H*_3_-C3′), 2.09–2.38 (m, 3H, H-7, H-9, H-11), 2.52–2.86 (m, 5H, H-4′, H-6, H-16), 3.68 (s, 3H, OMe), 4.31 and 4.33 (both s, total 1H, C13-O*H*), 6.03 (br s, 1H, C5′-O*H*), 6.59 (d, *J* = 2.8 Hz, 1H, H-4), 6.66 (dd, *J* = 8.6, 2.8 Hz, 1H, H-2), 7.16 (d, *J* = 8.6 Hz, 1H, H-1). ^13^C NMR (75 MHz, DMSO-*d*_6_) *δ* 8.56 and 8.62 (*C*H_3_-C4′), 13.96 (*C*H_3_-C3′), 21.06 (C-18), 22.68 and 22.80 (C-15), 25.90 (*C*H_3_-C5′), 26.90 (C-7), 28.33 (C-11), 30.07 (C-6), 36.67 and 36.70 (C-16), 42.38 (C-12), 42.87 (C-9), 43.40 (C-8), 51.56 (C-14), 52.75 and 52.77 (C-4′), 54.86 (OMe), 71.58 (C-13), 90.25 and 90.29 (C-5′), 111.68 (C-2), 113.04 (C-4), 126.50 (C-1), 131.96 (C-10), 137.39 (C-5), 157.06 (C-3), 157.44 and 157.46 (C-3′), 171.62 and 171.67 (C-17). HRMS (ESI) *m*/*z* [*M* + Na]^+^ calcd for [C_25_H_36_N_2_O_4_Na]^+^: 451.2567, found: 451.2560.

1-{3-[(1*S*,2*S*,4a*S*,10a*R*)-2-Hydroxy-7-methoxy-2-methyl-1,2,3,4,4a,9,10,10a-octahydrophenanthren-1-yl]propanoyl}-3,4,4,5-tetramethyl-4,5-dihydro-1*H*-pyrazol-5-ol (**3c**). Yield 62%. White solid, mp 58–60 °C, R_f_ = 0.42 (PE:EA = 1:1). ^1^H NMR (600 MHz, DMSO-*d*_6_) *δ* 0.900 and 0.903 (both s, total 3H, C*H*_3_-C4′), 0.979 (s, 3H, H-18), 0.996 and 0.998 (both s, total 3H, C*H*_3_-C4′), 1.08–1.20 (m, 3H, H-8, H-11, H-14), 1.23–1.31 (m, 1H, H-7), 1.38–1.48 (m, 1H, H-15), 1.55 (br t, *J* = 13.1 Hz, 1H, H-12α), 1.609 and 1.615 (both s, total 3H, C*H*_3_-C5′), 1.74 (dt, *J* = 13.1, 3.2 Hz, 1H, H-12β), 1.84–1.94 (m, 1H, H-15), 1.891 and 1.894 (both s, total 3H, C*H*_3_-C3′), 2.11–2.20 (m. 1H, H-7), 2.20–2.26 (m, 1H, H-9), 2.28–2.34 (m, 1H, H-11), 2.51–2.65 (m, 2H, H-16), 2.71–2.83 (m, 2H, H-6), 3.68 (s, 3H, OMe), 4.26 (s, 0.5H, C13-O*H*), 4.30 (s, 0.5H, C13-O*H*), 5.956 and 5.963 (both s, total 1H, C5′-O*H*), 6.60 (d, *J* = 2.8 Hz, 1H, H-4), 6.67 (dd, *J* = 8.7, 2.8 Hz, 1H, H-2), 7.17 (d, *J* = 8.7 Hz, 1H, H-1). ^13^C NMR (150 MHz, DMSO-*d*_6_) *δ* 12.02 (*C*H_3_-C3′), 16.52 and 16.64 (*C*H_3_-C4′), 21.00 and 21.04 (*C*H_3_-C5′), 21.10 (C-18), 21.25 and 21.31 (*C*H_3_-C4′), 22.69 (C-15), 26.84 and 26.89 (C-7), 28.28 (C-11), 30.02 (C-6), 36.73 and 36.95 (C-16), 42.35 and 42.40 (C-12), 42.85 (C-9), 43.31 and 43.38 (C-8), 51.61 (C-14), 53.43 (C-4′), 54.83 (OMe), 71.53 (C-13), 92.66 and 92.74 (C-5′), 111.64 (C-2), 113.00 (C-4), 126.45 (C-1), 131.94 (C-10), 137.35 (C-5), 157.03 (C-3), 161.42 and 161.44 (C-3′), 172.53 and 172.58 (C-17). HRMS (ESI) *m*/*z* [*M* + H]^+^ calcd for [C_26_H_39_N_2_O_4_]^+^: 443.2904, found: 443.2901.

4-Ethyl-1-{3-[(1*S*,2*S*,4a*S*,10a*R*)-2-hydroxy-7-methoxy-2-methyl-1,2,3,4,4a,9,10,10a-octahydrophenanthren-1-yl]propanoyl}-3,5-dimethyl-4,5-dihydro-1*H*-pyrazol-5-ol (**3d**). Yield 63%. White solid, mp 54–56 °C, R_f_ = 0.5 (PE:EA = 1:1). ^1^H NMR (300 MHz, DMSO-*d*_6_ *δ* 0.99 (s, 3H, H-18), 1.01 (t, *J* = 7.3 Hz, 3H, C*H*_3_CH_2_), 1.08–1.32 (m, 4H, H-7, H-8, H-11, H-14), 1.35–1.50 (m, 1H, H-15), 1.53–1.80 (m, 4H, H-12, CH_3_C*H*_2_), 1.77 (s, 3H, C*H*_3_-C5′), 1.84–1.99 (m, 1H, H-15), 1.91 (s, 3H, C*H*_3_-C3′), 2.12–2.36 (m, 3H, H-7, H-9, H-11), 2.53–2.85 (m, 5H, H-4′, H-6, H-16), 3.68 (s, 3H, OMe), 4.30 and 4.32 (both s, total 1H, C3-O*H*), 6.07 and 6.08 (both s, total 1H, C5′-O*H*), 6.60 (d, *J* = 2.8 Hz, 1H, H-4), 6.66 (dd, *J* = 8.7, 2.8 Hz, 1H, H-2), 7.16 (d, *J* = 8.7 Hz, 1H, H-1). ^13^C NMR (75 MHz, DMSO-*d*_6_) *δ* 12.43 and 12.46 (*C*H_3_CH_2_), 14.64 (*C*H_3_-C3′), 17.85 and 17.88 (CH_3_*C*H_2_), 21.06 (C-18), 22.70 and 22.83 (C-15), 26.91 (C-7), 27.48 (*C*H_3_-C5′), 28.33 (C-11), 30.08 (C-6), 36.70 (C-16), 42.39 (C-12), 42.88 (C-9), 43.40 (C-8), 51.65 (C-14), 54.87 (OMe), 59.02 (C-4′), 71.59 (C-13), 90.43 and 90.47 (C-5′), 111.69 (C-2), 113.04 (C-4), 126.50 (C-1), 131.97 (C-10), 137.38 (C-5), 156.65 (C-3′), 157.07 (C-3), 171.41 and 171.46 (C-17). HRMS (ESI) *m*/*z* [*M* + H]^+^ calcd for [C_26_H_39_N_2_O_4_]^+^: 443.2904, found: 443.2901.

4-Butyl-1-{3-[(1*S*,2*S*,4a*S*,10a*R*)-2-hydroxy-7-methoxy-2-methyl-1,2,3,4,4a,9,10,10a-octahydrophenanthren-1-yl]propanoyl}-3,5-dimethyl-4,5-dihydro-1*H*-pyrazol-5-ol (**3e**). Yield 73%. White solid, mp 69–71 °C, R_f_ = 0.48 (PE:EA = 1:1). ^1^H NMR (600 MHz, DMSO-*d*_6_) *δ* 0.89 (t, *J* = 7.2 Hz, 3H, C*H*_3_CH_2_), 0.97 (s, 3H, H-18), 1.07–1.19 (m, 3H, H-8, H-11, H-14), 1.22–1.60 (m, 9H, H-7, H-12α, H-15, 3 × C*H*_2_), 1.73 (dt, *J* = 12.4, 3.2 Hz, 1H, H-12β), 1.751 and 1.755 (both s, total 3H, C*H*_3_-C5′), 1.83–1.94 (m, 1H, H-15), 1.902 and 1.904 (both s, total 3H, C*H*_3_-C3′), 2.13–2.18 (m, 1H, H-7), 2.19–2.25 (m, 1H, H-9), 2.28–2.33 (m, 1H, H-11), 2.51–2.68 (m, 3H, H-4′, H-16), 2.71–2.82 (m, 2H, H-6), 3.68 (s, 3H, OMe), 4.30 (s, 0.5 H, C13-O*H*), 4.31 (s, 0.5H, C13-O*H*), 6.08 (s, 1H, C5′-O*H*), 6.59 (d, *J* = 2.8 Hz, 1H, H-4), 6.66 (dd, *J* = 8.7, 2.8 Hz, 1H, H-2), 7.16 (d, *J* = 8.6 Hz, 1H, H-1). ^13^C NMR (150 MHz, DMSO-*d*_6_) *δ* 13.82 (*C*H_3_CH_2_), 14.58 (*C*H_3_-C3′), 21.02 (C-18), 22.45 (CH_2_), 22.65 and 22.79 (C-15), 24.27 and 24.31 (*C*H_2_-C4′), 26.86 (C-7), 27.22 (*C*H_3_-C5′), 28.29 (C-11), 29.48 (CH_2_), 30.04 (C-6), 36.66 (C-16), 42.37 (C-12), 42.84 (C-9), 43.36 (C-8), 51.62 (C-14), 54.82 (OMe), 57.48 and 57.51 (C-4′), 71.52 (C-3), 90.38 and 90.41 (C-5′), 111.63 (C-2), 113.00 (C-4), 126.44 (C-1), 131.93 (C-10), 137.33 (C-5), 156.65 (C-3′), 157.02 (C-3), 171.40 and 171.45 (C-17). HRMS (ESI) *m*/*z* [*M* + H]^+^ calcd for [C_28_H_43_N_2_O_4_]^+^: 471.3217, found: 471.3218.

1-{3-[(1*S*,2*S*,4a*S*,10a*R*)-2-Hydroxy-7-methoxy-2-methyl-1,2,3,4,4a,9,10,10a-octahydrophenanthren-1-yl]propanoyl}-3,5-dimethyl-4-(3-methylbutyl)-4,5-dihydro-1*H*-pyrazol-5-ol (**3f**). Yield 76%. White solid, mp 60–62 °C, R_f_ = 0.53 (PE:EA = 1:1). ^1^H NMR (300 MHz, DMSO-*d*_6_) *δ* 0.83–0.92 (m, 6H, 2 × CH_3_), 0.98 (s, 3H, H-18), 1.06–1.65 (m, 11H, H-7, H-8, H-11, H-12, H-14, H-15, C*H*_2_C*H*_2_C*H*Me_2_), 1.67–1.79 (m, 1H, H-12), 1.75 (s, 3H, *C*H_3_-C5′), 1.83–1.99 (m, 1H, H-15), 1.91 (s, 3H, *C*H_3_-C3′), 2.10–2.36 (m, 3H, H-7, H-9, H-11), 2.51–2.85 (m, 5H, H-4′, H-6, H-16), 3.68 (s, 3H, OMe), 4.29 (s, 1H, C13-O*H*), 6.10 (br s, 1H, C5′-O*H*), 6.60 (d, *J* = 2.8 Hz, 1H, H-4), 6.67 (dd, *J* = 8.7, 2.8 Hz, 1H, H-2), 7.17 (d, *J* = 8.6 Hz, 1H, H-1). ^13^C NMR (75 MHz, DMSO-*d*_6_) *δ* 14.64 (*C*H_3_-C3′), 21.03 (C-18), 22.29 (*C*H_3_CH), 22.43 and 22.47 (*C*H_2_-C4′), 22.52 (*C*H_3_CH), 22.68 and 22.80 (C-15), 26.88 (C-7), 27.26 (*C*H_3_-C5′), 27.80 and 27.81 (*C*HMe_2_), 28.30 (C-11), 30.05 (C-6), 36.40 and 36.42 (CH_2_), 36.69 (C-16), 42.38 (C-12), 42.85 (C-9), 43.37 (C-8), 51.62 (C-14), 54.84 (OMe), 57.67 and 57.70 (C-4′), 71.53 (C-3), 90.40 and 90.43 (C-5′), 111.65 (C-2), 113.00 (C-4), 126.46 (C-1), 131.93 (C-10), 137.34 (C-5), 156.67 (C-3′), 157.03 (C-3), 171.39 and 171.44 (C-17). HRMS (ESI) *m*/*z* [*M* + Na]^+^ calcd for [C_29_H_44_N_2_O_4_Na]^+^: 507.3193, found: 507.3193.

4-Hexyl-1-{3-[(1*S*,2*S*,4a*S*,10a*R*)-2-hydroxy-7-methoxy-2-methyl-1,2,3,4,4a,9,10,10a-octahydrophenanthren-1-yl]propanoyl}-3,5-dimethyl-4,5-dihydro-1*H*-pyrazol-5-ol (**3g**). Yield 68%. White solid, mp 75–78 °C, R_f_ = 0.52 (PE:EA = 1:1). ^1^H NMR (500 MHz, DMSO-*d*_6_) *δ* 0.87 (t, *J* = 7.2 Hz, 3H, C*H*_3_CH_2_), 0.98 (s, 3H, H-18), 1.08–1.20 (m, 3H, H-8, H-11, H-14), 1.22–1.33 (m, 8H, H-7, C*H*_2_), 1.33–1.51 (m, 4H, H-15, C*H*_2_), 1.55 (td, *J* = 13.2, 3.5 Hz, 1H, H-12α), 1.72–1.77 (m, 1H, H-12β), 1.750 and 1.754 (both s, total 3H, C*H*_3_-C5′), 1.85–1.96 (m, 1H, H-15), 1.902 and 1.903 (both s, total 3H, C*H*_3_-C3′), 2.13–2.19 (m, 1H, H-7), 2.20–2.26 (m, 1H, H-9), 2.28–2.34 (m, 1H, H-11), 2.51–2.68 (m, 3H, H-4′, H-16), 2.71–2.82 (m, 2H, H-6), 3.69 (s, 3H, OMe), 4.26 (s, 0.5H, C13-O*H*), 4.27 (s, 0.5H, C13-O*H*), 6.01 (s, 0.5H, C5′-O*H*), 6.02 (s, 0.5H, C5′-O*H*), 6.60 (d, *J* = 2.8 Hz, 1H, H-4), 6.67 (dd, *J* = 8.7, 2.8 Hz, 1H, H-2), 7.16 (d, *J* = 8.6 Hz, 1H, H-1). ^13^C NMR (125 MHz, DMSO-*d*_6_) *δ* 13.84 (*C*H_3_CH_2_), 14.51 (*C*H_3_-C3′), 21.00 (C-18), 21.98 (CH_2_), 22.60 and 22.72 (C-15), 24.53 and 24.56 (*C*H_2_-C4′), 26.84 (C-7), 27.15 (CH_2_), 27.19 (*C*H_3_-C5′), 28.24 (C-11), 28.95 (CH_2_), 29.97 (C-6), 31.05 (CH_2_), 36.54 (C-16), 42.35 (C-12), 42.80 (C-9), 43.30 and 43.32 (C-8), 51.60 (C-14), 54.79 (OMe), 57.51 and 57.53 (C-4′), 71.48 (C-13), 90.37 and 90.40 (C-5′), 111.59 (C-2), 112.99 (C-4), 126.37 (C-1), 131.92 (C-10), 137.28 (C-5), 156.58 (C-3′), 157.00 (C-3), 171.38 and 171.43 (C-17). HRMS (ESI) *m*/*z* [*M* + Na]^+^ calcd for [C_30_H_46_N_2_O_4_Na]^+^: 521.3350, found: 521.3345.

4-Allyl-1-{3-[(1*S*,2*S*,4a*S*,10a*R*)-2-hydroxy-7-methoxy-2-methyl-1,2,3,4,4a,9,10,10a-octahydrophenanthren-1-yl]propanoyl}-3,5-dimethyl-4,5-dihydro-1*H*-pyrazol-5-ol (**3h**). Yield 72%. White solid, mp 63–65 °C, R_f_ = 0.42 (PE:EA = 1:1). ^1^H NMR (500 MHz, DMSO-*d*_6_) 0.98 (s, 3H, H-18), 1.09–1.19 (m, 3H, H-8, H-11, H-14), 1.22–1.31 (m, 1H, H-7), 1.37–1.46 (m, 1H, H-15), 1.55 (td, *J* = 13.1, 3.8 Hz, 1H, H-12α), 1.71–1.77 (m, 1H, H-12β), 1.732 and 1.737 (both s, total 3H, C*H*_3_-C5′), 1.84–1.96 (m, 1H, H-15), 1.91 (s, 3H, C*H*_3_-C3′), 2.12–2.19 (m, 1H, H-7), 2.19–2.26 (m, 1H, H-9), 2.26–2.40 (m, 3H, H-11, C*H*_2_-C4′), 2.51–2.66 (m, 2H, H-16), 2.70–2.89 (m, 3H, H-6, H-4′), 3.68 (s, 3H, OMe), 4.33 and 4.34 (both s, total 1H, C13-O*H*), 5.04 (d, *J* = 10.2 Hz, 1H), 5.14 (d, *J* = 17.0 Hz, 1H), 5.86–5.95 (m, 1H), 6.23 (s, 1H, C5′-O*H*), 6.60 (d, *J* = 2.8 Hz, 1H, H-4), 6.67 (dd, *J* = 8.7, 2.8 Hz, 1H, H-2), 7.17 (d, *J* = 8.7 Hz, 1H, H-1). ^13^C NMR (125 MHz, DMSO-*d*_6_) *δ* 14.95 (*C*H_3_-C3′), 21.07 (C-18), 22.71 and 22.85 (C-15), 26.83 (C-7), 26.91 (*C*H_3_-C5′), 28.36 (C-11), 28.97 and 29.03 (*C*H_2_-C4′), 30.11 (C-6), 36.77 (C-16), 42.42 (C-12), 42.90 (C-9), 43.41 and 43.43 (C-8), 51.65 (C-14), 54.88 (OMe), 57.28 (C-4′), 71.59 (C-13), 90.39 and 90.44 (C-5′), 111.71 (C-2), 113.02 (C-4), 116.61 (CH=*C*H_2_), 126.55 (C-1), 131.96 (C-10), 136.75 (*C*H=CH_2_), 137.41 (C-5), 156.29 (C-3′), 157.07 (C-3), 171.47 and 171.52 (C-17). HRMS (ESI) *m*/*z* [*M* + H]^+^ calcd for [C_27_H_39_N_2_O_4_]^+^: 455.2904, found: 455.2898.

4-Benzyl-1-{3-[(1*S*,2*S*,4a*S*,10a*R*)-2-hydroxy-7-methoxy-2-methyl-1,2,3,4,4a,9,10,10a-octahydrophenanthren-1-yl]propanoyl}-3,5-dimethyl-4,5-dihydro-1*H*-pyrazol-5-ol (**3i**). Yield 67%. White solid, mp 59–60 °C, R_f_ = 0.53 (PE:EA = 1:1). ^1^H NMR (300 MHz, DMSO-*d*_6_) *δ* 0.98 (s, 3H, H-18), 1.07–1.31 (m, 4H, H-7, H-8, H-11, H-14), 1.35–1.61 (m, 2H, H-12, H-15), 1.50 (s, 3H, C*H*_3_-C5′), 1.69–1.79 (m, 1H, H-12), 1.84 (s, 3H, C*H*_3_-C3′), 1.85–2.00 (m, 1H, H-15), 2.11–2.36 (m, 3H, H-7, H-9, H-11), 2.51–2.69 (m, 2H, H-16), 2.69–2.99 (m, 4H, H-6, C*H*_2_Ph), 3.10 (t, *J* = 7.5 Hz, 1H, H-4′), 3.68 (s, 3H, OMe), 4.31 (s, 1H, C13-O*H*), 6.32 (br s, 1H, C5′-O*H*), 6.60 (d, *J* = 2.8 Hz, 1H, H-4), 6.66 (dd, *J* = 8.7, 2.8 Hz, 1H, H-2), 7.14–7.23 (m, 2H), 7.24–7.36 (m, 4H). ^13^C NMR (75 MHz, DMSO-*d*_6_) *δ* 14.88 (*C*H_3_-C3′), 21.03 (C-18), 22.68 and 22.79 (C-15), 26.46 (*C*H_3_-C5′), 26.88 (C-7), 28.30 (C-11), 30.05 (2C, C-6 and *C*H_2_Ph), 36.71 and 36.76 (C-16), 42.37 (C-12), 42.85 (C-9), 43.35 and 43.40 (C-8), 51.62 (C-14), 54.84 (OMe), 58.84 (C-4′), 71.54 (C-13), 90.39 and 90.42 (C-5′), 111.66 (C-2), 113.01 (C-4), 125.96 (C-4″), 126.48 (C-1), 128.07 (2CH_Ar_), 129.13 (2CH_Ar_), 131.55 (C-10), 137.36 (C-5), 139.55 (C-1″), 156.12 (C-3′), 157.04 (C-3), 171.58 and 171.64 (C-17). HRMS (ESI) *m*/*z* [*M* + Na]^+^ calcd for [C_31_H_40_N_2_O_4_Na]^+^: 527.2880, found: 527.2864.

4-(4-Fluorobenzyl)-1-{3-[(1*S*,2*S*,4a*S*,10a*R*)-2-hydroxy-7-methoxy-2-methyl-1,2,3,4,4a,9,10,10a-octahydrophenanthren-1-yl]propanoyl}-3,5-dimethyl-4,5-dihydro-1*H*-pyrazol-5-ol (**3j**). Yield 61%. White solid, mp 73–76 °C, R_f_ = 0.44 (PE:EA = 1:1). ^1^H NMR (300 MHz, DMSO-*d*_6_ *δ* 0.97 (s, 3H, H-18), 1.03–1.31 (m, 4H, H-7, H-8, H-11, H-14), 1.32–1.61 (m, 2H, H-12, H-15), 1.47 (s, 3H, C*H*_3_-C5′), 1.69–1.78 (m, 1H, H-12), 1.86 (s, 3H, C*H*_3_-C3′), 1.86–1.97 (m, 1H, H-15), 2.09–2.37 (m, 3H, H-7, H-9, H-11), 2.51–2.68 (m, 2H, H-16), 2.70–2.95 (m, 4H, H-6, C*H*_2_Ph), 3.09 (t, *J* = 7.5 Hz, 1H, H-4′), 3.68 (s, 3H, OMe), 4.30 and 4.32 (both s, total 1H, C13-O*H*), 6.31 and 6.32 (both s, total 1H, C5′-O*H*), 6.60 (d, *J* = 2.8 Hz, 1H, H-4), 6.66 (dd, *J* = 8.7, 2.8 Hz, 1H, H-2), 7.11 (t, *J* = 8.6 Hz, 2H), 7.17 (d, *J* = 8.6 Hz, 1H, H-1), 7.36 (dd, J = 8.6, 5.7 Hz, 2H). ^13^C NMR (75 MHz, DMSO-*d*_6_) *δ* 14.89 (*C*H_3_-C3′), 21.06 (C-18), 22.75 and 22.86 (C-15), 26.57 (*C*H_3_-C5′), 26.92 (C-7), 28.36 (C-11), 29.31 (*C*H_2_Ph), 30.11 (C-6), 36.81 and 36.88 (C-16), 42.42 (C-12), 42.89 (C-9), 43.40 and 43.45 (C-8), 51.65 (C-14), 54.88 (OMe), 58.92 (C-4′), 71.59 (C-13), 90.35 and 90.37 (C-5′), 111.69 (C-2), 113.03 (C-4), 114.78 (d, ^2^*J*_CF_ = 20.7 Hz, 2C, C-3″), 126.55 (C-1), 130.99 and 131.01 (both d, ^3^*J*_CF_ = 7.6 Hz, 2C, C-2″), 131.95 (C-10), 135.62 and 135.65 (both d, ^4^*J*_CF_ = 2.7 Hz, C-1″), 137.39 (C-5), 156.05 (C-3′), 157.06 (C-3), 160.69 (d, ^1^*J*_CF_ = 241.4 Hz, C-4″), 171.60 and 171.66 (C-17). ^19^F NMR (282 MHz, DMSO-*d*_6_) *δ* −117.22 (s), −117.91 (s). HRMS (ESI) *m*/*z* [*M* + Na]^+^ calcd for [C_31_H_39_FN_2_O_4_Na]^+^: 545.2786, found: 545.2778.

4-(4-Chlorobenzyl)-1-{3-[(1*S*,2*S*,4a*S*,10a*R*)-2-hydroxy-7-methoxy-2-methyl-1,2,3,4,4a,9,10,10a-octahydrophenanthren-1-yl]propanoyl}-3,5-dimethyl-4,5-dihydro-1*H*-pyrazol-5-ol (**3k**). Yield 63%. White solid, mp 77–79 °C, R_f_ = 0.44 (PE:EA = 1:1). ^1^H NMR (300 MHz, DMSO-*d*_6_) *δ* 0.98 (s, 3H, H-18), 1.06–1.32 (m, 4H, H-7, H-8, H-11, H-14), 1.33–1.63 (m, 2H, H-12, H-15), 1.49 (s, 3H, C*H*_3_-C5′), 1.69–1.79 (m, 1H, H-12), 1.86 (s, 3H, C*H*_3_-C3′), 1.86–2.01 (m, 1H, H-15), 2.10–2.36 (m, 3H, H-7, H-9, H-11), 2.51–2.66 (m, 2H, H-16), 2.70–2.96 (m, 4H, H-6, C*H*_2_Ph), 3.10 (t, *J* = 7.5 Hz, 1H, H-4′), 3.68 (s, 3H, OMe), 4.28 and 4.29 (both s, total 1H, C13-O*H*), 6.28 and 6.29 (both s, total 1H, C5′-O*H*), 6.60 (d, *J* = 2.8 Hz, 1H, H-4), 6.66 (dd, *J* = 8.7, 2.8 Hz, 1H, H-2), 7.16 (d, *J* = 8.6 Hz, 1H, H-1), 7.31–7.39 (m, 4H). ^13^C NMR (75 MHz, DMSO-*d*_6_) *δ* 14.81 (*C*H_3_-C3′), 21.03 (C-18), 22.69 and 22.79 (C-15), 26.57 (*C*H_3_-C5′), 26.88 (C-7), 28.30 (C-11), 29.47 (*C*H_2_Ph), 30.05 (C-6), 36.69 and 36.75 (C-16), 42.39 (C-12), 42.85 (C-9), 43.34 and 43.38 (C-8), 51.62 (C-14), 54.83 (OMe), 58.69 (C-4′), 71.54 (C-13), 90.30 and 90.32 (C-5′), 111.64 (C-2), 113.01 (C-4), 126.47 (C-1), 127.96 (C-3″), 130.55 (C-4″), 131.05 and 131.07 (C-2″), 131.93 (C-10), 137.35 (C-5), 138.56 and 138.58 (C-1″), 155.87 (C-3′), 157.03 (C-3), 171.57 and 171.62 (C-17). HRMS (ESI) *m*/*z* [*M* + Na]^+^ calcd for [C_31_H_39_ClN_2_O_4_Na]^+^: 561.2491, found: 561.2475.

4-(4-Bromobenzyl)-1-{3-[(1*S*,2*S*,4a*S*,10a*R*)-2-hydroxy-7-methoxy-2-methyl-1,2,3,4,4a,9,10,10a-octahydrophenanthren-1-yl]propanoyl}-3,5-dimethyl-4,5-dihydro-1*H*-pyrazol-5-ol (**3l**). Yield 66%. White solid, mp 80–82 °C, R_f_ = 0.44 (PE:EA = 1:1). ^1^H NMR (300 MHz, DMSO-*d*_6_) *δ* 0.98 (s, 3H, H-18), 1.06–1.32 (m, 4H, H-7, H-8, H-11, H-14), 1.35–1.62 (m, 2H, H-12, H-15), 1.48 (s, 3H, C*H*_3_-C5′), 1.70–1.79 (m, 1H, H-12), 1.83–1.98 (m, 1H, H-15), 1.86 (s, 3H, C*H*_3_-C3′), 2.10–2.37 (m, 3H, H-7, H-9, H-11), 2.51–2.65 (m, 2H, H-16), 2.70–2.91 (m, 4H, H-6, C*H*_2_Ph), 3.10 (t, *J* = 7.5 Hz, 1H, H-4′), 3.68 (s, 3H, OMe), 4.27 and 4.29 (both s, total 1H, C13-O*H*), 6.28 and 6.29 (both s, total 1H, C5′-O*H*), 6.60 (d, *J* = 2.8 Hz, 1H, H-4), 6.67 (dd, *J* = 8.7, 2.8 Hz, 1H, H-2), 7.17 (d, *J* = 8.6 Hz, 1H, H-1), 7.30 (d, *J* = 8.3 Hz, 2H), 7.48 (d, *J* = 8.3 Hz, 2H). ^13^C NMR (75 MHz, DMSO-*d*_6_) *δ* 14.81 (*C*H_3_-C3′), 21.04 (C-18), 22.73 (C-15), 26.60 (*C*H_3_-C5′), 26.89 (C-7), 28.30 (C-11), 29.57 (*C*H_2_Ph), 30.05 (C-6), 36.72 (C-16), 42.39 (C-12), 42.84 (C-9), 43.37 (C-8), 51.63 (C-14), 54.82 (OMe), 58.65 (C-4′), 71.54 (C-13), 90.31 (C-5′), 111.62 (C-2), 113.01 (C-4), 119.01 (C-4″), 126.45, 130.89 (2CH_Ar_), 131.46 (2CH_Ar_), 131.93 (C-10), 137.34 (C-5), 138.99 (C-1″), 155.84 (C-3′), 157.04 (C-3), 171.60 (C-17). HRMS (ESI) *m*/*z* [*M* + Na]^+^ calcd for [C_31_H_39_BrN_2_O_4_Na]^+^: 605.1985, found: 605.1981.

3-(5-Hydroxy-1-{3-[(1*S*,2*S*,4a*S*,10a*R*)-2-hydroxy-7-methoxy-2-methyl-1,2,3,4,4a,9,10,10a-octahydrophenanthren-1-yl]propanoyl}-3,5-dimethyl-4,5-dihydro-1*H*-pyrazol-4-yl)propanenitrile (**3m**). Yield 61%. White solid, mp 68–70 °C, R_f_ = 0.3 (PE:EA = 1:2). ^1^H NMR (300 MHz, DMSO-*d*_6_) *δ* 0.99 (s, 3H, H-18), 1.07–1.34 (m, 4H, H-7, H-8, H-11, H-14), 1.35–1.49 (m, 1H, H-15), 1.49–1.62 (m, 1H, H-12), 1.70–1.81 (m, 1H, H-12), 1.77 (s, 3H, C*H*_3_-C5′), 1.84–2.00 (m, 3H, H-15, C*H*_2_-C4′), 1.93 (s, 3H, C*H*_3_-C3′), 2.11–2.35 (m, 3H, H-7, H-9, H-11), 2.51–2.70 (m, 4H, H-16, C*H*_2_CN), 2.70–2.87 (m, 3H, H-6, H-4′), 3.68 (s, 3H, OMe), 4.28 and 4.29 (both s, total 1H, C13-O*H*), 6.36 and 6.38 (both s, total 1H, C5′-OH), 6.60 (d, *J* = 2.8 Hz, 1H, H-4), 6.66 (dd, *J* = 8.7, 2.8 Hz, 1H, H-2), 7.16 (d, *J* = 8.6 Hz, 1H, H-1). ^13^C NMR (75 MHz, DMSO-*d*_6_) *δ* 14.40 (C-2″), 14.82 (*C*H_3_-C3′), 21.04 (C-18), 21.10 (C-1″), 22.64 and 22.76 (C-15), 26.89 (C-7), 27.28 (*C*H_3_-C5′), 28.31 (C-11), 30.05 (C-6), 36.67 (C-16), 42.39 (C-12), 42.86 (C-9), 43.35 (C-8), 51.61 (C-14), 54.85 (OMe), 56.26 (C-4′), 71.56 (C-13), 90.08 and 90.12 (C-5′), 111.66 (C-2), 113.02 (C-4), 120.32 (CN), 126.48 (C-1), 131.95 (C-10), 137.37 (C-5), 155.08 (C-3′), 157.05 (C-3), 171.33 and 171.38 (C-17). HRMS (ESI) *m*/*z* [*M* + Na]^+^ calcd for [C_27_H_37_N_3_O_4_Na]^+^: 490.2676, found: 490.2674.

Ethyl 3-(5-hydroxy-1-{3-[(1*S*,2*S*,4a*S*,10a*R*)-2-hydroxy-7-methoxy-2-methyl-1,2,3,4,4a,9,10,10a-octahydrophenanthren-1-yl]propanoyl}-3,5-dimethyl-4,5-dihydro-1*H*-pyrazol-4-yl)propanoate (**3n**). Yield 62%. White solid, mp 63–65 °C, R_f_ = 0.42 (PE:EA = 1:2). ^1^H NMR (600 MHz, DMSO-*d*_6_) *δ* 0.974 and 0.977 (both s, total 3H, H-18), 1.08–1.20 (m, 3H, H-8, H-11, H-14), 1.19 (t, *J* = 7.1 Hz, 3H, C*H*_3_CH_2_), 1.23–1.31 (m, 1H, H-7), 1.38–1.46 (m, 1H, H-15), 1.55 (td, *J* = 13.1, 4.0 Hz, 1H, H-12α), 1.71–1.80 (m, 1H, H-12β), 1.738 and 1.742 (both s, total 3H, C*H*_3_-C5′), 1.78–1.84 (m, 2H, C*H*_2_-C4′), 1.85–1.90 (m, 1H, H-15), 1.916 and 1.917 (both s, total 3H, C*H*_3_-C3′), 2.13–2.18 (m, 1H, H-7), 2.19–2.25 (m, 1H, H-9), 2.28–2.33 (m, 1H, H-11), 2.42–2.65 (m, 4H, H-16, C*H*_2_COO), 2.71–2.82 (m, 3H, H-6, H-4′), 3.68 (s, 3H, OMe), 4.07 (q, *J* = 7.1 Hz, 2H, C*H*_2_O), 4.29 (s, 0.5H, C13-O*H*), 4.31 (s, 0.5H, C13-O*H*), 6.26 (br s, 1H, C5′-O*H*), 6.60 (d, *J* = 2.8 Hz, 1H, H-4), 6.66 (dd, *J* = 8.7, 2.8 Hz, 1H, H-2), 7.17 (d, *J* = 8.6 Hz, 1H, H-1). ^13^C NMR (150 MHz, DMSO-*d*_6_) *δ* 14.04 (C*H*_3_CH_2_), 14.39 (*C*H_3_-C3′), 20.05 and 20.09 (*C*H_2_-C4′), 21.00 (C-18), 22.57 and 22.71 (C-15), 26.84 (C-7), 27.19 (*C*H_3_-C5′), 28.23 (C-11), 29.97 (C-6), 31.46 (*C*H_2_COO), 36.53 (C-16), 42.34 (C-12), 42.80 (C-9), 43.29 (C-8), 51.59 (C-14), 54.80 (OMe), 56.56 (C-4′), 59.75 (OCH_2_), 71.48 (C-13), 90.21 and 90.24 (C-5′), 111.60 (C-2), 112.99 (C-4), 126.37 (C-1), 131.92 (C-10), 137.30 (C-5), 155.79 (C-3′), 156.99 (C-3), 171.31 and 171.36 (C-17), 172.48 (COO). HRMS (ESI) *m*/*z* [*M* + Na]^+^ calcd for [C_29_H_42_N_2_O_6_Na]^+^: 537.2935, found: 537.2928.

3,5-Diethyl-1-{3-[(1*S*,2*S*,4a*S*,10a*R*)-2-hydroxy-7-methoxy-2-methyl-1,2,3,4,4a,9,10,10a-octahydrophenanthren-1-yl]propanoyl}-4,5-dihydro-1*H*-pyrazol-5-ol (**3o**). Yield 70%. White solid, mp 56–57 °C, R_f_ = 0.33 (PE:EA = 1:1). ^1^H NMR (600 MHz, DMSO-*d*_6_) *δ* 0.769 and 0.773 (both t, *J* = 7.3 Hz, total 3H, CH_3_), 0.977 and 0.983 (both s, total 3H, H-18), 1.07–1.20 (m, 3H, H-8, H-11, H-14), 1.10 (t, *J* = 7.3 Hz, 3H, CH_3_), 1.23–1.31 (m, 1H, H-7), 1.41–1.49 (m, 1H, H-15), 1.56 (br t, *J* = 12.4 Hz, 1H, H-12α), 1.74 (dt, *J* = 12.4, 3.0 Hz, 1H, H-12β), 1.88–1.96 (m, 1H, H-15), 2.01–2.11 (m, 1H, H-7), 2.15–2.25 (m, 3H, H-9, C*H*_2_CH_3_), 2.27–2.33 (m, 3H, H-11, C*H*_2_CH_3_), 2.44–2.57 (m, 1H, H-16), 2.62–2.80 (m, 4H, H-6, H-16, H-4′), 2.92 (d, *J* = 18.7 Hz, 1H, H-4′), 3.68 (s, 3H, OMe), 4.26 (s, 0.5H, C13-O*H*), 4.29 (s, 0.5H, C13-O*H*), 6.05 (s, 0.5H, C5′-O*H*), 6.06 (s, 0.5H, C5′-O*H*), 6.59 (d, *J* = 2.8 Hz, 1H, H-4), 6.66 (dd, *J* = 8.7, 2.8 Hz, 1H, H-2), 7.15 (d, *J* = 8.6 Hz, 1H, H-1). ^13^C NMR (150 MHz, DMSO-*d*_6_) *δ* 8.81 (CH_3_), 10.27 (CH_3_), 20.97 and 21.02 (C-18), 22.86 and 23.05 (C-15), 23.05 (CH_3_*C*H_2_-C3′), 26.79 and 26.82 (C-7), 28.28 (C-11), 30.04 (C-6), 30.48 and 30.53 (CH_3_*C*H_2_-C5′), 36.55 and 36.76 (C-16), 42.38 (C-12), 42.84 (C-9), 43.26 and 43.28 (C-8), 47.18 and 47.20 (C-4′), 51.68 and 51.78 (C-14), 54.79 (OMe), 71.48 (C-13), 93.35 (C-5′), 111.61 (C-2), 112.98 (C-4), 126.38 (C-1), 131.88 (C-10), 137.27 (C-5), 157.02 (C-3), 157.82 (C-3′), 171.25 and 171.30 (C-17). HRMS (ESI) *m*/*z* [*M* + Na]^+^ calcd for [C_26_H_38_N_2_O_4_Na]^+^: 465.2724, found: 465.2714.

3,5-Diethyl-1-{3-[(1*S*,2*S*,4a*S*,10a*R*)-2-hydroxy-7-methoxy-2-methyl-1,2,3,4,4a,9,10,10a-octahydrophenanthren-1-yl]propanoyl}-4-methyl-4,5-dihydro-1*H*-pyrazol-5-ol (**3p**). Yield 68%. White solid, mp 49–51 °C, R_f_ = 0.57 (PE:EA = 1:1). ^1^H NMR (300 MHz, DMSO-*d*_6_) *δ* 0.76 (t, *J* = 7.2 Hz, 3H, C*H*_3_CH_2_), 0.97 and 0.98 (both s, total 3H, H-18), 1.02 (d, *J* = 7.2 Hz, 3H, *C*H_3_-C4′), 1.07–1.36 (m, 4H, H-7, H-8, H-11, H-14), 1.11 (t, *J* = 7.2 Hz, 3H, C*H*_3_CH_2_), 1.37–1.63 (m, 2H, H-12, H-15), 1.70–1.79 (m, 1H, H-12), 1.84–2.04 (m, 2H, H-7, H-15), 2.14–2.36 (m, 6H, H-9, H-11, 2 × C*H*_2_CH_3_), 2.39–2.58 (m, 1H, H-16), 2.60–2.83 (m, 3H, H-6, H-16), 2.89 (q, *J* = 7.2 Hz, 1H, H-4′), 3.68 (s, 3H, OMe), 4.27 (s, 0.5H, C13-O*H*), 4.28 (s, 0.5H, C13-O*H*), 5.90 (s, 0.5H, C5′-O*H*), 5.92 (s, 0.5H, C5′-O*H*), 6.58 (d, *J* = 2.8 Hz, 1H, H-4), 6.66 (dd, *J* = 8.7, 2.8 Hz, 1H, H-2), 7.15 (d, *J* = 8.6 Hz, 1H, H-1). ^13^C NMR (150 MHz, DMSO-*d*_6_) *δ* 8.69 and 8.72 (CH_3_), 9.40 and 9.45 (CH_3_), 10.03 and 10.06 (CH_3_), 20.99 and 21.06 (C-18), 21.19 (CH_3_*C*H_2_-C3′), 22.92 and 23.25 (C-15), 26.85 (C-7), 28.35 (C-11), 30.14 (C-6), 30.38 (CH_3_*C*H_2_-C5′), 36.71 and 37.03 (C-16), 42.42 (C-12), 42.90 (C-9), 43.28 and 43.33 (C-8), 48.19 and 48.25 (C-4′), 51.70 and 51.86 (C-14), 54.82 (OMe), 71.52 (C-13), 93.35 (C-5′), 111.67 (C-2), 112.97 (C-4), 126.47 (C-1), 131.88 (C-10), 137.30 (C-5), 157.04 (C-3), 161.24 and 161.30 (C-3′), 171.64 and 171.72 (C-17). HRMS (ESI) *m*/*z* [*M* + H]^+^ calcd for [C_27_H_41_N_2_O_4_]^+^: 457.3061, found: 457.3055.

1-{3-[(1*S*,2*S*,4a*S*,10a*R*)-2-Hydroxy-7-methoxy-2-methyl-1,2,3,4,4a,9,10,10a-octahydrophenanthren-1-yl]propanoyl}-3-methyl-5-phenyl-4,5-dihydro-1*H*-pyrazol-5-ol (**3q**). Yield 67%. White solid, mp 82–83 °C, R_f_ = 0.31 (PE:EA = 1:1). ^1^H NMR (300 MHz, DMSO-*d*_6_) *δ* 0.96 and 0.97 (both s, total 3H, H-18), 1.06–1.34 (m, 4H, H-7, H-8, H-11, H-14), 1.35–1.48 (m, 1H, H-15), 1.48–1.61 (m, 1H, H-12), 1.68–1.78 (m, 1H, H-12), 1.78–1.95 (m, 1H, H-15), 2.01 (s, 3H, C*H*_3_-C3′), 2.11–2.36 (m, 3H, H-7, H-9, H-11), 2.55–2.82 (m, 4H, H-6, H-16), 2.96 (d, *J* = 19.0 Hz, 1H, H-4′), 3.10 (d, *J* = 19.0 Hz, 1H, H-4′), 3.69 (s, 3H, OMe), 4.20 (s, 0.5H, C13-O*H*), 4.22 (s, 0.5H, C13-O*H*), 6.57–6.64 (m, 2H, H-4, OH), 6.67 (dd, *J* = 8.7, 2.8 Hz, 1H, H-2), 7.17 (d, *J* = 8.6 Hz, 1H, H-1), 7.20–7.28 (m, 1H), 7.28–7.36 (m, 2H). ^13^C NMR (75 MHz, DMSO-*d*_6_) *δ* 15.72 (*C*H_3_-C3′), 20.99 (C-18), 22.60 (C-15), 26.88 (C-7), 28.30 (C-11), 30.03 (*C*H_2_Ph), 36.26 (C-16), 42.39 (C-12), 42.85 (C-9), 43.28 (C-8), 51.54 and 51.66 (C-14), 54.83 (OMe), 55.32 (C-4′), 71.51 (C-13), 91.82 (C-5′), 111.64 (C-2), 113.00 (C-4), 124.38 (2CH_Ar_), 126.46 (C-1), 126.85 (C-4″), 127.88 (2CH_Ar_), 131.92 (C-10), 137.33 (C-5), 144.37 (C-1″), 153.32 and 153.37 (C-3′), 157.03 (C-3), 170.10 and 170.17 (C-17). HRMS (ESI) *m*/*z* [*M* + Na]^+^ calcd for [C_29_H_36_N_2_O_4_Na]^+^: 499.2567, found: 499.2570.

5-(4-Fluorophenyl)-1-{3-[(1*S*,2*S*,4a*S*,10a*R*)-2-hydroxy-7-methoxy-2-methyl-1,2,3,4,4a,9,10,10a-octahydrophenanthren-1-yl]propanoyl}-3-methyl-4,5-dihydro-1*H*-pyrazol-5-ol (**3r**). Yield 70%. White solid, mp 43–44 °C, R_f_ = 0.38 (PE:EA = 1:1). ^1^H NMR (600 MHz, DMSO-*d*_6_) *δ* 0.96 and 0.97 (both s, total 3H, H-18), 1.07–1.19 (m, 3H, H-8, H-11, H-14), 1.24–1.33 (m, 1H, H-7), 1.38–1.46 (m, 1H, H-15), 1.50–1.59 (m, 1H, H-12), 1.71–1.76 (m, 1H, H-12), 1.81–1.89 (m, 1H, H-15), 2.01 (s, 3H, C*H*_3_-C3′), 2.12–2.18 (m, 1H, H-7), 2.19–2.25 (m, 1H, H-9), 2.27–2.34 (m, 1H, H-11), 2.56–2.73 (m, 2H, H-16), 2.73–2.84 (m, 2H, H-6), 2.99 (d, *J* = 19.2 Hz, 1H, H-4′), 3.09 (d, *J* = 19.2 Hz, 1H, H-4′), 3.68 (s, 3H, OMe), 4.18 (s, 0.5H, C13-O*H*), 4.25 (s, 0.5H, C13-O*H*), 6.60 (d, *J* = 2.8 Hz, 1H, H-4), 6.65–6.70 (m, 2H, H-2, C5′-OH), 7.13 (t, *J* = 8.3 Hz, 2H, H-3″), 7.16 (d, *J* = 8.7 Hz, 1H, H-1), 7.33–7.38 (m, 2H, H-2″). ^13^C NMR (150 MHz, DMSO-*d*_6_) *δ* 15.60 (*C*H_3_-C3′), 20.95 and 20.98 (C-18), 22.50 (C-15), 26.84 (C-7), 28.22 (C-11), 29.94 (C-6), 36.09 and 36.12 (C-16), 42.36 (C-12), 42.78 (C-9), 43.21 (C-8), 51.51 and 51.60 (C-14), 54.79 (OMe), 55.13 (C-4′), 71.46 and 71.49 (C-13), 90.86 and 91.37 (C-5′), 111.58 (C-2), 112.99 (C-4), 114.40 (d, ^2^*J*_CF_ = 21.3 Hz, 2C, C-3″), 126.34 (C-1), 126.50 (d, ^3^*J*_CF_ = 8.1 Hz, 2C, C-2″), 131.92 (C-10), 135.58 (d, ^4^*J*_CF_ = 2.7 Hz, C-1″), 137.27 (C-5), 153.25 and 153.28 (C-3′), 157.01 (C-3), 161.10 (d, ^1^*J*_CF_ = 242.6 Hz, C-4″), 170.19 and 170.25 (C-17). ^19^F NMR (564 MHz, DMSO-*d*_6_) *δ* -116.45 (s), -116.46 (s). HRMS (ESI) *m*/*z* [*M* + H]^+^ calcd for [C_29_H_36_FN_2_O_4_]^+^: 495.2654, found: 495.2650.

5-(4-Bromophenyl)-1-{3-[(1*S*,2*S*,4a*S*,10a*R*)-2-hydroxy-7-methoxy-2-methyl-1,2,3,4,4a,9,10,10a-octahydrophenanthren-1-yl]propanoyl}-3-methyl-4,5-dihydro-1*H*-pyrazol-5-ol (**3s**). Yield 85%. White solid, mp 86–88 °C, R_f_ = 0.48 (PE:EA = 1:1). ^1^H NMR (500 MHz, DMSO-*d*_6_) *δ* 0.96 and 0.97 (both s, total 3H, H-18), 1.08–1.20 (m, 3H, H-8, H-11, H-14), 1.24–1.34 (m, 1H, H-7), 1.38–1.47 (m, 1H, H-15), 1.51–1.59 (m, 1H, H-12), 1.71–1.78 (m, 1H, H-12), 1.81–1.90 (m, 1H, H-15), 2.01 (s, 3H, C*H*_3_-C3′), 2.11–2.18 (m, 1H, H-7), 2.19–2.26 (m, 1H, H-9), 2.27–2.34 (m, 1H, H-11), 2.56–2.70 (m, 2H, H-16), 2.70–2.84 (m, 2H, H-6), 3.00 (d, *J* = 18.9 Hz, 1H, H-4′), 3.08 (d, *J* = 18.9 Hz, 1H, H-4′), 3.69 (s, 3H, OMe), 4.16 (s, 0.5H, C13-O*H*), 4.22 (s, 0.5H, C13-O*H*), 6.60 (d, *J* = 2.8 Hz, 1H, H-4), 6.67 (dd, *J* = 8.7, 2.8 Hz, 1H, H-2), 6.71 (s, 0.5H, C5′-O*H*), 6.73 (s, 0.5H, C5′-O*H*), 7.16 (d, *J* = 8.7 Hz, 1H, H-1), 7.28 (d, *J* = 8.4 Hz, 2H), 7.50 (d, *J* = 8.4 Hz, 2H). ^13^C NMR (125 MHz, DMSO-*d*_6_) *δ* 15.63 (*C*H_3_-C3′), 20.96 and 20.99 (C-18), 22.52 (C-15), 26.85 (C-7), 28.25 (C-11), 29.96 (*C*H_2_Ph), 36.14 (C-16), 42.37 (C-12), 42.80 (C-9), 43.22 (C-8), 51.49 and 51.59 (C-14), 54.79 (OMe), 55.04 (C-4′), 71.47 (C-13), 90.66 and 91.25 (C-5′), 111.59 (C-2), 112.99 (C-4), 119.97 (C-4″), 126.38 (C-1), 126.84 (2CH), 130.65 (2CH), 131.92 (C-10), 137.29 (C-5), 143.76 (C-1″), 153.31 and 153.36 (C-3′), 157.02 (C-3), 170.08 and 170.14 (C-17). HRMS (ESI) *m*/*z* [*M* + Na]^+^ calcd for [C_29_H_35_BrN_2_O_4_Na]^+^: 577.1672, found: 577.1667.

1-{3-[(1*S*,2*S*,4a*S*,10a*R*)-2-Hydroxy-7-methoxy-2-methyl-1,2,3,4,4a,9,10,10a-octahydrophenanthren-1-yl]propanoyl}-3-methyl-5-(trifluoromethyl)-4,5-dihydro-1*H*-pyrazol-5-ol (**3t**). Yield 71%. White solid, mp 74–76 °C, R_f_ = 0.5 (PE:EA = 1:1). ^1^H NMR (600 MHz, DMSO-*d*_6_) *δ* 0.98 (s, 3H, H-18), 1.08–1.20 (m, 3H, H-8, H-11β, H-14), 1.24–1.32 (m, 1H, H-7), 1.42–1.49 (m, 1H, H-15), 1.56 (br t, *J* = 12.5 Hz, 1H, H-12α), 1.74 (dt, *J* = 12.5, 3.1 Hz, 1H, H-12β), 1.86–1.93 (m, 1H, H-15), 1.99 (s, 3H, C*H*_3_-C3′), 2.09–2.15 (m. 1H, H-7), 2.19–2.26 (m, 1H, H-9), 2.31 (dq, *J* = 12.8, 3.6 Hz, 1H, H-11α), 2.58–2.70 (m, 2H, H-16), 2.71–2.82 (m, 2H, H-6), 3.04 (d, *J* = 19.3 Hz, 1H, H-4′), 3.39 (d, *J* = 19.3 Hz, 1H, H-4′), 3.68 (s, 3H, OMe), 4.24 (br s, 1H, C13-O*H*), 6.60 (d, *J* = 2.8 Hz, 1H, H-4), 6.67 (dd, *J* = 8.7, 2.8 Hz, 1H, H-2), 7.16 (d, *J* = 8.7 Hz, 1H, H-1), 7.61 (s, 0.5 H, C5′-O*H*), 7.63 (s, 0.5H, C5′-O*H*). ^13^C NMR (150 MHz, DMSO-*d*_6_) *δ* 15.24 (*C*H_3_-C3′), 20.97 (C-18), 22.53 (C-15), 26.87 (C-7), 28.23 (C-11), 29.97 (C-6), 36.74 and 36.79 (C-16), 42.36 and 42.38 (C-12), 42.82 (C-9), 43.15 and 43.22 (C-8), 47.81 (C-4′), 51.50 and 51.55 (C-14), 54.83 (OMe), 71.55 (C-13), 90.28 (q, ^2^*J*_CF_ = 35.6 Hz, C-5′), 111.63 (C-2), 113.02 (C-4), 123.33 (q, ^1^*J*_CF_ = 286.8 Hz, *C*F_3_), 126.42 (C-1), 131.94 (C-10), 137.35 (C-5), 153.41 (C-3′), 157.05 (C-3), 171.41 and 171.49 (C-17). HRMS (ESI) *m*/*z* [*M* + H]^+^ calcd for [C_24_H_32_F_3_N_2_O_4_]^+^: 469.2309, found: 469.2307.

### 2.3. Biology

#### 2.3.1. Evaluation of Antiproliferative Activity

The antiproliferative properties of the synthesized compounds **3a**–**t** were assessed in vitro on MCF-7 and T47D hormone-responsive breast carcinoma cell lines. The MCF-7 cells, obtained from ATCC, were maintained in Dulbecco’s Modified Eagle Medium (DMEM) (PanEco, Moscow, Russia) supplemented with 10% fetal bovine serum (FBS; HyClone), 50 U/mL penicillin, and 50 µg/mL streptomycin (PanEco). T47D (ATCC) were cultured in RPMI 1640 (PanEco) supplemented with 10% FBS, 50 U/mL penicillin, 50 µg/mL streptomycin, and a vitamin complex (PanEco). All cells were incubated at 37 °C in a humidified atmosphere containing 5% CO_2_, 85–90% relative humidity in an NU-5840E incubator (NuAire Inc., Plymouth, MN, USA).

Antiproliferative activity was determined via an MTT assay protocol [87], which relies on the reduction of MTT (3-(4,5-dimethylthiazol-2-yl)-2,5-diphenyltetrazolium bromide) into insoluble purple formazan crystals by viable cells. Compounds were initially dissolved in DMSO (AppliChem GmbH, Darmstadt, Germany) to a stock concentration of 10 mM and stored at –20 °C. For testing, cells were seeded into 24-well plates (Corning Inc., Corning, NY, USA), and after 24 h, test compounds were added at concentrations ranging from 0.05 to 50 µM. Cisplatin (CDDP) and doxorubicin, as well as two taxane drugs (docetaxel and paclitaxel), included in current clinical guidelines for breast cancer therapy [88,89,90], served as reference cytotoxic agents. Control groups received equivalent volumes of DMSO, ensuring that the final solvent concentration did not exceed 0.5%.

Following 72 h exposure to the compounds, the medium was discarded, and fresh medium containing the MTT reagent (AppliChem) was added. After 1 h of incubation, the reagent was removed, and DMSO was added to solubilize the formazan crystals. Absorbance was measured at 571 nm using a MultiSkan FC microplate reader (Thermo Fisher Scientific, Waltham, MA, USA). Background absorbance (from blank wells) was subtracted, and the absorbance of untreated control cells was taken as 100%. Half-maximal inhibitory concentration (IC_50_) values were calculated in GraphPad Prism 7.0 using a four-parameter logistic regression model (log[inhibitor] vs. normalized response, variable slope).

All experiments were performed in triplicate. Data are presented as mean ± standard deviation (SD).

#### 2.3.2. Immunoblot Analysis

MCF-7 cells were seeded into 100 mm culture dishes (Corning Inc., Corning, NY, USA) and allowed to adhere for 24 h. Subsequently, compounds **3f**, **3j**, and **3k** were introduced in fresh medium, and cells were incubated for an additional 24 h. Post treatment, cells were rinsed twice with phosphate-buffered saline (PBS) and harvested. Cell lysis was performed on ice for 10 min using a modified buffer containing 50 mM Tris-HCl (pH 7.5), 1% Igepal CA-630, 150 mM NaCl, 1 mM EDTA, 1 mM DTT, 1 mM sodium orthovanadate, 1 mM sodium fluoride, and a protease inhibitor cocktail (bestatin, leupeptin, and pepstatin at 1 µg/mL each), as previously reported [91]. Protein concentration was quantified by the Bradford assay [92].

Equal amounts of protein were separated by SDS-PAGE on 10% gels under reducing conditions and transferred onto nitrocellulose membranes (GE HealthCare Inc., Chicago, IL, USA). Membranes were blocked with 5% non-fat dry milk in TBS buffer (20 mM Tris-HCl, pH 7.5; 150 mM NaCl) containing 0.1% Tween-20. Primary antibody incubation was carried out overnight at 4 °C.

Primary antibodies against androgen receptor (AR), estrogen receptor α (ERα), GREB1, S6K, and its phosphorylated form (p-S6K), Bcl-2, and its phosphorylated form (p-Bcl-2), and α-tubulin were employed (Cell Signaling Technology, Danvers, MA, USA). α-Tubulin served as the internal loading control. HRP-conjugated secondary goat anti-rabbit antibodies (Jackson ImmunoResearch, West Grove, PA, USA) were used for detection via enhanced chemiluminescence (ECL), following the method of Mruk and Cheng [93], using an ImageQuant LAS4000 imaging system (GE HealthCare).

#### 2.3.3. Toxicity Evaluation of Selected Compounds

The cytotoxicity of the selected compounds was evaluated against normal mammary epithelial cells MCF-10A (ATCC) using the resazurin viability assay by standard preclinical method [94]. Cells (5.5 × 10^4^/well) were seeded into 24-well plates in 900 μL of medium and treated with compounds **3f**, **3j**, and **3k** in 100 μL the next day. Cells were incubated for 72 h, followed by the addition of resazurin (0.017 mg/mL, 2.5 h) in serum-free medium. The fluorescence of the medium samples (130 µL) was measured spectrophotometrically (SpectraMax, Ex.: 560 nm/Em.: 590 nm) and IC_50_ values were calculated using GraphPad Prism 7.0. (Boston, MA, USA).

#### 2.3.4. Cell Cycle Analysis by Flow Cytometry

Cell cycle distribution was analyzed by DNA content measurement as described [95]. Briefly, MCF-7 cells were seeded in 6 mm Petri dishes and treated with compound **3j** or vehicle (DMSO) for 72 h. Following treatment, cells were collected, washed with PBS (PanEco, Moscow, Russia), and stained with a solution containing Propidium Iodide (PI, 50 µg/mL), RNase A (100 µg/mL), sodium citrate (0.1% in PBS), and Igepal CA-630 (0.3% in PBS; Sigma-Aldrich, Saint Louis, MO, USA) for 30 min at 4 °C in the dark. After incubation with the solution, samples were immediately analyzed on a CytoFLEX flow cytometer (Beckman Coulter, Indianapolis, IN, USA), measuring PI fluorescence in the PerCP channel (excitation: 488 nm/emission: 680 nm). A minimum of 10,000 single-cell events were acquired per sample. Cell cycle distribution (G1/G0, S, and G2/M phases, subG1 fraction, and polyploids) was determined by modeling DNA content histograms using GraphPad Prism 7.0 (Boston, MA, USA).

#### 2.3.5. Determination of the Synergistic Effect of the Lead Compounds in Therapeutic Combinations

ERα-positive breast cancer cells MCF-7 were seeded in 24-well plates (Corning) at a density of 4 × 10^4^ cells/well. After 24 h of incubation, one of the lead compounds, **3j**, along with chemotherapeutic drugs currently used in clinical practice to treat different forms of breast cancer, was added to the cells alone or in combinations based on the respective IC_50_ values from the cytotoxicity assay. We determined each drug’s maximal concentration to kill 80% of cells when taken as a monotherapy. Serial dilutions of the calculated IC_80_ value were used to acquire the remaining concentrations of the test substances. The MTT test, as previously mentioned, was used to colorimetrically evaluate cell viability 72 h after drug administration. The HSA model [96], which entails determining such concentrations at which the expected combined effect will surpass the effect of using the drugs separately, was used to compute the synergistic scopes (SS) of the combined use of chemotherapeutic drugs with the test compound **3j** using the open-source program SynergyFinder+ (https://synergyfinder.org (assessed on 20 June 2025)). Heat maps were made to evaluate the therapeutic significance of drug combinations based on the values derived from the model. In this instance, red patches show a synergistic impact within the chosen concentration range, and the therapeutic relevance of these areas grows in direct proportion to the rise in color intensity and numerical values.

### 2.4. Molecular Docking

Molecular docking calculations were carried out with Smina software (Smina version 15 October 2019) [97] using Vinardo scoring function [98]. The initial approximate 3D geometry of each ligand molecule was quantum-chemically optimized with MOPAC [99] employing the PM7 basis [100]. The geometry optimization was performed using the eigenvector following (EF) algorithm with the highest recommended precision (SCFCRT = 1.D–16; GNORM = 0.01 kcal/mol). The optimized 3D geometry was then converted into mol2 format using the Open Babel toolbox (v4.2.1) [101] and further processed with AutoDockTools4 [102]. Gasteiger partial atomic charges were calculated and assigned to the atoms according to the standard procedure. Nonpolar hydrogens and their charges were combined with the corresponding carbon atoms, whereas hydrogens bound to heteroatoms were retained explicitly to allow proper modeling interactions via hydrogen bonds. AutoDock Vina atom types were then assigned, and a rotatable bond tree was generated as a key step in defining ligand flexibility. Each ligand was subsequently saved in PDBQT file for docking simulations. The 3D coordinates of the protein targets Bcl-2 (PDB ID: 2W3L) and S6K (PDB ID: 3WF8) were obtained from the RCSB Protein Data Bank.

## 3. Results and Discussion

### 3.1. The Synthesis of the Secosteroid–2-Pyrazoline Hybrids

**Scheme 1:**
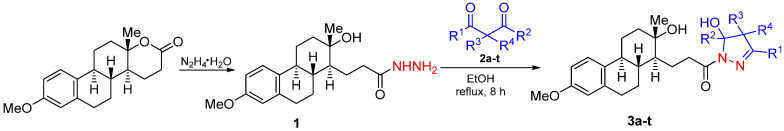
Synthesis of secosteroid–2-pyrazoline hybrid compounds **3a**–**t**.

The synthetic procedure for secosteroid–2-pyrazoline hybrids is outlined in Scheme 1. The starting 13α-hydroxy-3-methoxy-13,17-secoestra-1,3,5(10)-trien-17-oic acid hydrazide **1** was synthesized by the ring D opening in 13α-3-methoxy-17a-oxa-17a-homoestra-1,3,5(10)-trien-17-one with an excess of hydrazine hydrate in boiling ethanol [70]. Hydrazide **1** was then refluxed with diketones **2a**–**t** in ethanol [103] to obtain secosteroid–2-pyrazoline hybrids **3a**–**t** in good yield (Table 1). The consumption of the starting material was monitored by TLC. We found that the high conversion of the starting material was achieved after 8 h under reflux, and the TLC indicated product **3** as one spot. TLC analysis and ^1^H NMR spectra of both crude mixtures and purified products revealed that compounds **3a**–**t** are inseparable mixtures of two compounds with a ratio of 1:1 (See Supplementary Materials File S1). Two products appeared in all ring closure reactions to form secosteroid–pyrazoline derivatives **3a**–**t**, and their ratio was independent of the substituents in the pyrazoline moiety. Since the reaction results in the formation of a new stereocenter at the C5′ position of the pyrazoline ring, we assume that two diastereomers with *dr* = 1:1 were formed [63]. However, the difference between the distinct signals in the NMR spectra is so small (e.g., 0.001 ppm for C*H*_3_-C5′ and 0.02–0.07 ppm for certain carbon atoms in compound **3a**) that it is impossible to draw a conclusion about the correspondence of the signals to a specific diastereomer.

**Figure 2:**
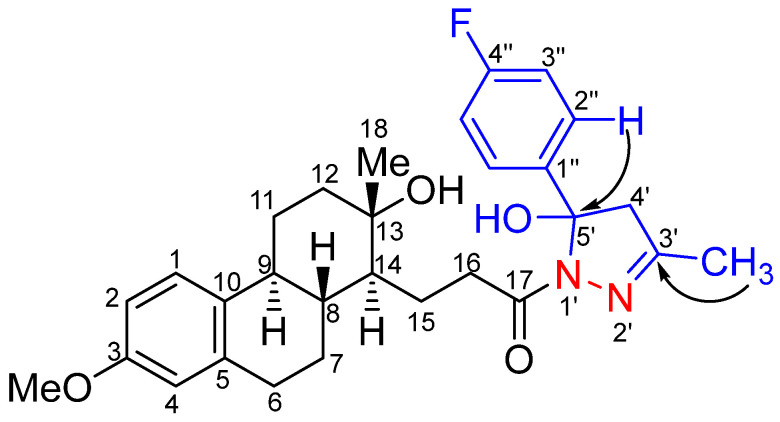
Atom numbering for compounds **3** and key HMBC correlations for compound **3r**.

The structure of all obtained compounds **3a**–**t** was determined based on ^1^H and ^13^C NMR spectra, including 2D correlation techniques and HRMS measurements. The fact of heterocycle formation was confirmed by the disappearance of the singlets of the NH group (8.98 ppm) and NH_2_ group (4.16 ppm) of the starting hydrazide; instead, signals appeared at 1.70–1.75 ppm (C*H*_3_-C5′), 1.89–1.93 (C*H*_3_-C3′) and ca. 6.0 (C5′-O*H*) (Figure 2). In the case of hybrids with 4′-benzyl substituents, the signals of the methyl group attached to C5′ are shifted upfield to 1.47–1.50 ppm. For 4′-unsubstituted compounds, two characteristic doublets of magnetically nonequivalent methylene protons at the 4′-position (2.8–3.3 ppm) with *J* ca. 19 Hz were observed. The ^13^C NMR spectra of compounds **3** confirmed the presence of the pyrazoline ring, revealing signals at 153–161, 47–57, and 90–91 ppm associated with the carbon atoms C3′, C4′, and C5′ of the heterocyclic fragment, respectively. The structure of the unsymmetrical compounds **3q**–**t** was confirmed by C*H*_3_-C3′ → C3′ and *H*-2″ (Ar) → C5′ HMBC correlations; for the hybrid **3t**, the C5′ carbon atom signal appeared at 90.28 as q, ^2^*J*_CF_ = 35.6 Hz. The C-18 methyl of the secosteroid skeleton appeared in ^1^H NMR spectra as a singlet at 0.96–1.00 ppm.

### 3.2. Evaluation of Cytotoxicity Against Two Breast Cancer Cell Lines

The antiproliferative activity of the synthesized compounds **3a**–**t** was assessed using the MTT colorimetric assay on the human estrogen-dependent breast carcinoma cell line MCF-7, utilizing cytostatic drugs from various pharmacological groups as reference, including taxanes (paclitaxel and docetaxel), anthracyclines (doxorubicin), and the alkylating agent cisplatin (CDDP). The corresponding IC_50_ values after 72 h incubation are summarized in Table 2.

Our initial investigation focused on the analysis of the impact of different substituents R^3^ in the 4-position of 3,5-dimethyl-2-pyrazoline moiety. A closer look into the structure–activity relationship suggests that the cytotoxic potency was highly dependent on the substitution types and patterns on the phenyl ring. The parent 4-unsubstituted hybrid **3a** showed moderate activity. However, upon introduction of an alkyl substituent into the 4-position of pyrazoline, the cytotoxicity of hybrids **3** increases significantly and passes through a maximum in the case of compound **3f** with R^3^ = *iso*-amyl (IC_50_ = 0.32 μM) (Table 2, entry 6). Interestingly, the hybrid compound **3n** with R^3^ = ethoxycarbonyl exhibits significantly greater activity compared to the compound **3m** with R^3^ = cyanoethyl. These results suggested that a definite length of the substituent at the 4-position of pyrazoline may contribute to the antiproliferative effect of this class of compounds. Similarly, the replacement of the hydrogen with the benzyl group attached to the 4-position of the pyrazoline resulted in a strong activity increase. All compounds **3i**–**l** with different 4-benzyl substituents were highly effective in inhibiting MCF-7 cancer cell growth with IC_50_ = 0.22–0.73 μM, which was probably caused by favorable steric interactions of the bulky hydrophobic groups with the binding site. Moreover, the results clearly indicate that the halogenated benzyl substituent attached to the 4-position of the pyrazoline ring provides higher activity against the MCF-7 cancer cell line, and the best results were obtained for the fluoro-substituted phenyl group. Hence, the biological response increased more for halogenated analogs than for their non-halogenated motifs. A similar trend was also observed among compounds **3q–s**, having a phenyl group in the 5-position of the pyrazoline ring. Comparable results were obtained previously for steroid–5-arylpyrazoline hybrids; this is probably due to enhanced lipophilicity, pharmacokinetic properties, physicochemical properties, endurance for metabolic destruction, and electronegativity [50,62,63,64,65]. It should be mentioned that the incorporation of an aromatic moiety into the secosteroidal hydrazide derivatives enhances the cytotoxicity that was previously reported for secosteroid–1,2,4-triazole and secosteroid–1,3,4-oxadiazole hybrids [71,72,73]. Increasing the length of the alkyl substituents in positions 3 and 5 of the pyrazoline cycle (compounds **3o** and **3p**), as well as introducing a second substituent in the 4-position (compound **3c**), significantly reduces the cytotoxic activity of the hybrids. Hybrid **3t**, bearing a trifluoromethyl group in the 5-position of the pyrazoline ring, also displayed only moderate antiproliferative potential against the MCF-7 tumor cell line.

**Figure 3:**
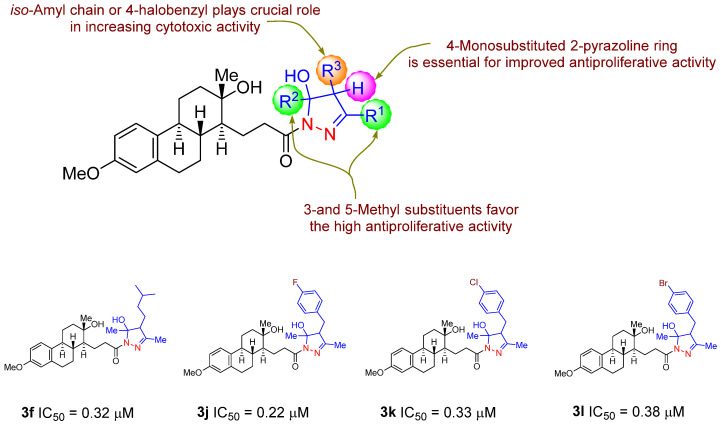
SAR and the most potent compounds **3f**, **3j**, **3k**, **3l**.

Overall, the synthesized secosteroid–pyrazoline hybrid compounds **3a**–**t** exhibited moderate to very high activity against estrogen-responsive human MCF-7 breast cancer cells. Notably, the four lead compounds—**3f**, **3j**, **3k**, and **3l**—demonstrated remarkable IC_50_ values ranging from 0.22 to 0.38 µM (Figure 3). Thus, the antiproliferative activity of the lead compounds was comparable to that of doxorubicin and up to 25 times higher than the activity of cisplatin.

In addition to the MCF-7 cell line, we used T47D cells as a model for the luminal A subtype of breast cancer to screen the resulting compounds. Their defining characteristic is a more pronounced expression of progesterone receptors compared to MCF-7, making them an indispensable tool for studying hormone signaling pathways and more accurately assessing the efficacy of novel cytotoxic compounds and, more importantly, targeted therapies. Using an additional cell model expressing the protein target of interest allows us to avoid certain pitfalls associated with experimental design and formulate conclusions, including those related to the mechanism of action, based on more comprehensive data.

Table 2 reveals that the previously selected lead compounds **3f**, **3k**, and especially **3j** demonstrate similar half-maximal inhibitory concentrations. The data obtained indicate a common mechanism of action in both cell lines, which may be related, among other things, to the inhibition of key players in apoptosis and hormonal regulation, among which estrogen and androgen receptors play a significant role.

### 3.3. Analysis of Signaling Pathways, Assessment of Cell Cycle Progression and Evaluation of Toxicity

One of the main areas of pharmaceutical research in modern oncology is the development of highly selective drugs that have a specific effect on the main targets in malignant tumors. One of these molecular targets is the hormone receptors responsible for steroidogenesis, cell survival, and proliferation in hormone-dependent malignancies like prostate and breast cancer. These types of cancer can be effectively treated with hormone receptor modulators [104,105] or steroidogenesis enzyme inhibitors [106], which have demonstrated a favorable efficacy and safety profile and are currently used as first-line therapy [107]. Additionally, one study has emphasized the importance of crucial co-regulators such as GREB1 (growth regulating estrogen receptor binding 1), the most estrogen-enriched ERα interactor, which functions as a chromatin-bound co-activator to stabilize ERα interactions with other co-factors like p300 and CBP, making it necessary for ERα-mediated transcription and tumor growth in breast cancer models [108]. In addition to being induced by estrogen, GREB1 expression is a potential biomarker and therapeutic target because it predicts favorable clinical outcomes in ERα-positive cancers.

**Figure 4:**
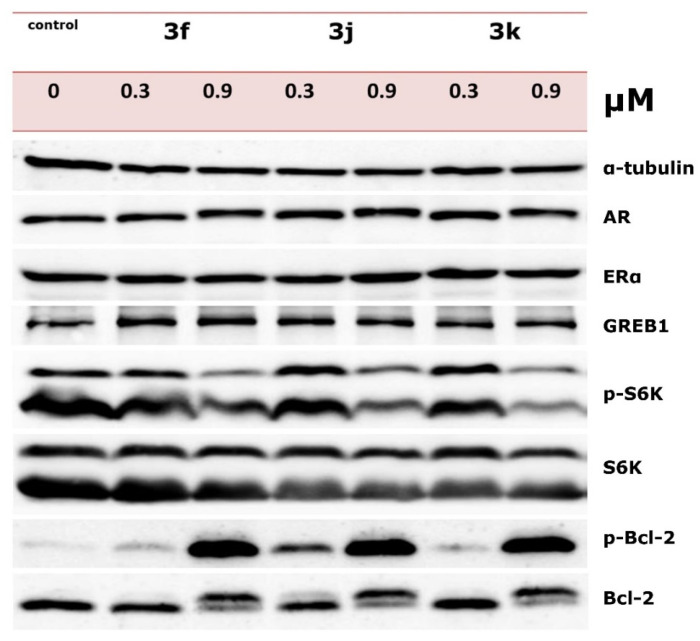
Effect of lead secosteroid–2-pyrazoline hybrids **3f**, **3j**, and **3k** on signaling pathways in MCF-7 breast cancer cells. Antibodies against androgen receptor (AR), estrogen receptor α (ERα), GREB1, S6K, and its phosphorylated form (p-S6K), Bcl-2, and its phosphorylated form (p-Bcl-2), and α-tubulin were used for immunoblotting.

Given the significant therapeutic potential of synthetic steroid derivatives, we investigated the effects of synthesized secosteroid–2-pyrazoline hybrids on the molecular pathways of ERα+ breast cancer cells MCF-7 (Figure 4), including targeting hormone receptors and GREB1 protein, a key estrogen-responsive gene that regulates cell proliferation. The results of the Western blot analysis indicate that none of the tested compounds has inhibitory activity against androgen and estrogen receptors, and they do not have antiproliferative activity due to the downregulation of GREB1.

Serine/threonine kinase S6 (S6K) is one of the most important effectors of the mTORC1 complex, a component of the Ras-activated MAP kinase pathway [109,110]. The phosphorylated (activated) form of S6K plays an important role in the growth and progression of tumor cells, since it is capable, in turn, of activating multiple molecular targets, including the ribosomal protein S6, as well as key regulators of translation initiation, such as eIF4B and PDCD4 [111,112]. Although no specific inhibitors of S6K have been developed to date, it is considered a promising molecular target for the development of next-generation antitumor drugs. As can be seen from Figure 4, the studied secosteroid–2-pyrazoline hybrids exert a suppressive effect on the expression of both S6 kinase and its phosphorylated form (p-S6K). Compounds **3j** and **3k** at a concentration of 0.9 μM demonstrate the most pronounced inhibitory effect on p-S6K, which can be considered as one of the mechanisms of action of this class of derivatives that negatively regulate protein synthesis and growth of MCF-7 breast cancer cells.

Another important approach to inhibiting tumor cell growth may be targeting the developed molecules to the Bcl-2 protein, a key regulator of apoptosis. It localizes to the outer mitochondrial membrane, where it prevents cell death by inhibiting the activation of pro-apoptotic proteins like Bax and Bak, thereby preserving mitochondrial integrity and blocking the release of cytochrome C [113]. In cancers, overexpression of Bcl-2 is a common event; that is why this molecular agent seemed to be a good target for the effective treatment of malignancies. In addition to direct inhibition of the expression of anti-apoptotic Bcl-2, which has been described for many chemotherapeutic drugs being developed and used in practice, there is an alternative approach to negative regulation of the activity of this protein [114,115]. Notably, paclitaxel and other microtubule-targeting agents like vincristine can induce phosphorylation of Bcl-2 [116,117]. Thus, activation of its phosphorylation leads to accumulation of the inactive form (p-Bcl-2) and a decrease in the protective function of the original protein.

Activation of Bcl-2 phosphorylation and the accumulation of its inactive form, which is a marker of increased apoptosis, may represent another mechanism of action for the studied derivatives. A significant increase in p-Bcl-2 expression is characteristic of all selected secosteroid–pyrazoline hybrids (Figure 4), with the most pronounced effect observed for molecule **3j**, which promotes Bcl-2 phosphorylation at a lower dose (0.3 μM).

Evaluation of the selectivity of new compounds is one of the key steps in preclinical research. For this purpose, primary cultures or immortalized cell lines are commonly used (for example, MCF-10A and MCF-12A, representing mammary epithelium; HDF, representing skin fibroblasts; and, in rare cases, human embryonic kidney cells HEK293) [118,119,120,121,122]. The MCF-10A line is one of the most widely used models of normal mammary epithelium. It was derived from fibrocystic breast tissue, is documented as non-tumorigenic, has been extensively characterized, and is available from several cell collections. For these reasons, MCF-10A is frequently used for toxicological comparisons with tumor cell lines (such as MCF-7, MDA-MB-231, and T47D).

**Figure 5:**
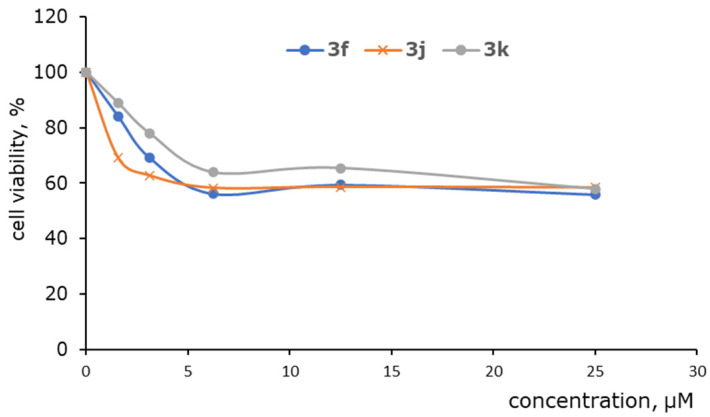
Viability of epithelial cells treated with compounds **3f**, **3j**, and **3k**. Toxic effects of compounds on the MCF-10A cell line were assessed using the resazurin assay.

MCF-10A cells were incubated with the lead compounds **3f**, **3j**, and **3k** for 72 h, after which cytotoxic effects were assessed using the resazurin assay. At low concentrations, the selected molecules did not exert toxic effects towards MCF-10A cells. Increasing the concentration above 10 μM resulted in 35–45% cell death; however, the IC_50_ values for all compounds exceeded 25 μM. These findings indicate low toxicity of the selected hybrid molecules (Figure 5).

Drugs used in the treatment of malignant diseases often exhibit high toxicity, primarily because antiproliferative agents target rapidly dividing cells. As a result, achieving selectivity between malignant cells and normal cells is a challenging task. In our previous study, we observed low selectivity for doxorubicin, which induced pronounced cell death in both breast cancer cells and fibroblasts [73]. Importantly, the selected secosteroids **3f**, **3j**, and **3k** demonstrate excellent selectivity towards tumor cells and cause minimal damage to normal MCF-10A epithelial cells.

Based on the data obtained during the assessment of the antiproliferative potential of new steroid derivatives, hybrid **3j** was chosen as a lead compound for further studies, including the analysis of the cell cycle and the study of possible therapeutic combinations.

**Figure 6:**
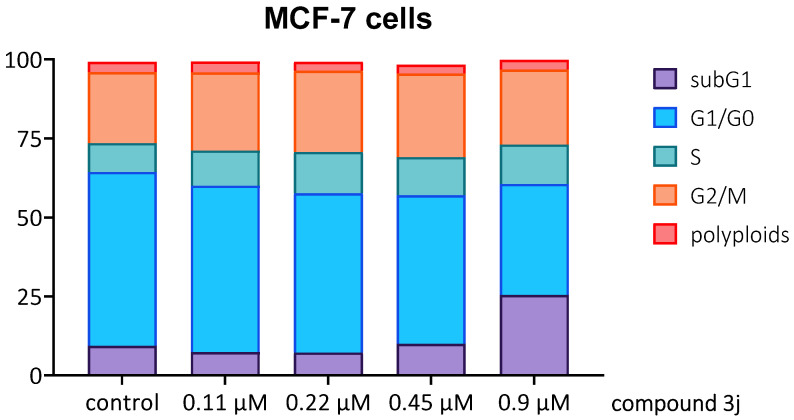
Dose-dependent effect of compound **3j** on MCF-7 cell cycle distribution. Cells were treated with 0.11, 0.22, 0.45, or 0.9 μM of compound **3j** or an equivalent volume of vehicle (DMSO) for 72 h.

To investigate the antiproliferative effects of the compound **3j**, we analyzed the cell cycle distribution of MCF-7 cells after a 72 h treatment. Flow cytometric analysis demonstrated in Figure 6 reveals a dose-dependent shift in the cell cycle profile of the cell population. A significant decrease in the proportion of cells in the G1/G0 phase was observed with increasing concentrations of the compound, compared to the vehicle control (from 55% to 35%). This reduction in the G1/G0 population was not accompanied by concomitant changes in the S or G2/M phases, which remained stable across the tested dose range. At the highest concentration of 0.9 µM compound **3j**, an increase in the subG1 population was also detected (Figure 6). Together, these results indicate that the primary effect of the compound **3j** is a cell death induction accompanied by a reduction of the G1/G0 phase in MCF-7 cells.

### 3.4. Molecular Docking

Since Western blot analysis results revealed a modulatory effect of the lead compounds **3f**, **3j**, and **3k** on Bcl-2/p-Bcl-2 and S6K/p-S6K, molecular docking was performed on these two targets, Bcl-2 and S6K. The X-ray crystal structures of Bcl-2 domain in complex with 1-(2-([(3S)-3-(aminomethyl)-3,4-dihydroisoquinolin-2(1H)-yl]carbonyl)phenyl)-4-chloro-5-methyl-N,N-diphenyl-1H-pyrazole-3-carboxamide (DRO) (PDB code 2W3L) [123] and S6K domain in complex with 2-oxo-2-[(4-sulfamoylphenyl)amino]ethyl 7,8,9,10-tetrahydro-6H-cyclohepta[b]quinoline-11-carboxylate (F76) (PDB code 3WF8) [124] were chosen as target proteins for docking studies. The choice of these complexes was due to some structural similarity of their native ligands DRO and F76 with the compounds under investigation.

**Figure 7:**
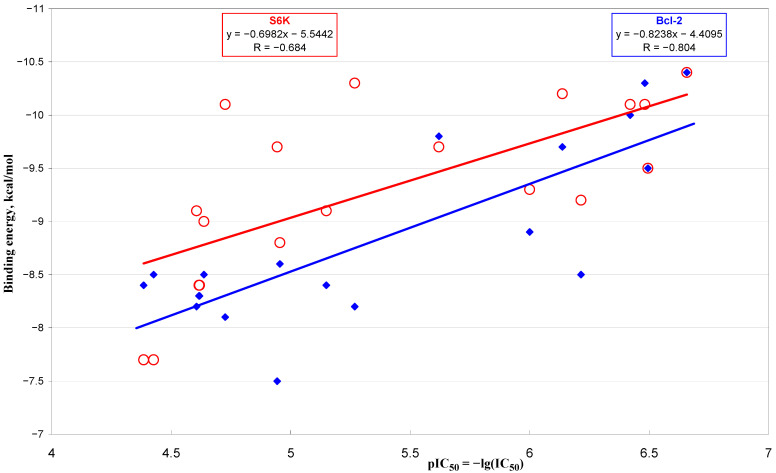
Correlations between docking score and cytotoxicity for compounds **3a**–**t**. Blue color (squares) corresponds to Bcl-2; red color (circles) corresponds to S6K.

**Figure 8:**
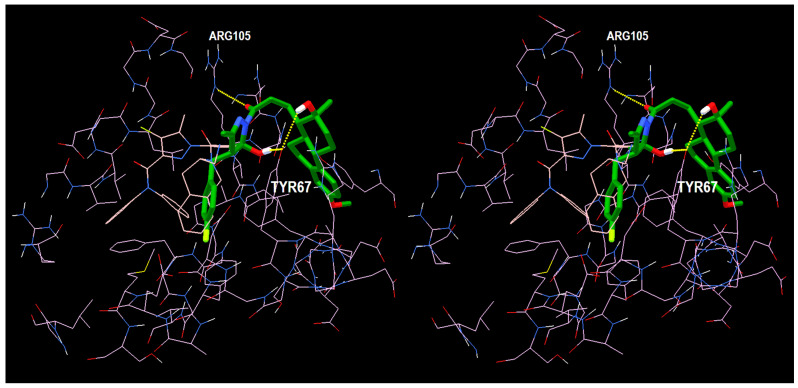
Stereo cross-view of a docking pose for **3j** (green sticks) within the Bcl-2 active site. The residues of the 2W3L are depicted in magenta. The original ligand, DRO, is shown as an orange wireframe. Yellow dotted lines show possible hydrogen bonds.

The docking results revealed that the estimated binding energies for the compounds **3a**–**t** with Bcl-2 ranged from −7.5 to −10.4 kcal/mol (Table 3). Figure 7 (in blue) demonstrates some correlation between estimated binding energy and cytotoxicity. The best result (−10.4 kcal/mol) was observed for the most active compound **3j**, which is more negative compared to the native ligand DRO (−8.9 kcal/mol). The docking pose for the compound **3j** at the binding sites of Bcl-2 is shown in Figure 8. The 4-fluorobenzyl moiety occupies a hydrophobic cavity on the left, while the tricyclic secosteroid moiety is located inside another hydrophobic cavity on the right. These two cavities were denoted by the authors [123] as ‘first-site’ and ‘second-site’ binding sub-pockets; both are important for binding pro-apoptotic proteins like Bak. In addition to these key interactions, the protein–ligand complex can be stabilized by hydrogen bonds with TYR67 and ARG105.

**Figure 9:**
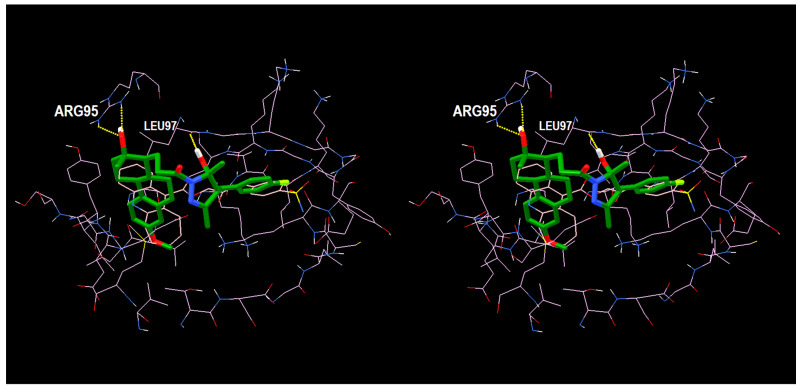
Stereo cross-view of a docking pose for **3j** (green sticks) within the S6K active site. The residues of the 3WF8 are depicted in magenta. The original ligand, F76, is shown as an orange wireframe. Yellow dotted lines show possible hydrogen bonds.

The estimated binding energies for compounds **3a**–**t** with S6K are in approximately the same range (Table 3). Overall, the binding energy estimates are less negative than for the native ligand F76 (−11.8 kcal/mol); however, the docking results indicate that some compounds can bind efficiently to the S6K active site. Although the correlation with cytotoxicity is not as pronounced as for Bcl-2, the trend remains (Figure 7, in red). Again, the most active secosteroid hybrid **3j** exhibits the best binding energy (−10.4 kcal/mol). The docking pose for the compound **3j** at the binding site of S6K is shown in Figure 9. The secosteroid and 4-fluorobenzyl moieties correspond approximately to the tricyclic and phenylsulfonamide moieties of the native F76 ligand, respectively. Moreover, the protein–ligand complex is stabilized by hydrogen bonds with ARG95 residue and the backbone carbonyl of LEU97.

### 3.5. Development of Therapeutic Combinations

As part of assessing the efficacy of using synthesized secosteroid–2-pyrazoline hybrids in combination therapy for ERα-positive breast cancer, therapeutic pairs consisting of the test compound **3j** and standard chemotherapeutic drugs in different concentrations were developed and tested in vitro.

**Figure 10:**
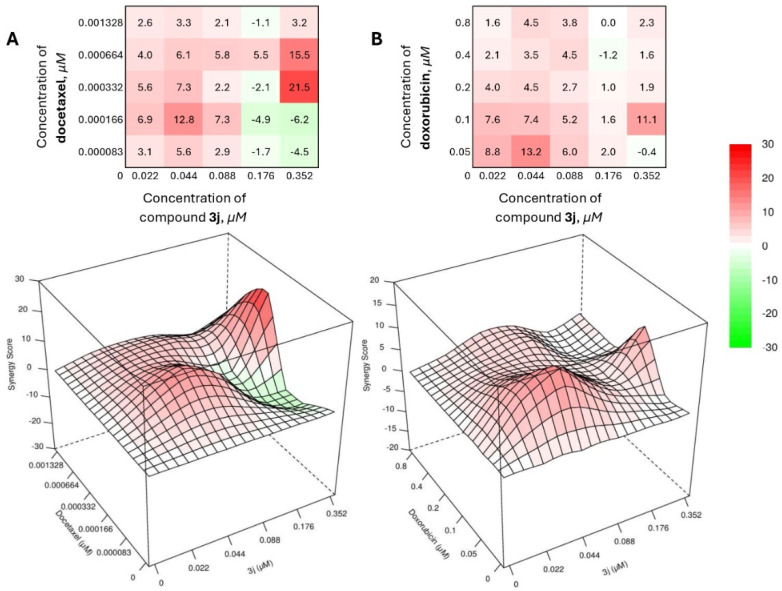
Combined effect of the synthesized compound **3j** with docetaxel (**A**) and doxorubicin (**B**) on the MCF-7 cell line.

The most successful combinations were steroid **3j** with docetaxel (Figure 10A) and doxorubicin (Figure 10B), considered as adjuvant chemotherapy (both individually, in the case of docetaxel, and as part of the DC and AC regimens combined with cyclophosphamide) for the treatment of luminal breast cancer.

The greatest value in the development of effective therapeutic combinations is those pairs of drugs whose concentrations, when used together, are minimal. Upon analyzing the heat maps and 3D visualizations of the data obtained using the open web resource SynergyFinder+ 3.0 [125,126], the synergy score (SS) value matrix for docetaxel and **3j** combinations, as well as doxorubicin and **3j**, there are regions that emerge as particularly appealing in this context. In the case of combinations with docetaxel, three therapeutic pairs with the greatest synergistic effect can be identified, with one of them located in the zone of the lowest concentrations of both drugs (0.044 + 0.000166 μM, SS = 12.8). The other area reflects the combined use of docetaxel at a concentration of 0.000664 μM and **3j** at a concentration that causes 80% death of MCF-7 cells when used individually (0.000664 + 0.352 μM, SS = 15.5). In the case of halving docetaxel concentration (0.000332 μM), it is possible to achieve an increase in the synergistic effect by 1.4 times (SS = 21.5). Also, a reliable synergistic effect is achieved with the combined use of 0.044 μM **3j** with doxorubicin at its minimum concentration (0.05 μM), while the SS value is 13.2.

## 4. Conclusions

In conclusion, we have discovered secosteroid–2-pyrazoline hybrids as novel anti-breast cancer agents, and their simple and efficient synthesis has been successfully developed. Most of the synthesized compounds were found to exhibit remarkable cytotoxicity against hormone-responsive human breast cancer cells MCF-7 and T47D. Key structural motifs contributing to enhanced antiproliferative activity were identified, including substituents in the pyrazoline moiety. The antiproliferative activity of the lead hybrids **3f**, **3j**, and **3k** with IC_50_ values of 0.2–0.5 μM was comparable to doxorubicin and up to 25 times higher than the activity of cisplatin. Furthermore, the lead compounds exhibited minimal toxicity to normal epithelial cells, indicating excellent selectivity. Mechanistic investigations suggested that pro-apoptotic effects of secosteroid–2-pyrazoline hybrids are likely mediated through a suppressive effect on the expression of both S6 kinase and its phosphorylated form (p-S6K) as well as modulation of Bcl-2 protein and its phosphorylated form expression. Apoptosis accompanied by a reduction of the G1/G0 phase was observed in MCF-7 cells treated with compound **3j**. Promising secosteroid–2-pyrazoline hybrids can be further modified to develop clinically useful analogs. The potent antiproliferative activity and high selectivity of these hybrids may serve as a basis for the development of innovative anticancer treatments.

## Figures and Tables

**Table 1:** Scope of synthesized secosteroid–2-pyrazoline hybrid compounds **3a**–**t**.

Product	Structure of Compound	Product Yield, %
**3a**	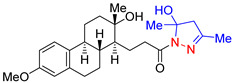	74
**3b**	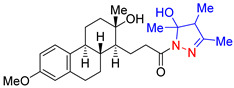	67
**3c**	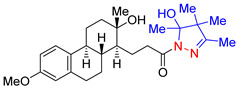	62
**3d**	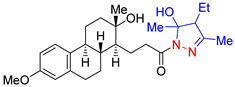	63
**3e**	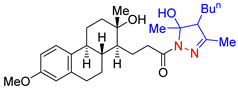	73
**3f**	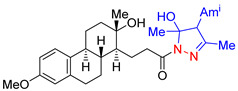	76
**3g**	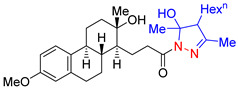	68
**3h**	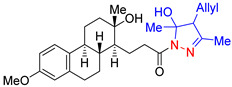	72
**3i**	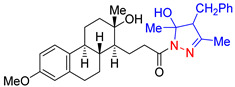	67
**3j**	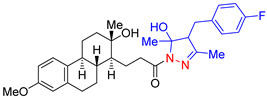	61
**3k**	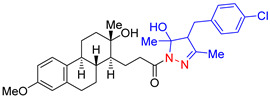	63
**3l**	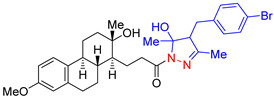	66
**3m**	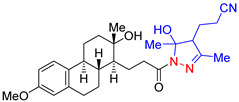	61
**3n**	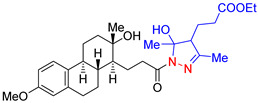	62
**3o**	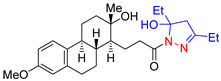	70
**3p**	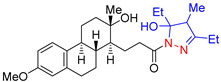	68
**3q**	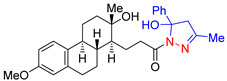	67
**3r**	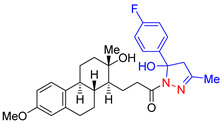	70
**3s**	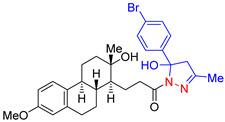	85
**3t**	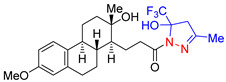	71

**Table 2:** IC_50_ values ^1^ for the synthesized hybrid compounds **3a**–**t**.

Entry	Compound	IC_50_ for MCF-7 Cells (Mean ± SD), µM	IC_50_ for T47D Cells (Mean ± SD), µM
1	**3a**	24.2 ± 2.3	16.6 ± 1.5
2	**3b**	23.1 ± 2.3	22.1 ± 2.2
3	**3c**	41.3 ± 5.0	21.7 ± 2.2
4	**3d**	24.1 ± 2.1	20.9 ± 2.1
5	**3e**	0.61 ± 0.07	1.0 ± 0.9
6	**3f**	0.32 ± 0.03	0.51 ± 0.05
7	**3g**	2.4 ± 0.3	7.7 ± 0.7
8	**3h**	7.1 ± 0.8	14.7 ± 1.5
19	**3i**	0.73 ± 0.09	0.82 ± 0.07
10	**3j**	0.22 ± 0.02	0.28 ± 0.03
11	**3k**	0.33 ± 0.03	0.50 ± 0.04
12	**3l**	0.38 ± 0.04	1.1 ± 0.1
13	**3m**	11.1 ± 1.2	6.9 ± 0.07
14	**3n**	1.0 ± 0.09	3.9 ± 0.04
15	**3o**	>50	33.3 ± 2.9
16	**3p**	37.5 ± 3.6	16.0 ± 1.5
17	**3q**	18.8 ± 1.7	9.6 ± 0.08
18	**3r**	5.4 ± 0.6	7.7 ± 0.06
19	**3s**	11.4 ± 1.2	15.5 ± 1.4
20	**3t**	24.8 ± 2.5	19.7 ± 2.0
21	cisplatin (CDDP)	6.2 ± 0.7	13.1 ± 2.1
22	doxorubicin	0.30 ± 0.03	0.15 ± 0.03
23	docetaxel	0.0015 ± 0.0002	0.0011 ± 0.0003
24	paclitaxel	0.0049 ± 0.0001	0.0068 ± 0.0009

^1^ Data from three independent experiments.

**Table 3:** Estimated binding energies of synthesized compounds **3a**–**t** with Bcl-2 and S6K.

Entry	Compound	Bcl-2 Estimated Binding Energy, kcal mol^−1^	S6K Estimated Binding Energy, kcal mol^−1^
1	**3a**	−8.3	−8.4
2	**3b**	−8.5	−9.0
3	**3c**	−8.4	−7.7
4	**3d**	−8.3	−8.4
5	**3e**	−8.5	−9.2
6	**3f**	−9.5	−9.5
7	**3g**	−9.8	−9.7
8	**3h**	−8.4	−9.1
19	**3i**	−9.7	−10.2
10	**3j**	−10.4	−10.4
11	**3k**	−10.3	−10.1
12	**3l**	−10	−10.1
13	**3m**	−8.6	−8.8
14	**3n**	−8.9	−9.3
15	**3o**	−8.1	−9.0
16	**3p**	−8.5	−7.7
17	**3q**	−8.1	−10.1
18	**3r**	−8.2	−10.3
19	**3s**	−7.5	−9.7
20	**3t**	−8.2	−9.1
22	DRO	−8.9	
23	F76		−11.8

## Data Availability

All the data are contained in the manuscript and Supplementary Materials.

## Supplementary Materials

The following supporting information can be downloaded at: https://www.mdpi.com/article/10.3390/biomedicines13123057/s1. File S1: NMR and HRMS spectra of compounds.
