# Supplementary material for: Secosteroid–2-Pyrazoline Hybrids: Design, Synthesis, Biological Evaluation and Development of Therapeutic Combinations Against ERα-Positive Breast Cancer Cells
Source: Biomedicines. 2025 Dec 11;13(12):3057. doi: 10.3390/biomedicines13123057 (PMC12731114; doi:10.3390/biomedicines13123057)

Supporting Information

Secosteroid – 2-Pyrazoline Hybrids: Design, Synthesis, Biological Evaluation and Development of Therapeutic Combinations against ERα-positive Breast Cancer Cells

Alexey I. Ilovaisky [1,*], Alexander M. Scherbakov [2,3], Fedor B. Bogdanov [2], Dumitru Miciurov [1], Elena I. Chernoburova [1], Valentina M. Merkulova [1], Eugene I. Bozhenko [1], Andrey S. Dmitrenok [1], Diana I. Salnikova [1,2], Danila V. Sorokin [2], Alvina I. Khamidullina [1,4], Mikhail A. Krasil'nikov [2], Igor V. Zavarzin [1] and Alexander O. Terent'ev [1,*]

[1] N. D. Zelinsky Institute of Organic Chemistry, Russian Academy of Sciences, Leninsky Prospekt 47, 119991 Moscow, Russia; dimon.dimon284@gmail.com (D.M.); chernoburova@mail.ru (E.I.C.); merk68@mail.ru (V.M.M.); dr_bozhenko@mail.ru (E.I.B.); dmt@ioc.ac.ru (A.S.D.), igorzavarzin@yandex.ru (I.V.Z.)

[2] N.N. Blokhin National Medical Research Center of Oncology, Kashirskoye shosse 24, Moscow 115522, Russia; a.sherbakov@ronc.ru (A.M.S.); f.bogdanov.f@yandex.ru (F.B.B.); d.salnikova@ronc.ru (D.I.S.); d.sorokin@ronc.ru (D.V.S.); krasilnikovm1@yandex.ru (M.A.K.)

[3] Gause Institute of New Antibiotics, Bol'shaya Pirogovskaya ulitsa 11, Moscow 119021, Russia

[4] Institute of Gene Biology, Russian Academy of Sciences, Moscow, ulitsa Vavilova 34/5, Moscow 119334, Russia; alvina@genebiology.ru (A.I.K.)

***** Correspondence: ilov@ioc.ac.ru (A.I.I.); terentev@ioc.ac.ru (A.O.T.)

List of Contents

NMR spectra of compounds **3a-t**	S2-S68
HRMS spectra of compounds **3a-t**	S69-S88

^{1}H NMR (DMSO-d$_6$, 600 MHz) spectrum of 1-{3-[(1*S*,2*S*,4a*S*,10a*R*)-2-hydroxy-7-methoxy-2-methyl-1,2,3,4,4a,9,10,10a-octahydrophenanthren-1-yl]propanoyl}-3,5-dimethyl-4,5-dihydro-1*H*-pyrazol-5-ol **(3a)**

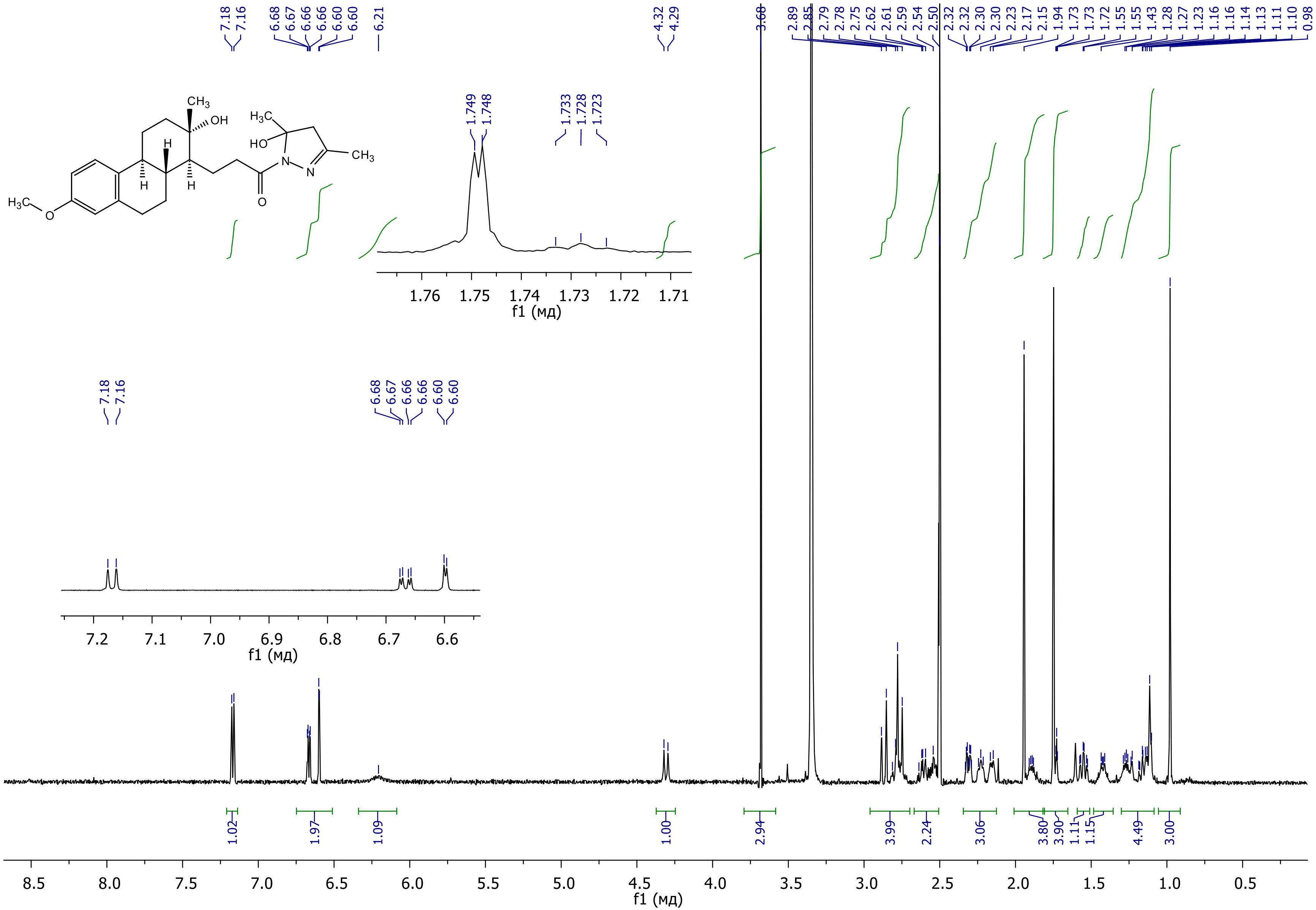

^{13}C NMR (DMSO-d$_6$, 150 MHz) spectrum of 1-{3-[(1*S*,2*S*,4a*S*,10a*R*)-2-hydroxy-7-methoxy-2-methyl-1,2,3,4,4a,9,10,10a-octahydrophenanthren-1-yl]propanoyl}-3,5-dimethyl-4,5-dihydro-1*H*-pyrazol-5-ol **(3a)**

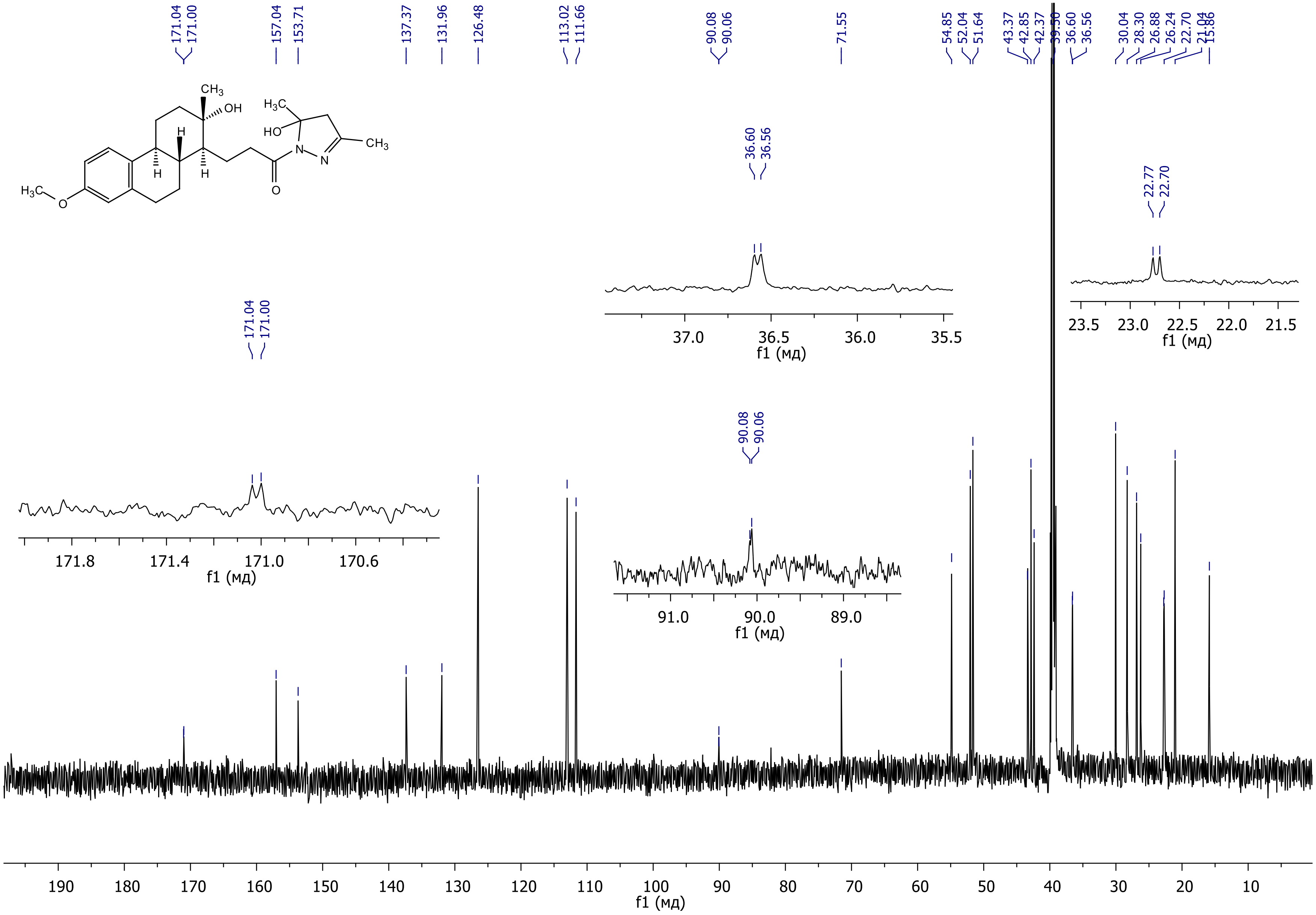

^{1}H – ^{1}H COSY (DMSO-d$_6$, 600 MHz) spectrum of 1-{3-[(1*S*,2*S*,4a*S*,10a*R*)-2-hydroxy-7-methoxy-2-methyl-1,2,3,4,4a,9,10,10a-octahydrophenanthren-1-yl]propanoyl}-3,5-dimethyl-4,5-dihydro-1*H*-pyrazol-5-ol **(3a)**

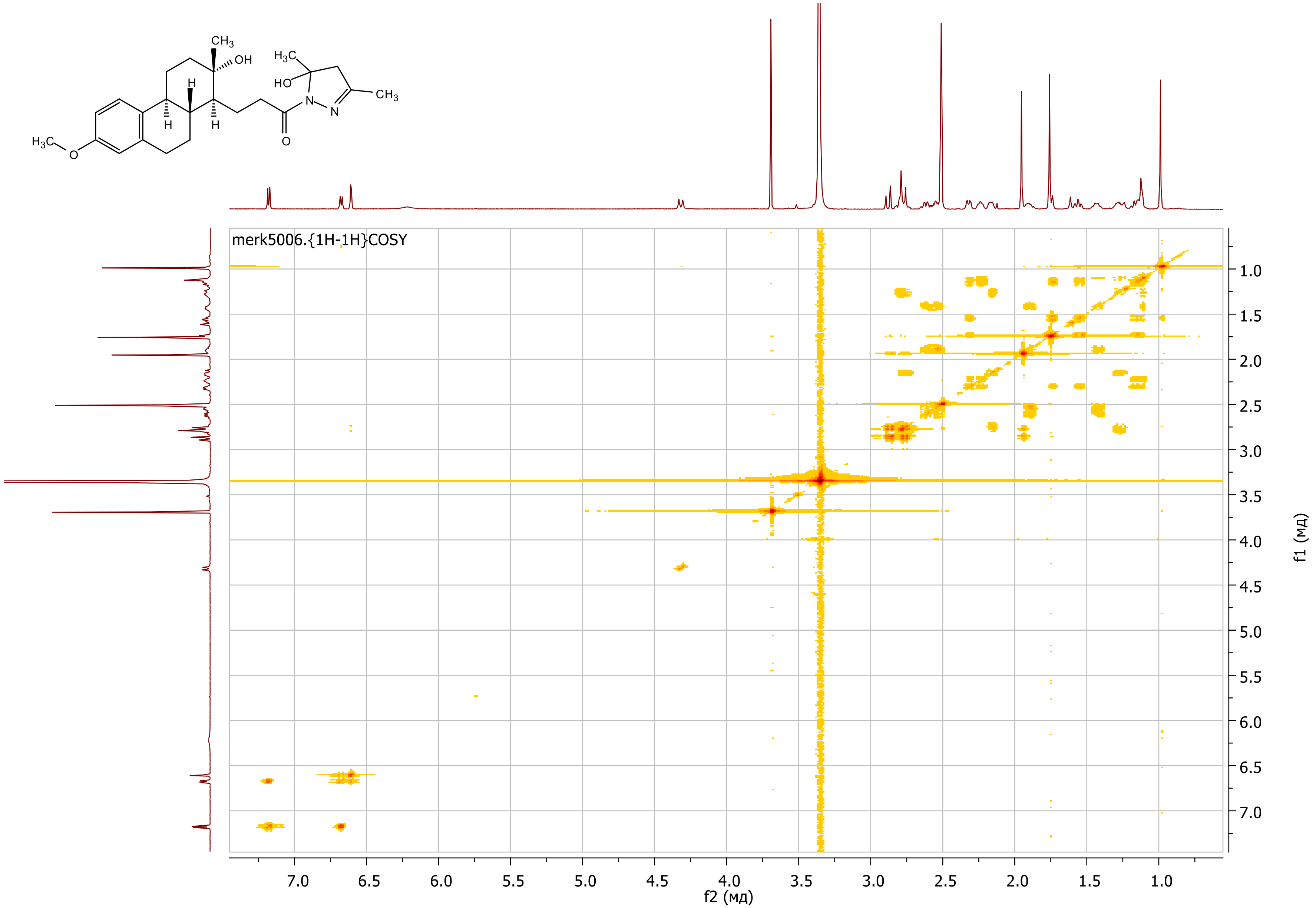

^{1}H – ^{13}C HSQC (DMSO-d$_6$, 600 MHz, 150 MHz) spectrum of 1-{3-[(1*S*,2*S*,4a*S*,10a*R*)-2-hydroxy-7-methoxy-2-methyl-1,2,3,4,4a,9,10,10a-octahydrophenanthren-1-yl]propanoyl}-3,5-dimethyl-4,5-dihydro-1*H*-pyrazol-5-ol **(3a)**

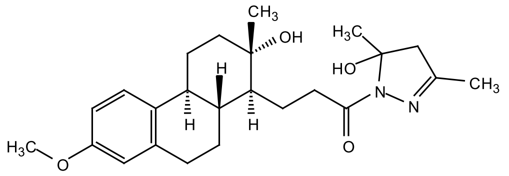

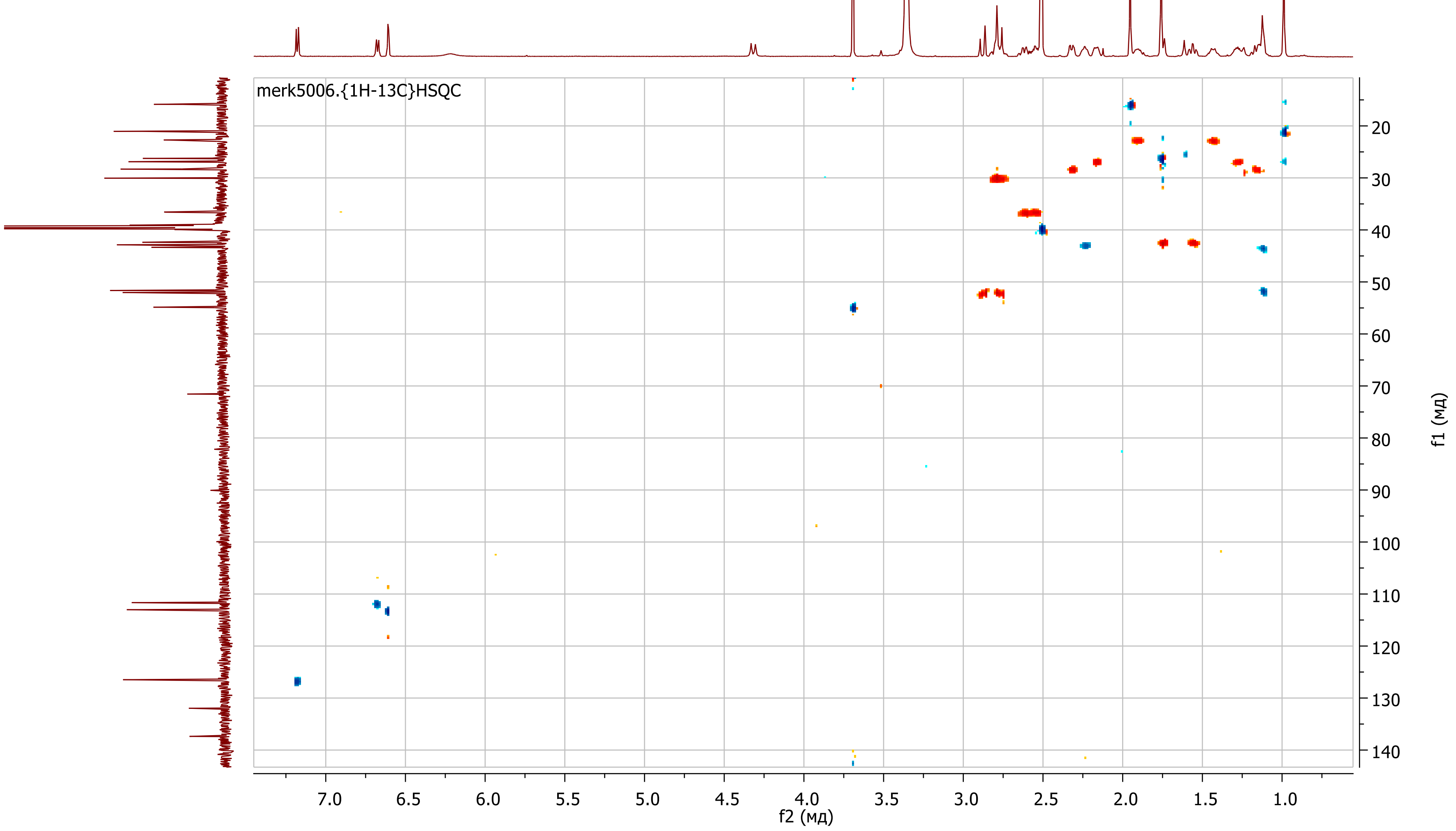

^{1}H – ^{13}C HMBC (DMSO-d$_6$, 600 MHz, 150 MHz) spectrum of 1-{3-[(1*S*,2*S*,4a*S*,10a*R*)-2-hydroxy-7-methoxy-2-methyl-1,2,3,4,4a,9,10,10a-octahydrophenanthren-1-yl]propanoyl}-3,5-dimethyl-4,5-dihydro-1*H*-pyrazol-5-ol **(3a)**

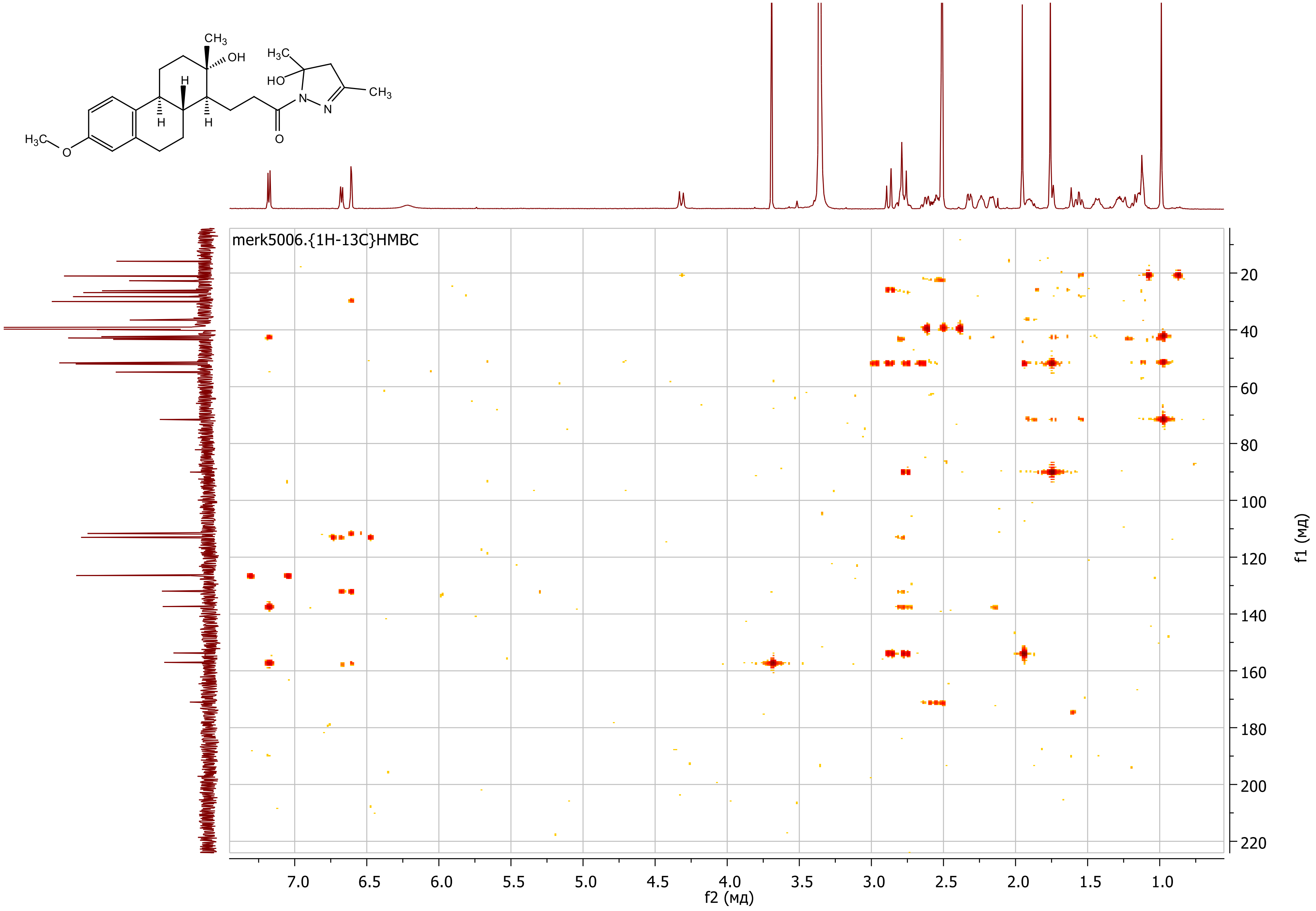

^{1}H – ^{1}H NOESY (DMSO-d$_6$, 600 MHz) spectrum of 1-{3-[(1*S*,2*S*,4a*S*,10a*R*)-2-hydroxy-7-methoxy-2-methyl-1,2,3,4,4a,9,10,10a-octahydrophenanthren-1-yl]propanoyl}-3,5-dimethyl-4,5-dihydro-1*H*-pyrazol-5-ol **(3a)**

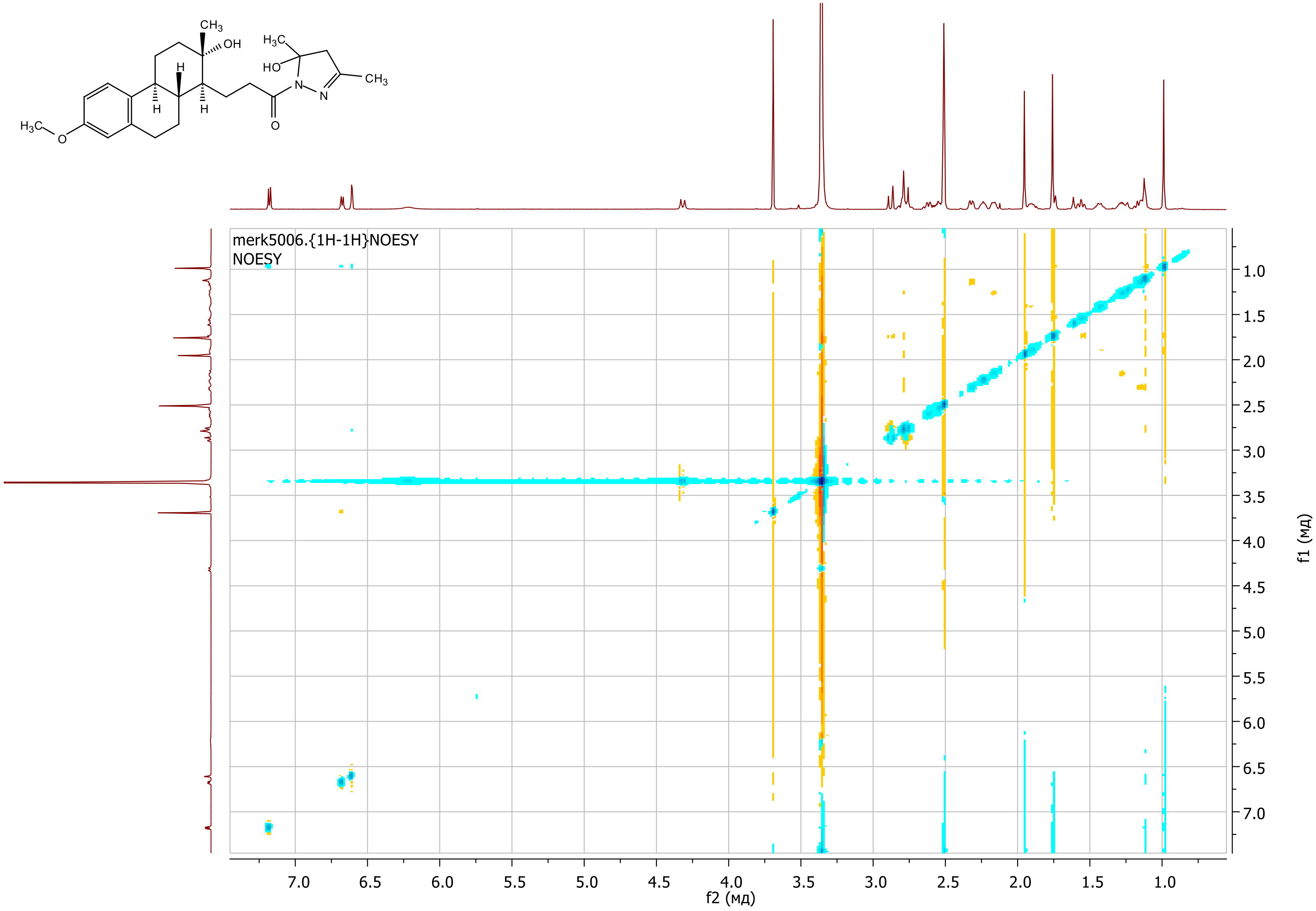

^{1}H NMR (DMSO-d$_6$, 300 MHz) spectrum of 1-{3-[(1*S*,2*S*,4a*S*,10a*R*)-2-hydroxy-7-methoxy-2-methyl-1,2,3,4,4a,9,10,10a-octahydrophenanthren-1-yl]propanoyl}-3,4,5-trimethyl-4,5-dihydro-1*H*-pyrazol-5-ol **(3b)**

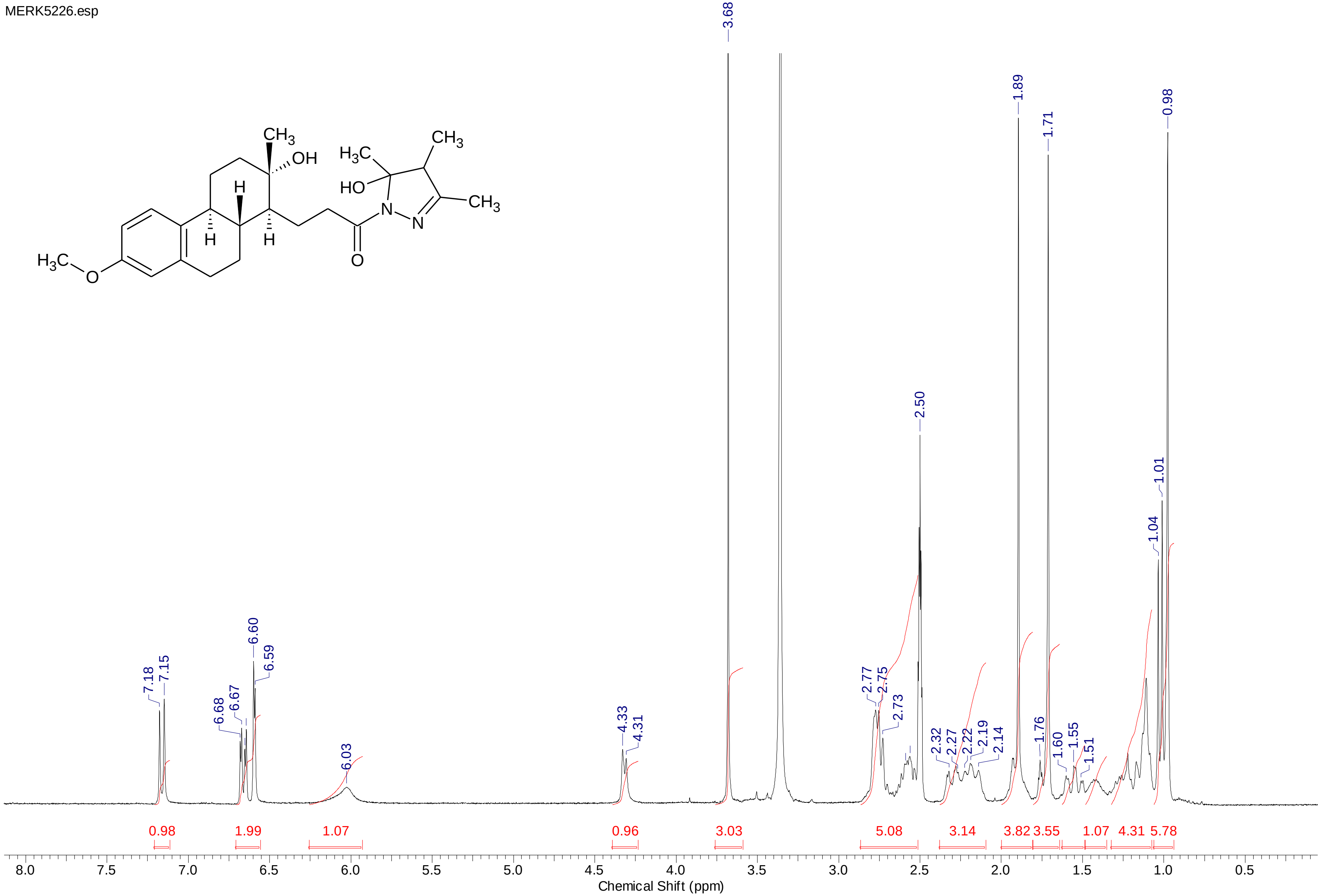

^{13}C NMR (DMSO-d$_6$, 75 MHz) spectrum of 1-{3-[(1*S*,2*S*,4a*S*,10a*R*)-2-hydroxy-7-methoxy-2-methyl-1,2,3,4,4a,9,10,10a-octahydrophenanthren-1-yl]propanoyl}-3,4,5-trimethyl-4,5-dihydro-1*H*-pyrazol-5-ol **(3b)**

merk5226.esp

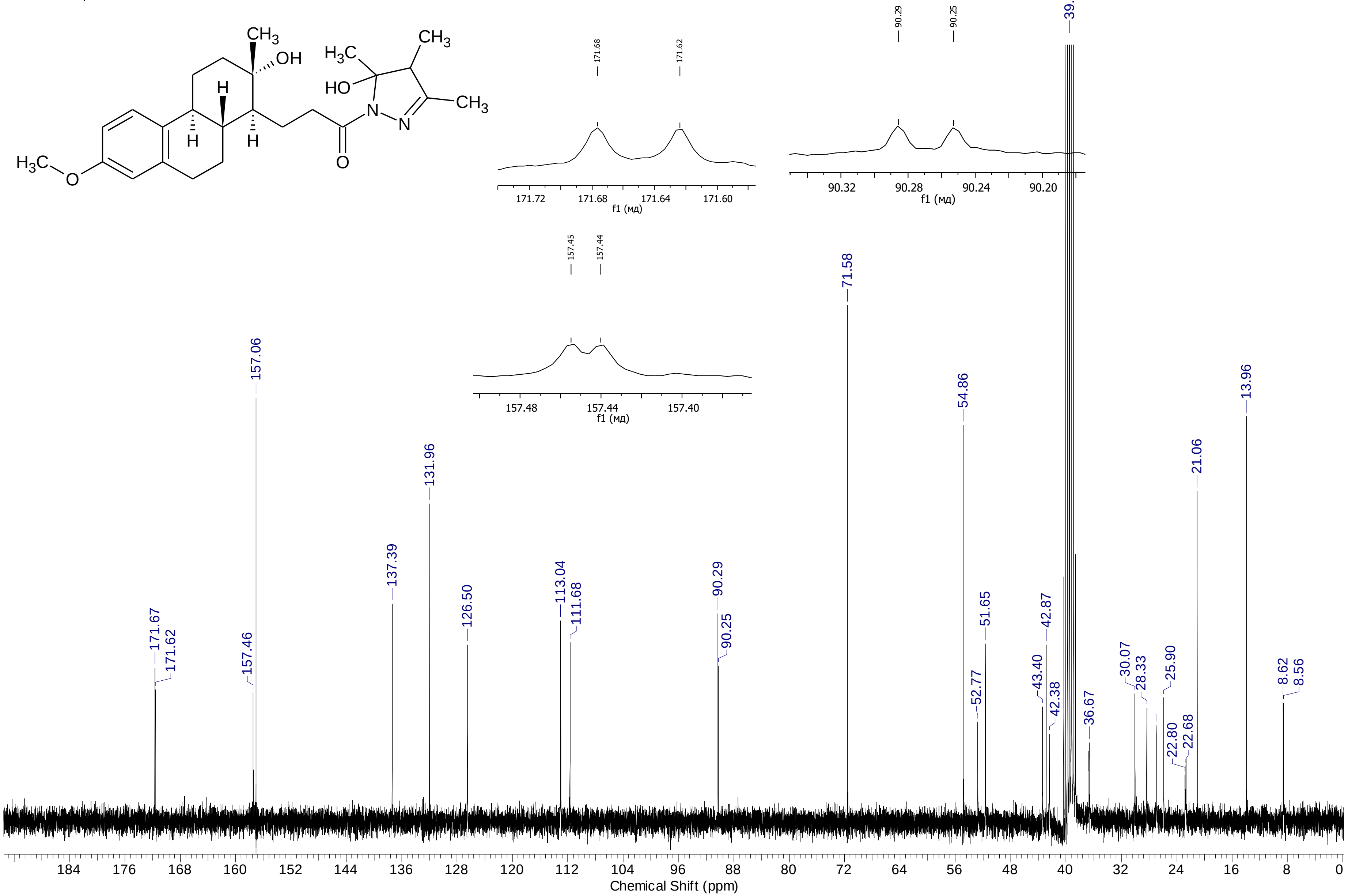

^{13}C NMR (DMSO-d$_6$, 75 MHz) spectrum of 1-{3-[(1*S*,2*S*,4a*S*,10a*R*)-2-hydroxy-7-methoxy-2-methyl-1,2,3,4,4a,9,10,10a-octahydrophenanthren-1-yl]propanoyl}-3,4,5-trimethyl-4,5-dihydro-1*H*-pyrazol-5-ol **(3b)**

merk5226.esp

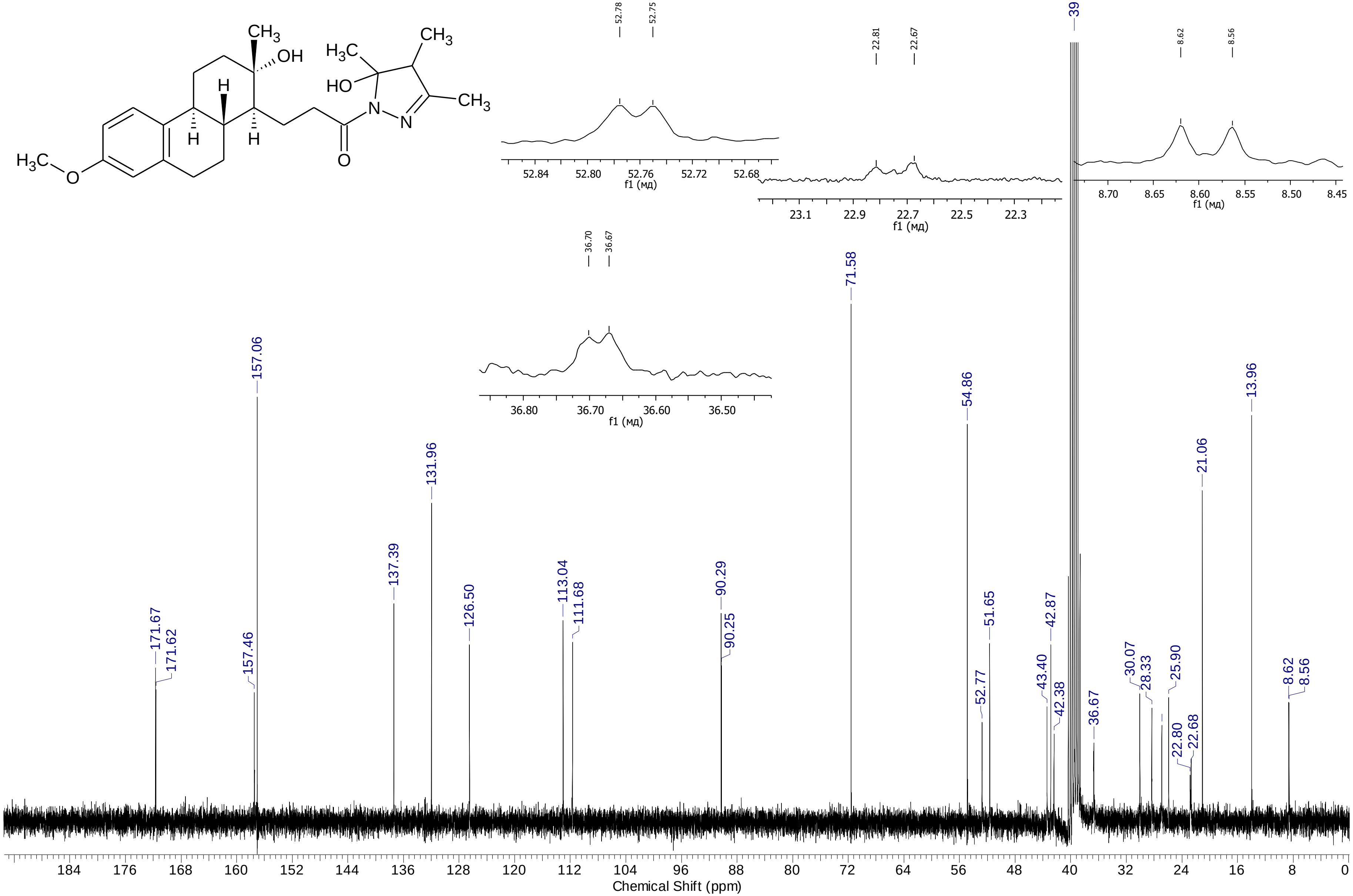

^{1}H NMR (DMSO-d$_6$, 600 MHz) spectrum of 1-{3-[(1*S*,2*S*,4a*S*,10a*R*)-2-hydroxy-7-methoxy-2-methyl-1,2,3,4,4a,9,10,10a-octahydrophenanthren-1-yl]propanoyl}-3,4,4,5-tetramethyl-4,5-dihydro-1*H*-pyrazol-5-ol **(3c)**

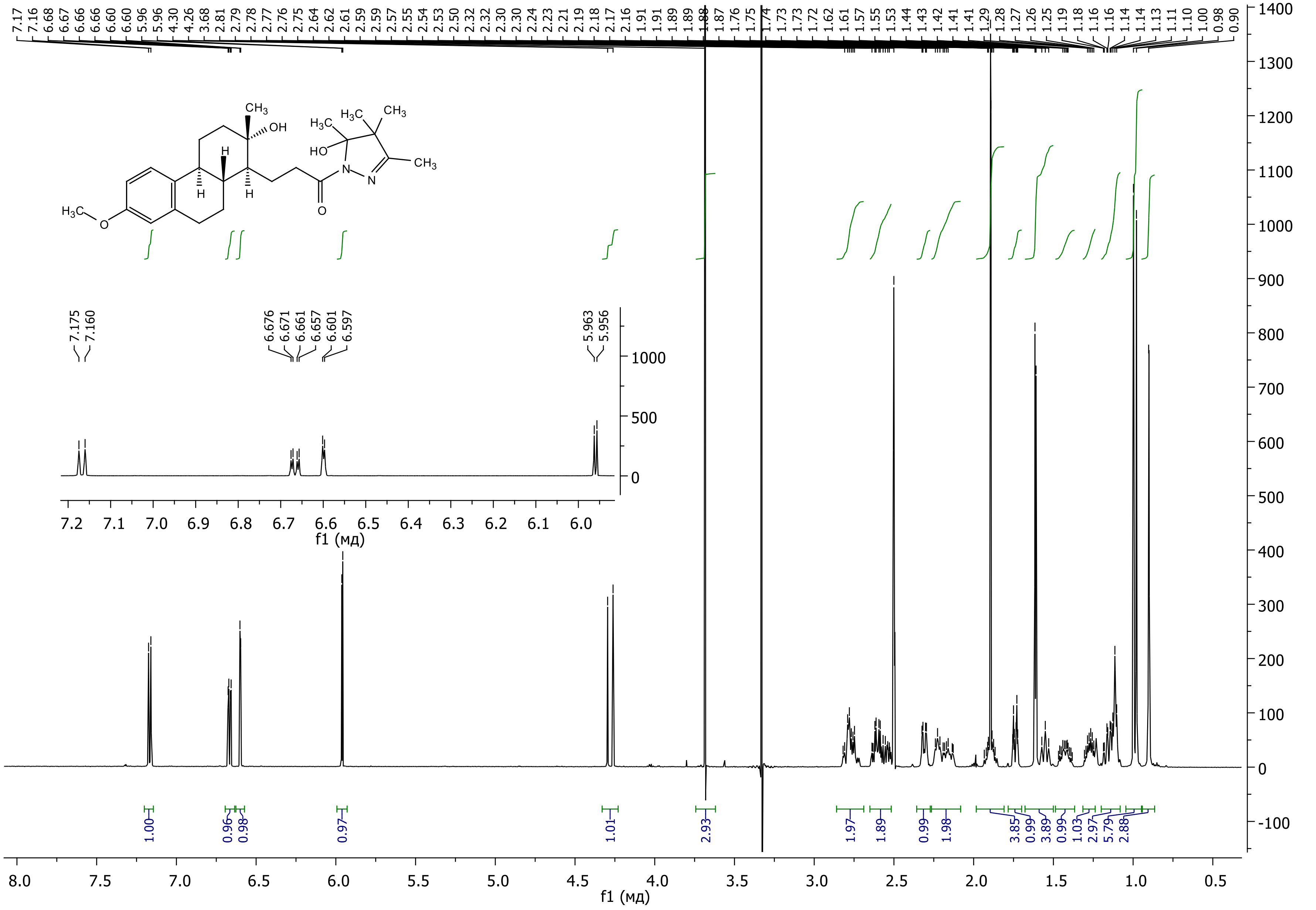

^{1}H NMR (DMSO-d$_6$, 600 MHz) spectrum of 1-{3-[(1*S*,2*S*,4a*S*,10a*R*)-2-hydroxy-7-methoxy-2-methyl-1,2,3,4,4a,9,10,10a-octahydrophenanthren-1-yl]propanoyl}-3,4,4,5-tetramethyl-4,5-dihydro-1*H*-pyrazol-5-ol **(3c)**

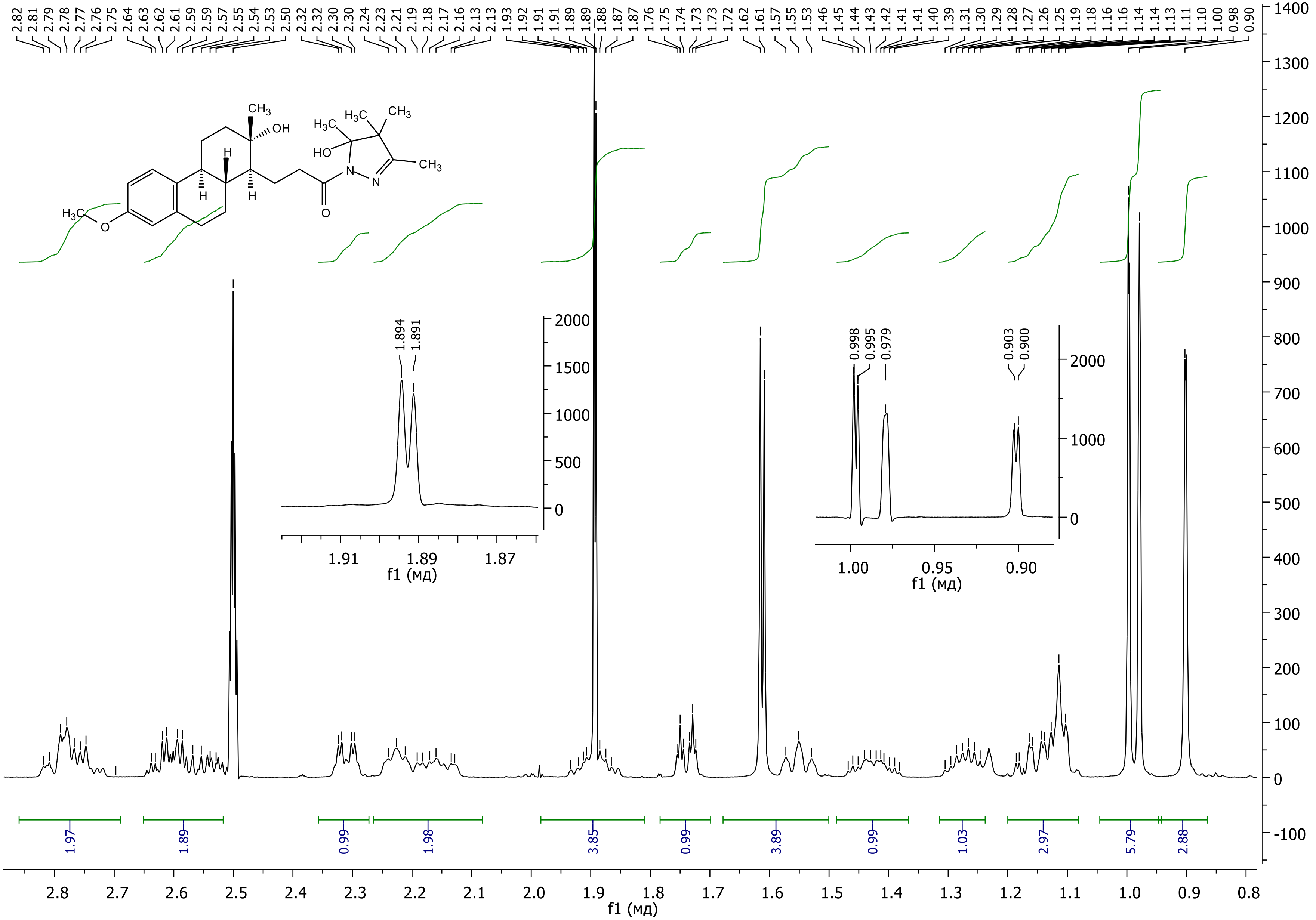

^{13}C NMR (DMSO-d$_6$, 150 MHz) spectrum of 1-{3-[(1*S*,2*S*,4a*S*,10a*R*)-2-hydroxy-7-methoxy-2-methyl-1,2,3,4,4a,9,10,10a-octahydrophenanthren-1-yl]propanoyl}-3,4,4,5-tetramethyl-4,5-dihydro-1*H*-pyrazol-5-ol **(3c)**

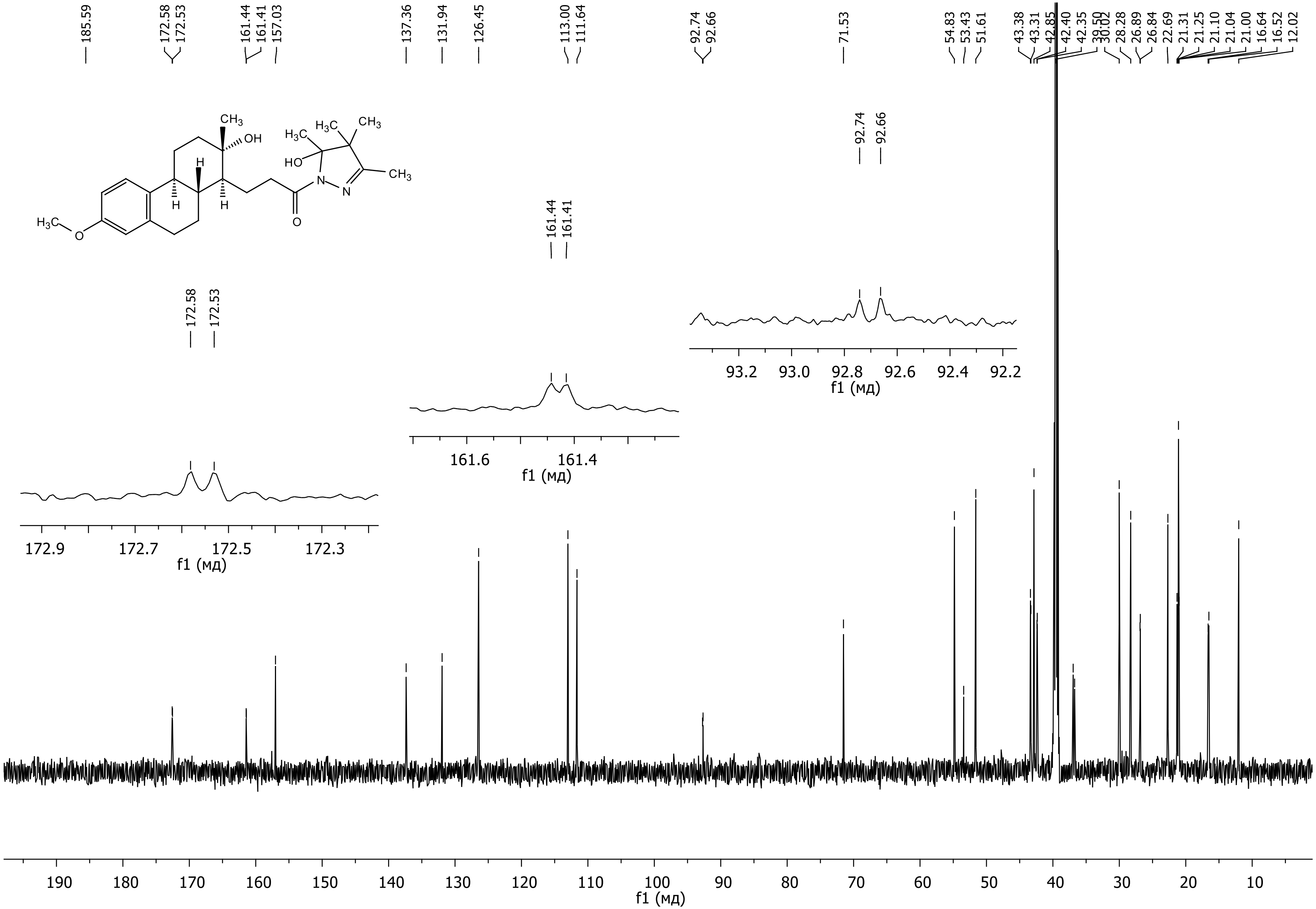

^{13}C NMR (DMSO-d_6, 150 MHz) spectrum of 1-{3-[(1*S*,2*S*,4a*S*,10a*R*)-2-hydroxy-7-methoxy-2-methyl-1,2,3,4,4a,9,10,10a-octahydrophenanthren-1-yl]propanoyl}-3,4,4,5-tetramethyl-4,5-dihydro-1*H*-pyrazol-5-ol **(3c)**

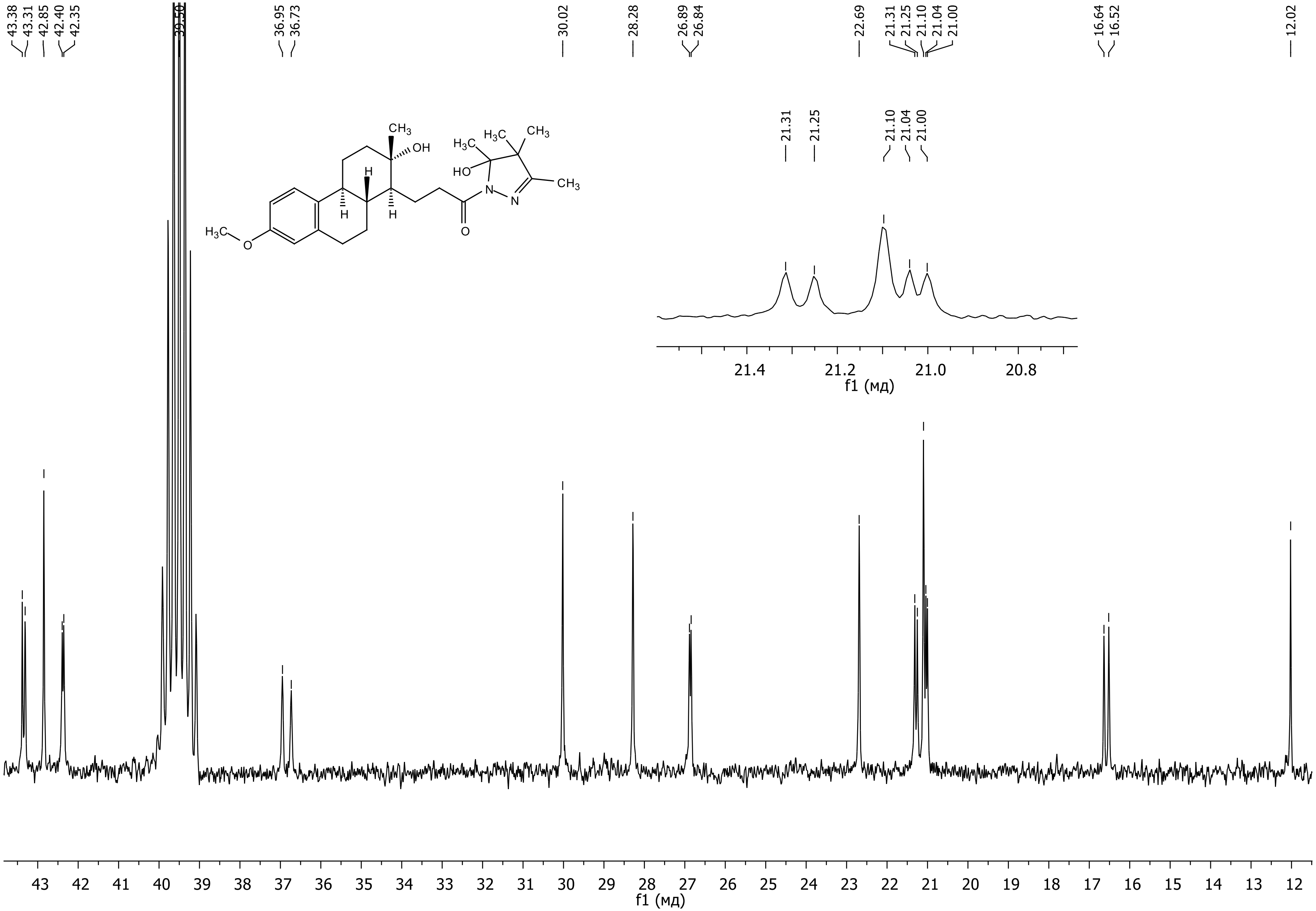

^{1}H – ^{1}H COSY (DMSO-d$_6$, 600 MHz) spectrum of 1-{3-[(1*S*,2*S*,4a*S*,10a*R*)-2-hydroxy-7-methoxy-2-methyl-1,2,3,4,4a,9,10,10a-octahydrophenanthren-1-yl]propanoyl}-3,4,4,5-tetramethyl-4,5-dihydro-1*H*-pyrazol-5-ol **(3c)**

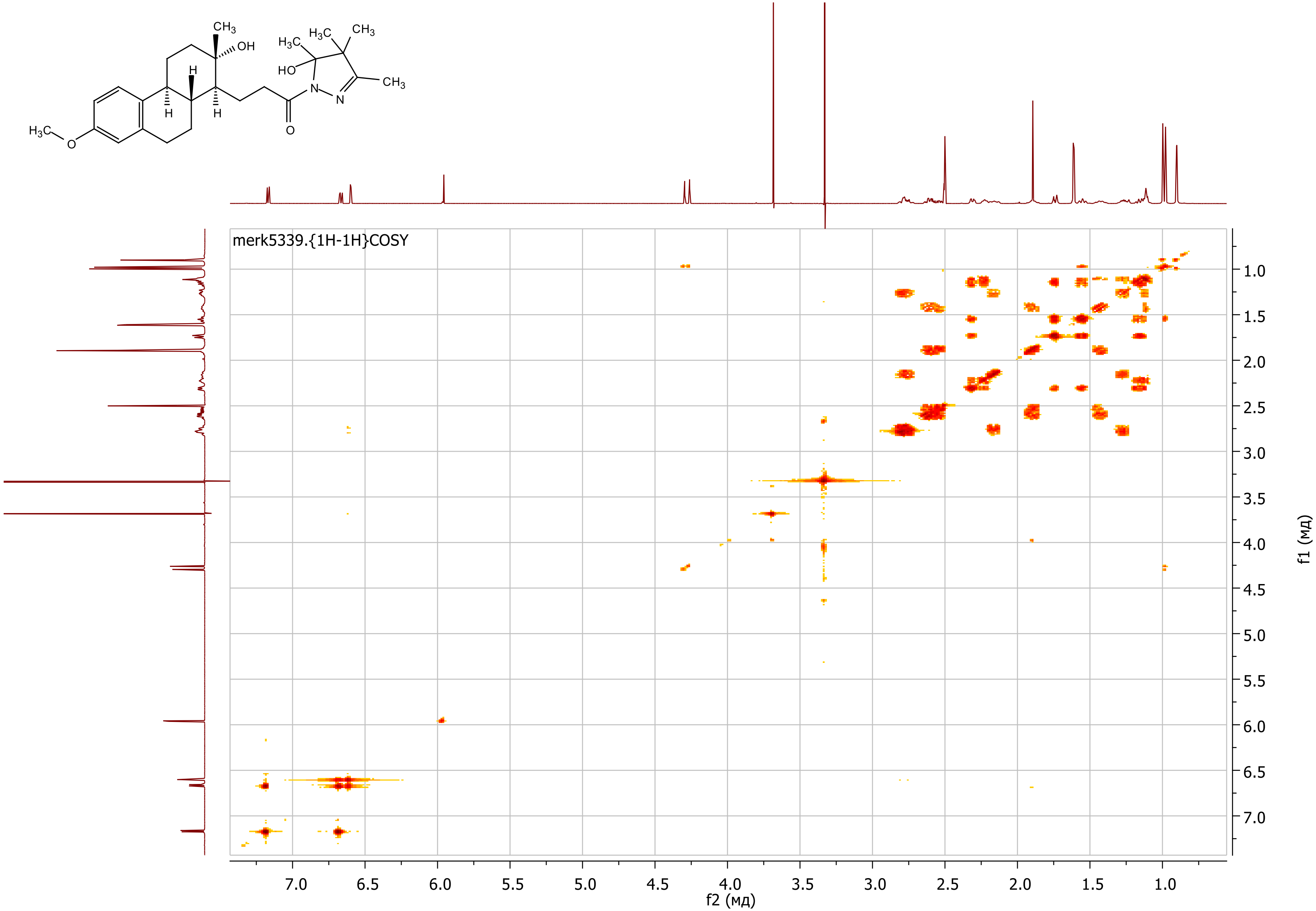

^{1}H – ^{13}C HSQC (DMSO-d$_6$, 600 MHz, 150 MHz) spectrum of 1-{3-[(1*S*,2*S*,4a*S*,10a*R*)-2-hydroxy-7-methoxy-2-methyl-1,2,3,4,4a,9,10,10a-octahydrophenanthren-1-yl]propanoyl}-3,4,4,5-tetramethyl-4,5-dihydro-1*H*-pyrazol-5-ol **(3c)**

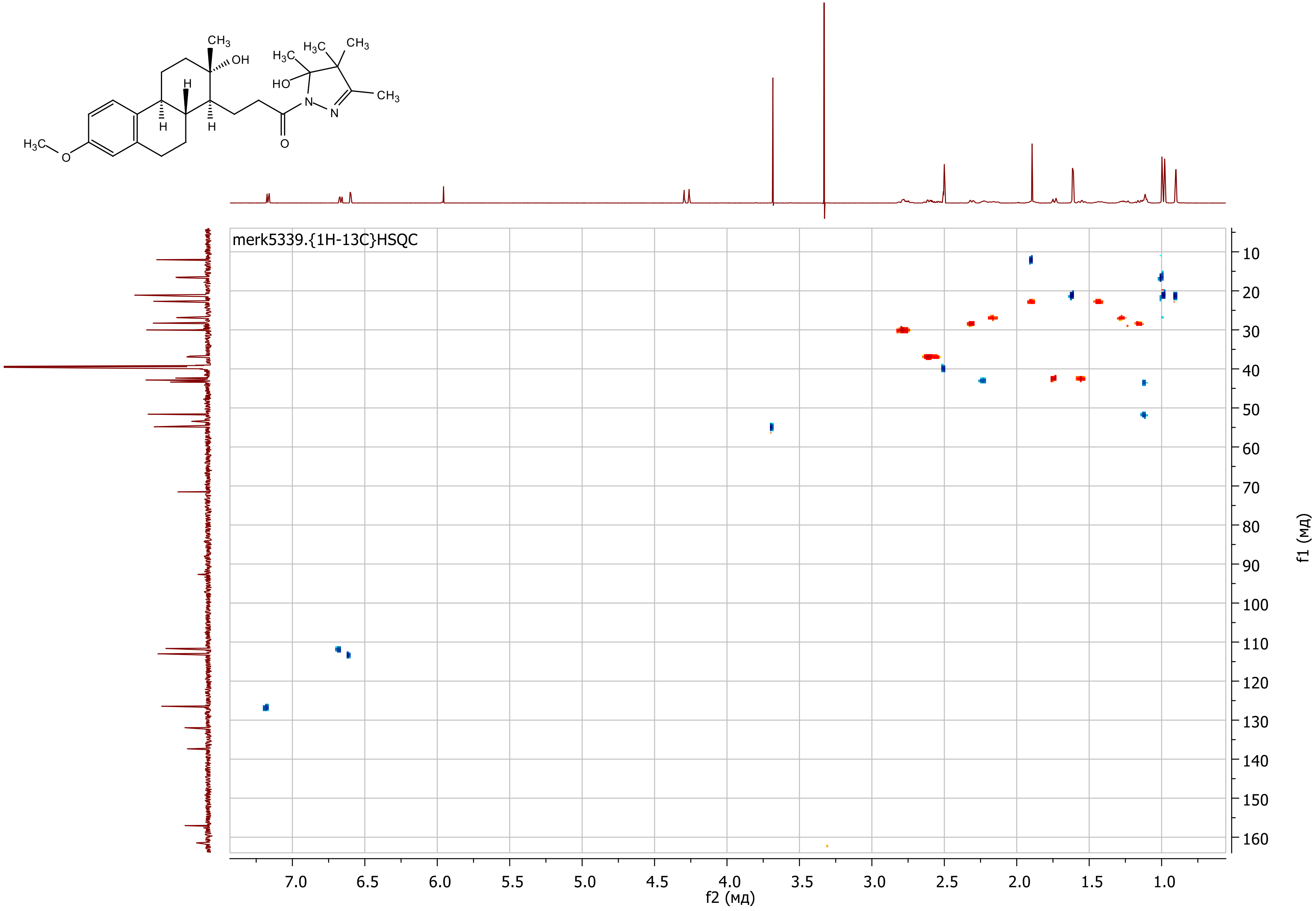

^{1}H – ^{13}C HMBC (DMSO-d_6, 600 MHz, 150 MHz) spectrum of 1-{3-[(1*S*,2*S*,4a*S*,10a*R*)-2-hydroxy-7-methoxy-2-methyl-1,2,3,4,4a,9,10,10a-octahydrophenanthren-1-yl]propanoyl}-3,4,4,5-tetramethyl-4,5-dihydro-1*H*-pyrazol-5-ol **(3c)**

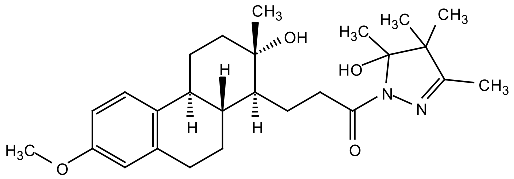

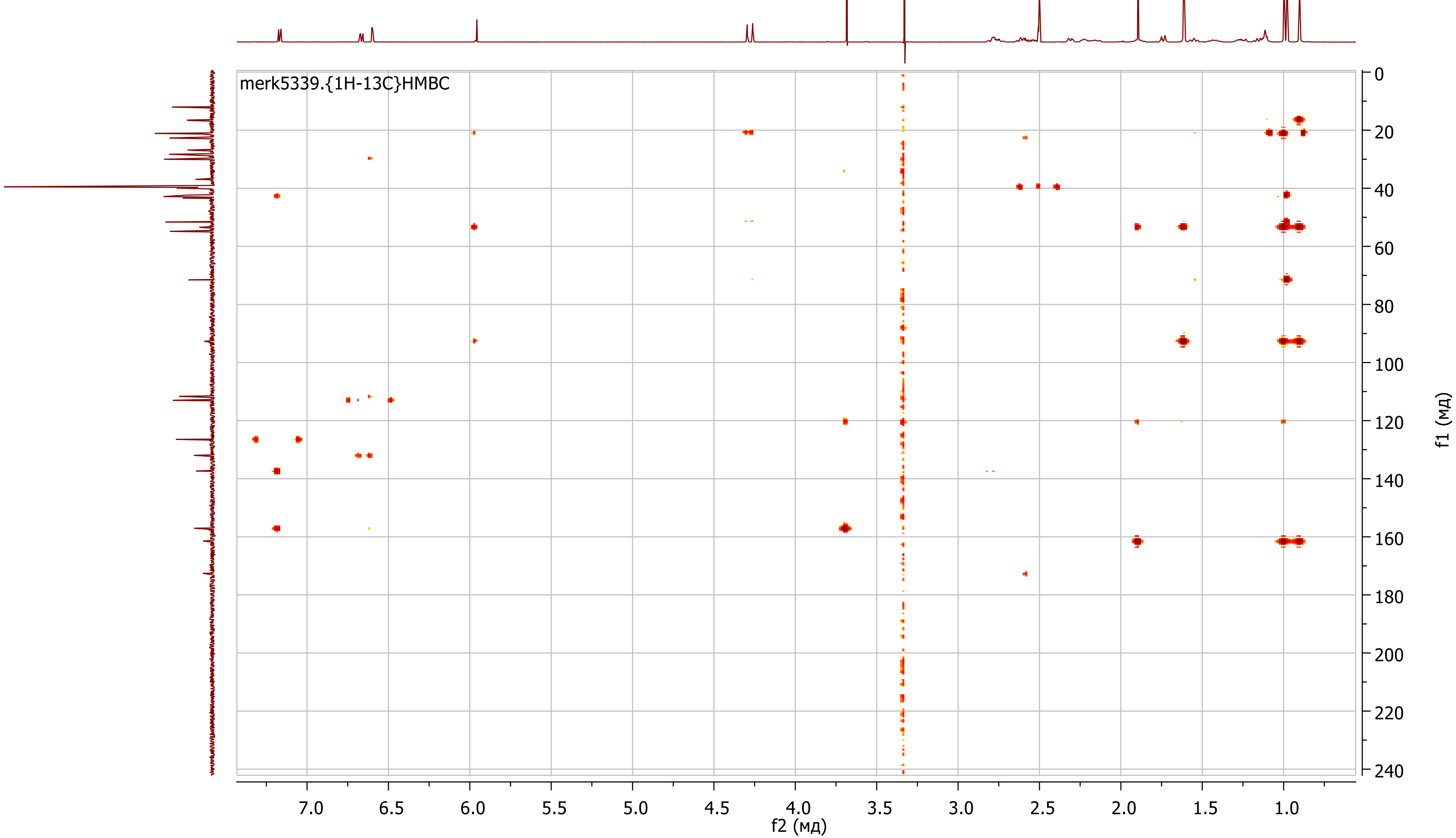

^{1}H NMR (DMSO-d$_6$, 300 MHz) spectrum of 4-Ethyl-1-{3-[(1*S*,2*S*,4a*S*,10a*R*)-2-hydroxy-7-methoxy-2-methyl-1,2,3,4,4a,9,10,10a-octahydrophenanthren-1-yl]propanoyl}-3,5-dimethyl-4,5-dihydro-1*H*-pyrazol-5-ol **(3d)**

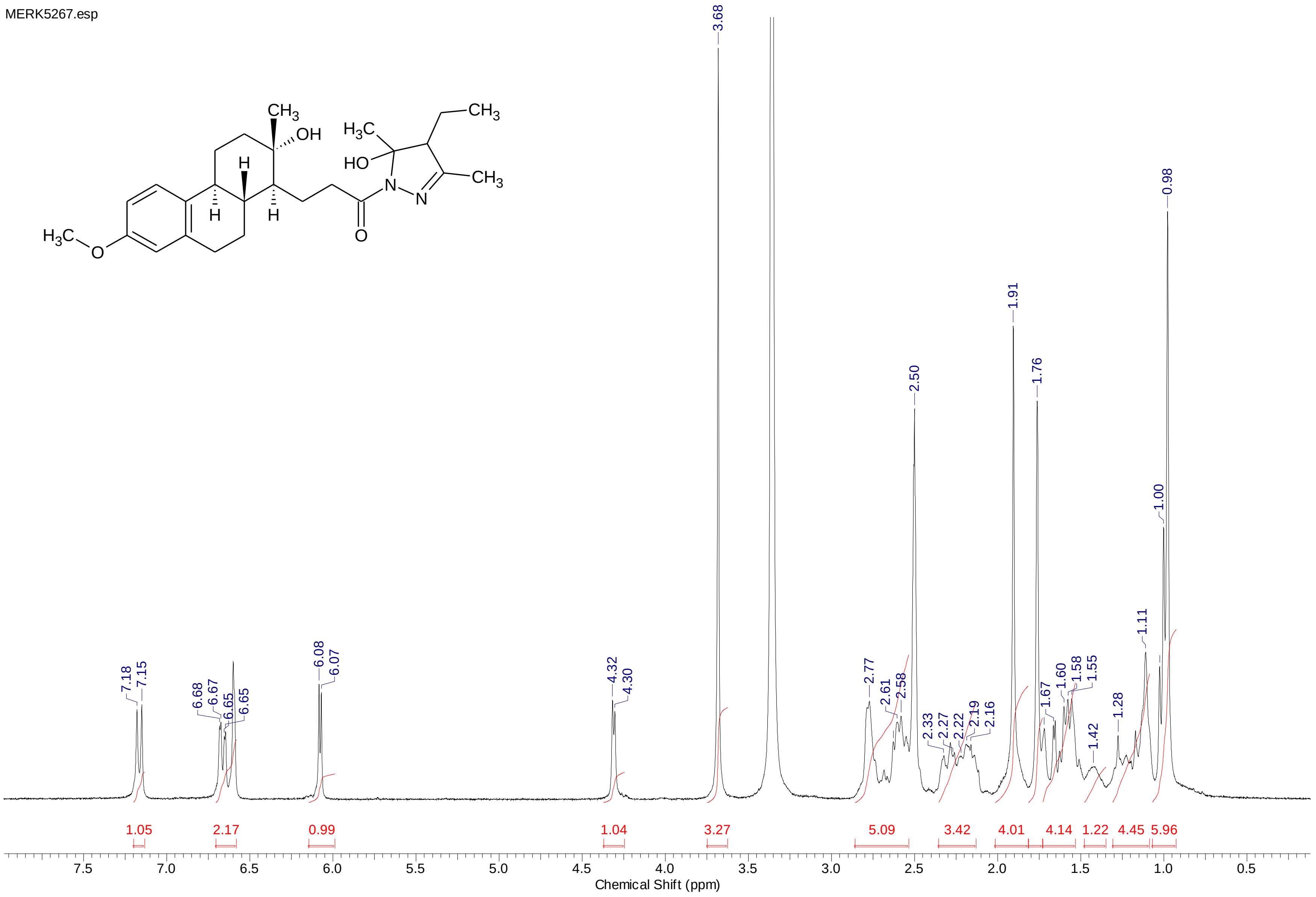

^{13}C NMR (DMSO-d_6, 75 MHz) spectrum of 4-Ethyl-1-{3-[(1*S*,2*S*,4a*S*,10a*R*)-2-hydroxy-7-methoxy-2-methyl-1,2,3,4,4a,9,10,10a-octahydrophenanthren-1-yl]propanoyl}-3,5-dimethyl-4,5-dihydro-1*H*-pyrazol-5-ol **(3d)**

merk5267.{13C}_002000fid

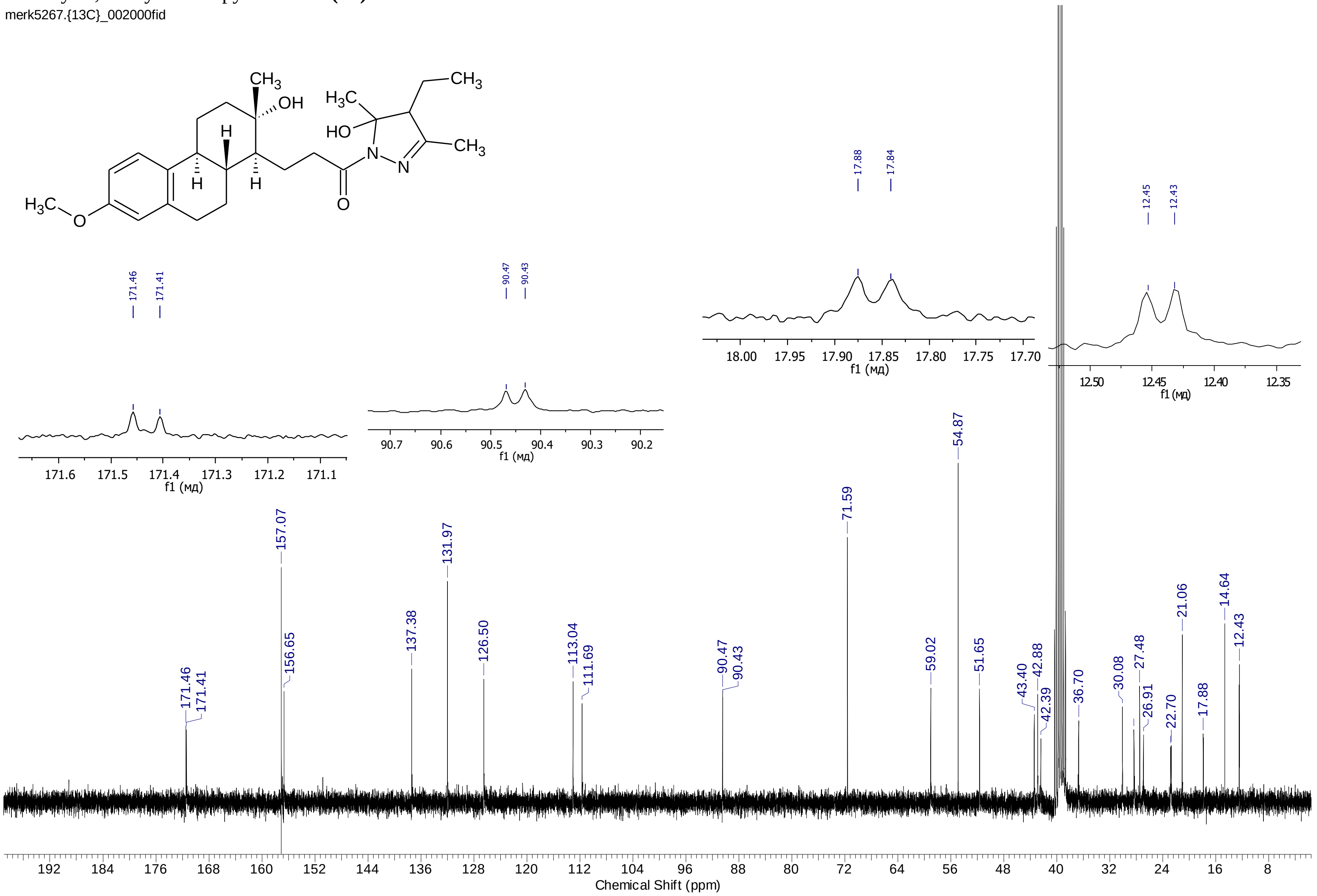

^{1}H NMR (DMSO-d6, 600 MHz) spectrum of 4-Butyl-1-{3-[(1*S*,2*S*,4a*S*,10a*R*)-2-hydroxy-7-methoxy-2-methyl-1,2,3,4,4a,9,10,10a-octahydrophenanthren-1-yl]propanoyl}-3,5-dimethyl-4,5-dihydro-1*H*-pyrazol-5-ol **(3e)**

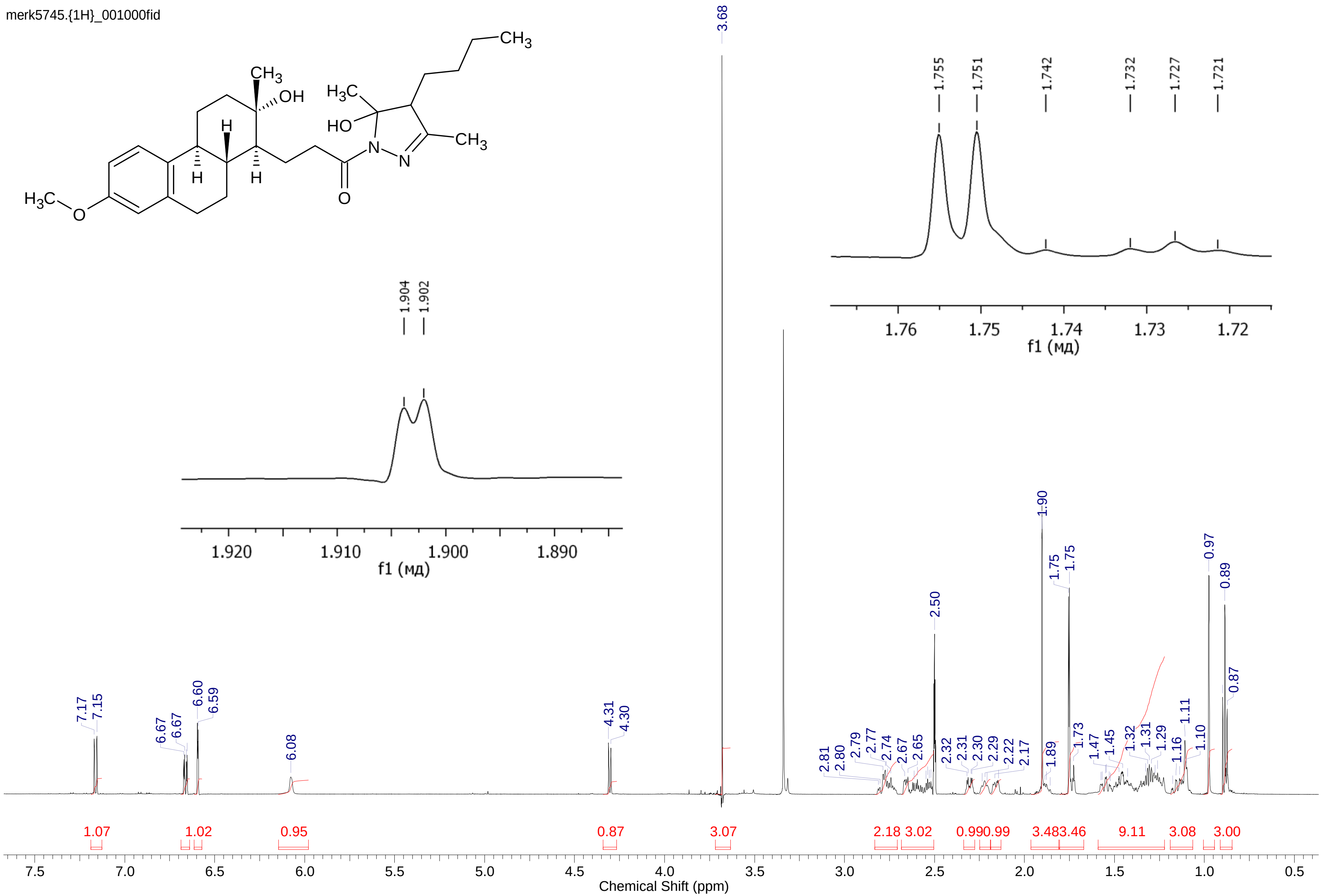

^{13}C NMR (DMSO-d_6, 150 MHz) spectrum of 4-Butyl-1-{3-[(1*S*,2*S*,4a*S*,10a*R*)-2-hydroxy-7-methoxy-2-methyl-1,2,3,4,4a,9,10,10a-octahydrophenanthren-1-yl]propanoyl}-3,5-dimethyl-4,5-dihydro-1*H*-pyrazol-5-ol **(3e)**

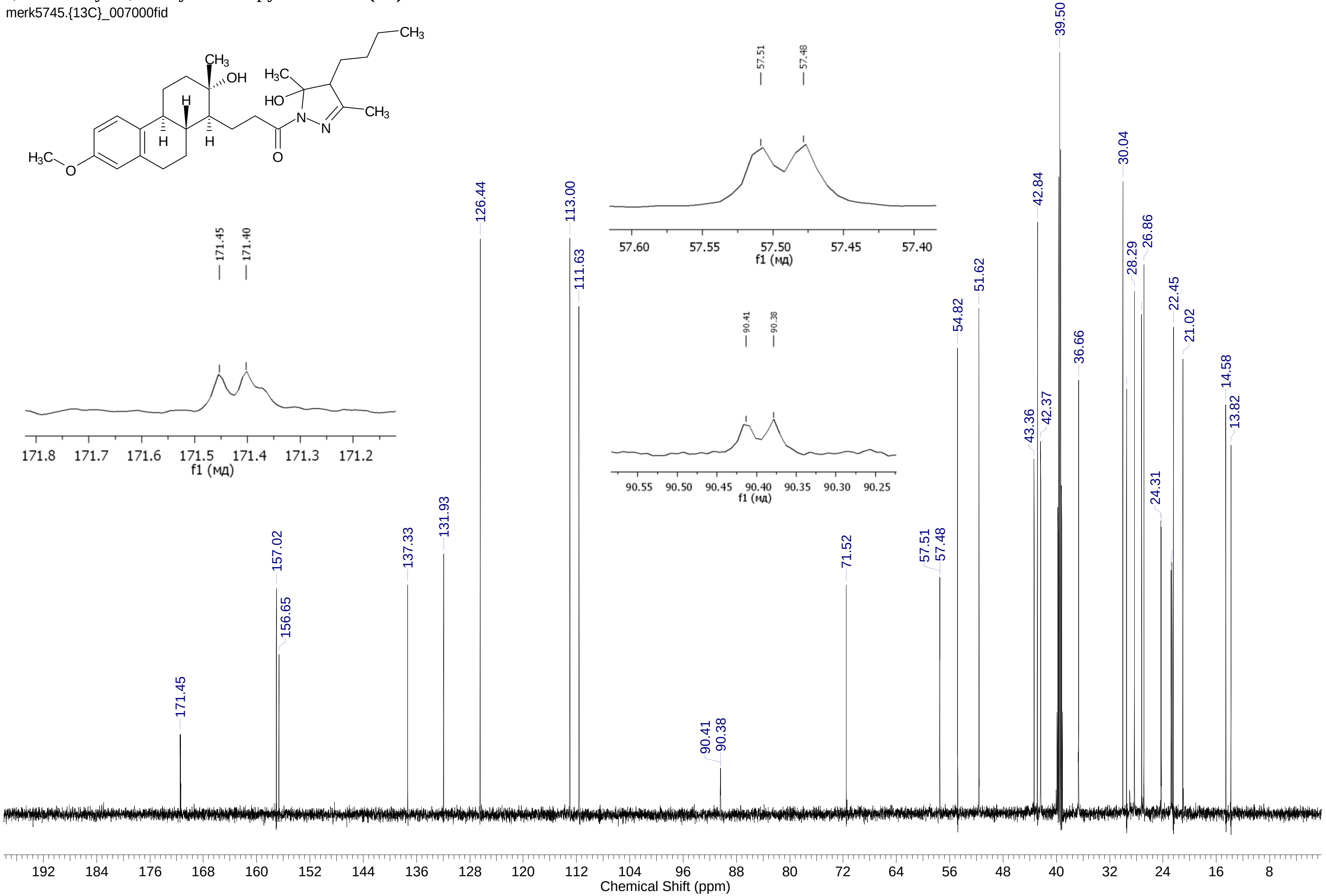

^{13}C NMR (DMSO-d6, 150 MHz) spectrum of 4-Butyl-1-{3-[(1*S*,2*S*,4a*S*,10a*R*)-2-hydroxy-7-methoxy-2-methyl-1,2,3,4,4a,9,10,10a-octahydrophenanthren-1-yl]propanoyl}-3,5-dimethyl-4,5-dihydro-1*H*-pyrazol-5-ol **(3e)**

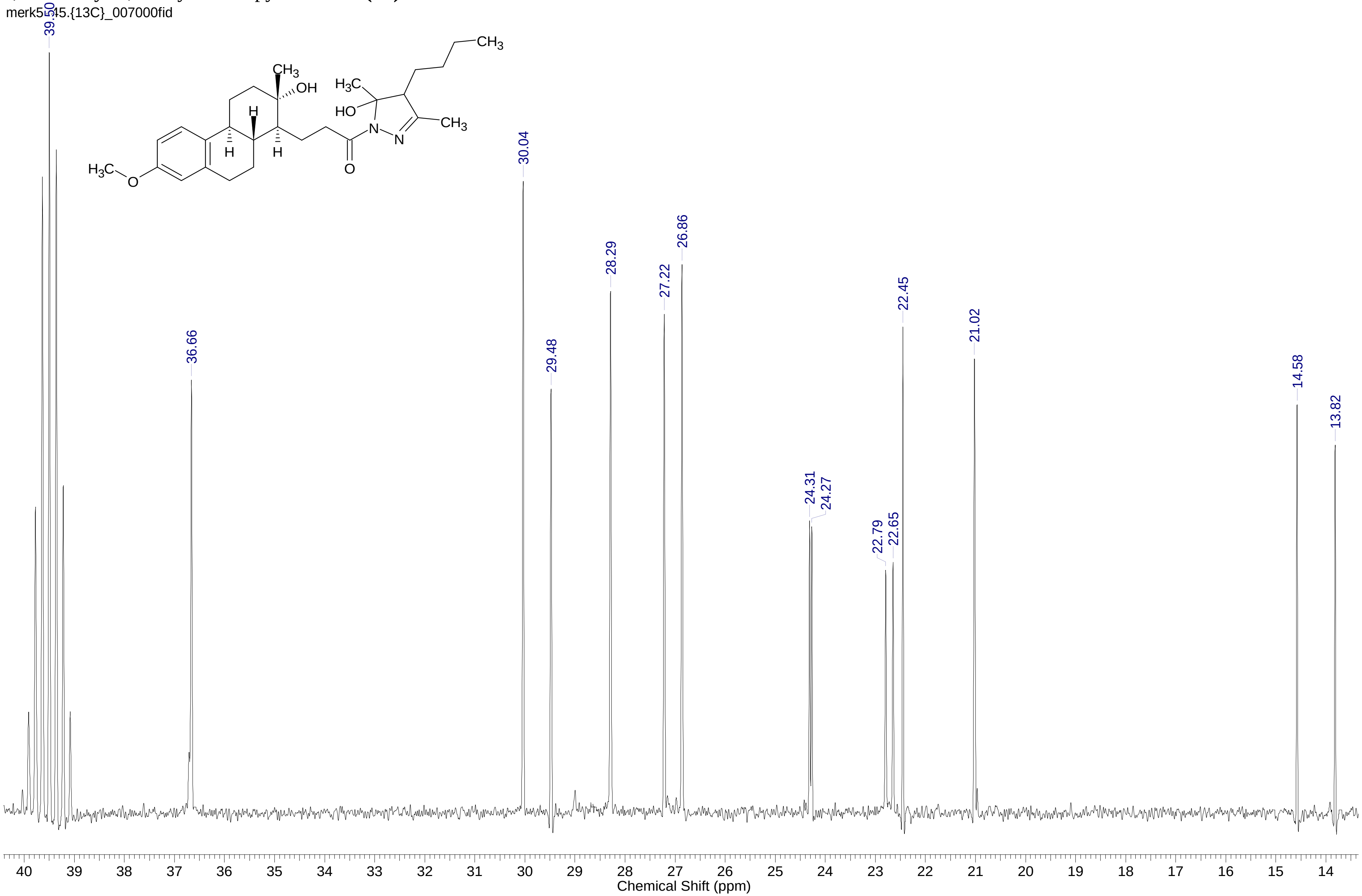

^{1}H NMR (DMSO-d$_6$, 300 MHz) spectrum of 1-{3-[(1*S*,2*S*,4a*S*,10a*R*)-2-Hydroxy-7-methoxy-2-methyl-1,2,3,4,4a,9,10,10a-octahydrophenanthren-1-yl]propanoyl}-3,5-dimethyl-4-(3-methylbutyl)-4,5-dihydro-1*H*-pyrazol-5-ol **(3f)**

MERK5208.esp

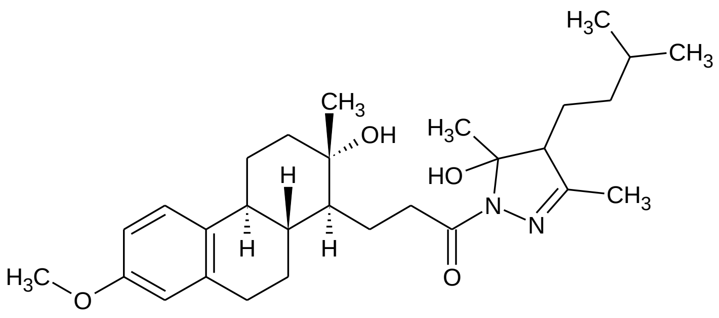

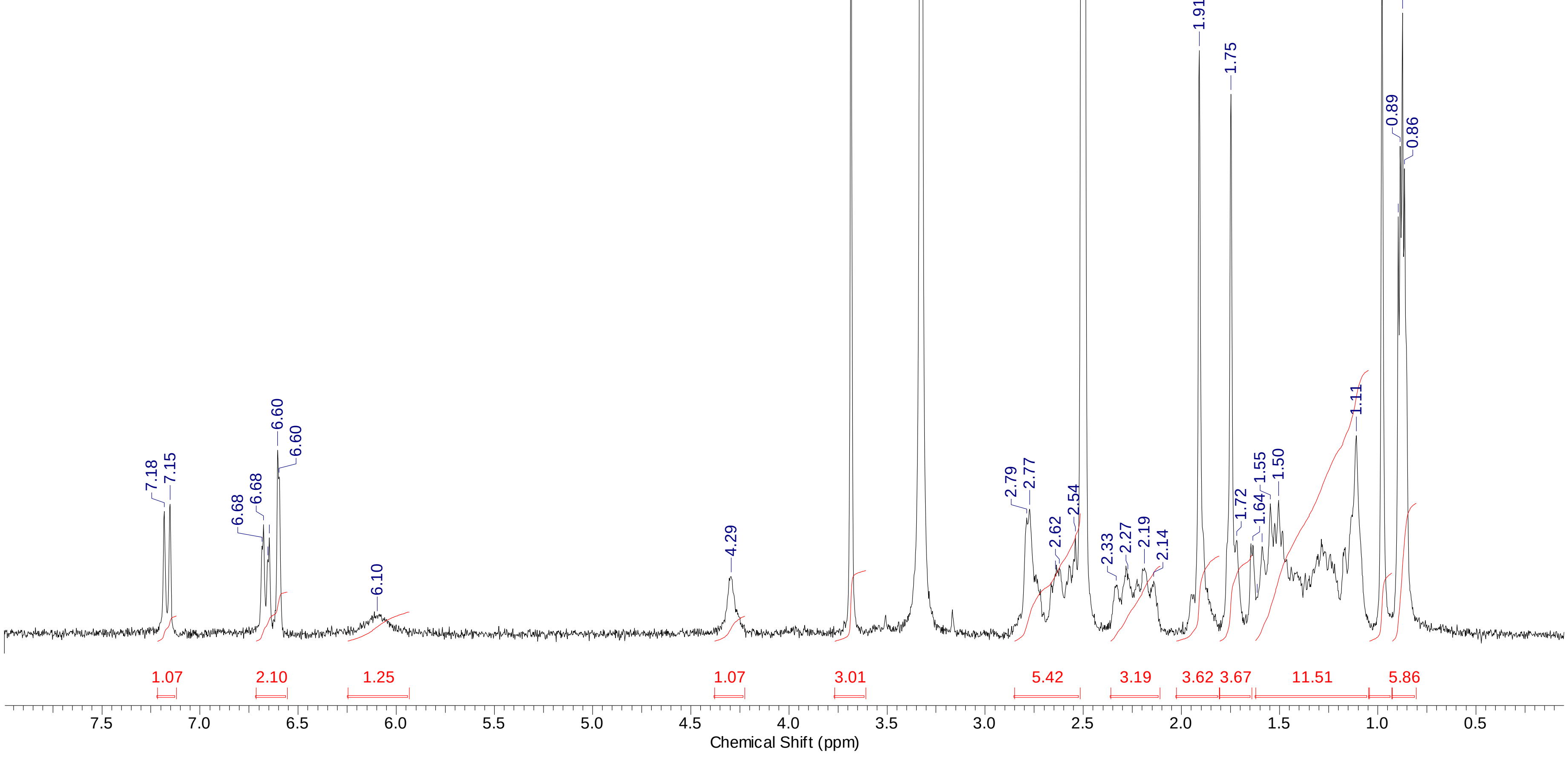

^{13}C NMR (DMSO-d_6, 75 MHz) spectrum of 1-{3-[(1*S*,2*S*,4a*S*,10a*R*)-2-Hydroxy-7-methoxy-2-methyl-1,2,3,4,4a,9,10,10a-octahydrophenanthren-1-yl]propanoyl}-3,5-dimethyl-4-(3-methylbutyl)-4,5-dihydro-1*H*-pyrazol-5-ol **(3f)**

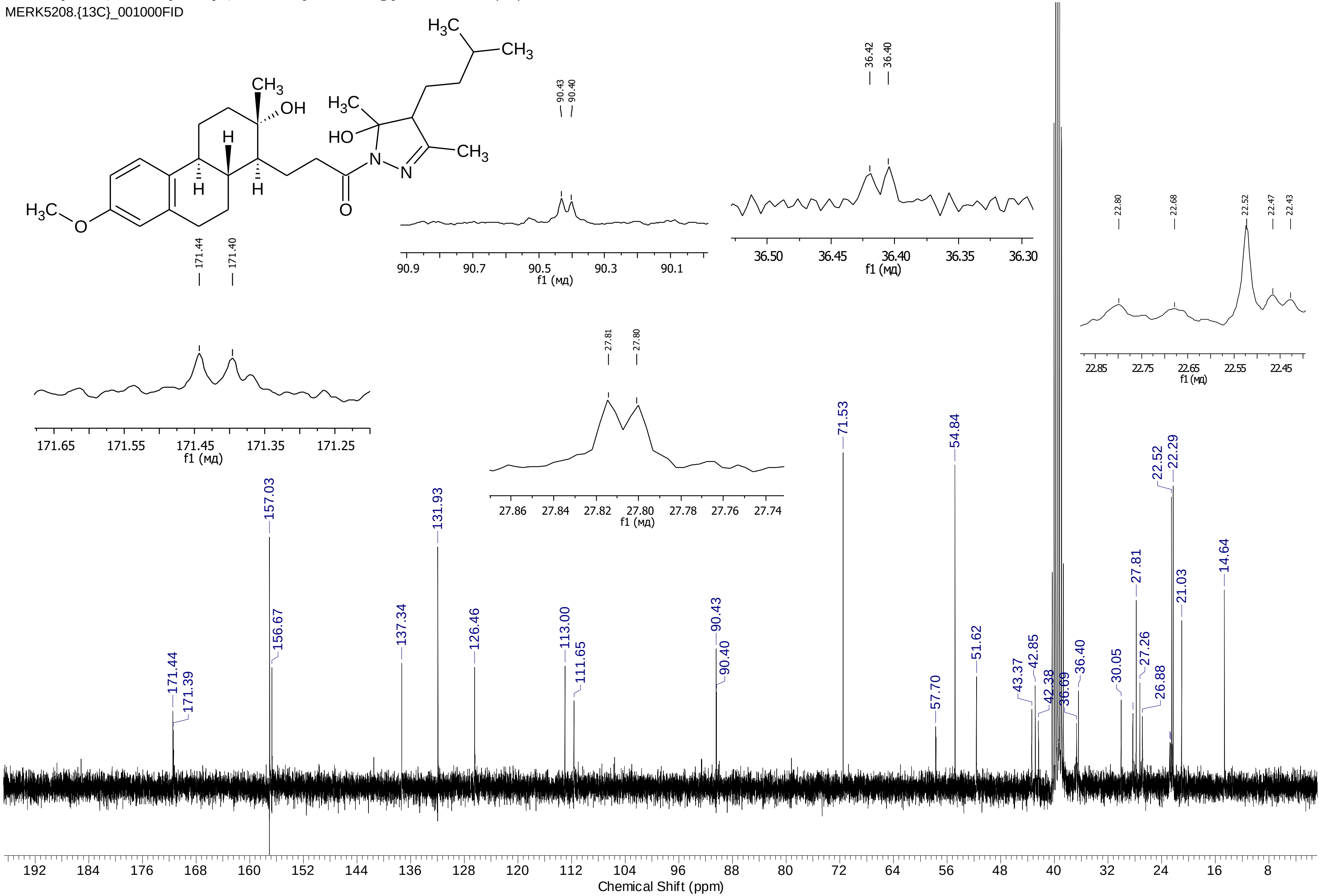

^{1}H NMR (DMSO-d_6, 500 MHz) spectrum of 4-Hexyl-1-{3-[(1*S*,2*S*,4a*S*,10a*R*)-2-hydroxy-7-methoxy-2-methyl-1,2,3,4,4a,9,10,10a-octahydrophenanthren-1-yl]propanoyl}-3,5-dimethyl-4,5-dihydro-1*H*-pyrazol-5-ol **(3g)**

MERK5694.esp

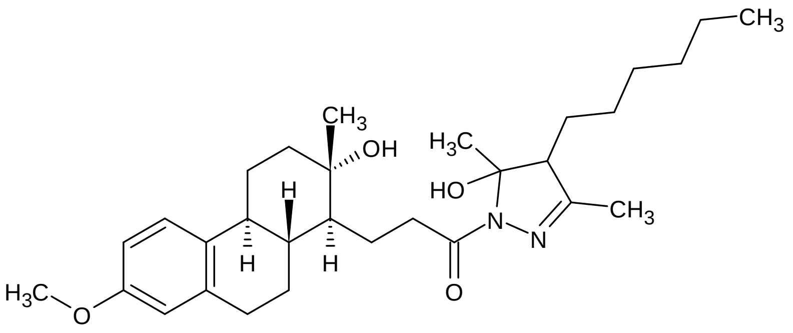

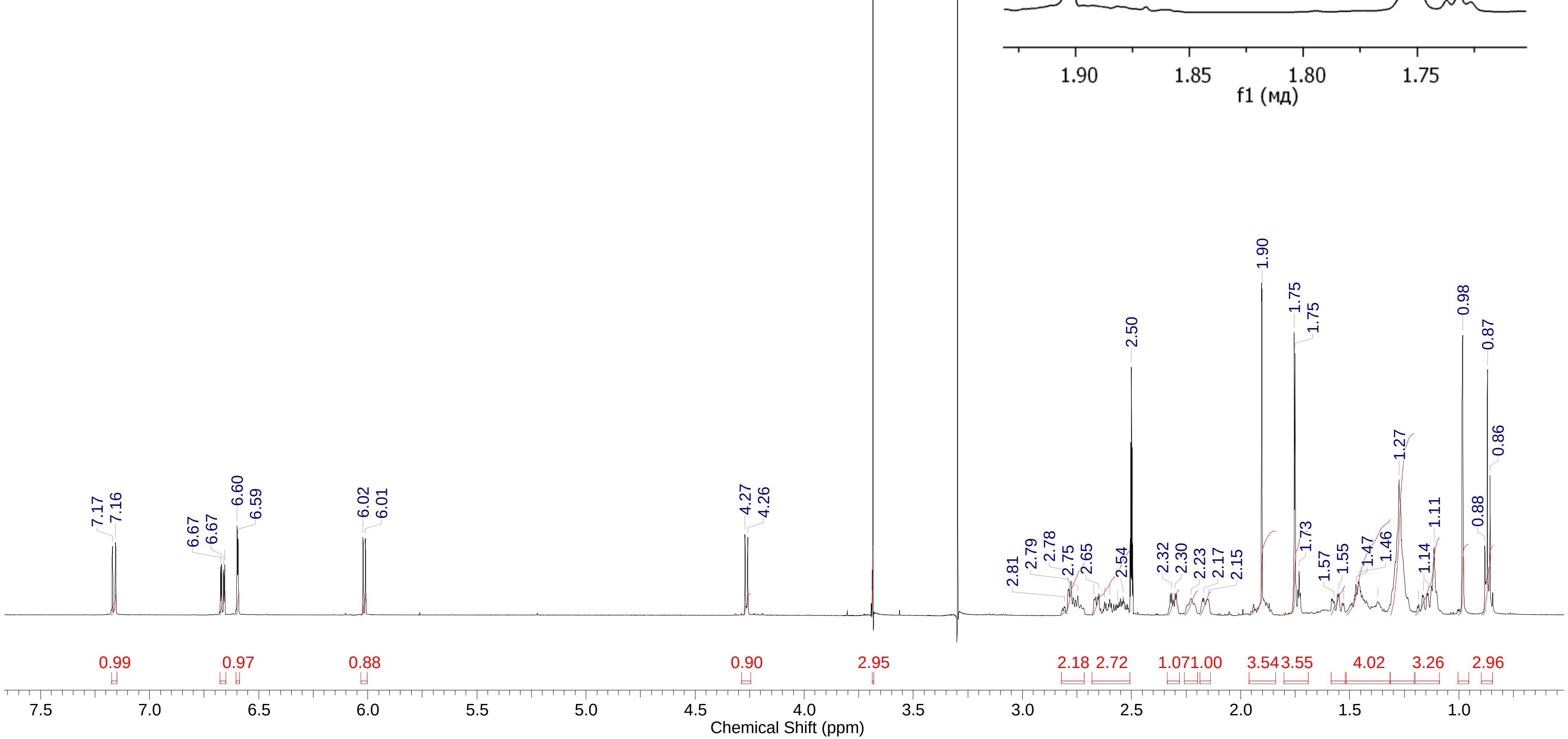

^{13}C NMR (DMSO-d_6, 125 MHz) spectrum of 4-Hexyl-1-{3-[(1*S*,2*S*,4a*S*,10a*R*)-2-hydroxy-7-methoxy-2-methyl-1,2,3,4,4a,9,10,10a-octahydrophenanthren-1-yl]propanoyl}-3,5-dimethyl-4,5-dihydro-1*H*-pyrazol-5-ol **(3g)**

MERK5694_NS41000.esp

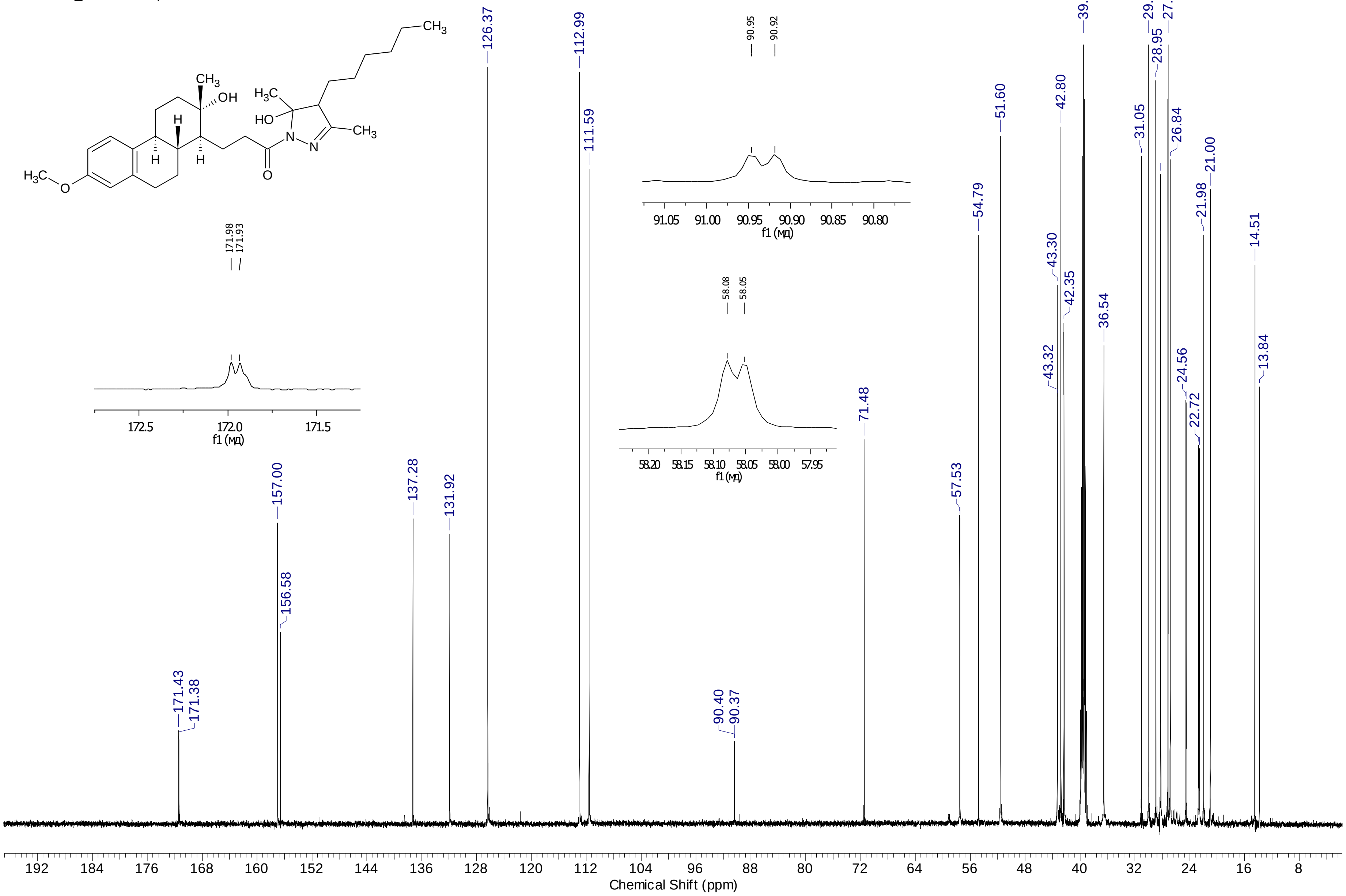

^{13}C NMR (DMSO-d_6, 125 MHz) spectrum of 4-Hexyl-1-{3-[(1*S*,2*S*,4a*S*,10a*R*)-2-hydroxy-7-methoxy-2-methyl-1,2,3,4,4a,9,10,10a-octahydrophenanthren-1-yl]propanoyl}-3,5-dimethyl-4,5-dihydro-1*H*-pyrazol-5-ol **(3g)**

MERK5694_NS41000.esp

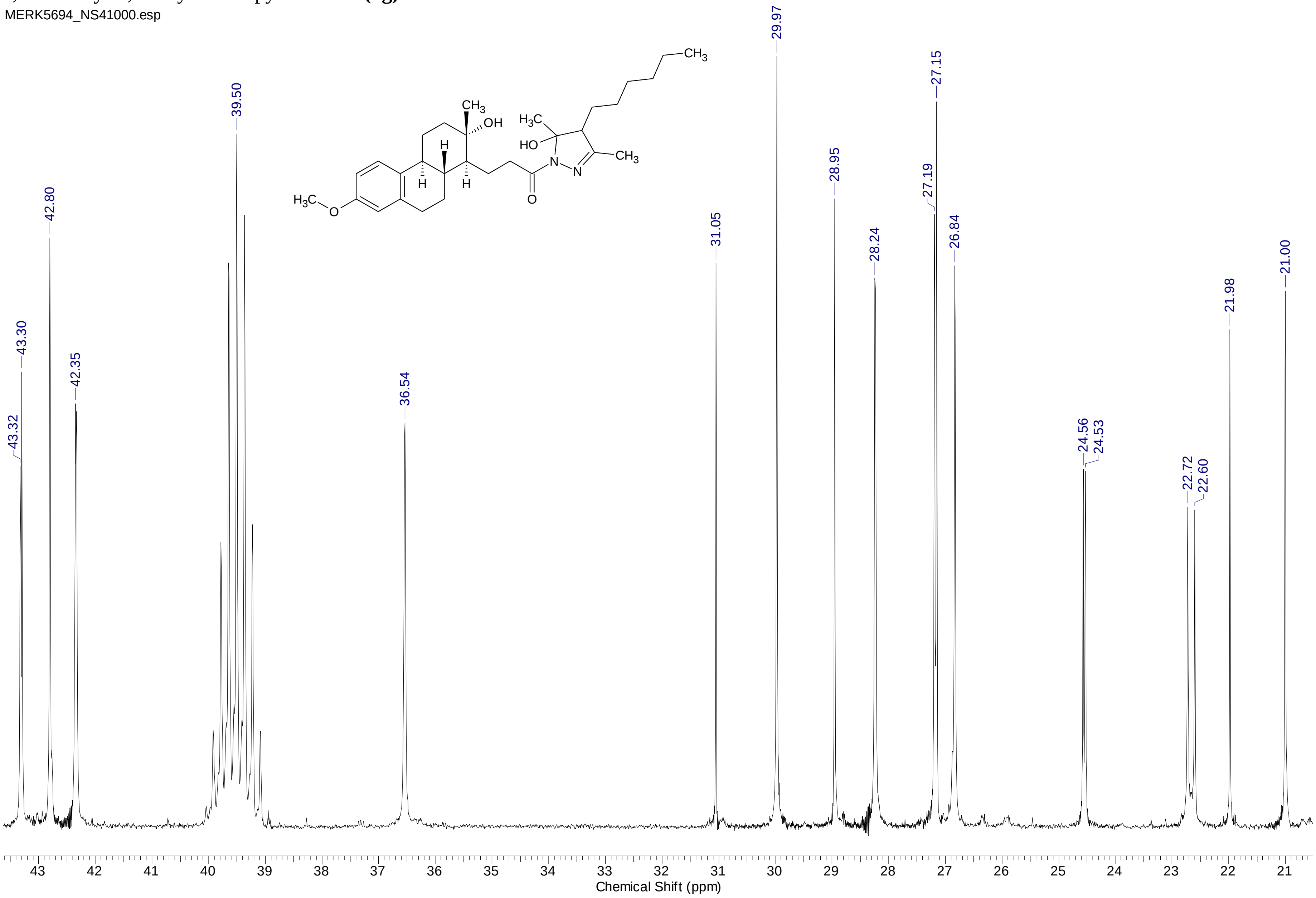

^{1}H NMR (DMSO-d6, 500 MHz) spectrum of 4-Allyl-1-{3-[(1*S*,2*S*,4a*S*,10a*R*)-2-hydroxy-7-methoxy-2-methyl-1,2,3,4,4a,9,10,10a-octahydrophenanthren-1-yl]propanoyl}-3,5-dimethyl-4,5-dihydro-1*H*-pyrazol-5-ol (**3h**)

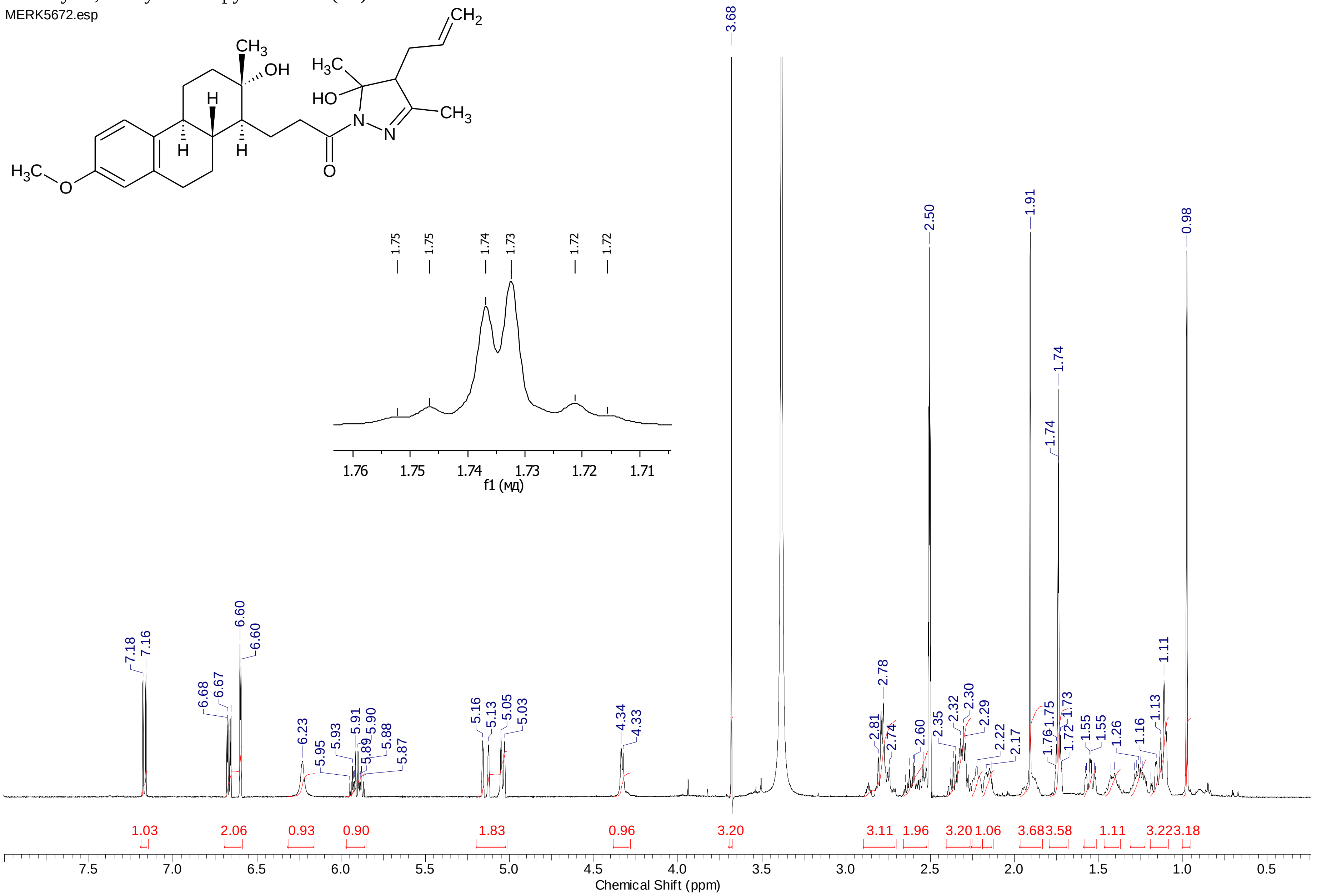

^{13}C NMR (DMSO-d$_6$, 125 MHz) spectrum of 4-Allyl-1-{3-[(1*S*,2*S*,4a*S*,10a*R*)-2-hydroxy-7-methoxy-2-methyl-1,2,3,4,4a,9,10,10a-octahydrophenanthren-1-yl]propanoyl}-3,5-dimethyl-4,5-dihydro-1*H*-pyrazol-5-ol (**3h**)

MERK5672.esp

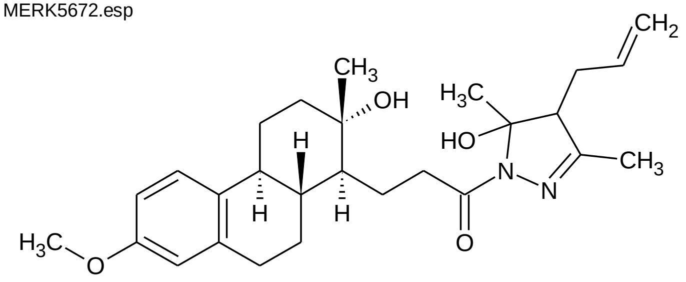

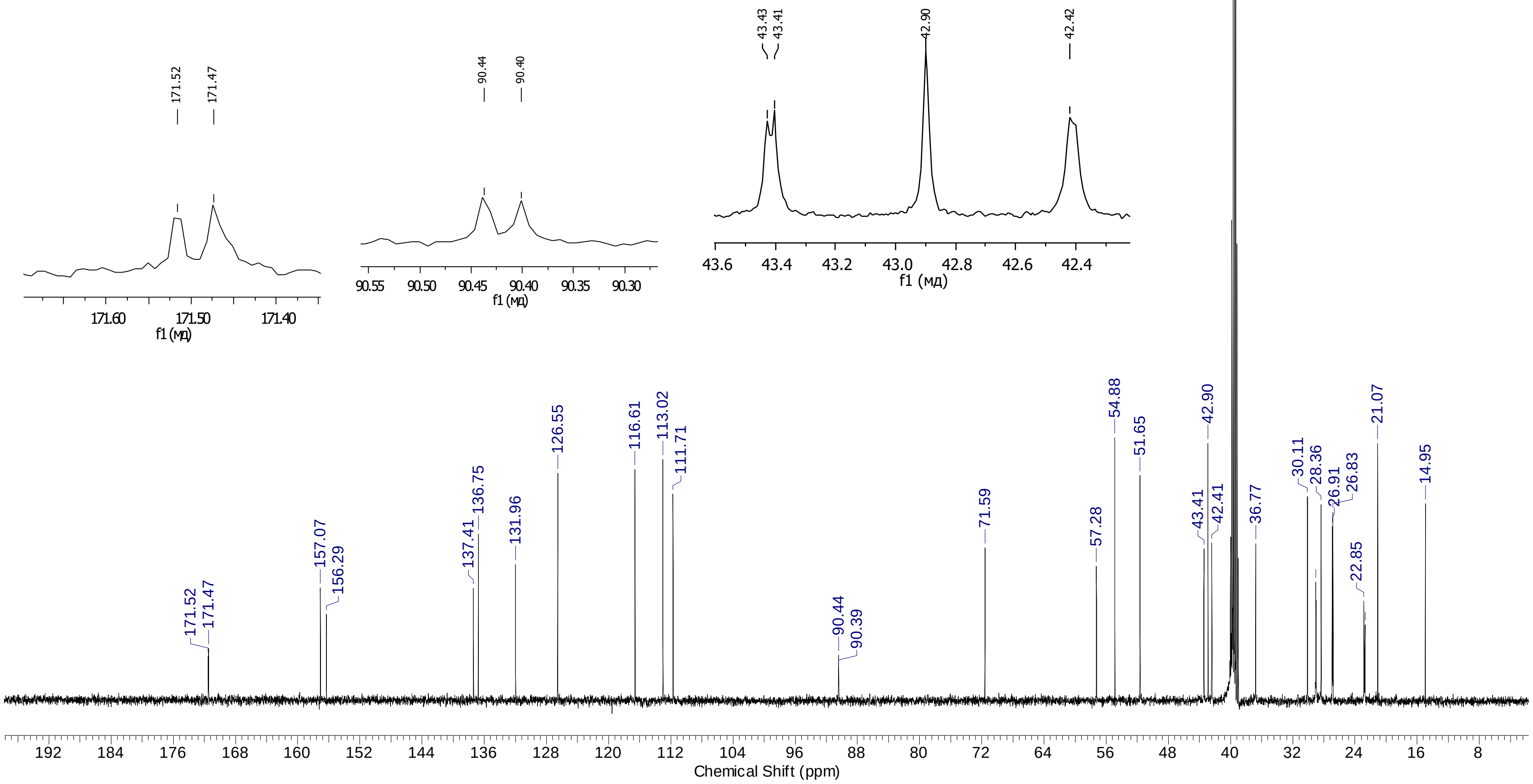

^{13}C NMR (DMSO-d_6, 125 MHz) spectrum of 4-Allyl-1-{3-[(1*S*,2*S*,4a*S*,10a*R*)-2-hydroxy-7-methoxy-2-methyl-1,2,3,4,4a,9,10,10a-octahydrophenanthren-1-yl]propanoyl}-3,5-dimethyl-4,5-dihydro-1*H*-pyrazol-5-ol (**3h**)

MERK5672.esp

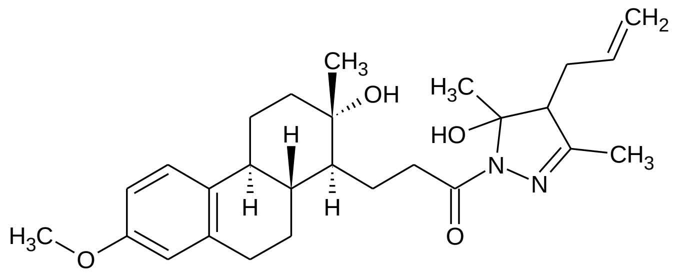

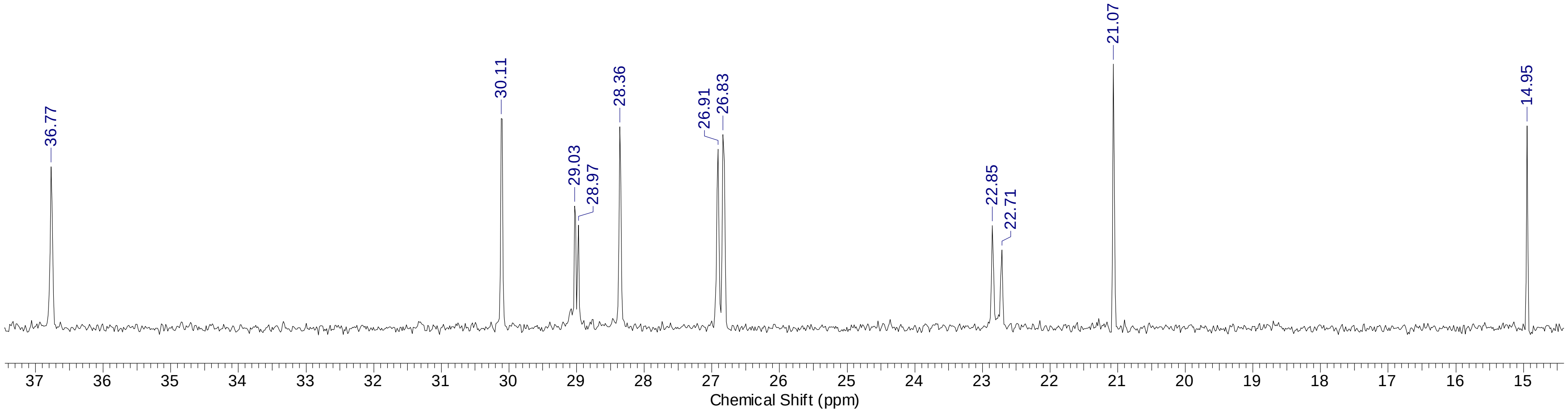

^{1}H NMR (DMSO-d$_6$, 300 MHz) spectrum of 4-Benzyl-1-{3-[(1*S*,2*S*,4a*S*,10a*R*)-2-hydroxy-7-methoxy-2-methyl-1,2,3,4,4a,9,10,10a-octahydrophenanthren-1-yl]propanoyl}-3,5-dimethyl-4,5-dihydro-1*H*-pyrazol-5-ol **(3i)**

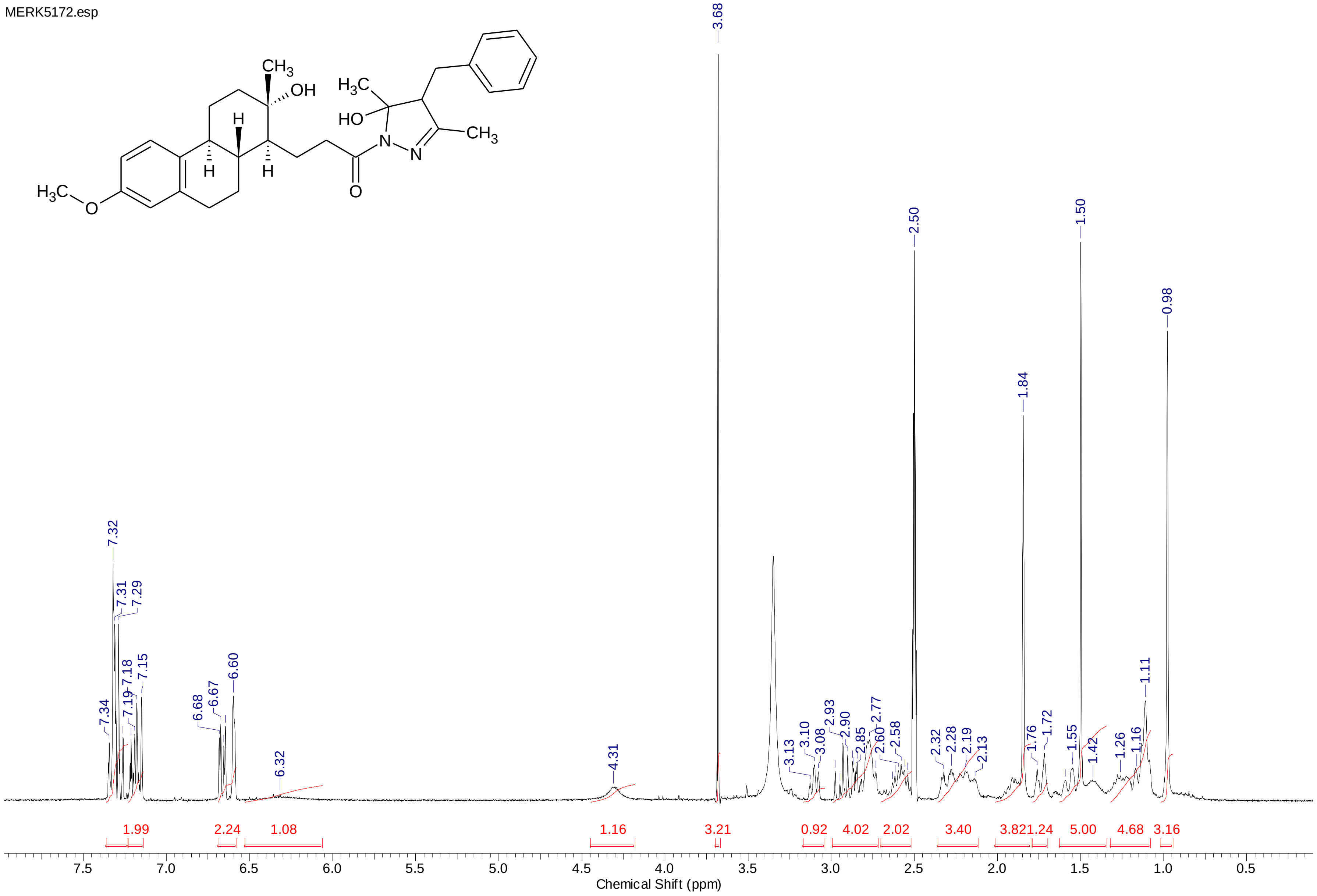

^{13}C NMR (DMSO-d$_6$, 75 MHz) spectrum of 4-Benzyl-1-{3-[(1*S*,2*S*,4a*S*,10a*R*)-2-hydroxy-7-methoxy-2-methyl-1,2,3,4,4a,9,10,10a-octahydrophenanthren-1-yl]propanoyl}-3,5-dimethyl-4,5-dihydro-1*H*-pyrazol-5-ol **(3i)**

MERK5172.esp

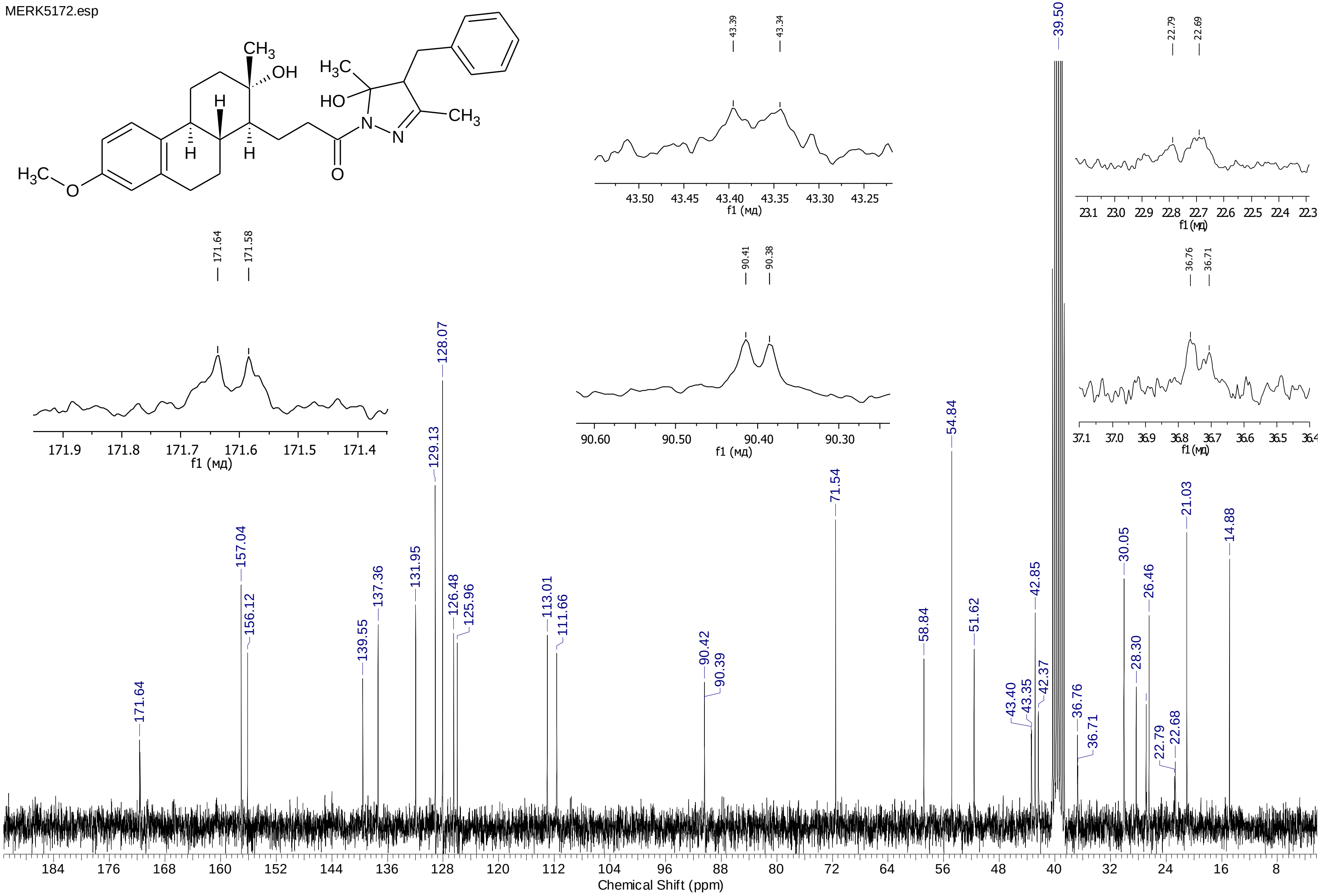

^{1}H NMR (DMSO-d$_6$, 300 MHz) spectrum of 4-(4-Fluorobenzyl)-1-{3-[(1*S*,2*S*,4a*S*,10a*R*)-2-hydroxy-7-methoxy-2-methyl-1,2,3,4,4a,9,10,10a-octahydrophenanthren-1-yl]propanoyl}-3,5-dimethyl-4,5-dihydro-1*H*-pyrazol-5-ol **(3j)**

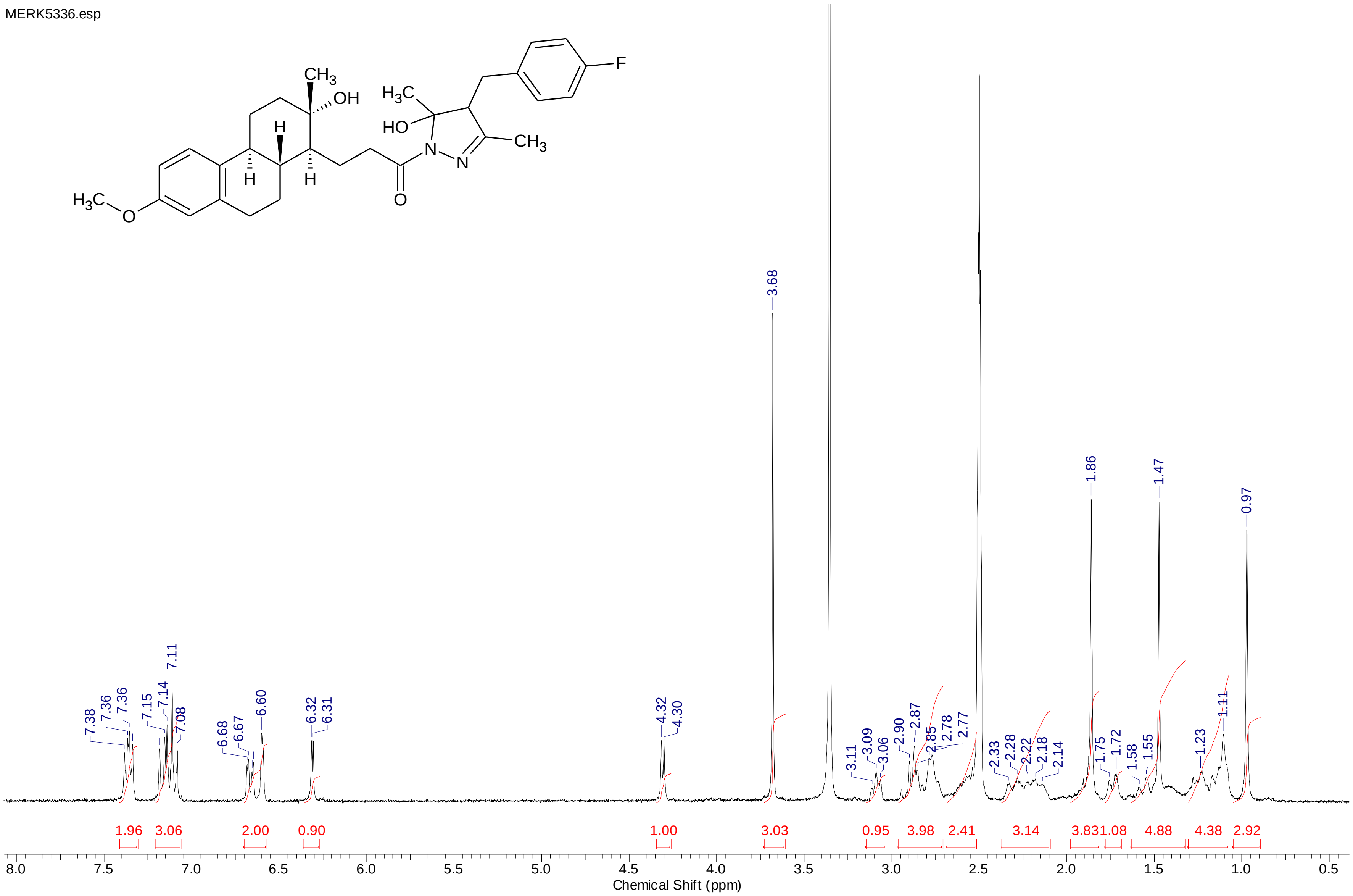

^{13}C NMR (DMSO-d_6, 75 MHz) spectrum of 4-(4-Fluorobenzyl)-1-{3-[(1*S*,2*S*,4a*S*,10a*R*)-2-hydroxy-7-methoxy-2-methyl-1,2,3,4,4a,9,10,10a-octahydrophenanthren-1-yl]propanoyl}-3,5-dimethyl-4,5-dihydro-1*H*-pyrazol-5-ol **(3j)**

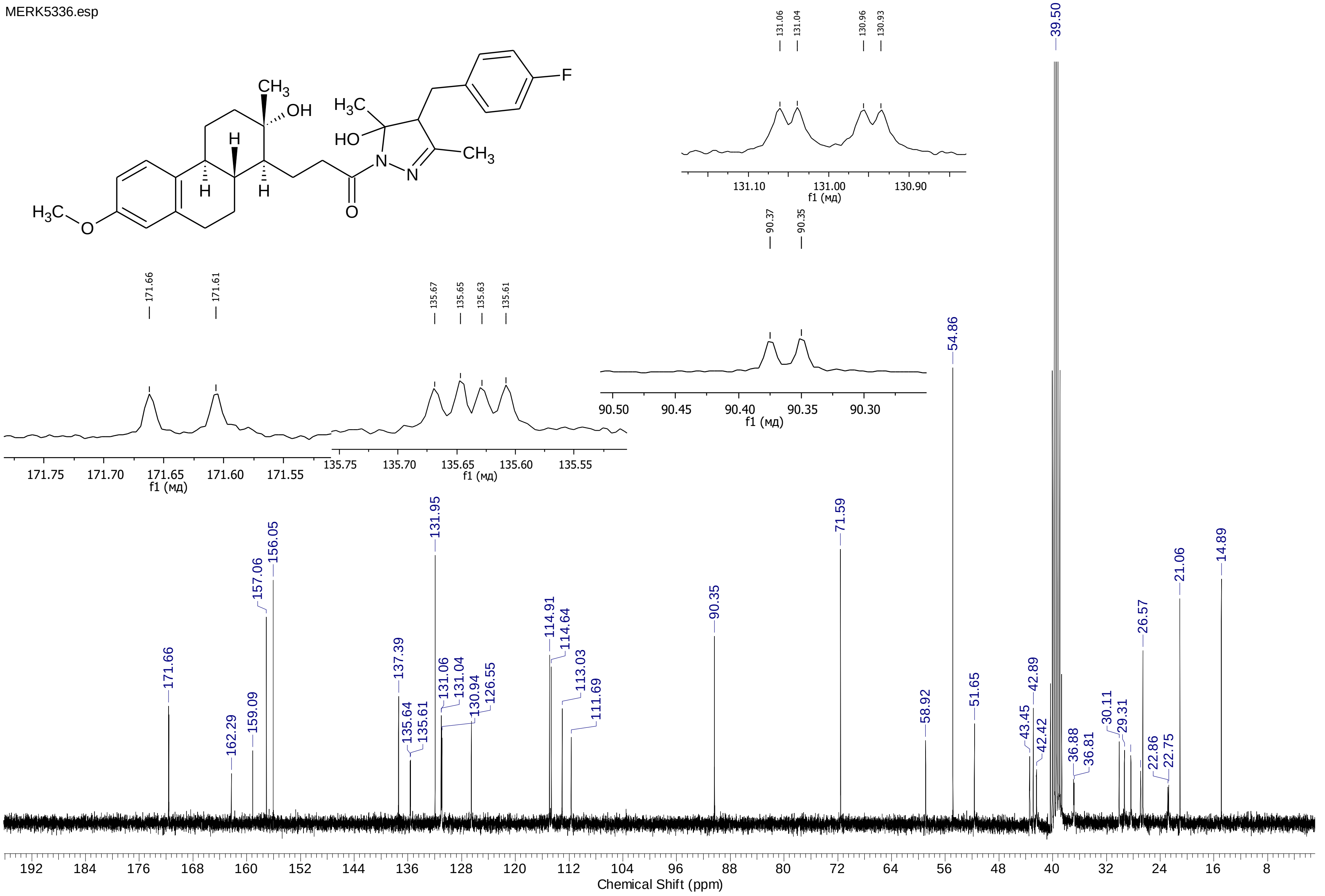

^{13}C NMR (DMSO-d$_6$, 75 MHz) spectrum of 4-(4-Fluorobenzyl)-1-{3-[(1*S*,2*S*,4a*S*,10a*R*)-2-hydroxy-7-methoxy-2-methyl-1,2,3,4,4a,9,10,10a-octahydrophenanthren-1-yl]propanoyl}-3,5-dimethyl-4,5-dihydro-1*H*-pyrazol-5-ol **(3j)**

MERK5336.esp

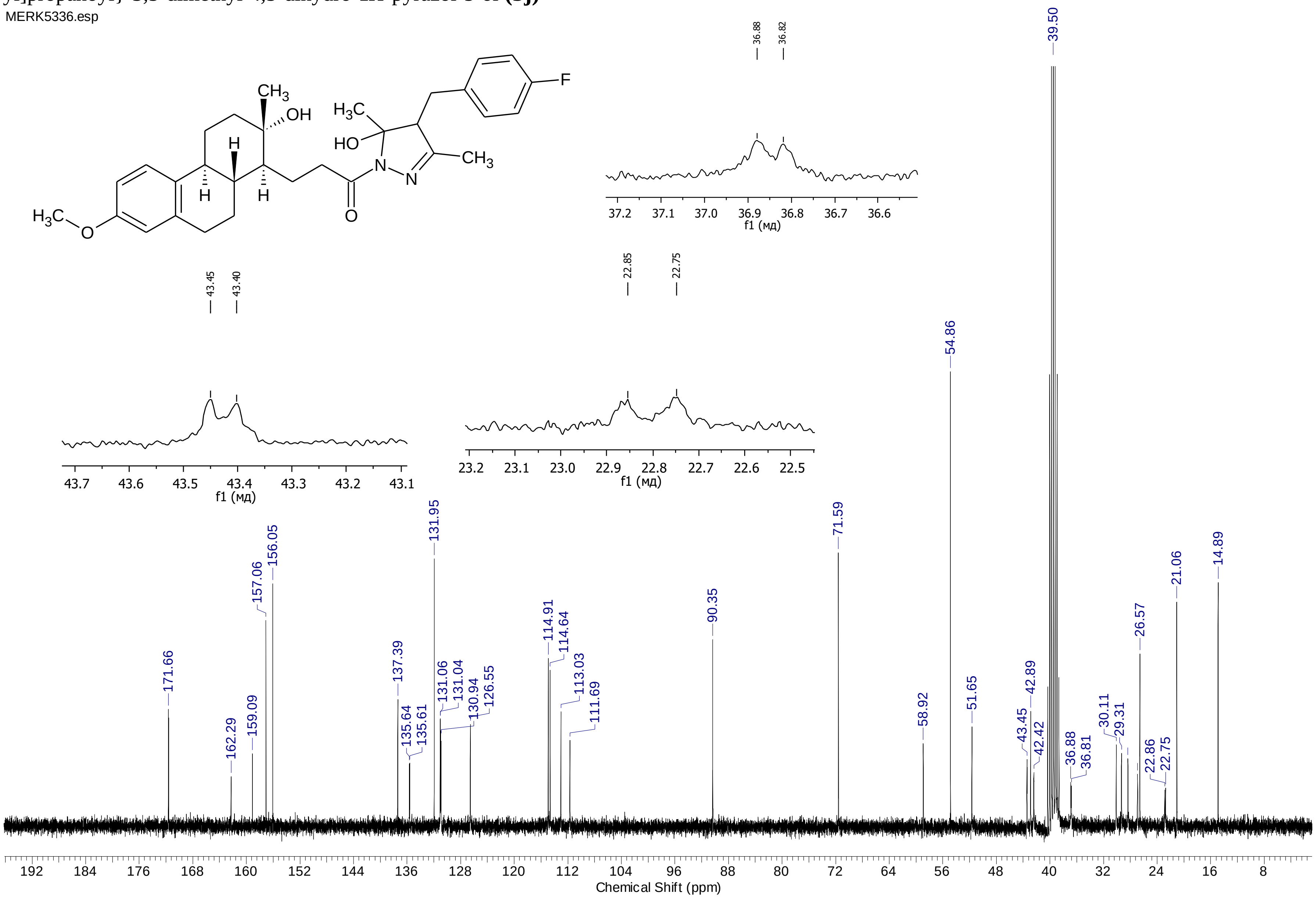

^{19}F NMR (DMSO-d_6, 282 MHz) spectrum of 4-(4-Fluorobenzyl)-1-{3-[(1*S*,2*S*,4a*S*,10a*R*)-2-hydroxy-7-methoxy-2-methyl-1,2,3,4,4a,9,10,10a-octahydrophenanthren-1-yl]propanoyl}-3,5-dimethyl-4,5-dihydro-1*H*-pyrazol-5-ol **(3j)**

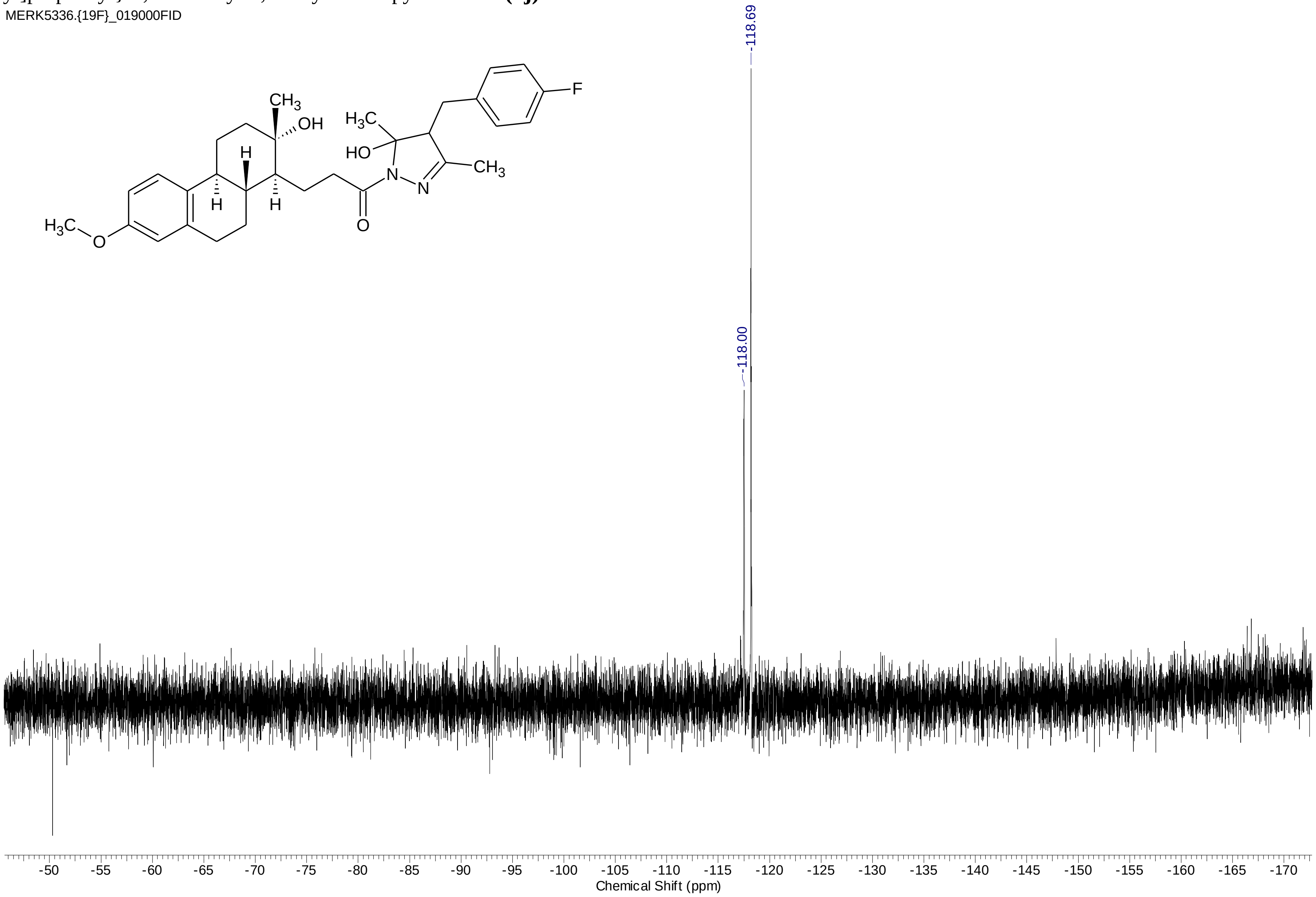

^{1}H NMR (DMSO-d_6, 300 MHz) spectrum of 4-(4-Chlorobenzyl)-1-{3-[(1*S*,2*S*,4a*S*,10a*R*)-2-hydroxy-7-methoxy-2-methyl-1,2,3,4,4a,9,10,10a-octahydrophenanthren-1-yl]propanoyl}-3,5-dimethyl-4,5-dihydro-1*H*-pyrazol-5-ol **(3k)**

MERK5183.esp

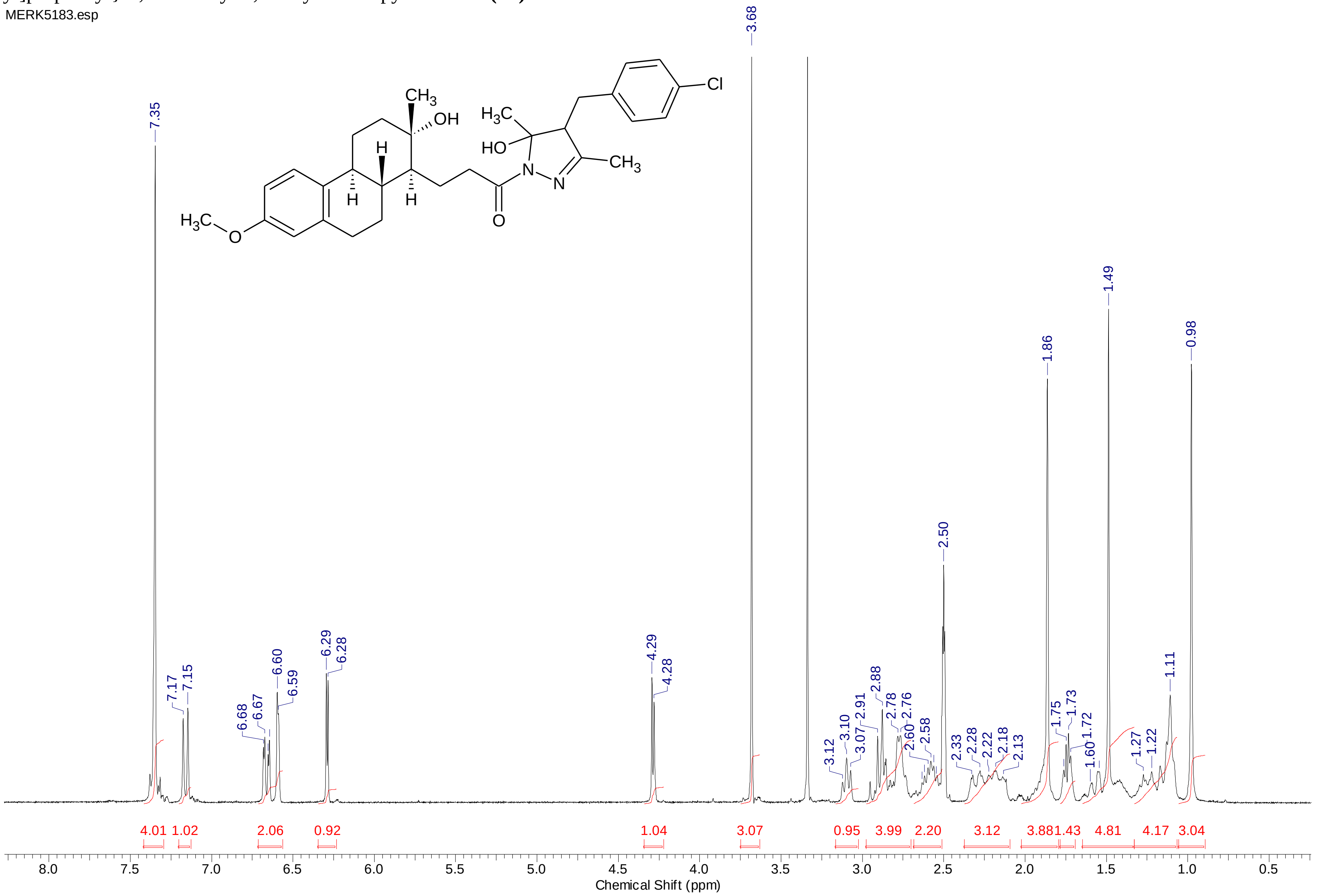

^{13}C NMR (DMSO-d_6, 75 MHz) spectrum of 4-(4-Chlorobenzyl)-1-{3-[(1*S*,2*S*,4a*S*,10a*R*)-2-hydroxy-7-methoxy-2-methyl-1,2,3,4,4a,9,10,10a-octahydrophenanthren-1-yl]propanoyl}-3,5-dimethyl-4,5-dihydro-1*H*-pyrazol-5-ol **(3k)**

MERK5183.esp

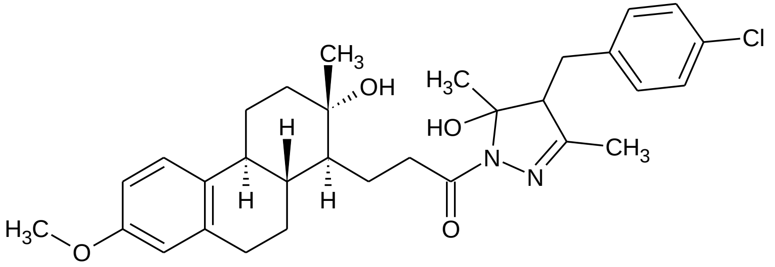

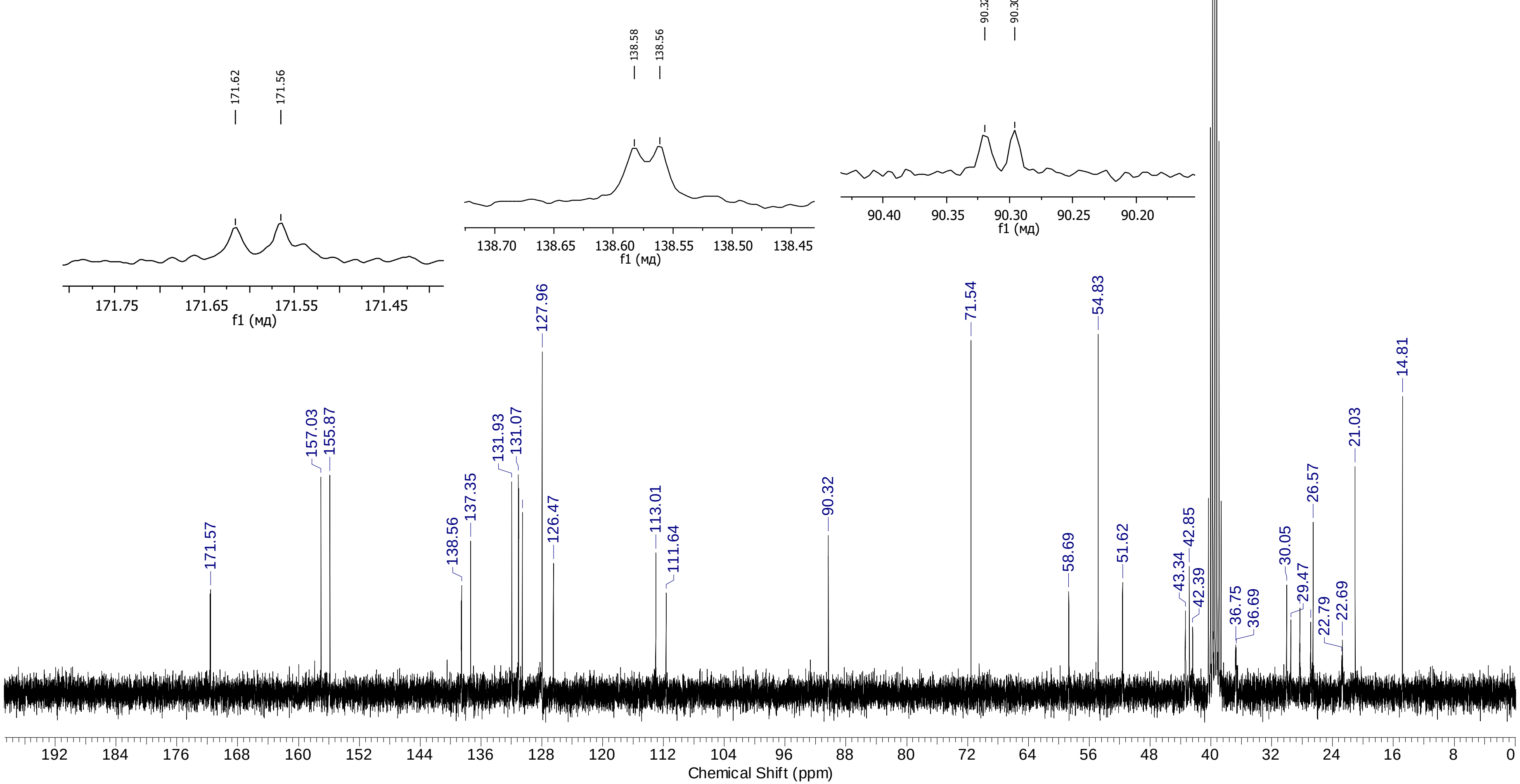

^{13}C NMR (DMSO-d_6, 75 MHz) spectrum of 4-(4-Chlorobenzyl)-1-{3-[(1*S*,2*S*,4a*S*,10a*R*)-2-hydroxy-7-methoxy-2-methyl-1,2,3,4,4a,9,10,10a-octahydrophenanthren-1-yl]propanoyl}-3,5-dimethyl-4,5-dihydro-1*H*-pyrazol-5-ol **(3k)**

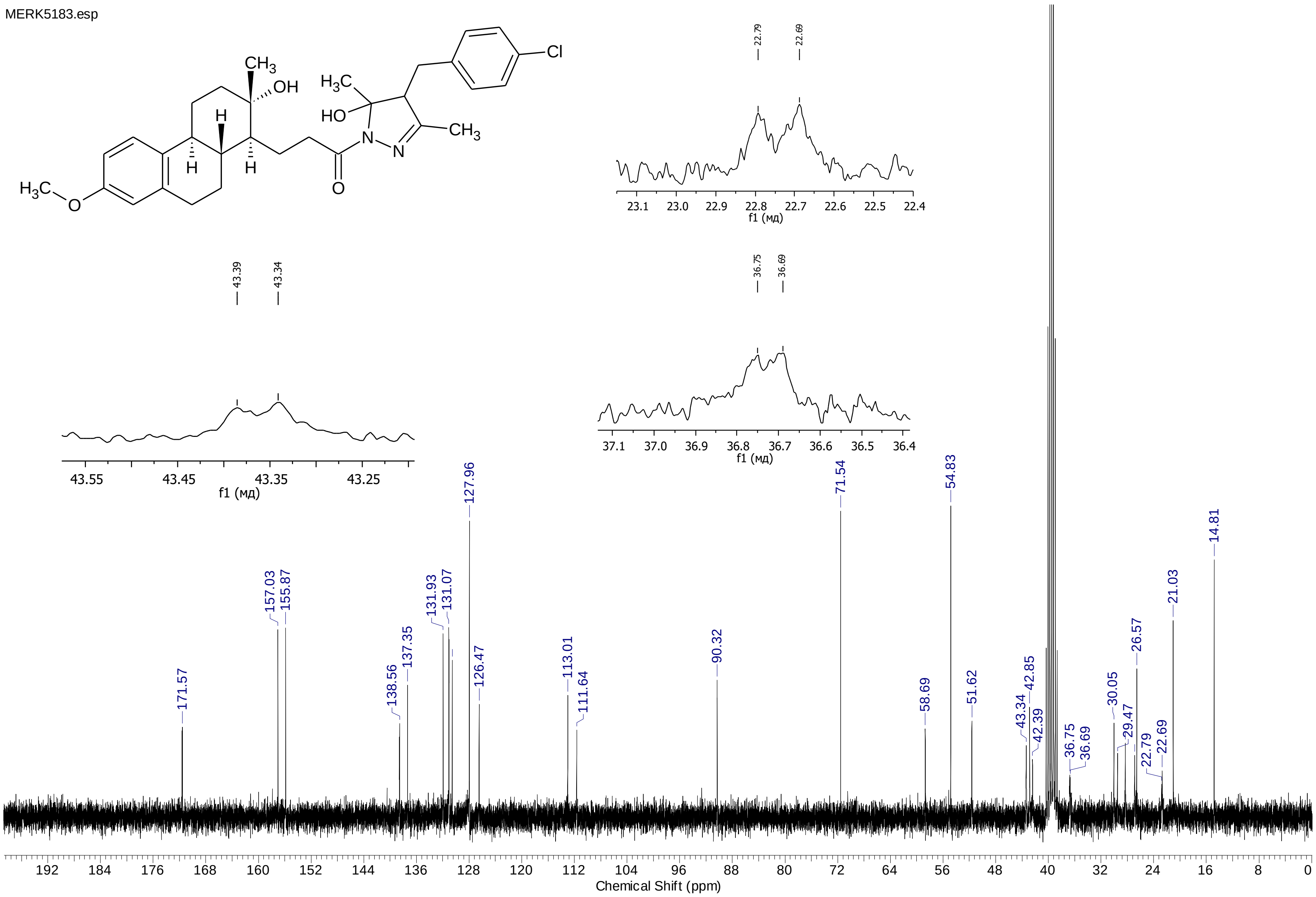

^{1}H NMR (DMSO-d_6, 300 MHz) spectrum of 4-(4-Bromobenzyl)-1-{3-[(1*S*,2*S*,4a*S*,10a*R*)-2-hydroxy-7-methoxy-2-methyl-1,2,3,4,4a,9,10,10a-octahydrophenanthren-1-yl]propanoyl}-3,5-dimethyl-4,5-dihydro-1*H*-pyrazol-5-ol **(3l)**

MERK5308.ESP

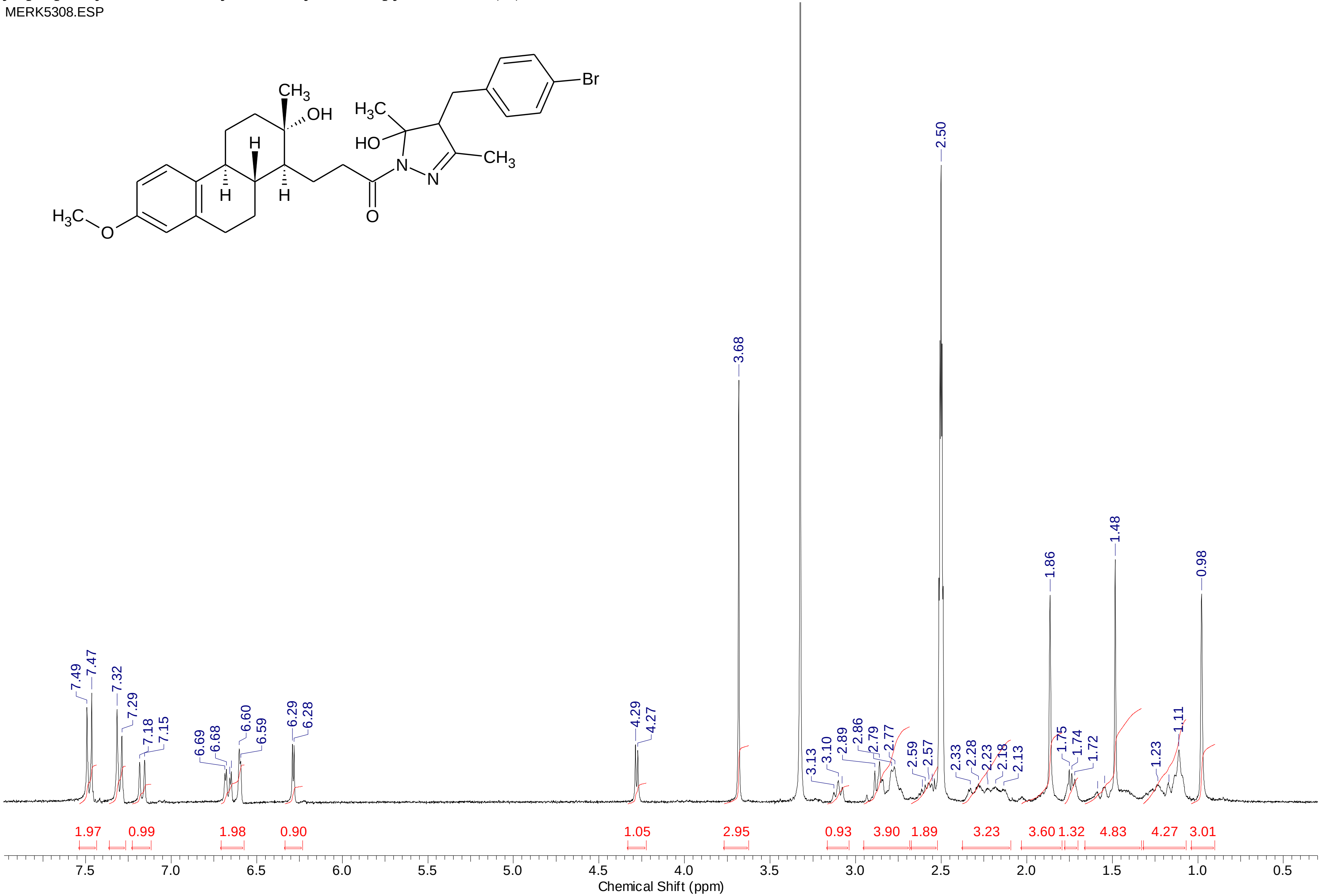

^{13}C NMR (DMSO-d_6, 75 MHz) spectrum of 4-(4-Bromobenzyl)-1-{3-[(1*S*,2*S*,4a*S*,10a*R*)-2-hydroxy-7-methoxy-2-methyl-1,2,3,4,4a,9,10,10a-octahydrophenanthren-1-yl]propanoyl}-3,5-dimethyl-4,5-dihydro-1*H*-pyrazol-5-ol **(3l)**

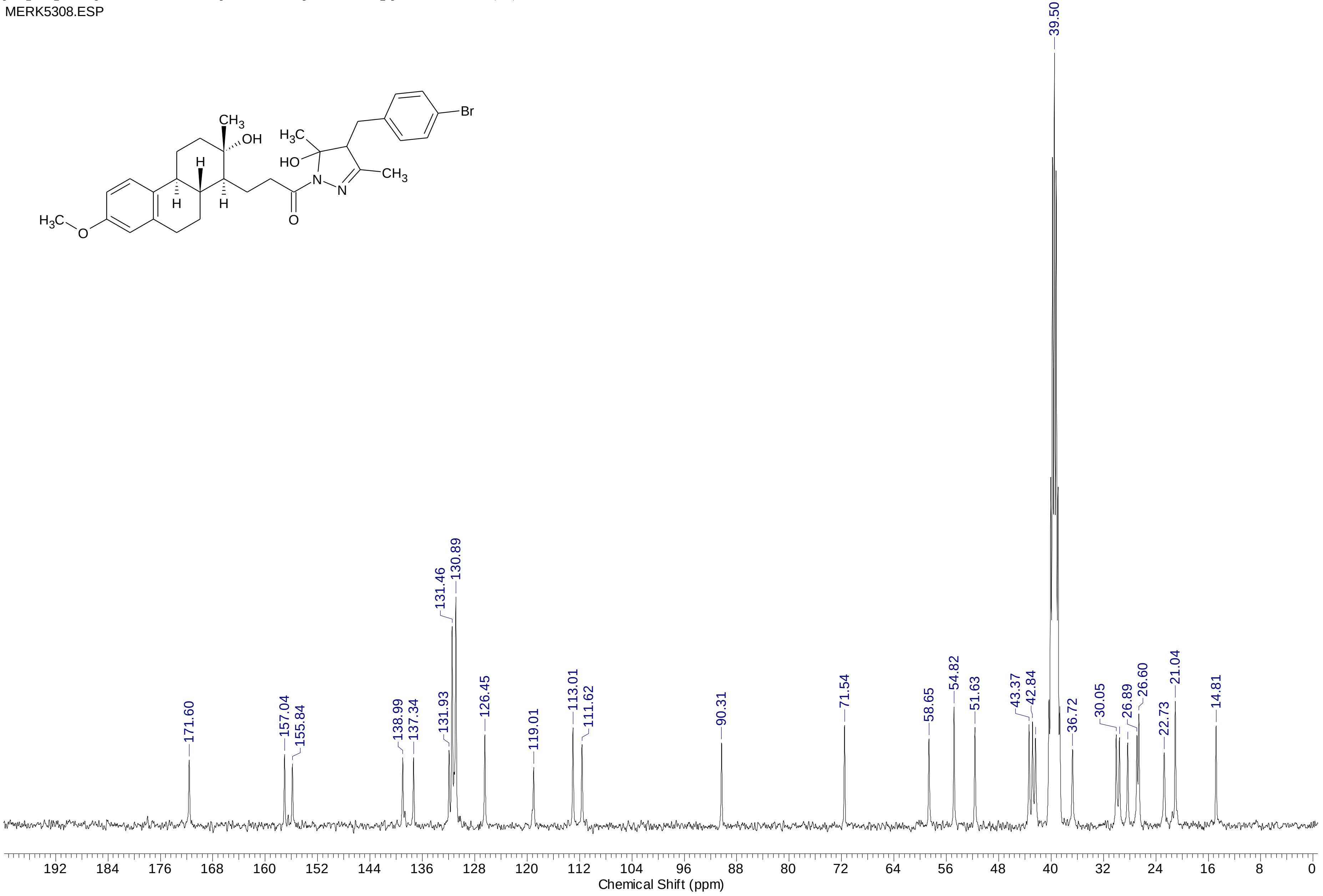

^{1}H NMR (DMSO-d6, 300 MHz) spectrum of 3-(5-Hydroxy-1-{3-[(1*S*,2*S*,4a*S*,10a*R*)-2-hydroxy-7-methoxy-2-methyl-1,2,3,4,4a,9,10,10a-octahydrophenanthren-1-yl]propanoyl}-3,5-dimethyl-4,5-dihydro-1*H*-pyrazol-4-yl)propanenitrile **(3m)**

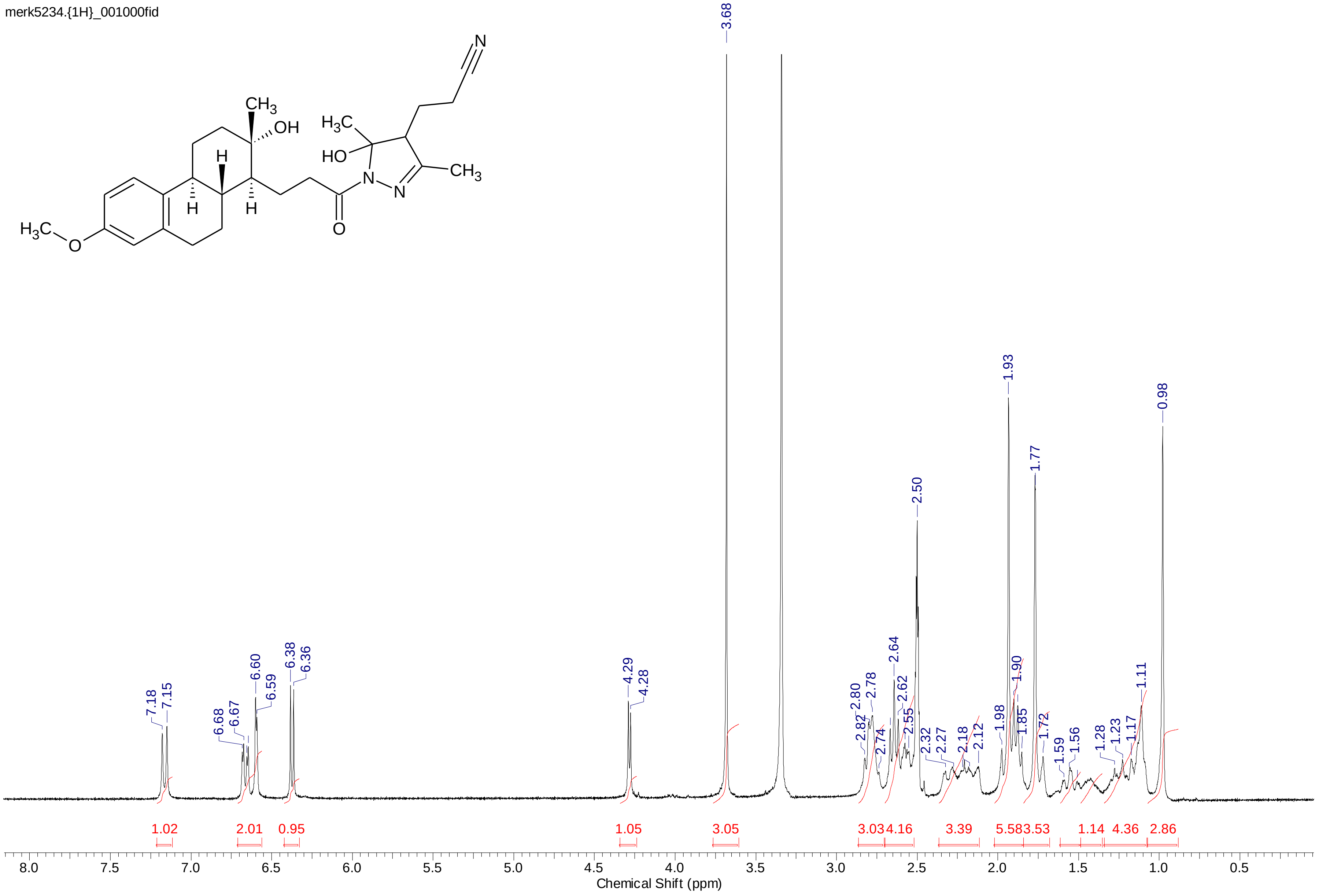

^{13}C NMR (DMSO-d_6, 75 MHz) spectrum of 3-(5-Hydroxy-1-{3-[(1*S*,2*S*,4a*S*,10a*R*)-2-hydroxy-7-methoxy-2-methyl-1,2,3,4,4a,9,10,10a-octahydrophenanthren-1-yl]propanoyl}-3,5-dimethyl-4,5-dihydro-1*H*-pyrazol-4-yl)propanenitrile **(3m)**

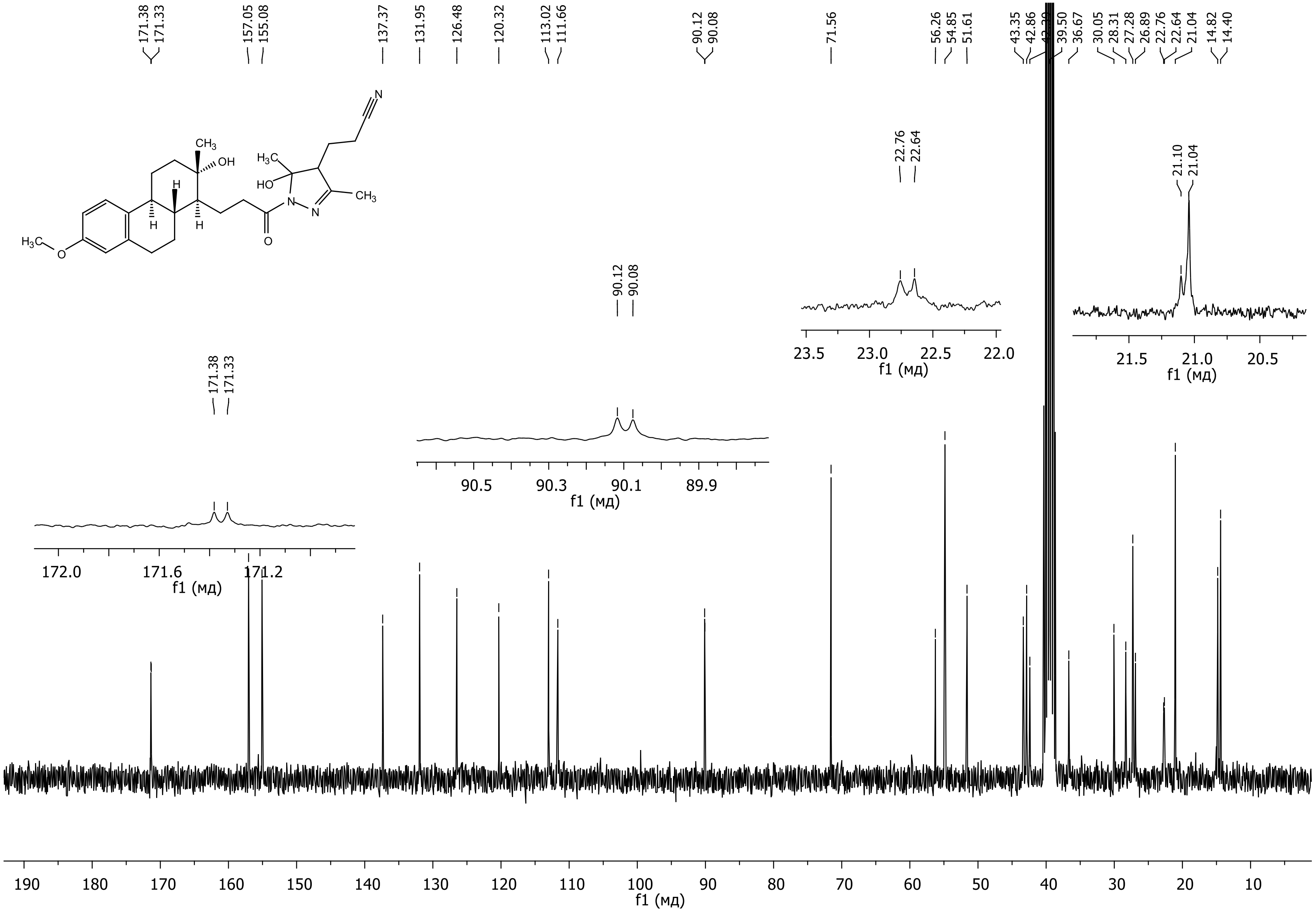

^{1}H NMR (DMSO-d$_6$, 600 MHz) spectrum of Ethyl 3-(5-hydroxy-1-{3-[(1*S*,2*S*,4a*S*,10a*R*)-2-hydroxy-7-methoxy-2-methyl-1,2,3,4,4a,9,10,10a-octahydrophenanthren-1-yl]propanoyl}-3,5-dimethyl-4,5-dihydro-1*H*-pyrazol-4-yl)propanoate (**3n**)

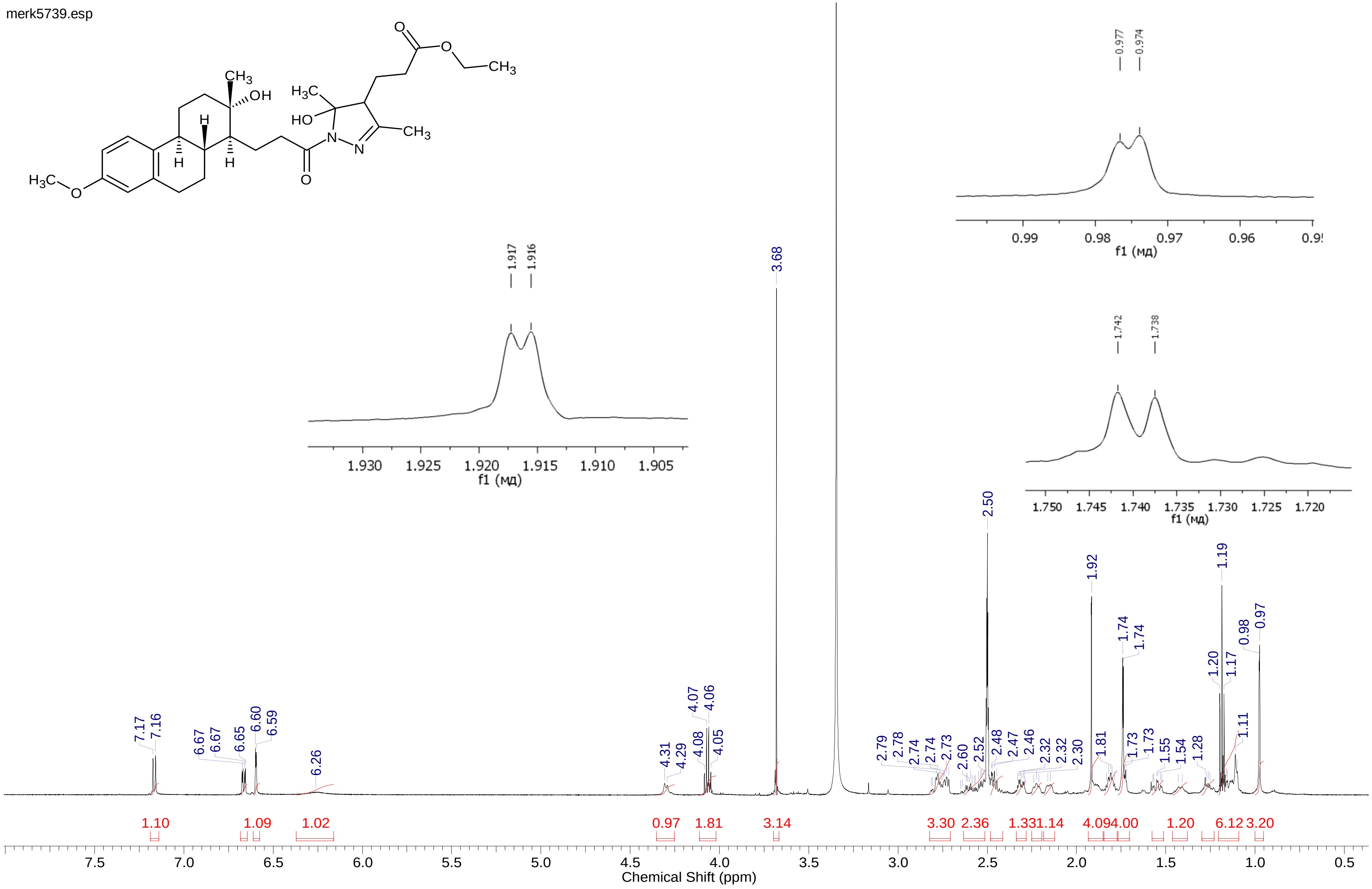

^{13}C NMR (DMSO-d$_6$, 150 MHz) spectrum of Ethyl 3-(5-hydroxy-1-{3-[(1*S*,2*S*,4a*S*,10a*R*)-2-hydroxy-7-methoxy-2-methyl-1,2,3,4,4a,9,10,10a-octahydrophenanthren-1-yl]propanoyl}-3,5-dimethyl-4,5-dihydro-1*H*-pyrazol-4-yl)propanoate (**3n**)

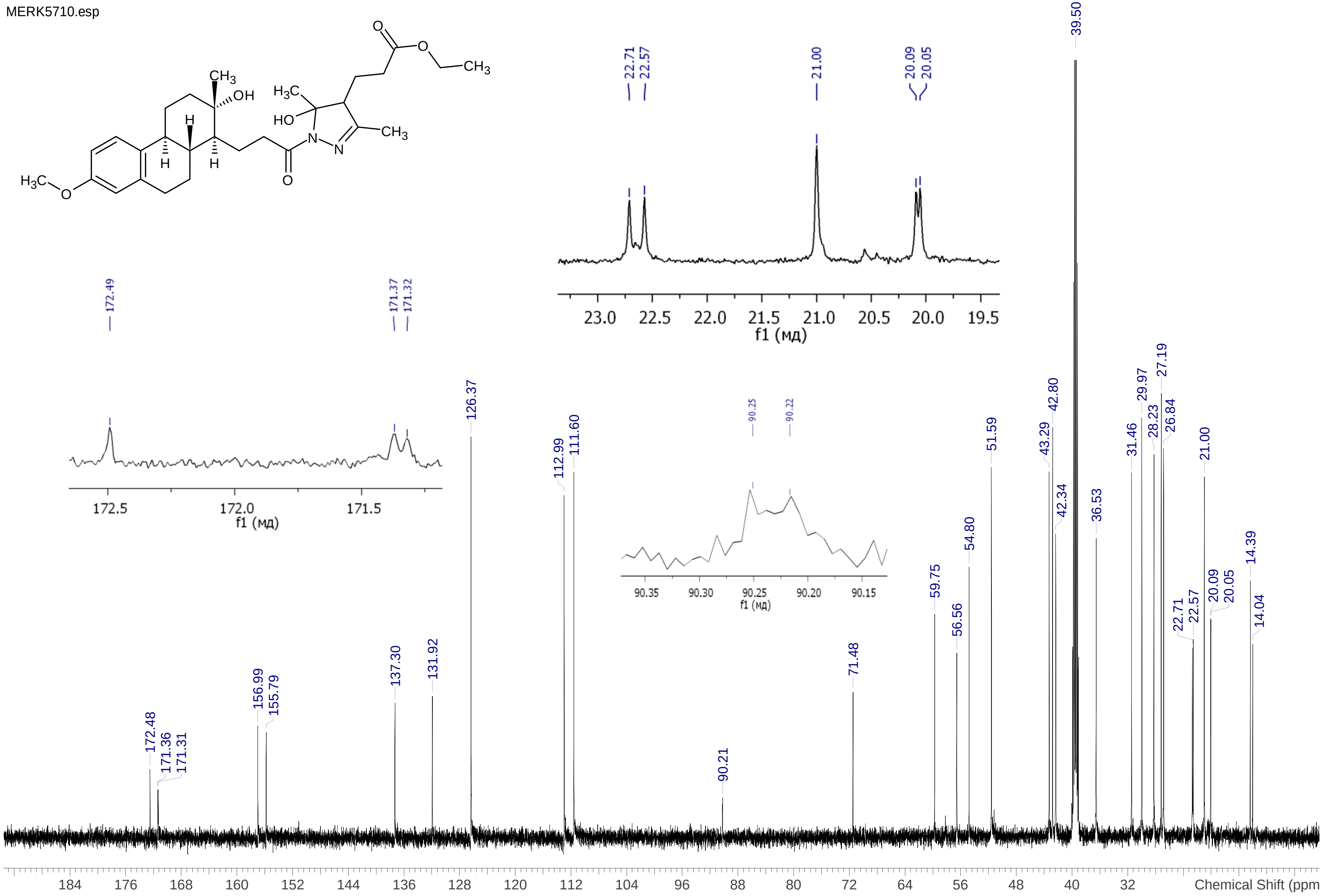

^{1}H NMR (DMSO-d_6, 600 MHz) spectrum of 3,5-Diethyl-1-{3-[(1*S*,2*S*,4a*S*,10a*R*)-2-hydroxy-7-methoxy-2-methyl-1,2,3,4,4a,9,10,10a-octahydrophenanthren-1-yl]propanoyl}-4,5-dihydro-1*H*-pyrazol-5-ol **(3o)**

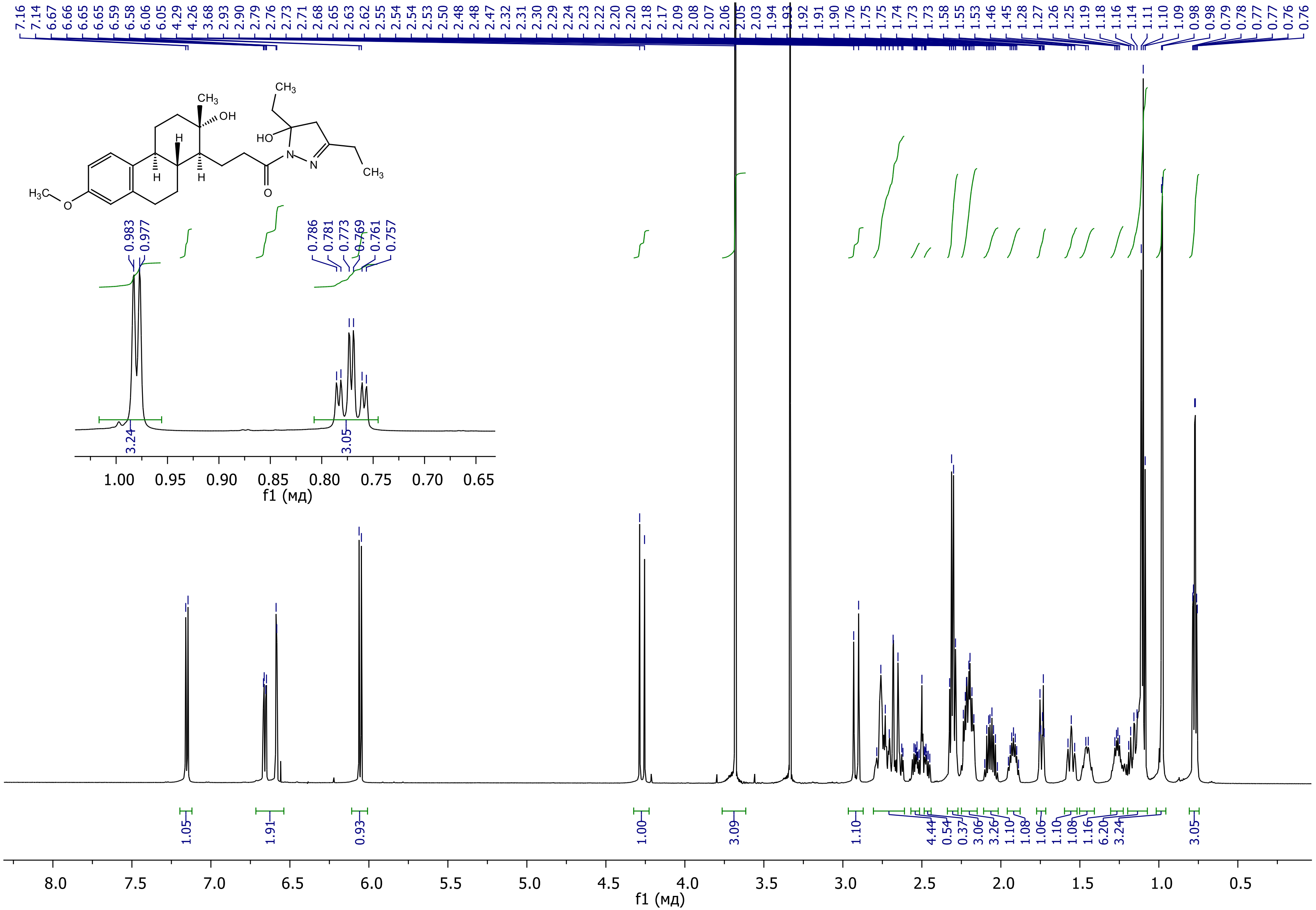

^{13}C NMR (DMSO-d_6, 150 MHz) spectrum of 3,5-Diethyl-1-{3-[(1*S*,2*S*,4a*S*,10a*R*)-2-hydroxy-7-methoxy-2-methyl-1,2,3,4,4a,9,10,10a-octahydrophenanthren-1-yl]propanoyl}-4,5-dihydro-1*H*-pyrazol-5-ol **(3o)**

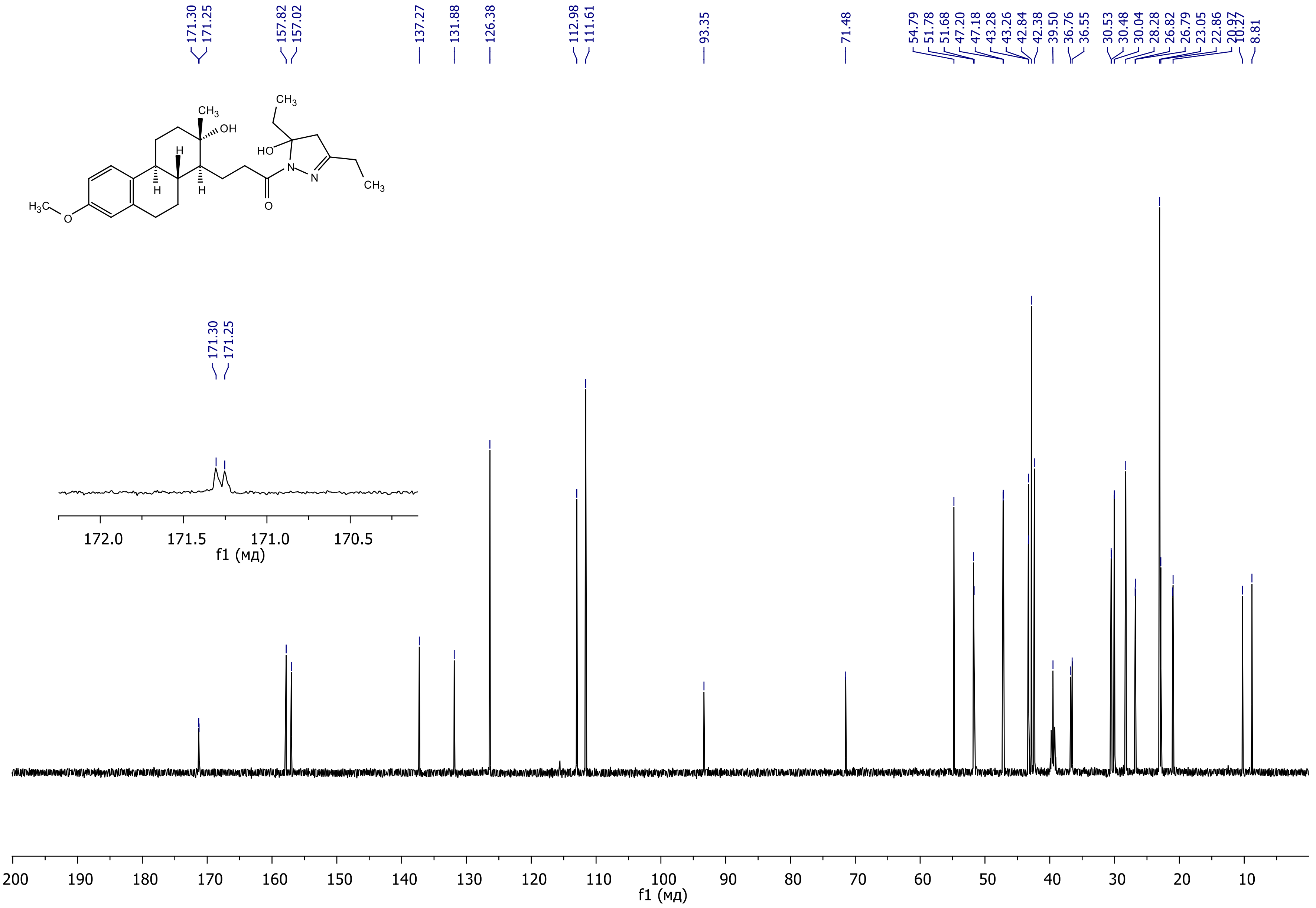

^{13}C NMR (DMSO-d$_6$, 150 MHz) spectrum of 3,5-Diethyl-1-{3-[(1*S*,2*S*,4a*S*,10a*R*)-2-hydroxy-7-methoxy-2-methyl-1,2,3,4,4a,9,10,10a-octahydrophenanthren-1-yl]propanoyl}-4,5-dihydro-1*H*-pyrazol-5-ol **(3o)**

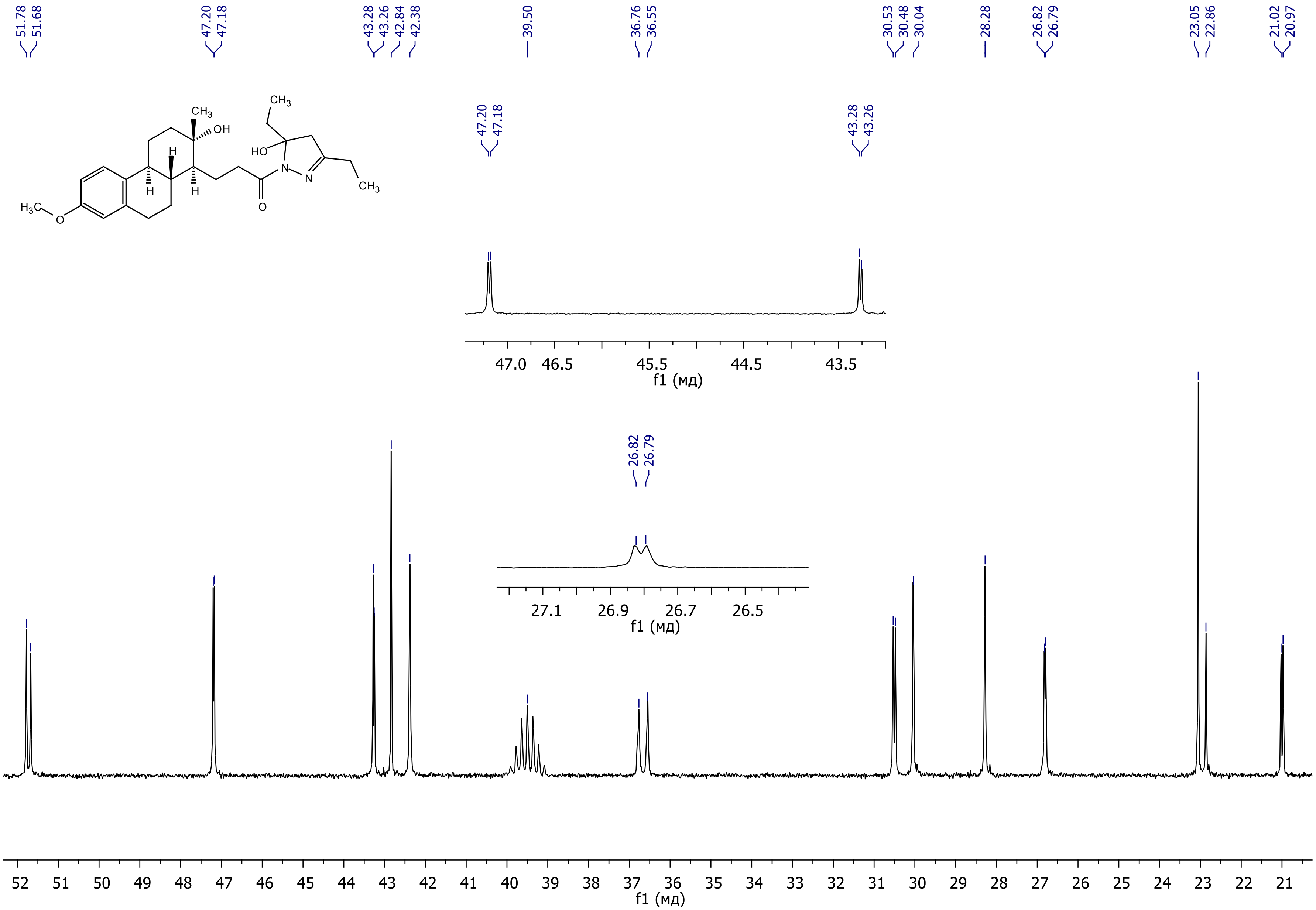

^{1}H NMR (DMSO-d_6, 300 MHz) of 3,5-Diethyl-1-{3-[(1*S*,2*S*,4a*S*,10a*R*)-2-hydroxy-7-methoxy-2-methyl-1,2,3,4,4a,9,10,10a-octahydrophenanthren-1-yl]propanoyl}-4-methyl-4,5-dihydro-1*H*-pyrazol-5-ol (**3p**)

MERK5297-1.esp

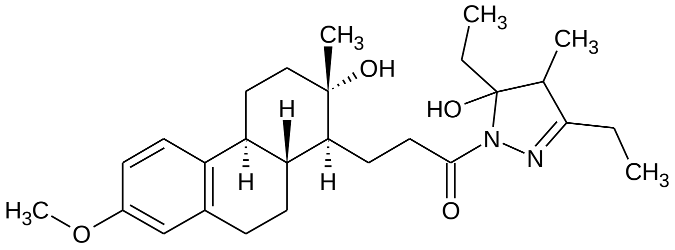

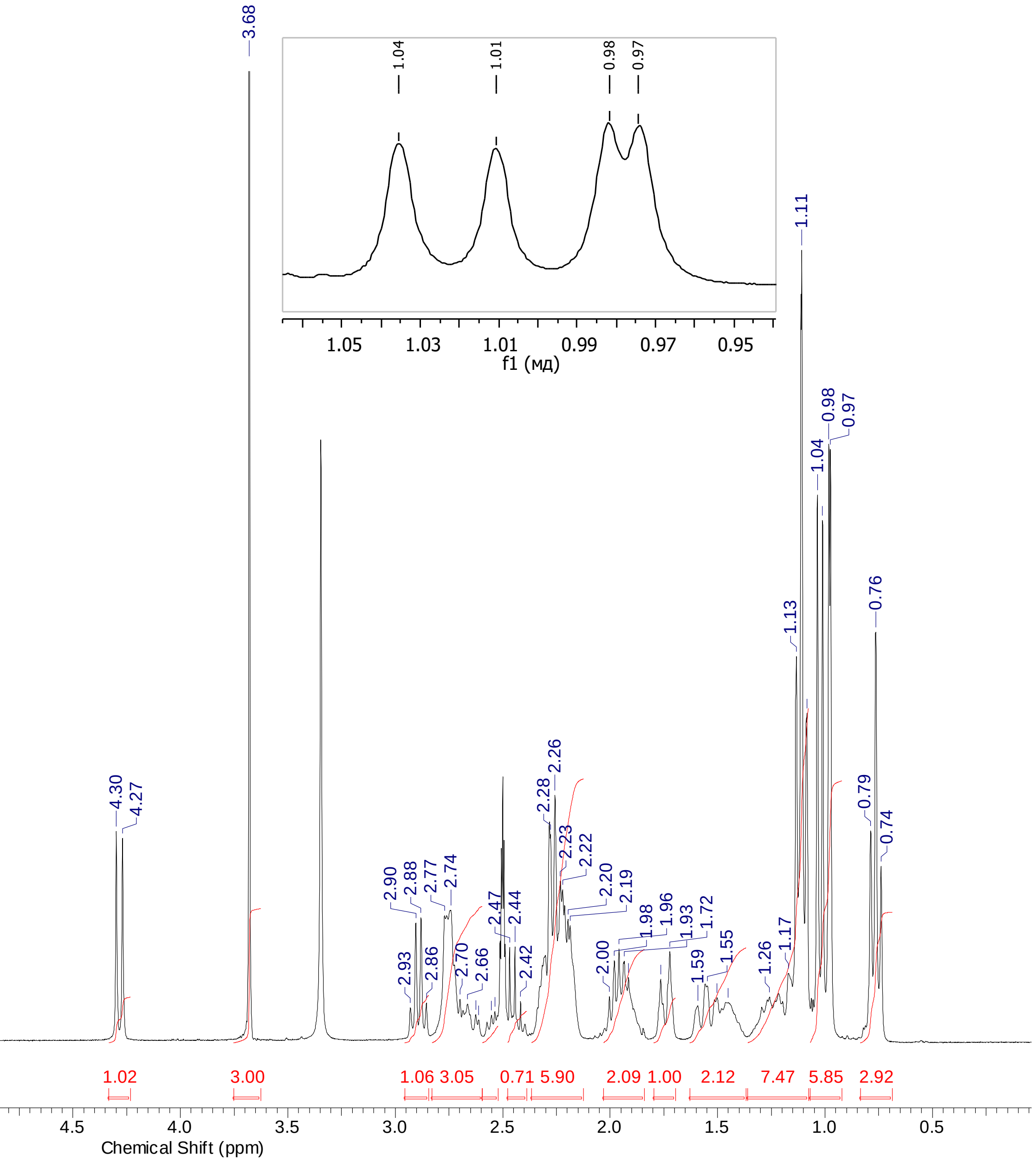

^{13}C NMR (DMSO-d_6, 75 MHz) spectrum of 3,5-Diethyl-1-{3-[(1*S*,2*S*,4a*S*,10a*R*)-2-hydroxy-7-methoxy-2-methyl-1,2,3,4,4a,9,10,10a-octahydrophenanthren-1-yl]propanoyl}-4-methyl-4,5-dihydro-1*H*-pyrazol-5-ol (**3p**)

MERK5297-1.ESP

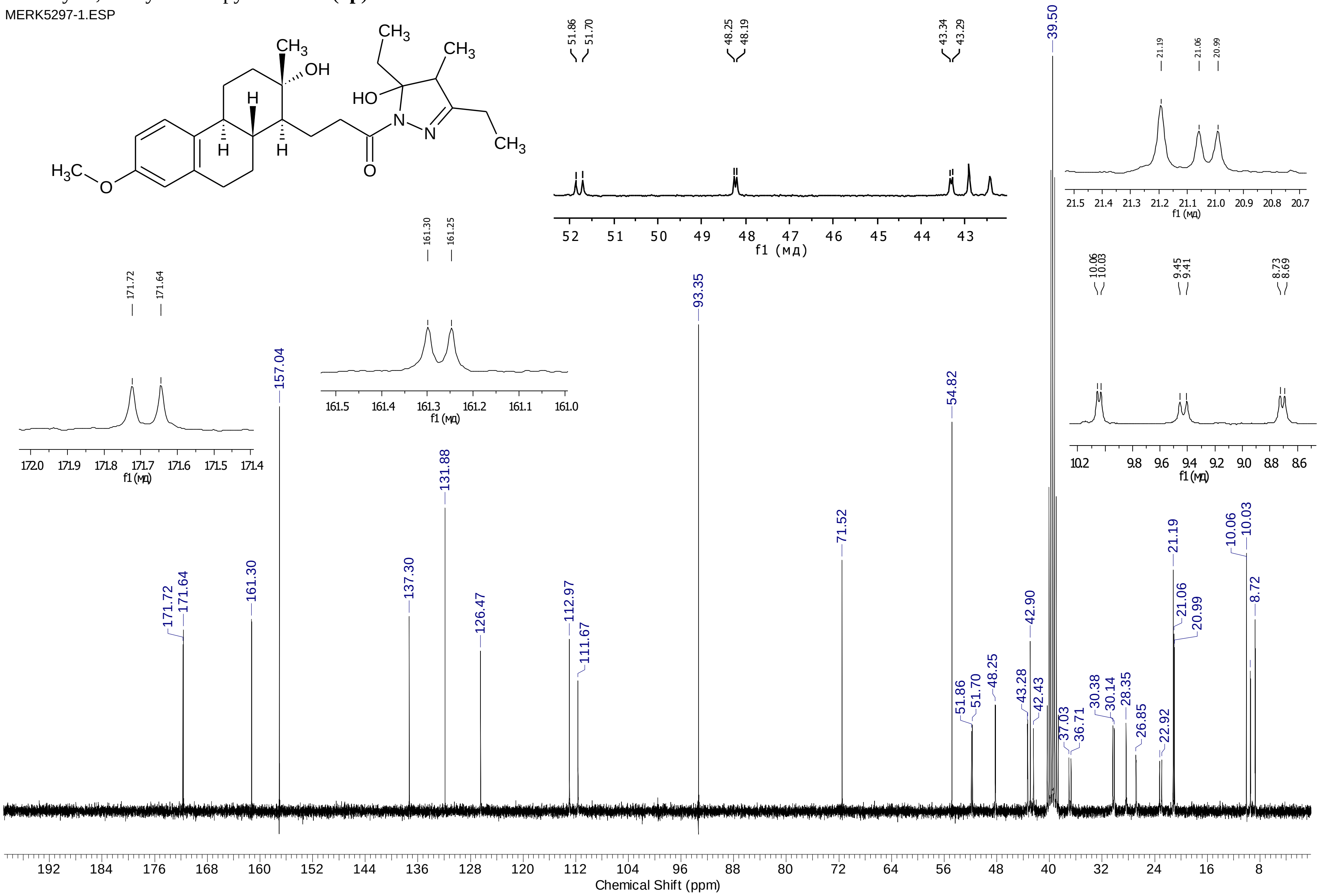

^{1}H NMR (DMSO-d$_6$, 300 MHz) of 1-{3-[(1*S*,2*S*,4a*S*,10a*R*)-2-Hydroxy-7-methoxy-2-methyl-1,2,3,4,4a,9,10,10a-octahydrophenanthren-1-yl]propanoyl}-3-methyl-5-phenyl-4,5-dihydro-1*H*-pyrazol-5-ol (**3q**)

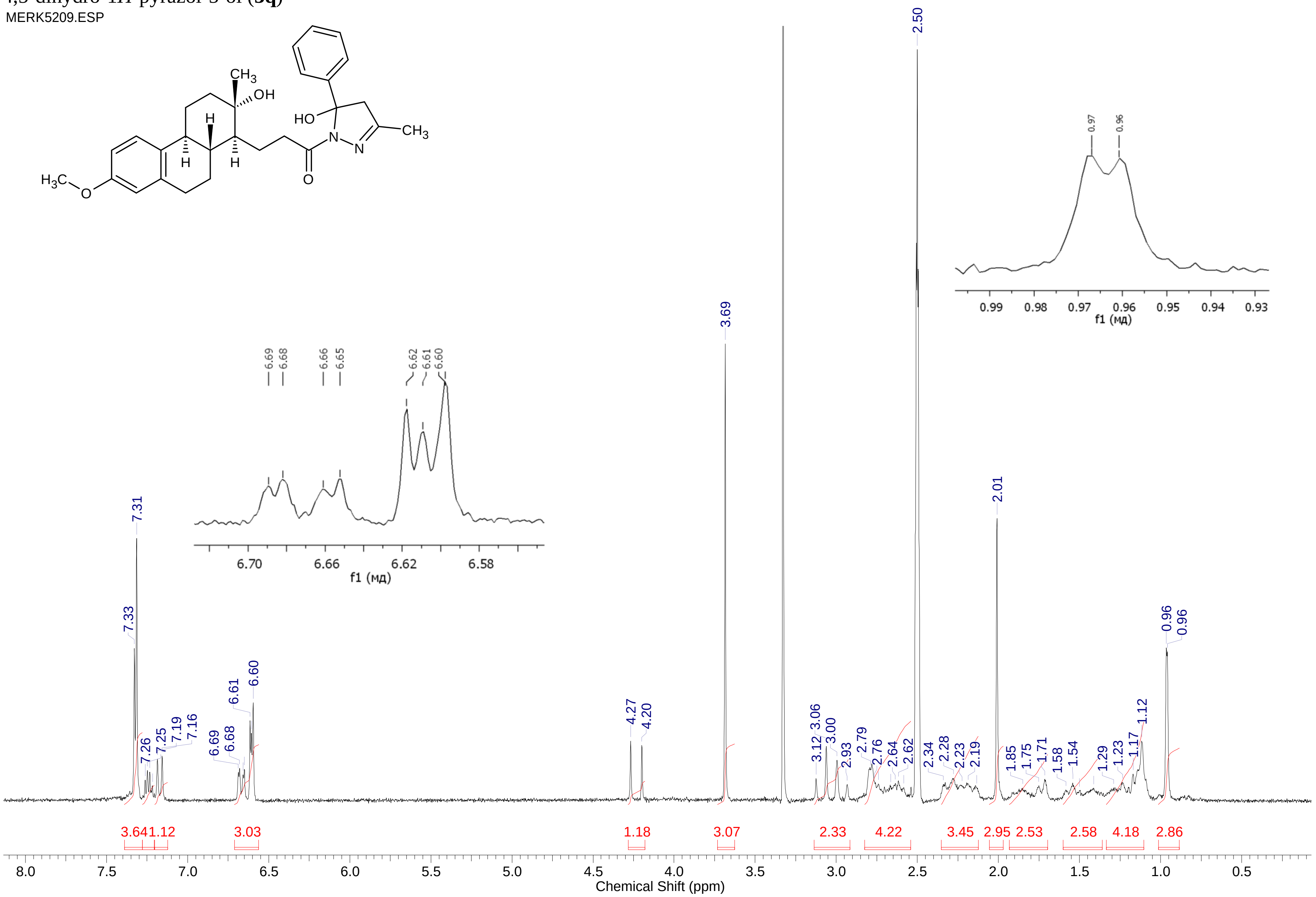

^{13}C NMR (DMSO-d_6, 75 MHz) spectrum of 1-{3-[(1*S*,2*S*,4a*S*,10a*R*)-2-Hydroxy-7-methoxy-2-methyl-1,2,3,4,4a,9,10,10a-octahydrophenanthren-1-yl]propanoyl}-3-methyl-5-phenyl-4,5-dihydro-1*H*-pyrazol-5-ol (**3q**)

MERK5293.esp

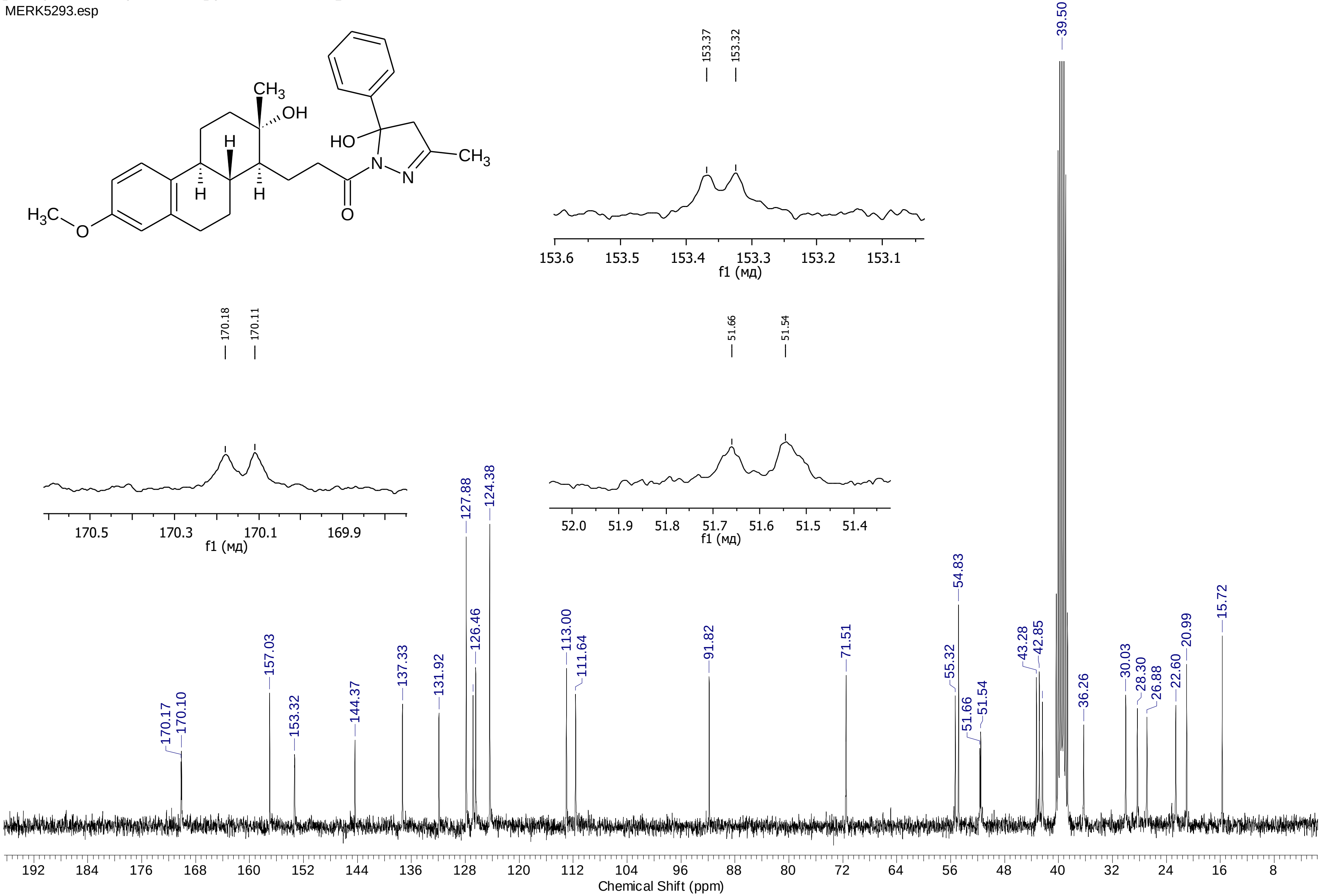

^{13}C NMR (DMSO-d_6, 75 MHz) spectrum of 1-{3-[(1*S*,2*S*,4a*S*,10a*R*)-2-Hydroxy-7-methoxy-2-methyl-1,2,3,4,4a,9,10,10a-octahydrophenanthren-1-yl]propanoyl}-3-methyl-5-phenyl-4,5-dihydro-1*H*-pyrazol-5-ol (**3q**)

MERK5293.esp

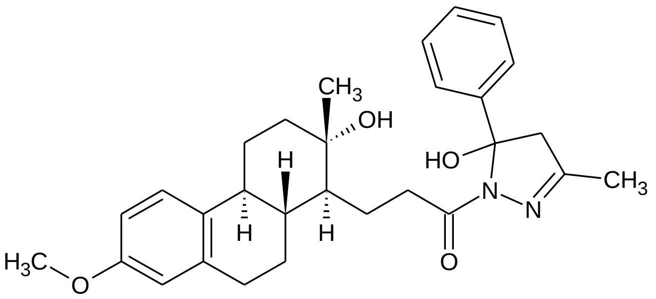

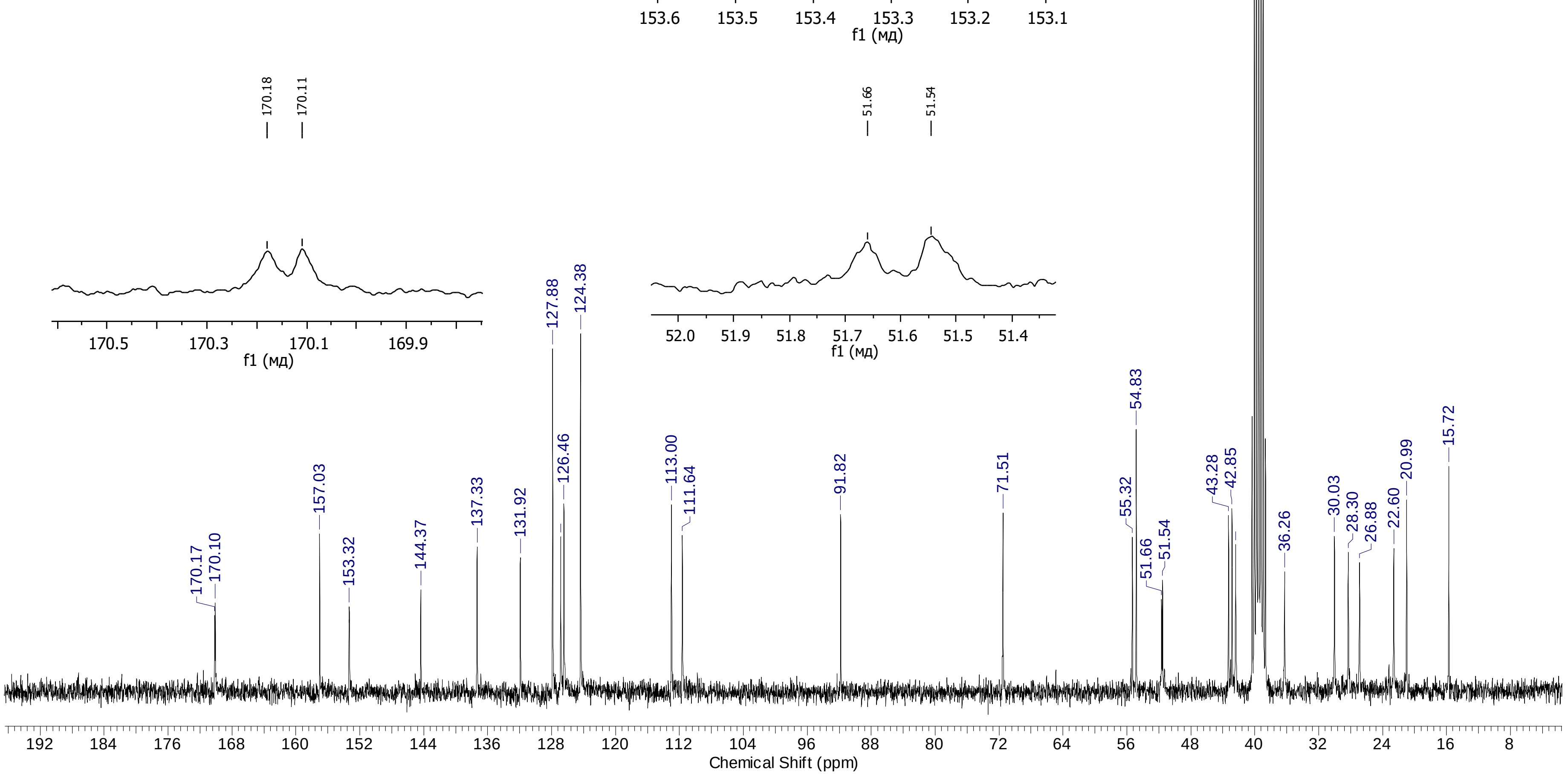

^{1}H NMR (DMSO-d$_6$, 600 MHz) of 5-(4-Fluorophenyl)-1-{3-[(1*S*,2*S*,4a*S*,10a*R*)-2-hydroxy-7-methoxy-2-methyl-1,2,3,4,4a,9,10,10a-octahydrophenanthren-1-yl]propanoyl}-3-methyl-4,5-dihydro-1*H*-pyrazol-5-ol (**3r**)

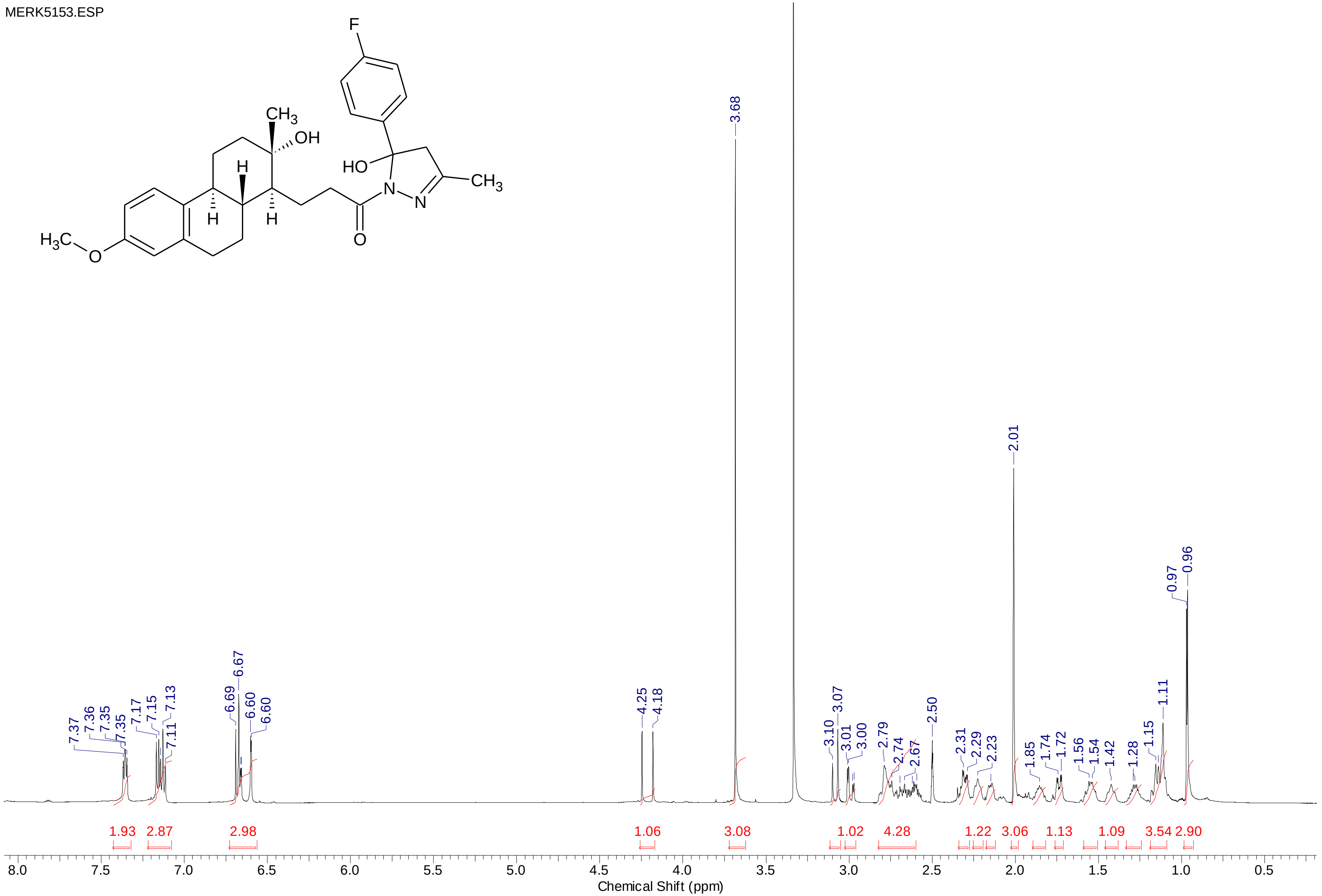

^{13}C NMR (DMSO-d$_6$, 150 MHz) spectrum of 5-(4-Fluorophenyl)-1-{3-[(1*S*,2*S*,4a*S*,10a*R*)-2-hydroxy-7-methoxy-2-methyl-1,2,3,4,4a,9,10,10a-octahydrophenanthren-1-yl]propanoyl}-3-methyl-4,5-dihydro-1*H*-pyrazol-5-ol (**3r**)

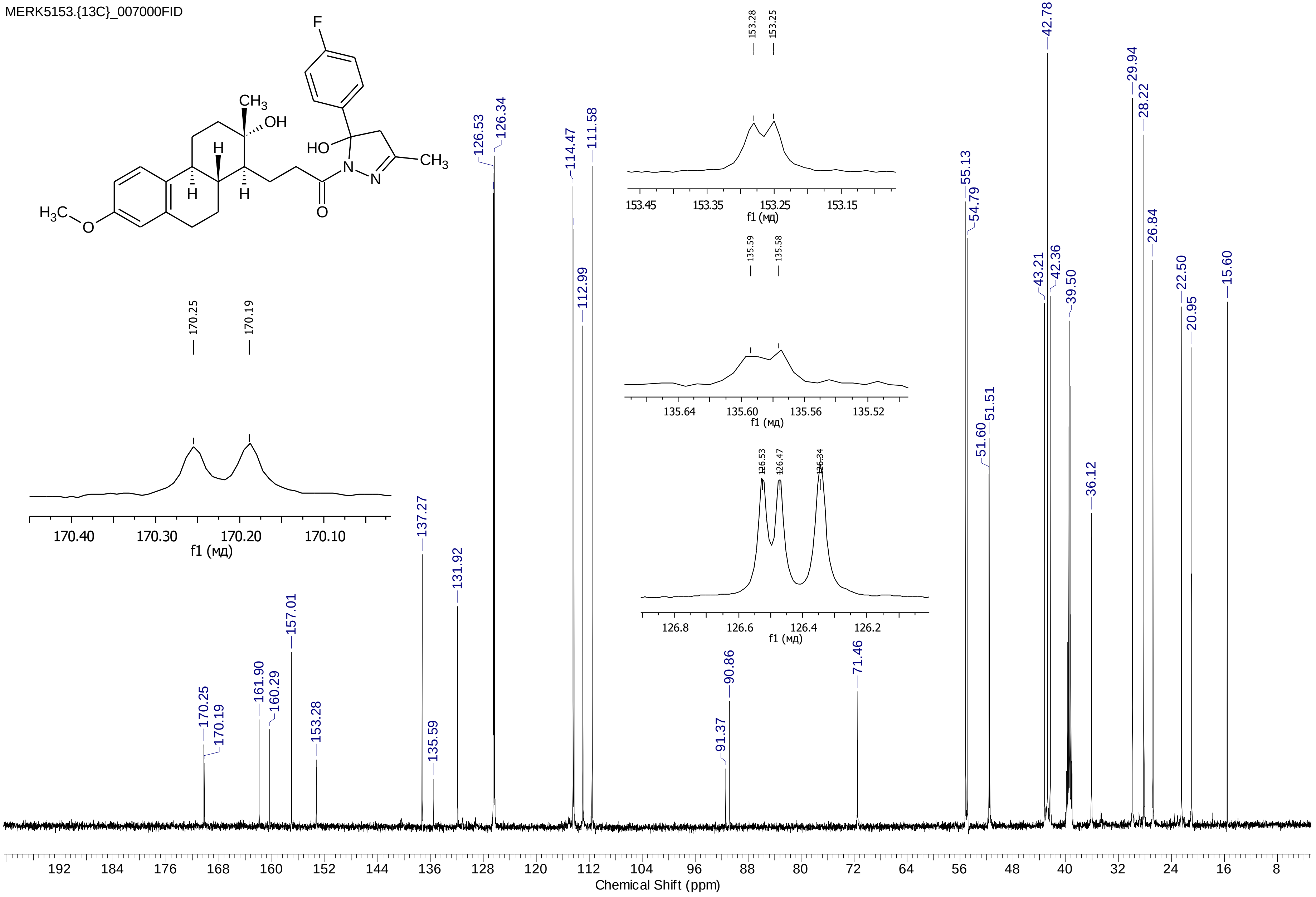

^{13}C NMR (DMSO-d_6, 150 MHz) spectrum of 5-(4-Fluorophenyl)-1-{3-[(1*S*,2*S*,4a*S*,10a*R*)-2-hydroxy-7-methoxy-2-methyl-1,2,3,4,4a,9,10,10a-octahydrophenanthren-1-yl]propanoyl}-3-methyl-4,5-dihydro-1*H*-pyrazol-5-ol (**3r**)

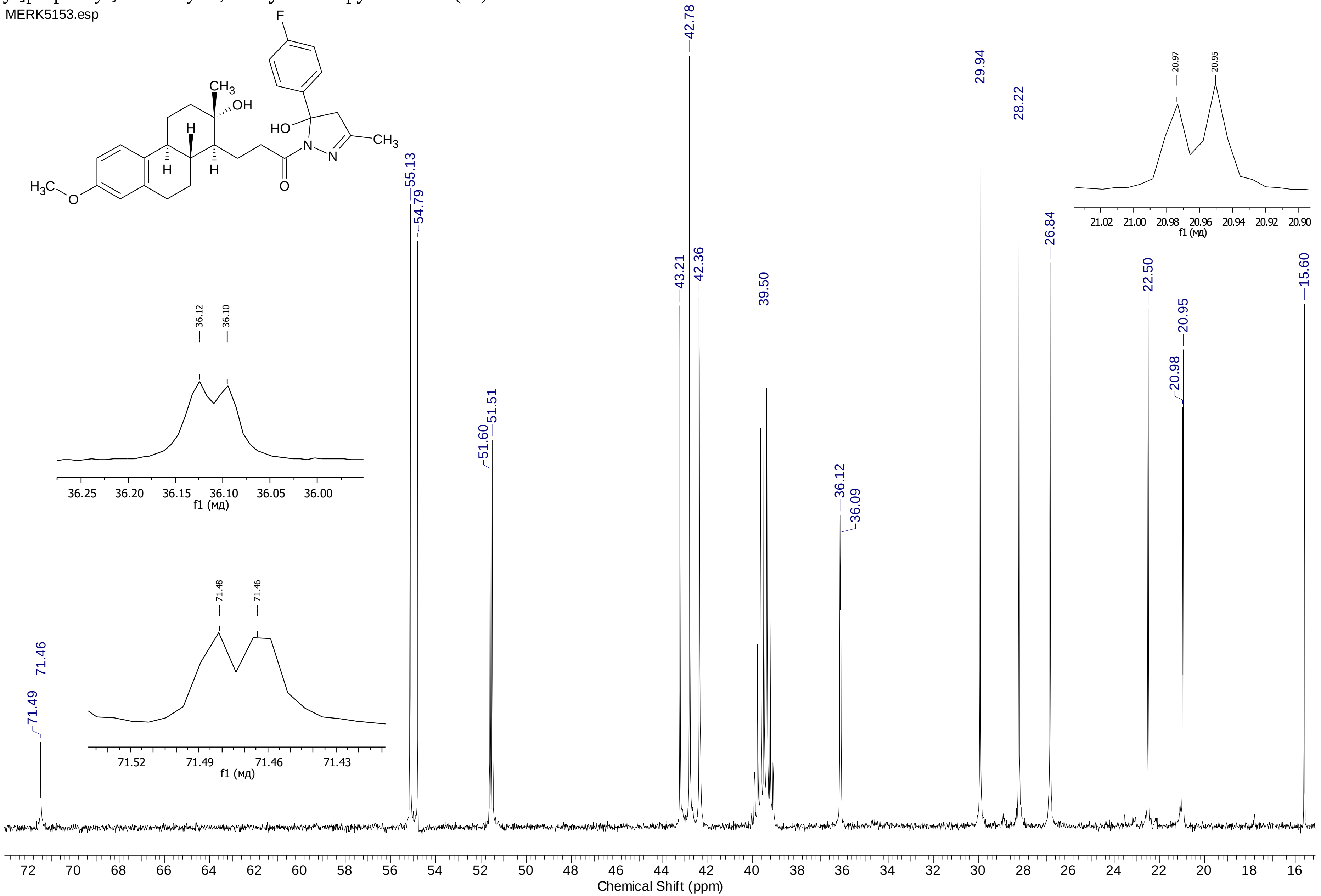

^{19}F NMR (DMSO-d_6, 564 MHz) spectrum of 5-(4-Fluorophenyl)-1-{3-[(1*S*,2*S*,4a*S*,10a*R*)-2-hydroxy-7-methoxy-2-methyl-1,2,3,4,4a,9,10,10a-octahydrophenanthren-1-yl]propanoyl}-3-methyl-4,5-dihydro-1*H*-pyrazol-5-ol (**3r**)

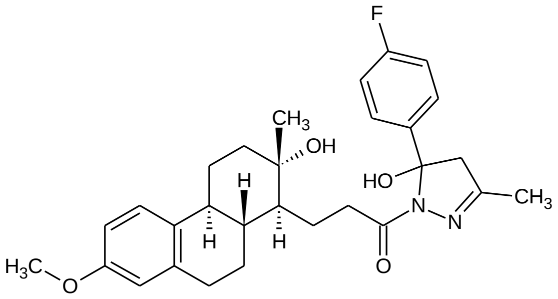

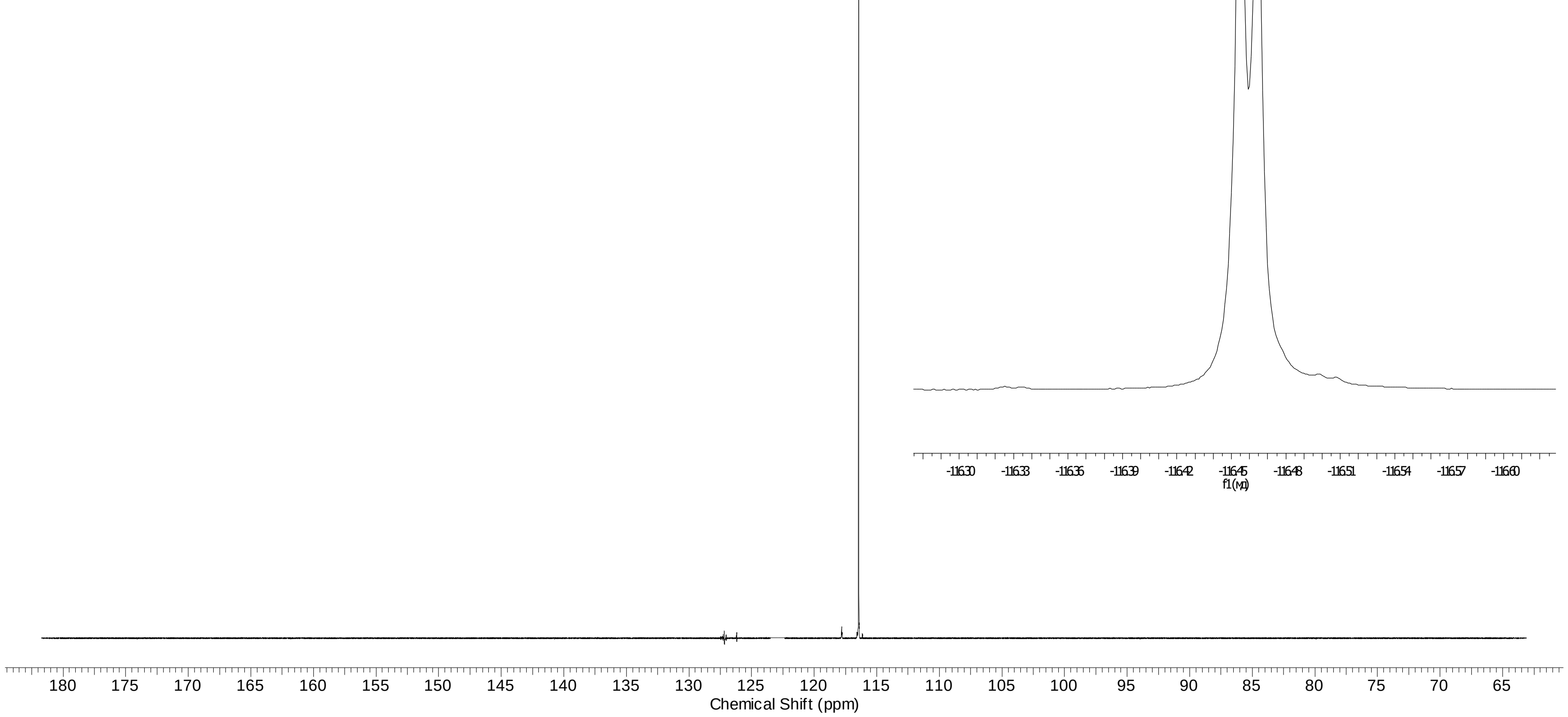

^{1}H – ^{1}H COSY (DMSO-d$_6$, 600 MHz) spectrum of 5-(4-Fluorophenyl)-1-{3-[(1*S*,2*S*,4a*S*,10a*R*)-2-hydroxy-7-methoxy-2-methyl-1,2,3,4,4a,9,10,10a-octahydrophenanthren-1-yl]propanoyl}-3-methyl-4,5-dihydro-1*H*-pyrazol-5-ol (**3r**)

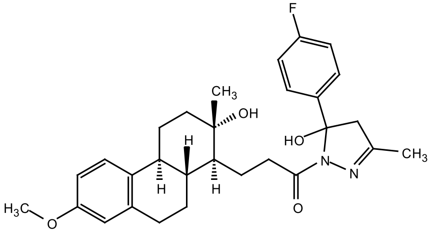

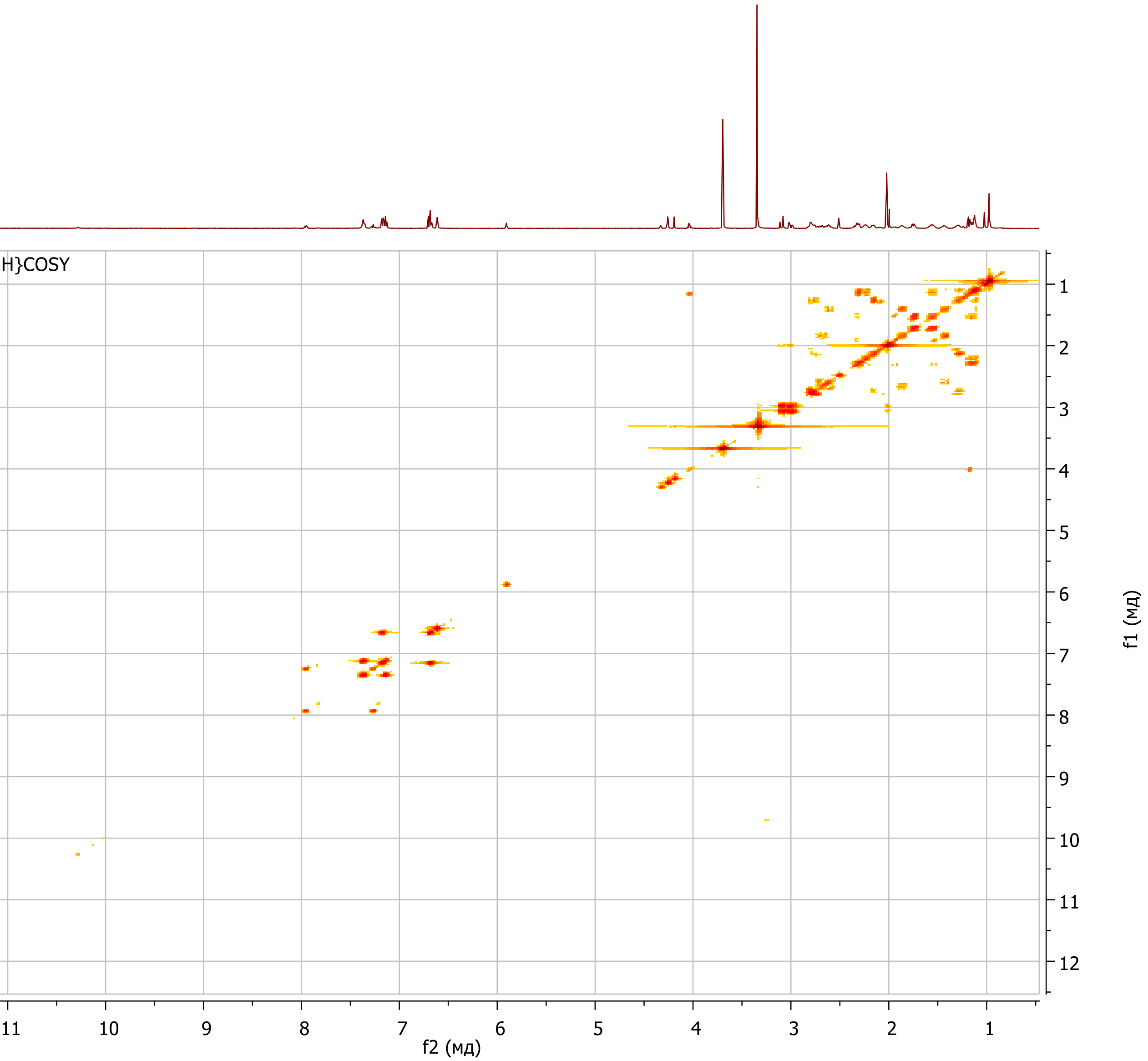

^{1}H – ^{13}C HSQC (DMSO-d_6, 600 MHz) spectrum of 5-(4-Fluorophenyl)-1-{3-[(1*S*,2*S*,4a*S*,10a*R*)-2-hydroxy-7-methoxy-2-methyl-1,2,3,4,4a,9,10,10a-octahydrophenanthren-1-yl]propanoyl}-3-methyl-4,5-dihydro-1*H*-pyrazol-5-ol (**3r**)

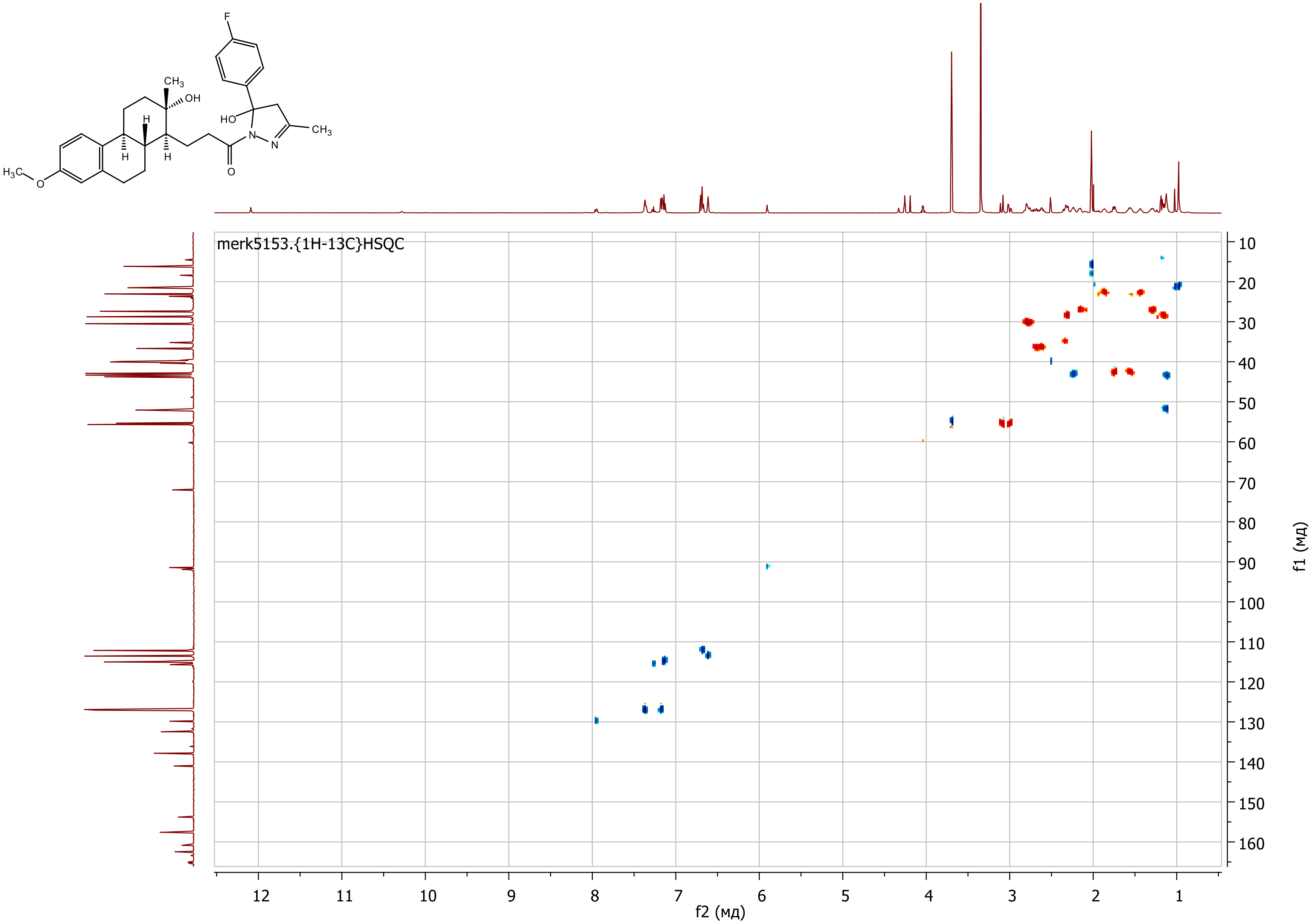

^{1}H – ^{13}C HMBC (DMSO-d_6, 600 MHz) spectrum of 5-(4-Fluorophenyl)-1-{3-[(1*S*,2*S*,4a*S*,10a*R*)-2-hydroxy-7-methoxy-2-methyl-1,2,3,4,4a,9,10,10a-octahydrophenanthren-1-yl]propanoyl}-3-methyl-4,5-dihydro-1*H*-pyrazol-5-ol (**3r**)

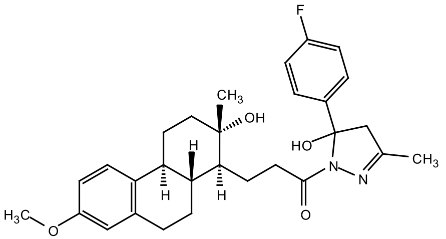

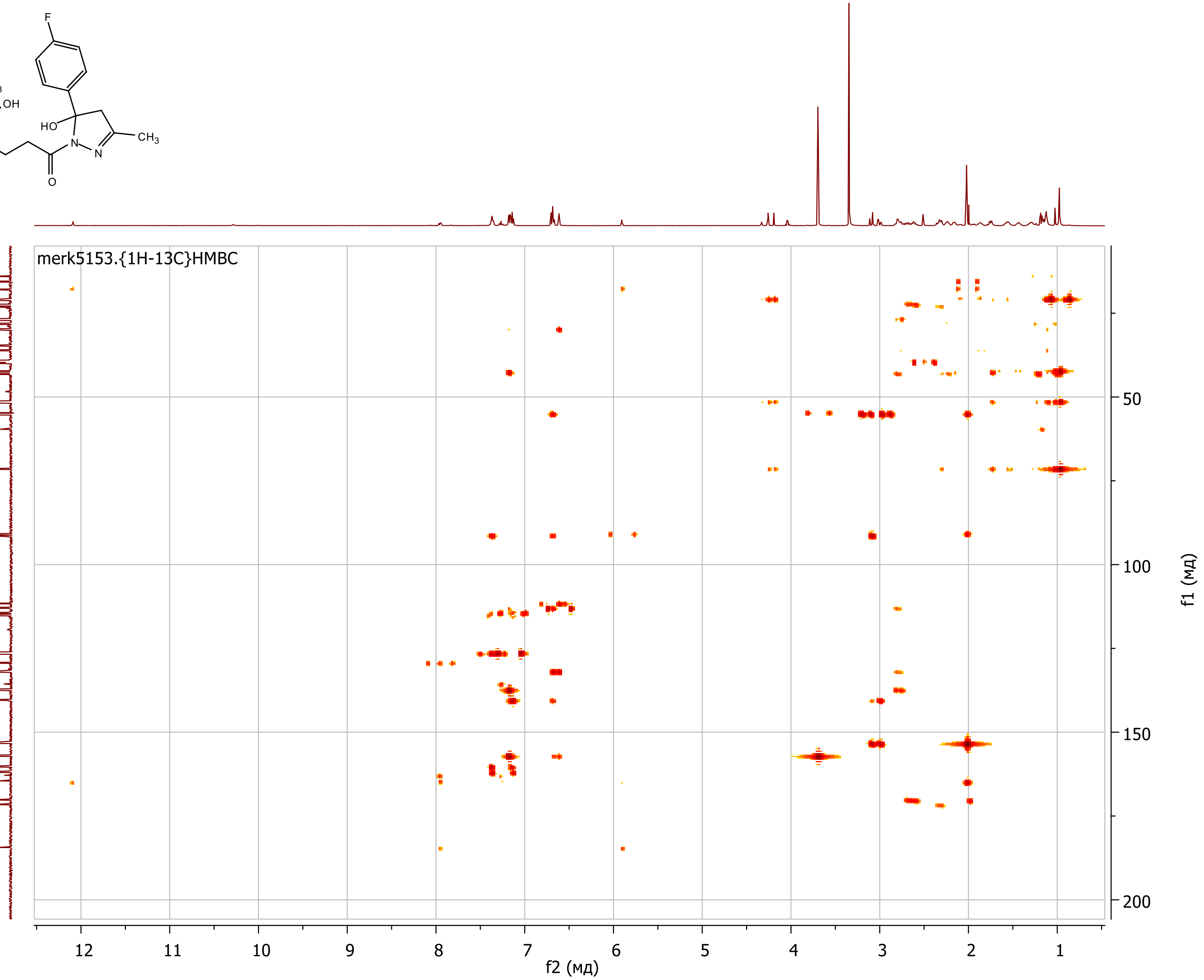

^{1}H NMR (DMSO-d$_6$, 500 MHz) of 5-(4-Bromophenyl)-1-{3-[(1*S*,2*S*,4a*S*,10a*R*)-2-hydroxy-7-methoxy-2-methyl-1,2,3,4,4a,9,10,10a-octahydrophenanthren-1-yl]propanoyl}-3-methyl-4,5-dihydro-1*H*-pyrazol-5-ol (**3s**)

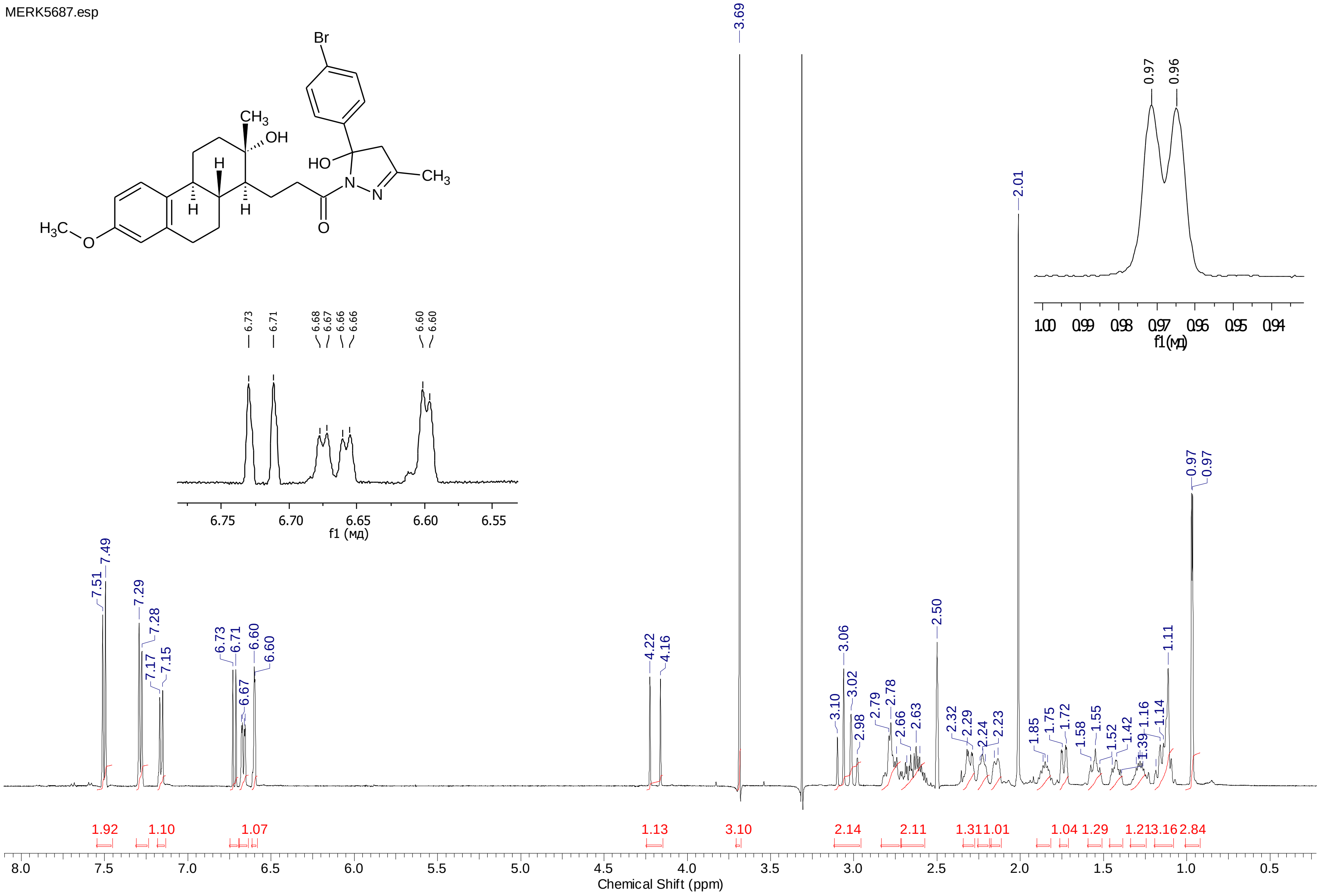

^{13}C NMR (DMSO-d$_6$, 125 MHz) of 5-(4-Bromophenyl)-1-{3-[(1*S*,2*S*,4a*S*,10a*R*)-2-hydroxy-7-methoxy-2-methyl-1,2,3,4,4a,9,10,10a-octahydrophenanthren-1-yl]propanoyl}-3-methyl-4,5-dihydro-1*H*-pyrazol-5-ol (**3s**)

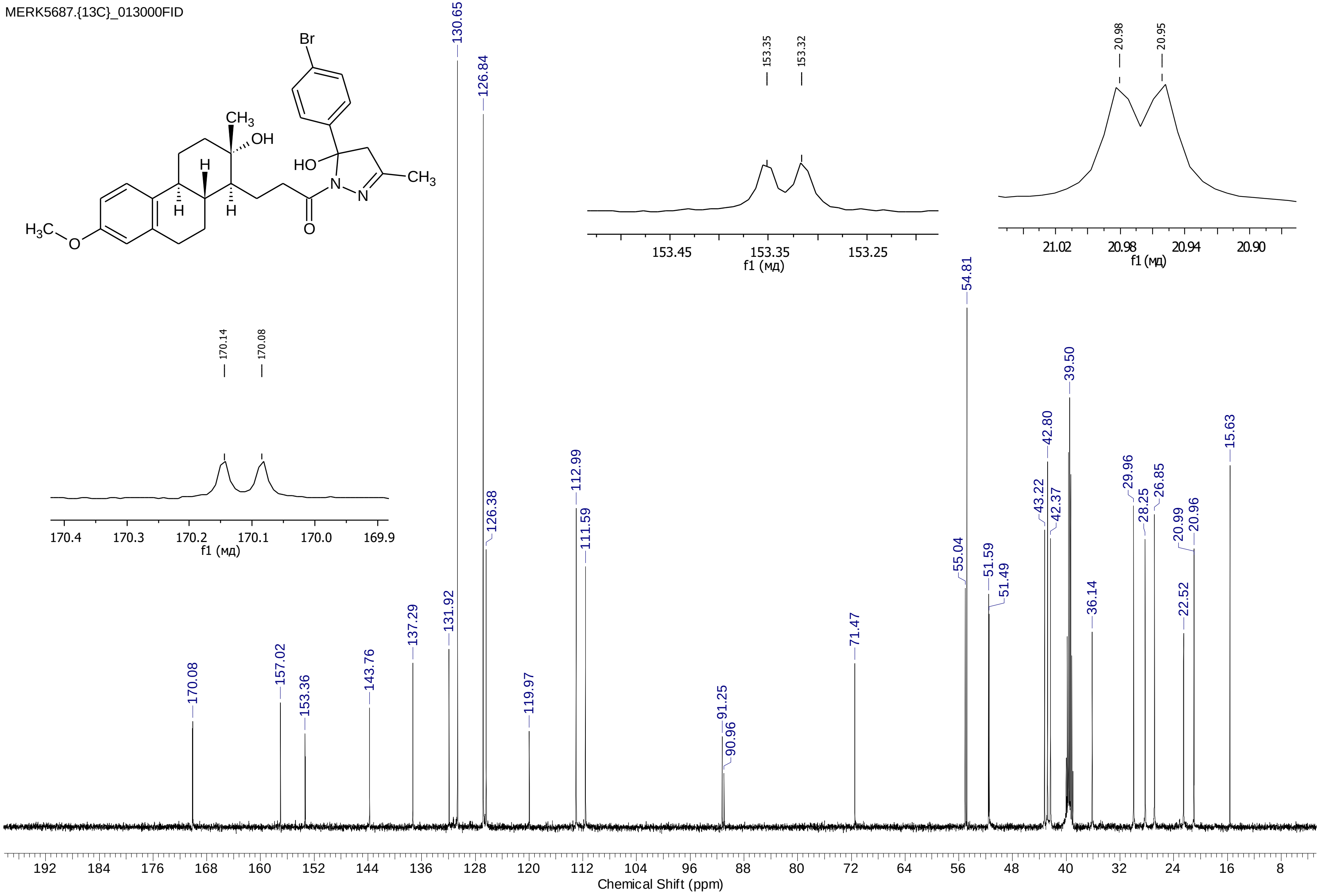

^{1}H NMR (DMSO-d6, 600 MHz) of 1-{3-[(1*S*,2*S*,4a*S*,10a*R*)-2-Hydroxy-7-methoxy-2-methyl-1,2,3,4,4a,9,10,10a-octahydrophenanthren-1-yl]propanoyl}-3-methyl-5-(trifluoromethyl)-4,5-dihydro-1*H*-pyrazol-5-ol (**3t**)

MERK5096.{1H}_001000FID

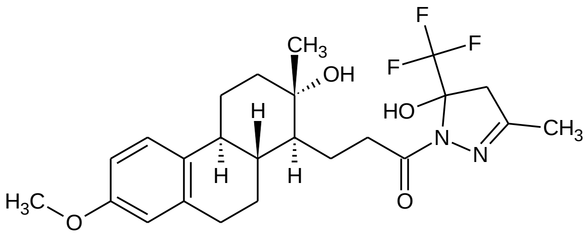

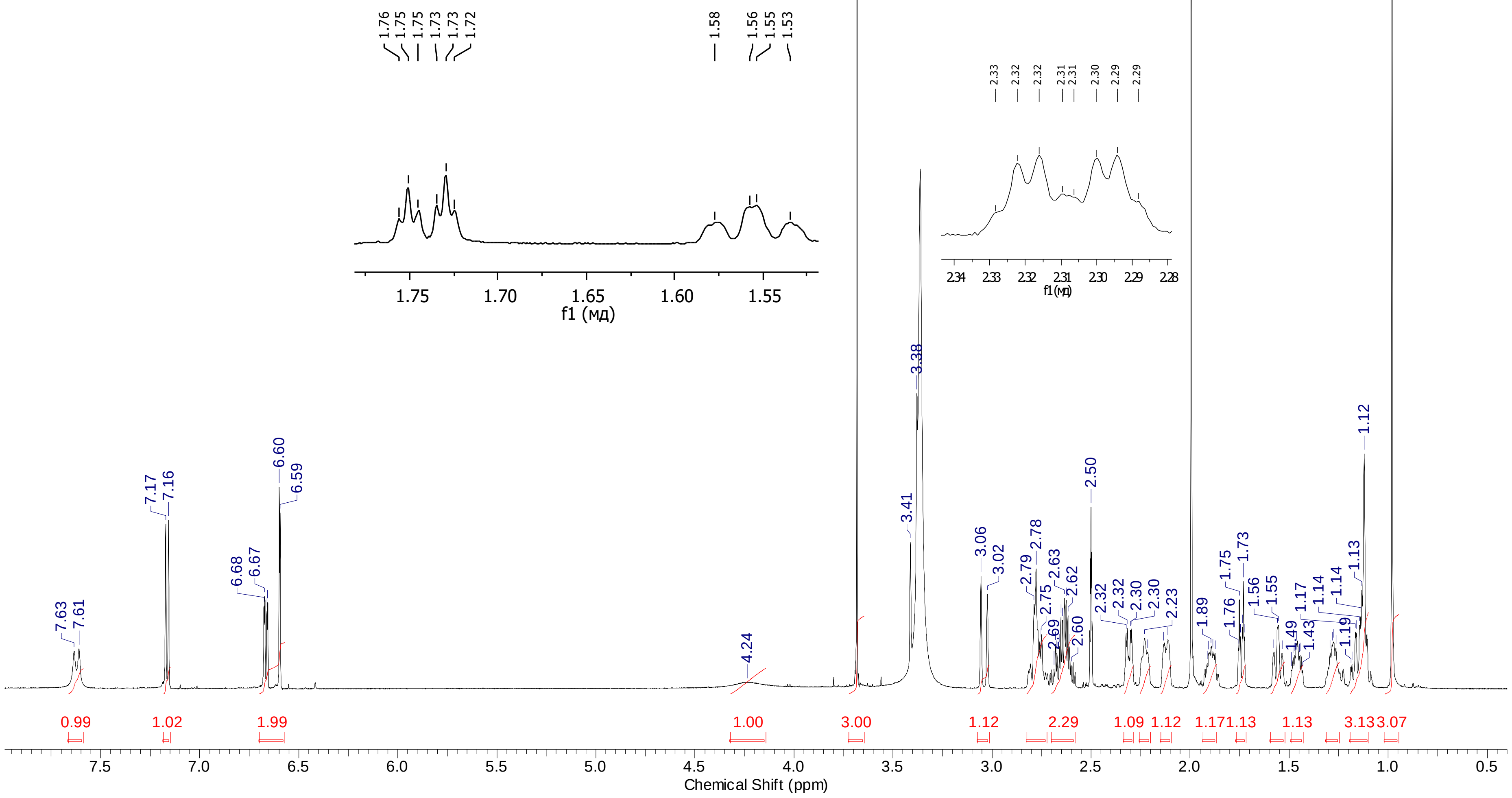

^{13}C NMR (DMSO-d_6, 150 MHz) spectrum of 1-{3-[(1*S*,2*S*,4a*S*,10a*R*)-2-Hydroxy-7-methoxy-2-methyl-1,2,3,4,4a,9,10,10a-octahydrophenanthren-1-yl]propanoyl}-3-methyl-5-(trifluoromethyl)-4,5-dihydro-1*H*-pyrazol-5-ol (**3t**)

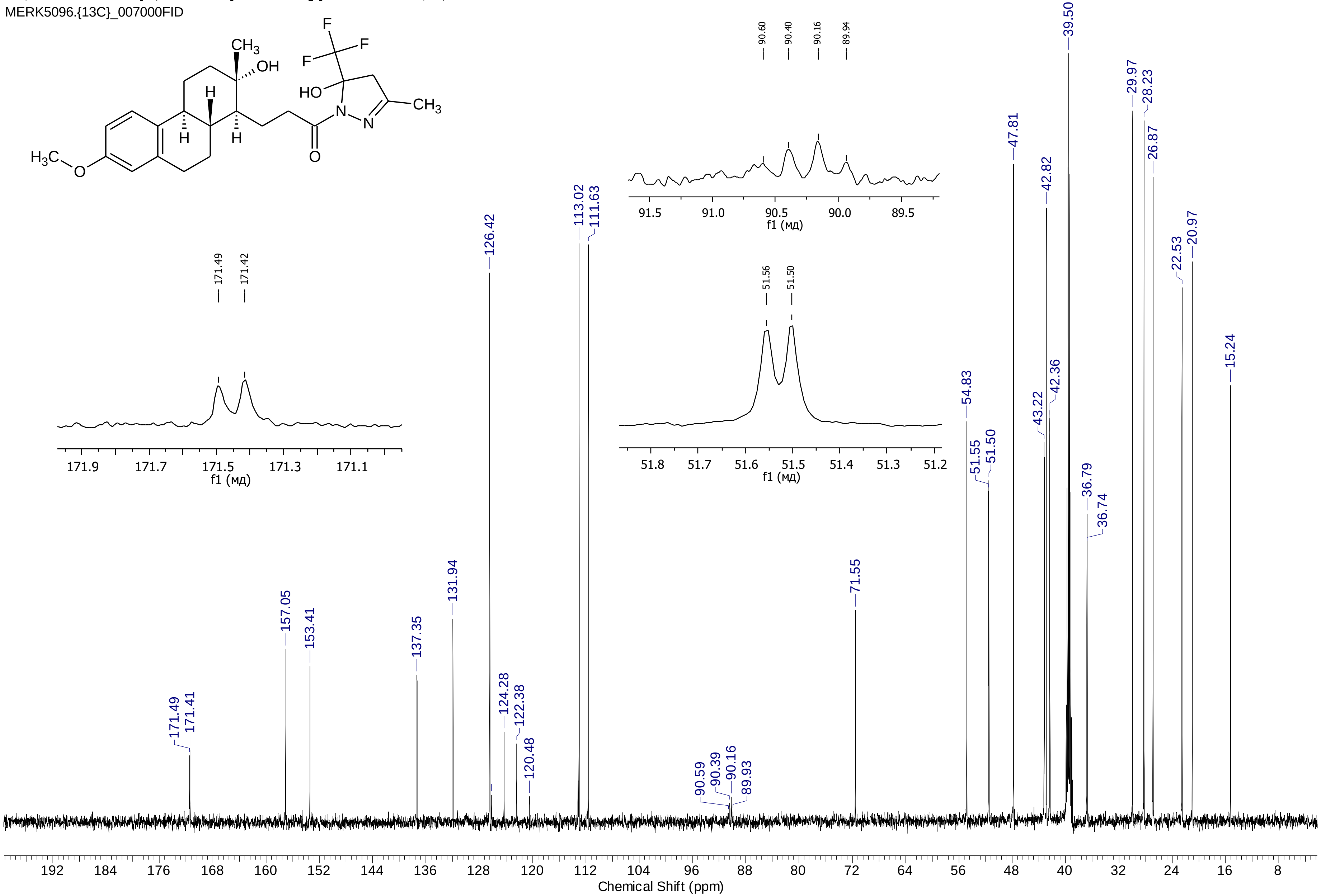

^{13}C NMR (DMSO-d_6, 150 MHz) spectrum of 1-{3-[(1*S*,2*S*,4a*S*,10a*R*)-2-Hydroxy-7-methoxy-2-methyl-1,2,3,4,4a,9,10,10a-octahydrophenanthren-1-yl]propanoyl}-3-methyl-5-(trifluoromethyl)-4,5-dihydro-1*H*-pyrazol-5-ol (**3t**)

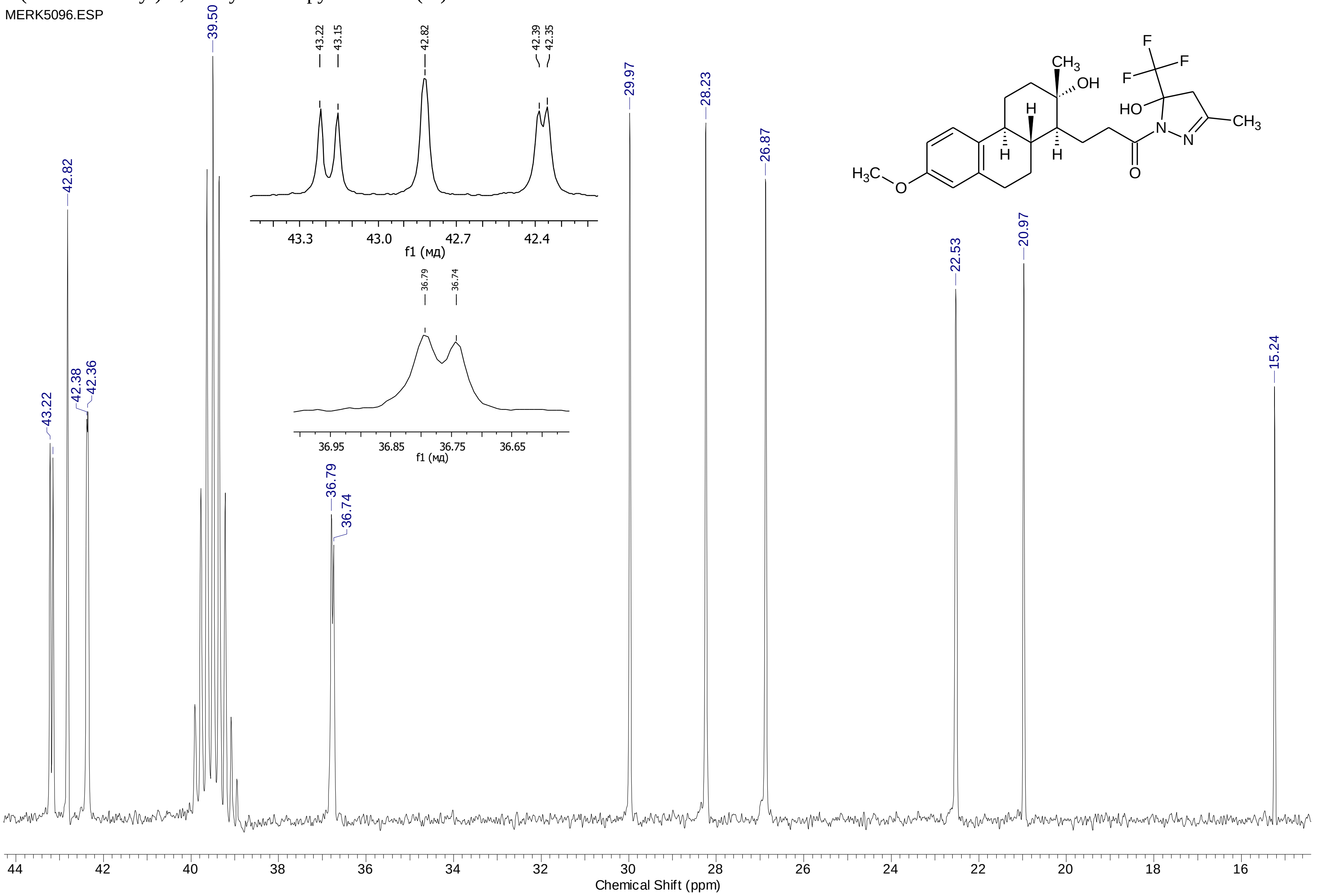

^{1}H – ^{1}H COSY (DMSO-d$_6$, 600 MHz) spectrum of 1-{3-[(1*S*,2*S*,4a*S*,10a*R*)-2-Hydroxy-7-methoxy-2-methyl-1,2,3,4,4a,9,10,10a-octahydrophenanthren-1-yl]propanoyl}-3-methyl-5-(trifluoromethyl)-4,5-dihydro-1*H*-pyrazol-5-ol (**3t**)

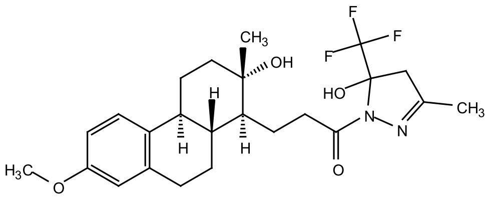

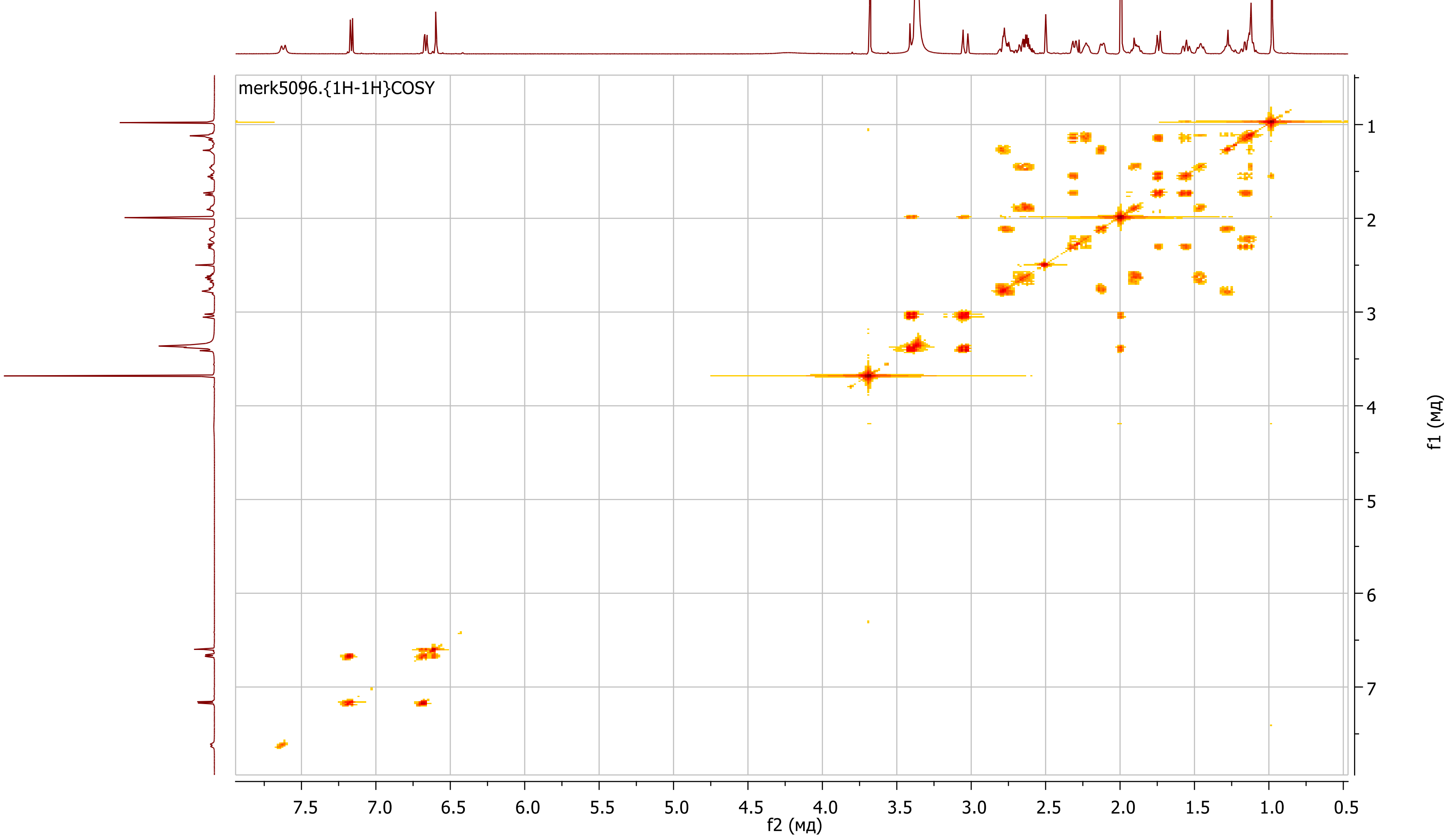

^{1}H – ^{13}C HSQC (DMSO-d_6, 600 MHz) spectrum of 1-{3-[(1*S*,2*S*,4a*S*,10a*R*)-2-Hydroxy-7-methoxy-2-methyl-1,2,3,4,4a,9,10,10a-octahydrophenanthren-1-yl]propanoyl}-3-methyl-5-(trifluoromethyl)-4,5-dihydro-1*H*-pyrazol-5-ol (**3t**)

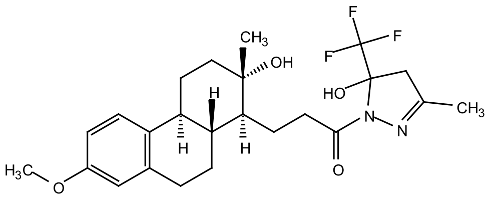

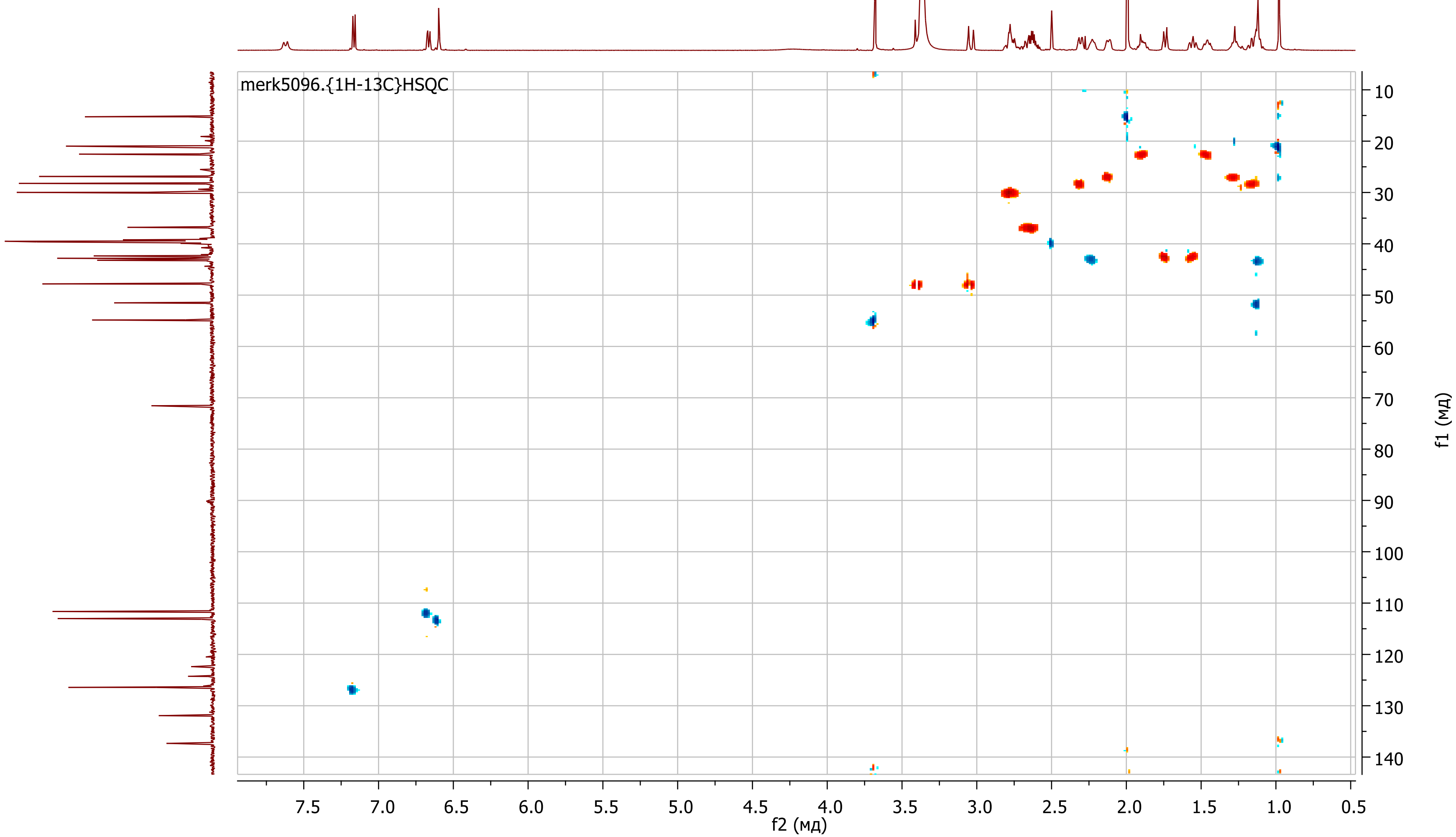

^{1}H – ^{13}C HMBC (DMSO-d_6, 600 MHz) spectrum of 1-{3-[(1*S*,2*S*,4a*S*,10a*R*)-2-Hydroxy-7-methoxy-2-methyl-1,2,3,4,4a,9,10,10a-octahydrophenanthren-1-yl]propanoyl}-3-methyl-5-(trifluoromethyl)-4,5-dihydro-1*H*-pyrazol-5-ol (**3t**)

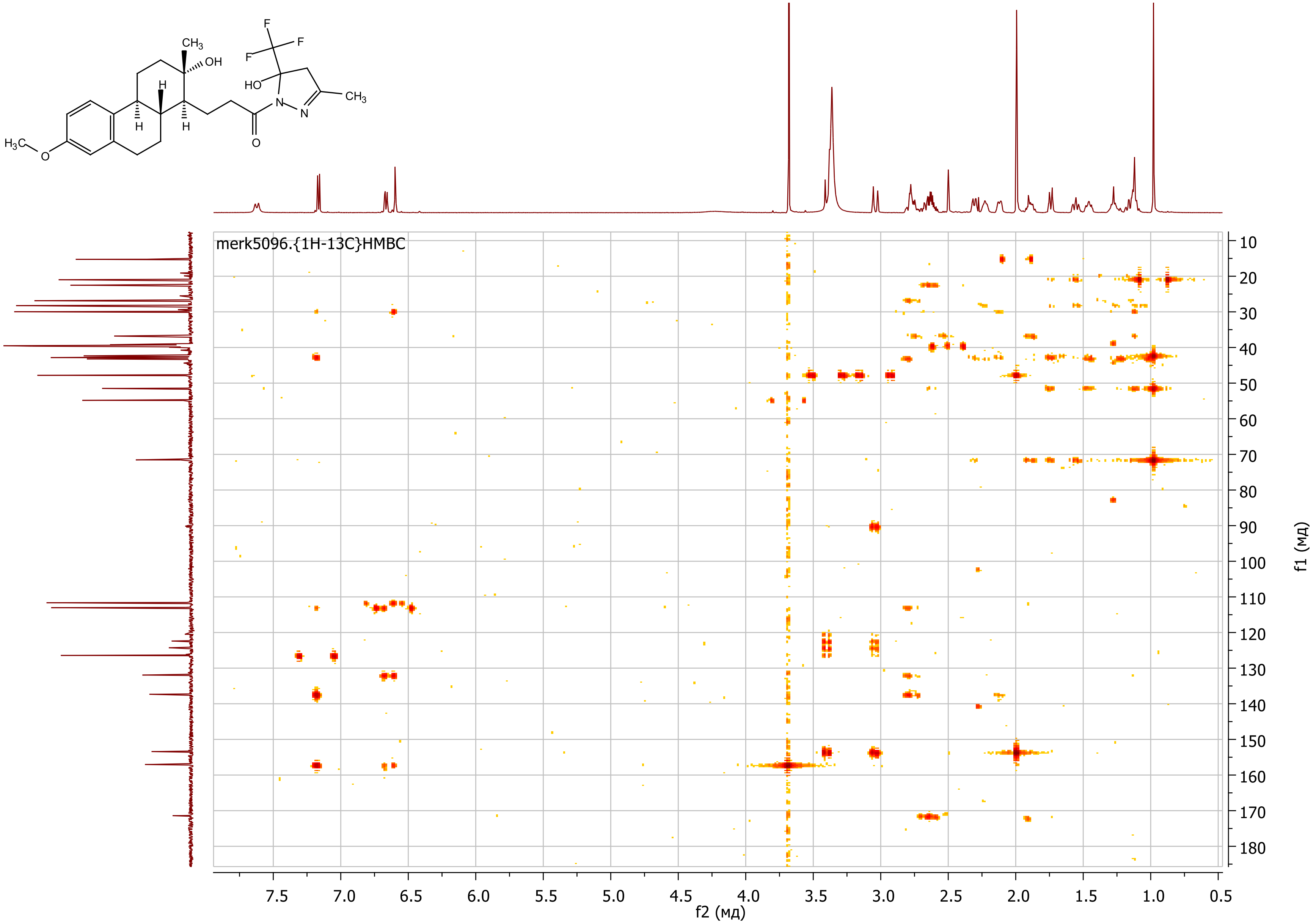

HRMS spectrum of 1-{3-[(1*S*,2*S*,4a*S*,10a*R*)-2-hydroxy-7-methoxy-2-methyl-1,2,3,4,4a,9,10,10a-octahydrophenanthren-1-yl]propanoyl}-3,5-dimethyl-4,5-dihydro-1*H*-pyrazol-5-ol **(3a)**

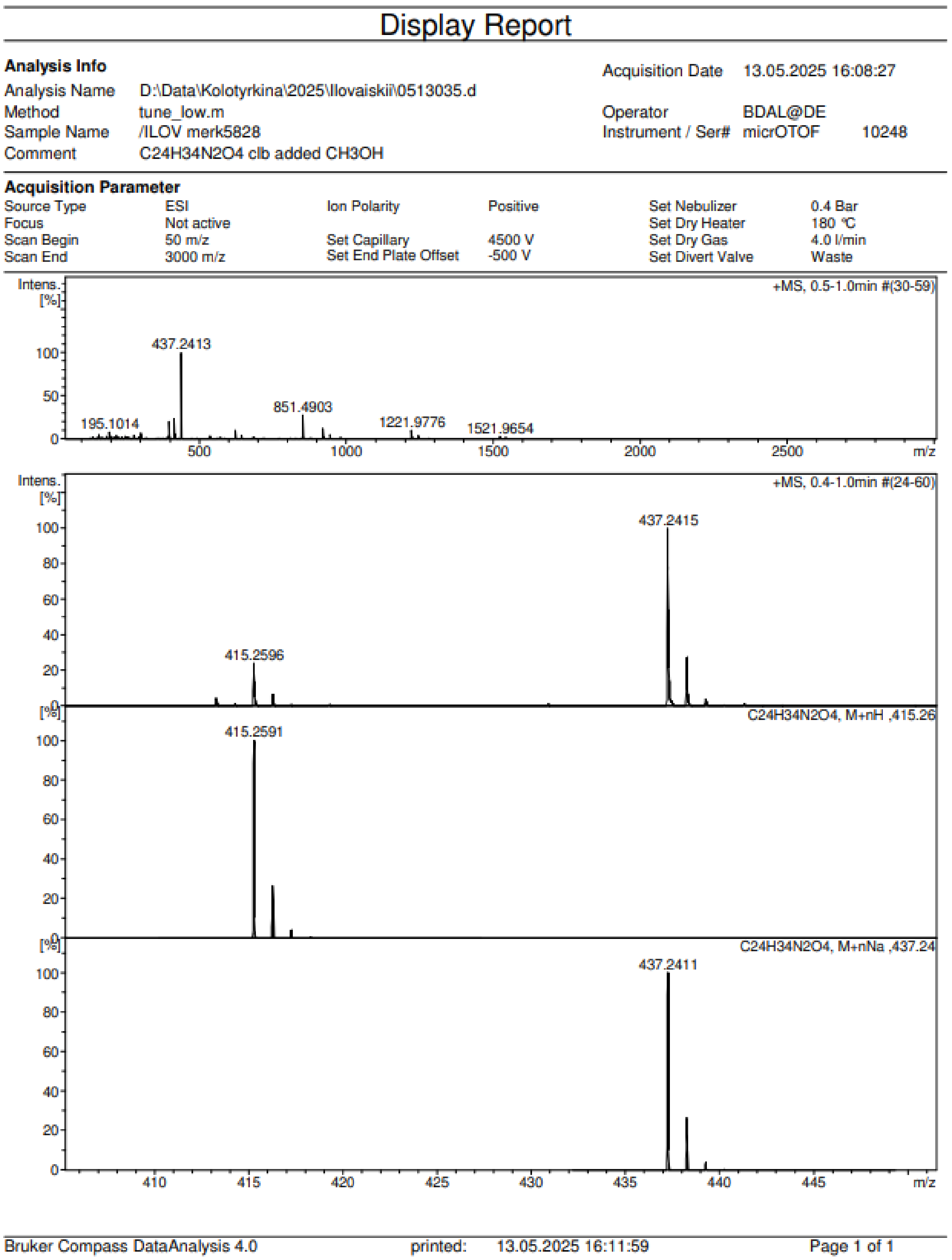

HRMS spectrum of 1-{3-[(1*S*,2*S*,4a*S*,10a*R*)-2-hydroxy-7-methoxy-2-methyl-1,2,3,4,4a,9,10,10a-octahydrophenanthren-1-yl]propanoyl}-3,4,5-trimethyl-4,5-dihydro-1*H*-pyrazol-5-ol **(3b)**

Display Report

Analysis Info

Analysis Name	D:\Data\Kolotyrkina\2024\Ilovaiskii\0111026.d	Acquisition Date	11.01.2024 17:23:45
Method	tune_low.m	Operator	BDAL@DE
Sample Name	/ILOV Merk5218	Instrument / Ser#	micrOTOF 10248
Comment	C25H36N2O4 mH429.2758 calibrant added CH3CN		

Acquisition Parameter

Source Type	ESI	Ion Polarity	Positive	Set Nebulizer	0.4 Bar
Focus	Not active			Set Dry Heater	180 °C
Scan Begin	50 m/z	Set Capillary	4500 V	Set Dry Gas	4.0 l/min
Scan End	3000 m/z	Set End Plate Offset	-500 V	Set Divert Valve	Waste

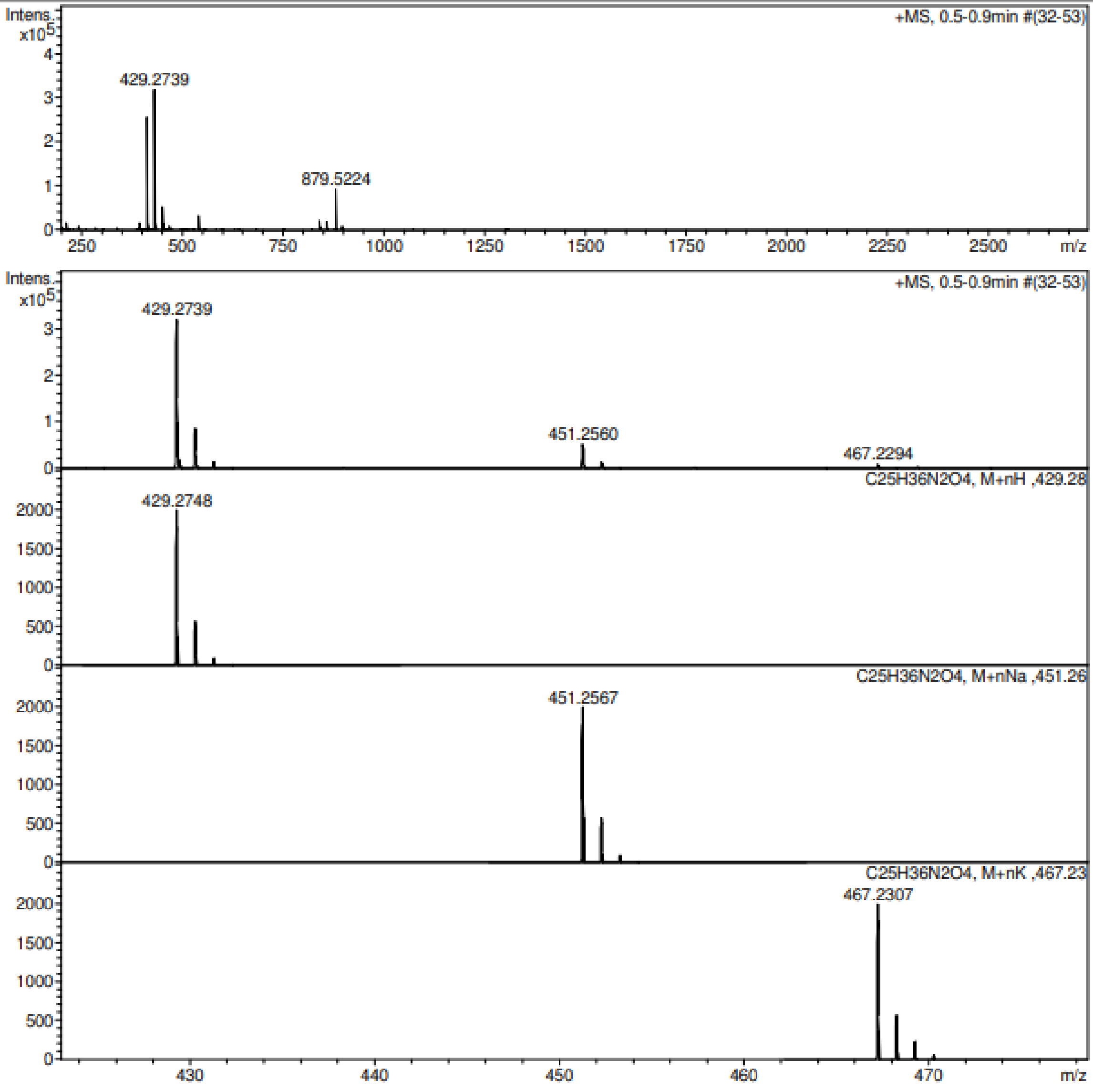

Bruker Compass DataAnalysis 4.0 printed: 11.01.2024 17:26:59 Page 1 of 1

HRMS spectrum of 1-{3-[(1*S*,2*S*,4a*S*,10a*R*)-2-hydroxy-7-methoxy-2-methyl-1,2,3,4,4a,9,10,10a-octahydrophenanthren-1-yl]propanoyl}-3,4,4,5-tetramethyl-4,5-dihydro-1*H*-pyrazol-5-ol **(3c)**

Display Report

Analysis Info

Analysis Name	D:\Data\Kolotyrkina\2024\Ilovaiskii\0208036.d	Acquisition Date	08.02.2024 17:29:50
Method	tune_low.m	Operator	BDAL@DE
Sample Name	/ILOV MERK5222	Instrument / Ser#	micrOTOF 10248
Comment	C26H38N2O4 mH443.2904 calibrant added CH3OH		

Acquisition Parameter

Source Type	ESI	Ion Polarity	Positive	Set Nebulizer	0.4 Bar
Focus	Not active			Set Dry Heater	180 °C
Scan Begin	50 m/z	Set Capillary	4500 V	Set Dry Gas	4.0 l/min
Scan End	3000 m/z	Set End Plate Offset	-500 V	Set Divert Valve	Waste

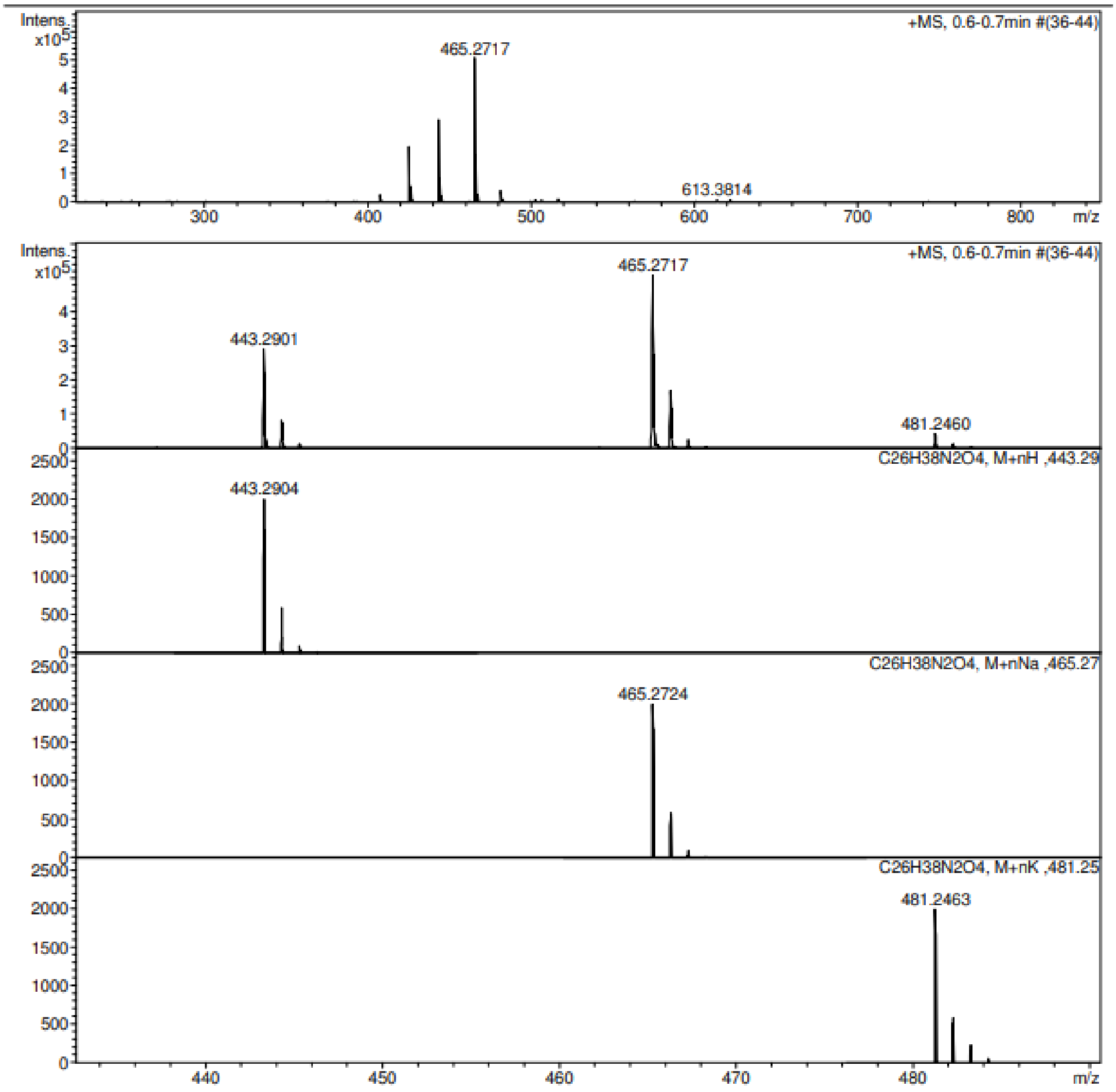

Bruker Compass DataAnalysis 4.0 printed: 08.02.2024 17:32:45 Page 1 of 1

HRMS spectrum of 4-Ethyl-1-{3-[(1*S*,2*S*,4a*S*,10a*R*)-2-hydroxy-7-methoxy-2-methyl-1,2,3,4,4a,9,10,10a-octahydrophenanthren-1-yl]propanoyl}-3,5-dimethyl-4,5-dihydro-1*H*-pyrazol-5-ol **(3d)**

Display Report

Analysis Info

Analysis Name	D:\Data\Kolotyrkina\2024\Ilovaiskii\0124015.d	Acquisition Date	24.01.2024 12:32:17
Method	tune_low.m	Operator	BDAL@DE
Sample Name	/ILOV MERK5252	Instrument / Ser#	micrOTOF 10248
Comment	C26H38N2O4 mH443.2904 calibrant added CH3CN		

Acquisition Parameter

Source Type	ESI	Ion Polarity	Positive	Set Nebulizer	0.4 Bar
Focus	Not active			Set Dry Heater	180 °C
Scan Begin	50 m/z	Set Capillary	4500 V	Set Dry Gas	4.0 l/min
Scan End	3000 m/z	Set End Plate Offset	-500 V	Set Divert Valve	Waste

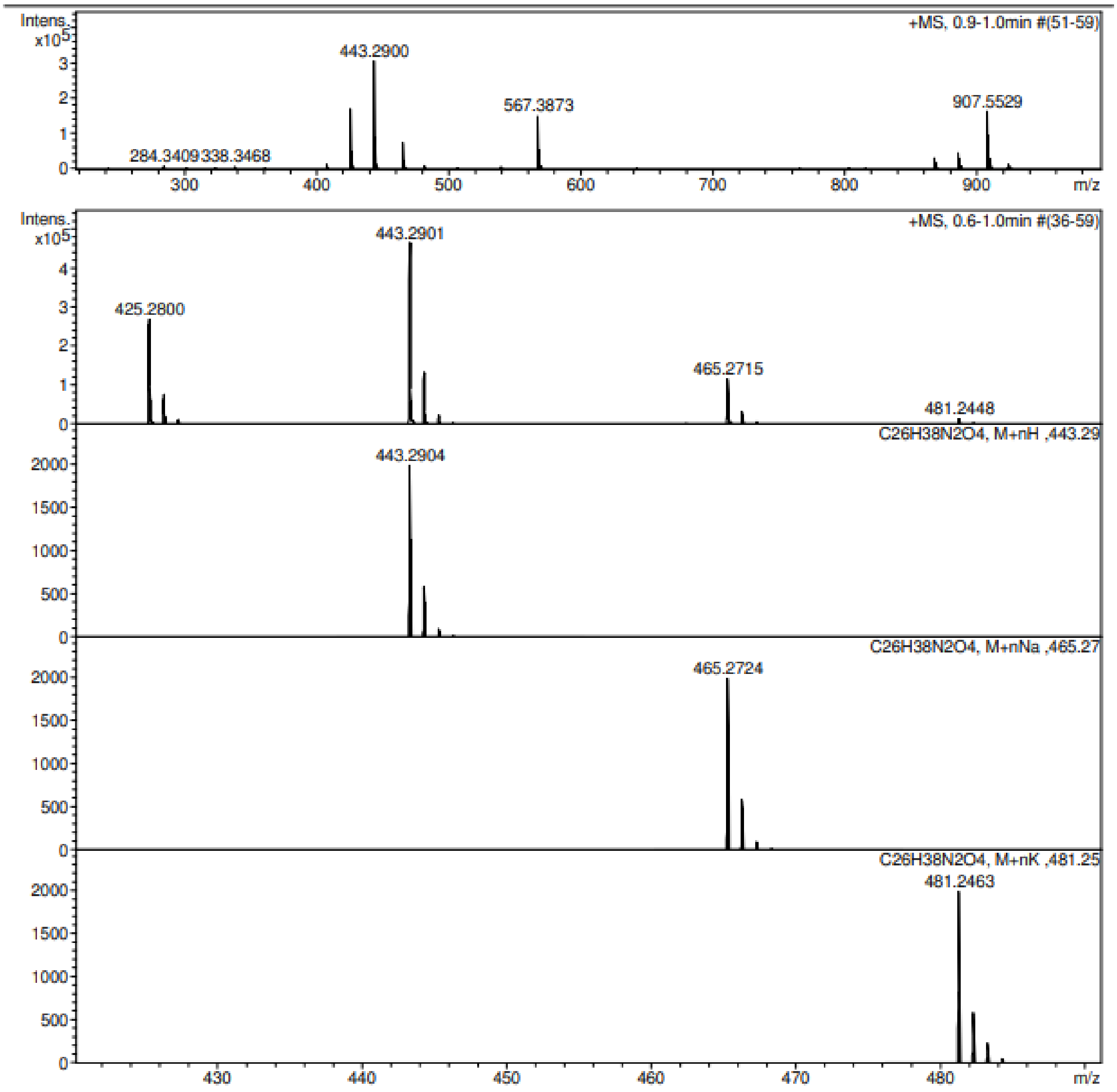

Bruker Compass DataAnalysis 4.0 printed: 24.01.2024 12:40:27 Page 1 of 1

HRMS spectrum of 4-Butyl-1-{3-[(1*S*,2*S*,4a*S*,10a*R*)-2-hydroxy-7-methoxy-2-methyl-1,2,3,4,4a,9,10,10a-octahydrophenanthren-1-yl]propanoyl}-3,5-dimethyl-4,5-dihydro-1*H*-pyrazol-5-ol **(3e)**

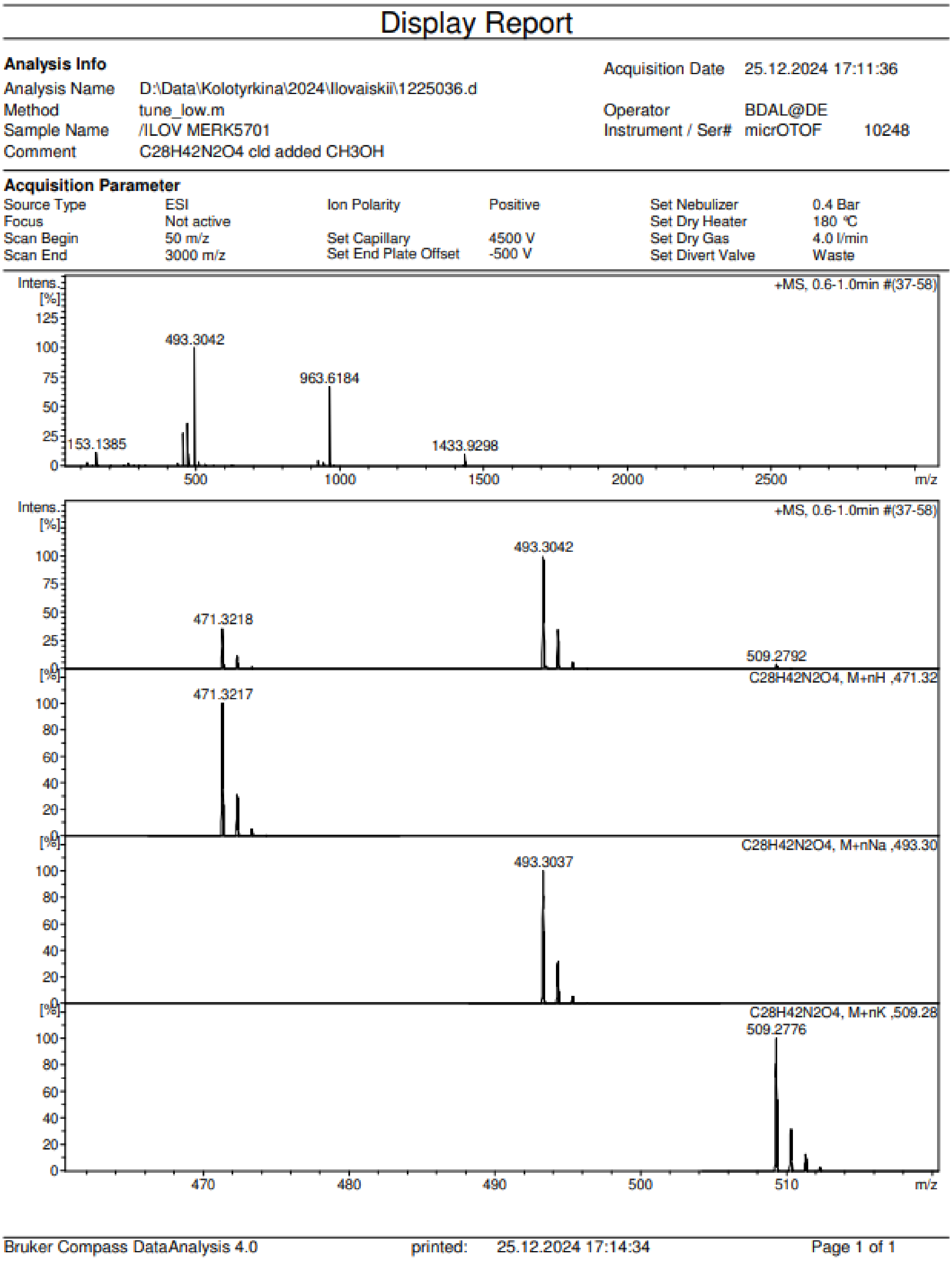

HRMS spectrum of 1-{3-[(1*S*,2*S*,4a*S*,10a*R*)-2-Hydroxy-7-methoxy-2-methyl-1,2,3,4,4a,9,10,10a-octahydrophenanthren-1-yl]propanoyl}-3,5-dimethyl-4-(3-methylbutyl)-4,5-dihydro-1*H*-pyrazol-5-ol **(3f)**

Display Report

Analysis Info

Analysis Name	D:\Data\Kolotyrkina\2024\Ilovaiskii\0125034.d	Acquisition Date	25.01.2024 17:03:35
Method	tune_low.m	Operator	BDAL@DE
Sample Name	/ILOV Merk5208	Instrument / Ser#	micrOTOF 10248
Comment	C29H44N2O4 mH485.3384 calibrant added CH3OH		

Acquisition Parameter

Source Type	ESI	Ion Polarity	Positive	Set Nebulizer	0.4 Bar
Focus	Not active			Set Dry Heater	180 °C
Scan Begin	50 m/z	Set Capillary	4500 V	Set Dry Gas	4.0 l/min
Scan End	3000 m/z	Set End Plate Offset	-500 V	Set Divert Valve	Waste

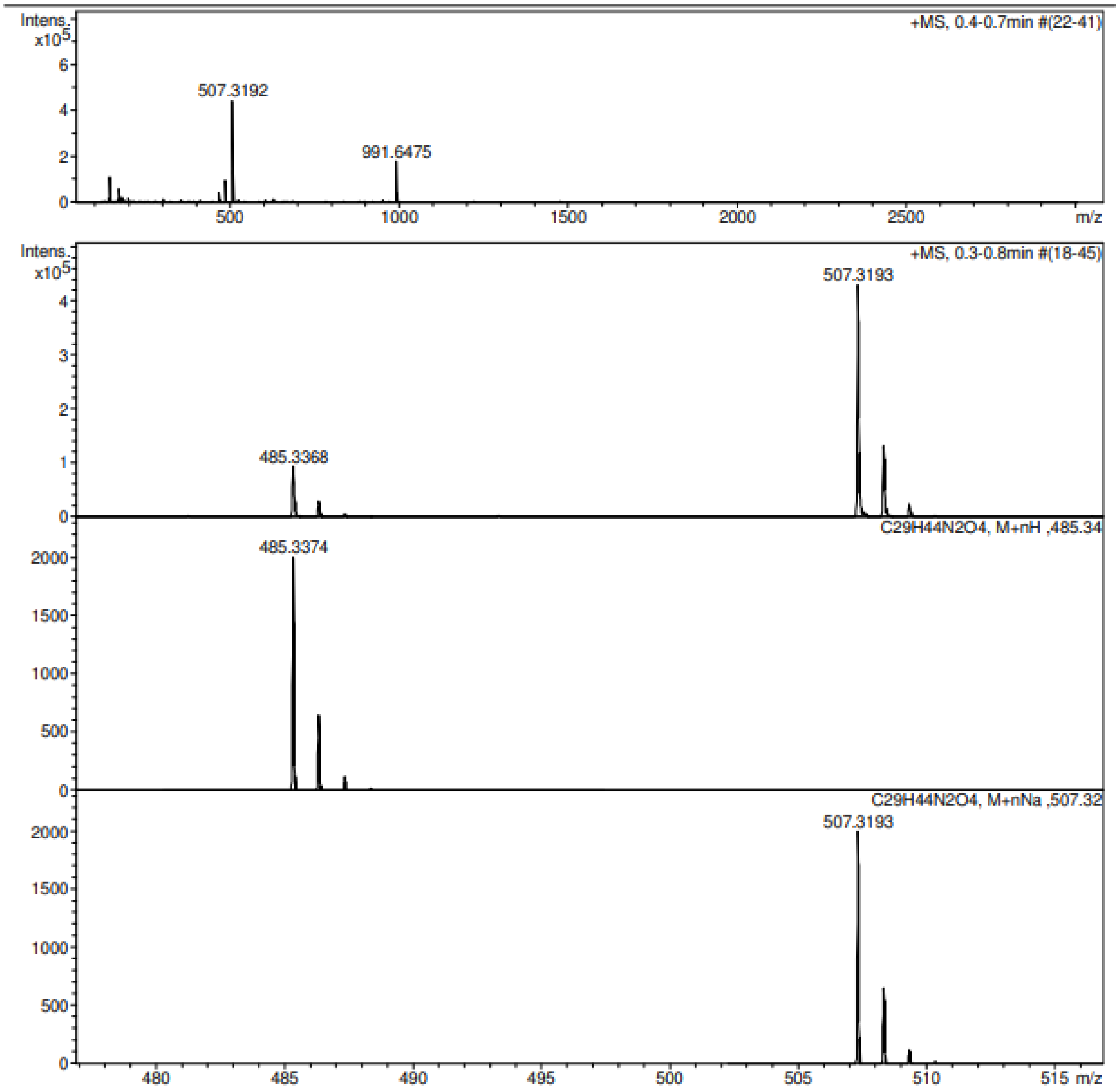

Bruker Compass DataAnalysis 4.0 printed: 25.01.2024 17:09:03 Page 1 of 1

HRMS spectrum of 4-Hexyl-1-{3-[(1*S*,2*S*,4a*S*,10a*R*)-2-hydroxy-7-methoxy-2-methyl-1,2,3,4,4a,9,10,10a-octahydrophenanthren-1-yl]propanoyl}-3,5-dimethyl-4,5-dihydro-1*H*-pyrazol-5-ol **(3g)**

Display Report

Analysis Info

Analysis Name	D:\Data\Kolotyrkina\2024\Ilovaiskii\1218018.d	Acquisition Date	18.12.2024 17:23:10
Method	tune_low.m	Operator	BDAL@DE
Sample Name	/ILOV MERK5667	Instrument / Ser#	micrOTOF 10248
Comment	C30H46N2O4 clb added CH3OH		

Acquisition Parameter

Source Type	ESI	Ion Polarity	Positive	Set Nebulizer	0.4 Bar
Focus	Not active			Set Dry Heater	180 °C
Scan Begin	50 m/z	Set Capillary	4500 V	Set Dry Gas	4.0 l/min
Scan End	3000 m/z	Set End Plate Offset	-500 V	Set Divert Valve	Waste

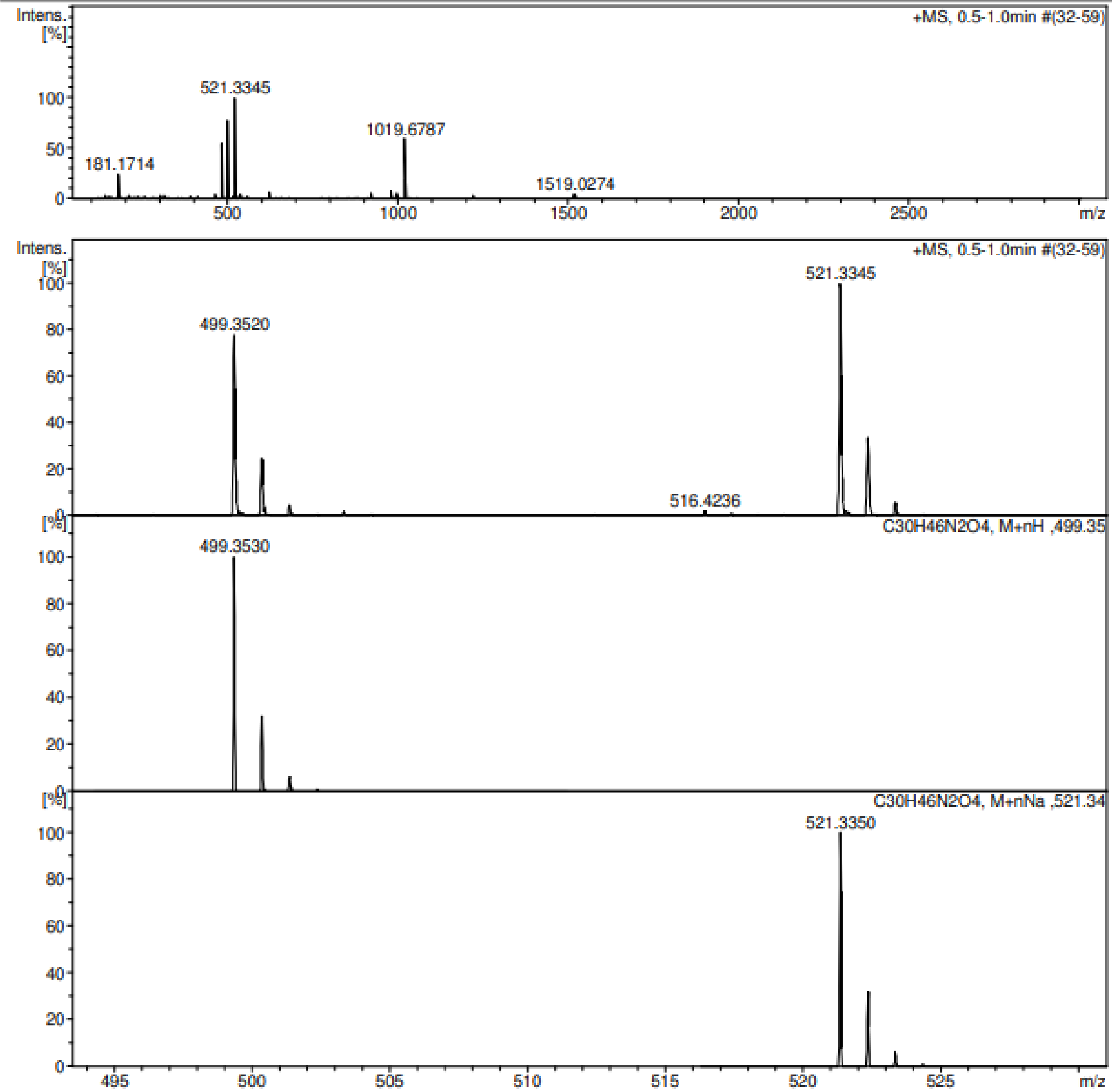

HRMS spectrum of 4-Allyl-1-{3-[(1*S*,2*S*,4a*S*,10a*R*)-2-hydroxy-7-methoxy-2-methyl-1,2,3,4,4a,9,10,10a-octahydrophenanthren-1-yl]propanoyl}-3,5-dimethyl-4,5-dihydro-1*H*-pyrazol-5-ol (**3h**)

Display Report

Analysis Info

Analysis Name	D:\Data\Kolotyrkina\2024\Ilovaiskii\1127028.d	Acquisition Date	27.11.2024 16:44:01
Method	tune_low.m	Operator	BDAL@DE
Sample Name	/ILOV MERK5664	Instrument / Ser#	micrOTOF 10248
Comment	C27H38N2O4 clb added CH3OH		

Acquisition Parameter

Source Type	ESI	Ion Polarity	Positive	Set Nebulizer	0.4 Bar
Focus	Not active			Set Dry Heater	180 °C
Scan Begin	50 m/z	Set Capillary	4500 V	Set Dry Gas	4.0 l/min
Scan End	3000 m/z	Set End Plate Offset	-500 V	Set Divert Valve	Waste

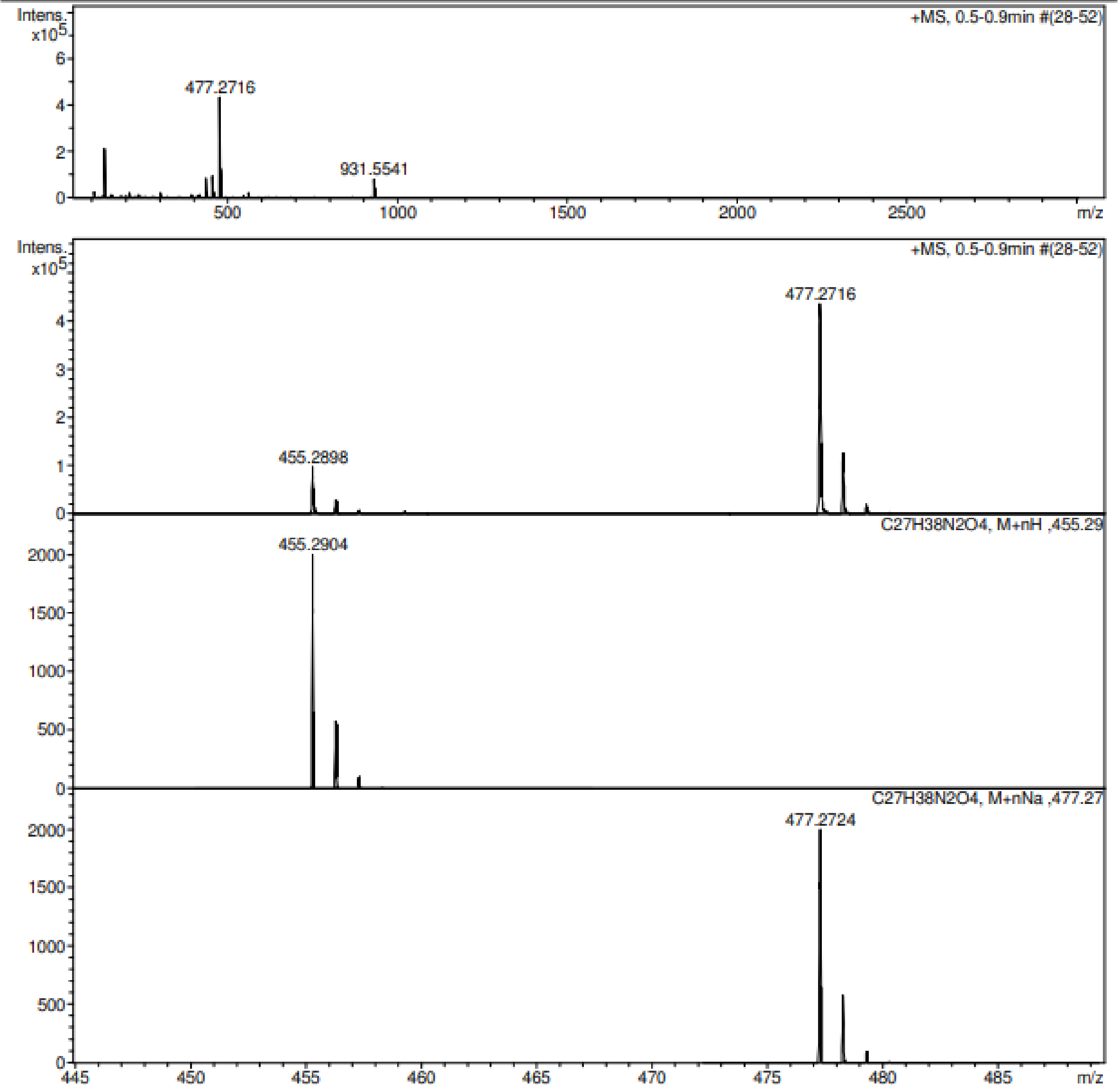

HRMS spectrum of 4-Benzyl-1-{3-[(1*S*,2*S*,4a*S*,10a*R*)-2-hydroxy-7-methoxy-2-methyl-1,2,3,4,4a,9,10,10a-octahydrophenanthren-1-yl]propanoyl}-3,5-dimethyl-4,5-dihydro-1*H*-pyrazol-5-ol **(3i)**

Display Report

Analysis Info

Analysis Name	D:\Data\Kolotyrkina\2023\Ilovaisky\1114027.d	Acquisition Date	14.11.2023 16:29:28
Method	tune_low.m	Operator	BDAL@DE
Sample Name	/ILOV Merk5112	Instrument / Ser#	micrOTOF 10248
Comment	C31H40N2O4 mH505.3060 calibrant added CH3OH		

Acquisition Parameter

Source Type	ESI	Ion Polarity	Positive	Set Nebulizer	0.4 Bar
Focus	Not active			Set Dry Heater	180 °C
Scan Begin	50 m/z	Set Capillary	4500 V	Set Dry Gas	4.0 l/min
Scan End	3000 m/z	Set End Plate Offset	-500 V	Set Divert Valve	Waste

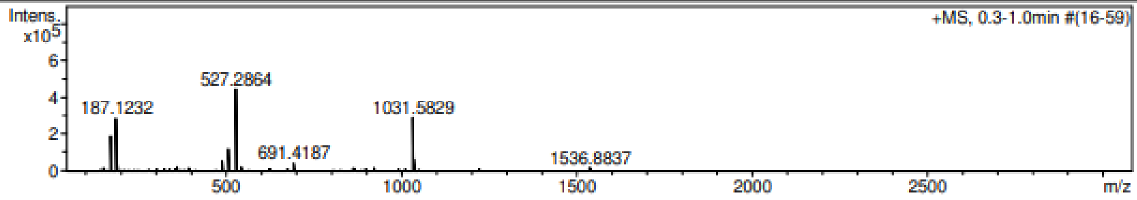

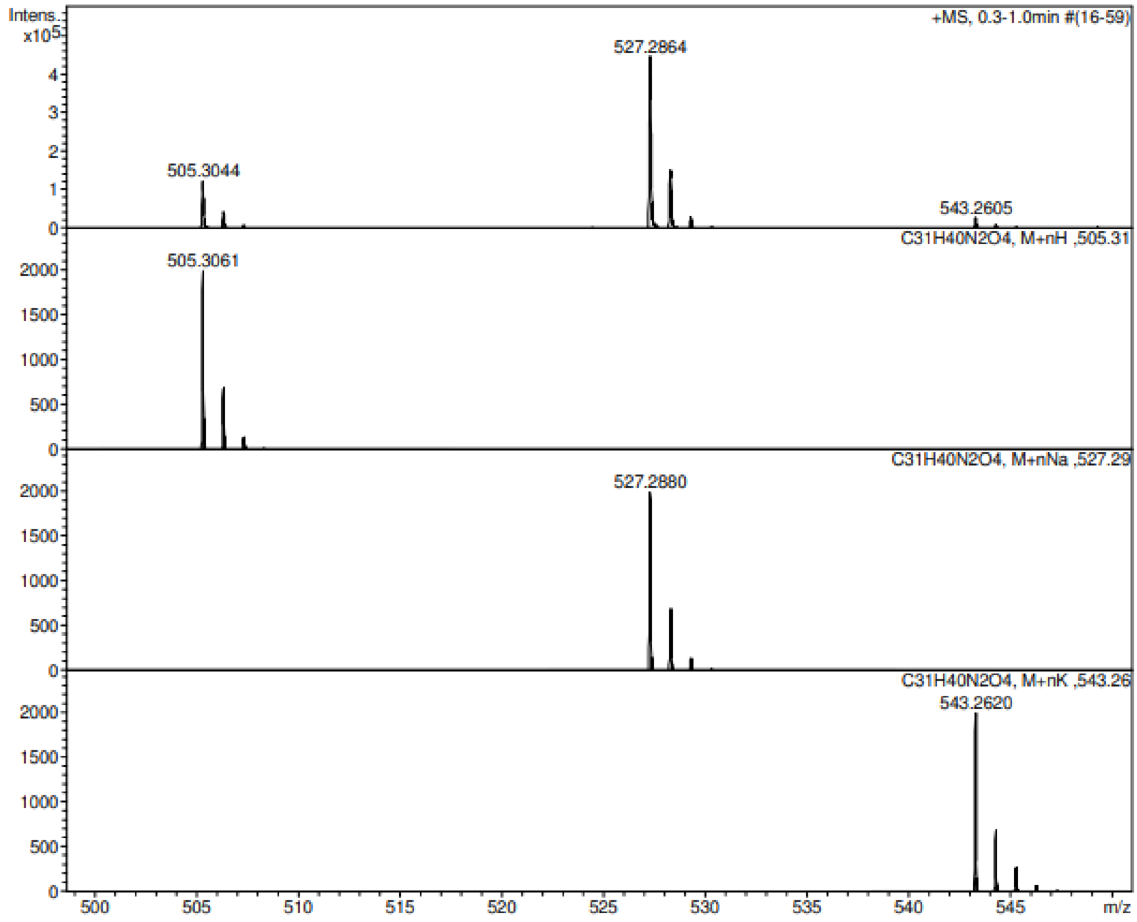

Bruker Compass DataAnalysis 4.0 printed: 14.11.2023 16:33:25 Page 1 of 1

HRMS spectrum of 4-(4-Fluorobenzyl)-1-{3-[(1*S*,2*S*,4a*S*,10a*R*)-2-hydroxy-7-methoxy-2-methyl-1,2,3,4,4a,9,10,10a-octahydrophenanthren-1-yl]propanoyl}-3,5-dimethyl-4,5-dihydro-1*H*-pyrazol-5-ol **(3j)**

Display Report

Analysis Info

Analysis Name	D:\Data\Kolotyrkina\2024\Ilovaiskii\0227019.d	Acquisition Date	27.02.2024 15:15:40
Method	tune_low.m	Operator	BDAL@DE
Sample Name	/ILOV merk5311	Instrument / Ser#	micrOTOF 10248
Comment	C30H37FN2O4 mH509.2810 calibrant added CH3OH		

Acquisition Parameter

Source Type	ESI	Ion Polarity	Positive	Set Nebulizer	0.4 Bar
Focus	Not active			Set Dry Heater	180 °C
Scan Begin	50 m/z	Set Capillary	4500 V	Set Dry Gas	4.0 l/min
Scan End	3000 m/z	Set End Plate Offset	-500 V	Set Divert Valve	Waste

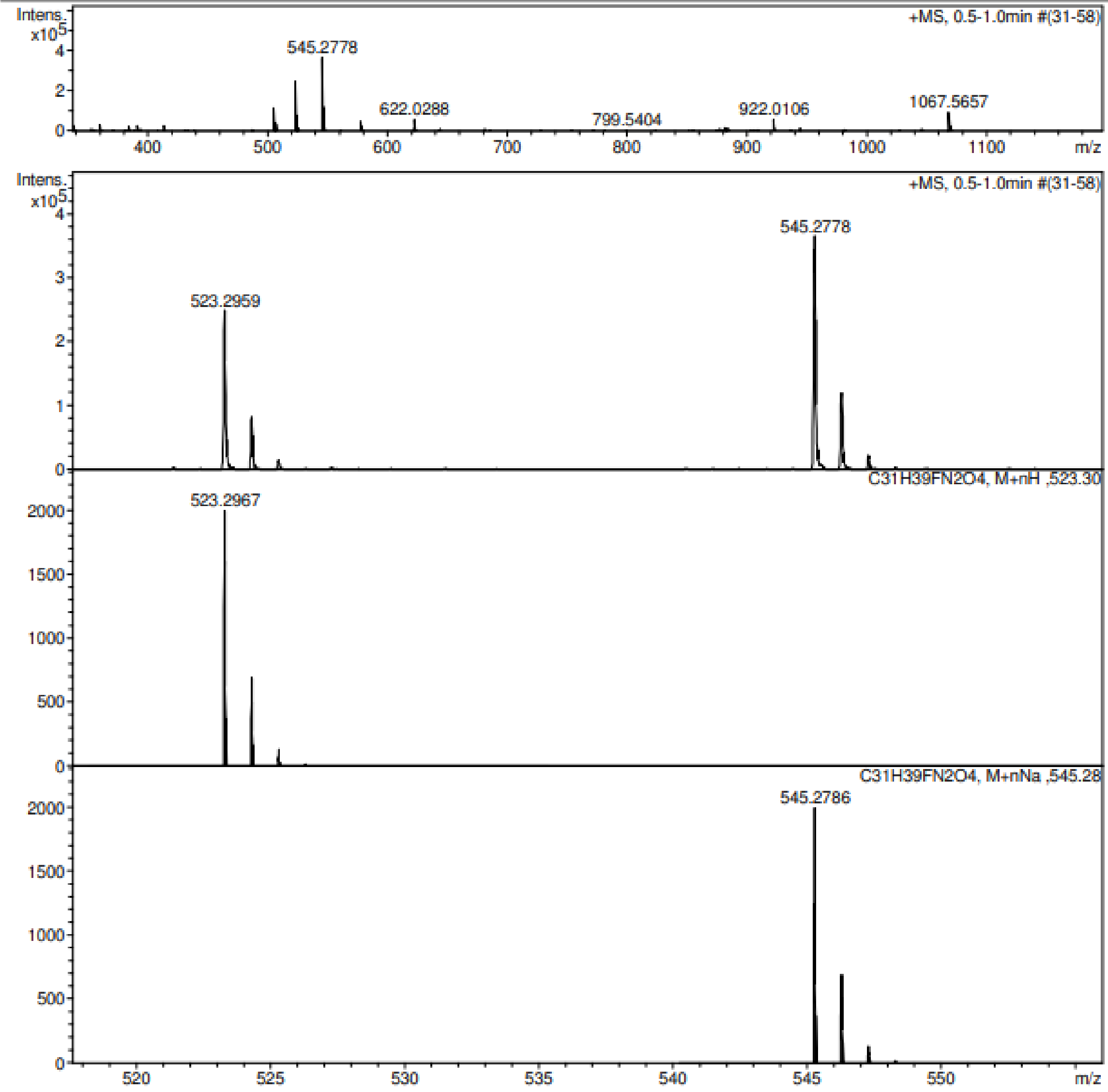

HRMS spectrum of 4-(4-Chlorobenzyl)-1-{3-[(1*S*,2*S*,4a*S*,10a*R*)-2-hydroxy-7-methoxy-2-methyl-1,2,3,4,4a,9,10,10a-octahydrophenanthren-1-yl]propanoyl}-3,5-dimethyl-4,5-dihydro-1*H*-pyrazol-5-ol **(3k)**

Display Report

Analysis Info

Analysis Name	D:\Data\Kolotyrkina\2023\Ilovaisky\1214024.d	Acquisition Date	14.12.2023 17:45:13
Method	tune_low.m	Operator	BDAL@DE
Sample Name	/ILOV merk5166	Instrument / Ser#	micrOTOF 10248
Comment	C30H37ClN2O4 mH 525.2514 clb added CH3OH		

Acquisition Parameter

Source Type	ESI	Ion Polarity	Positive	Set Nebulizer	0.4 Bar
Focus	Not active			Set Dry Heater	180 °C
Scan Begin	50 m/z	Set Capillary	4500 V	Set Dry Gas	4.0 l/min
Scan End	3000 m/z	Set End Plate Offset	-500 V	Set Divert Valve	Waste

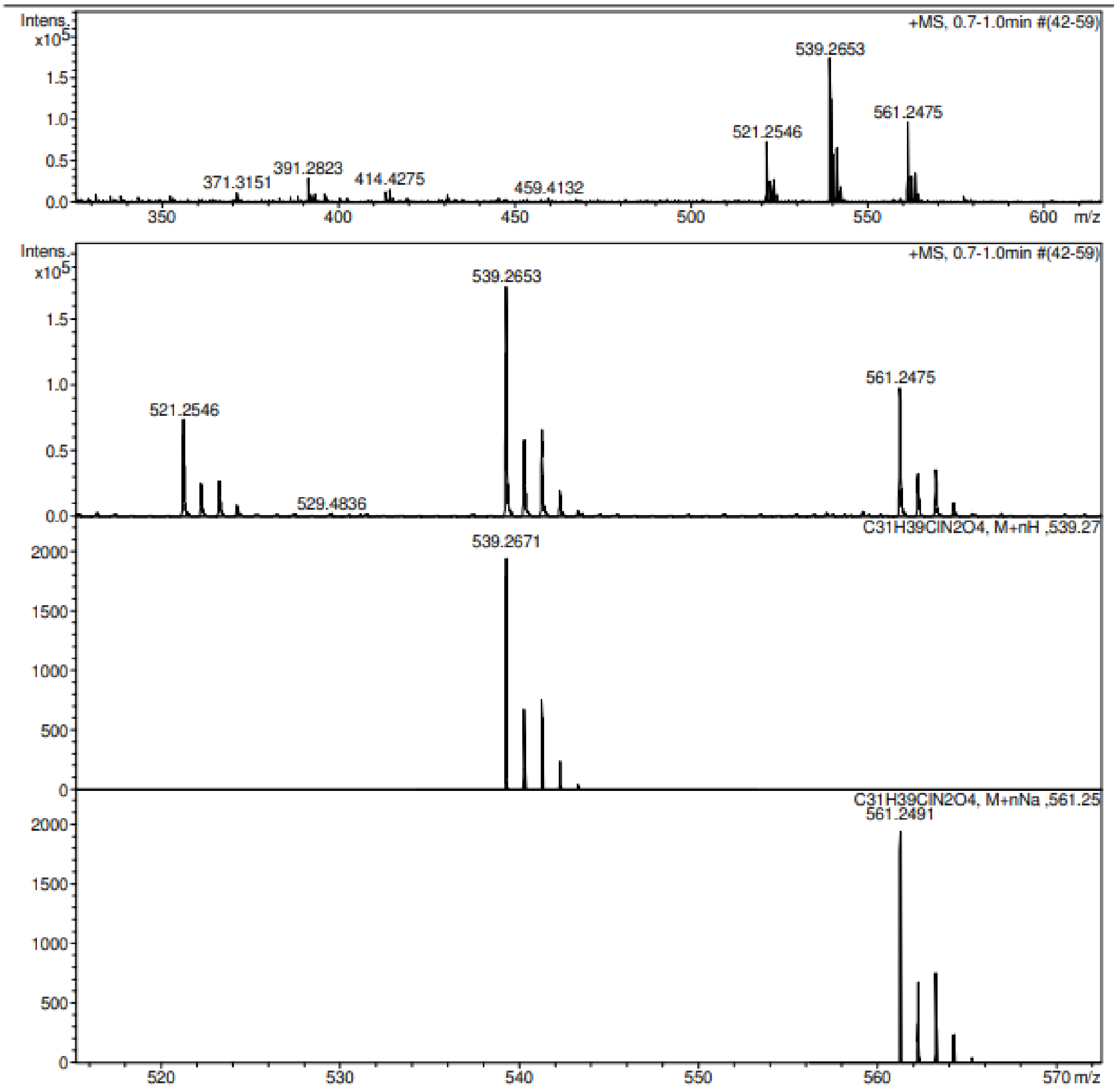

HRMS spectrum of 4-(4-Bromobenzyl)-1-{3-[(1*S*,2*S*,4a*S*,10a*R*)-2-hydroxy-7-methoxy-2-methyl-1,2,3,4,4a,9,10,10a-octahydrophenanthren-1-yl]propanoyl}-3,5-dimethyl-4,5-dihydro-1*H*-pyrazol-5-ol **(3l)**

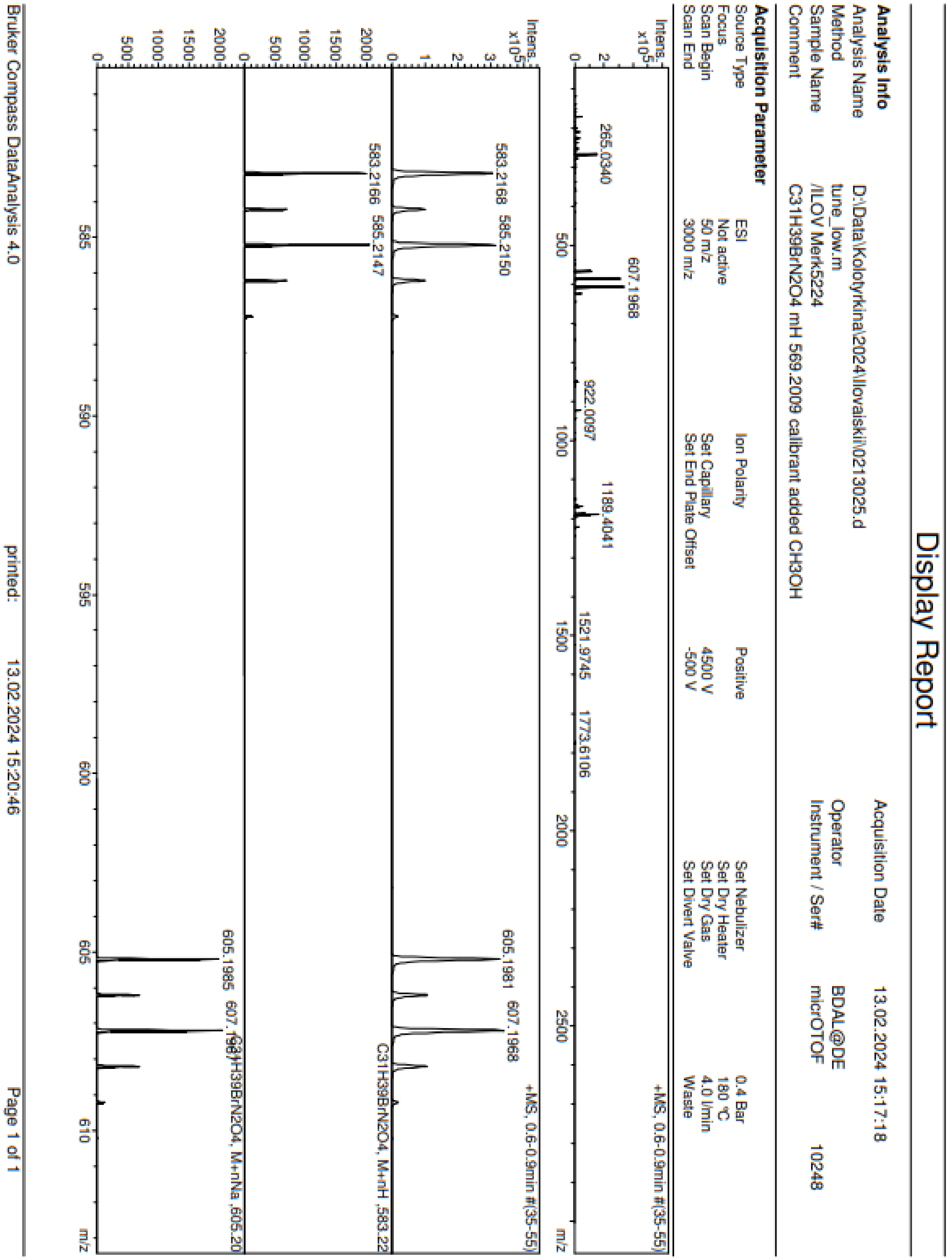

HRMS spectrum of 3-(5-Hydroxy-1-{3-[(1*S*,2*S*,4a*S*,10a*R*)-2-hydroxy-7-methoxy-2-methyl-1,2,3,4,4a,9,10,10a-octahydrophenanthren-1-yl]propanoyl}-3,5-dimethyl-4,5-dihydro-1*H*-pyrazol-4-yl)propanenitrile **(3m)**

Display Report

Analysis Info

Analysis Name	D:\Data\Kolotyrkina\2024\Ilovaiskii\0111027.d	Acquisition Date	11.01.2024 17:28:44
Method	tune_low.m	Operator	BDAL@DE
Sample Name	/ILOV Merk5206	Instrument / Ser#	micrOTOF 10248
Comment	C27H37N3O4 mH468.2867 calibrant added CH3CN		

Acquisition Parameter

Source Type	ESI	Ion Polarity	Positive	Set Nebulizer	0.4 Bar
Focus	Not active			Set Dry Heater	180 °C
Scan Begin	50 m/z	Set Capillary	4500 V	Set Dry Gas	4.0 l/min
Scan End	3000 m/z	Set End Plate Offset	-500 V	Set Divert Valve	Waste

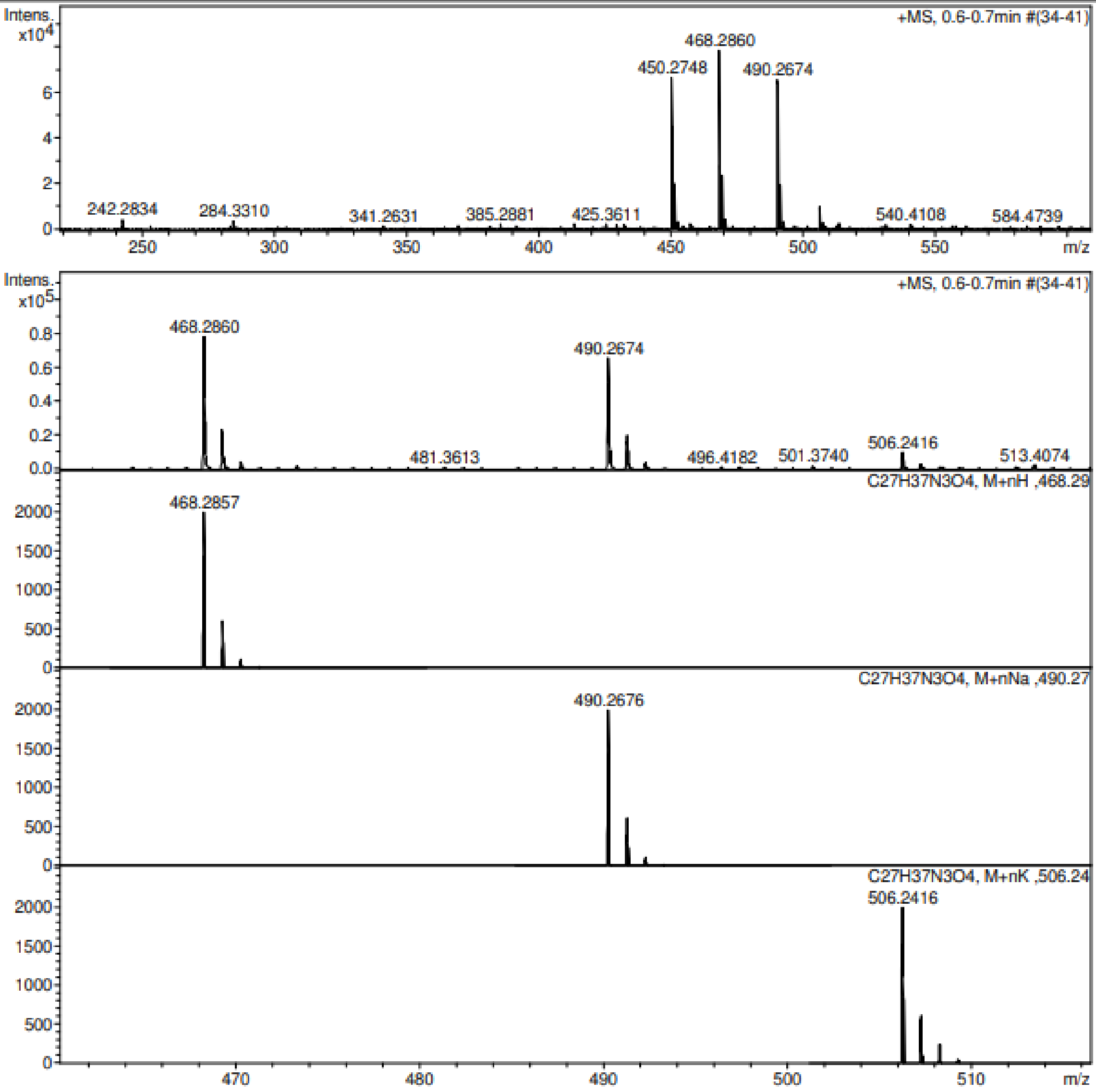

Bruker Compass DataAnalysis 4.0 printed: 11.01.2024 17:32:27 Page 1 of 1

HRMS spectrum of Ethyl 3-(5-hydroxy-1-{3-[(1*S*,2*S*,4a*S*,10a*R*)-2-hydroxy-7-methoxy-2-methyl-1,2,3,4,4a,9,10,10a-octahydrophenanthren-1-yl]propanoyl}-3,5-dimethyl-4,5-dihydro-1*H*-pyrazol-4-yl)propanoate **(3n)**

Display Report

Analysis Info

Analysis Name	D:\Data\Kolotyrkina\2024\Ilovaiskii\1218020.d	Acquisition Date	18.12.2024 17:31:42
Method	tune_low.m	Operator	BDAL@DE
Sample Name	/ILOV MERK5695	Instrument / Ser#	micrOTOF 10248
Comment	C29H42N2O6 clb added CH3OH		

Acquisition Parameter

Source Type	ESI	Ion Polarity	Positive	Set Nebulizer	0.4 Bar
Focus	Not active			Set Dry Heater	180 °C
Scan Begin	50 m/z	Set Capillary	4500 V	Set Dry Gas	4.0 l/min
Scan End	3000 m/z	Set End Plate Offset	-500 V	Set Divert Valve	Waste

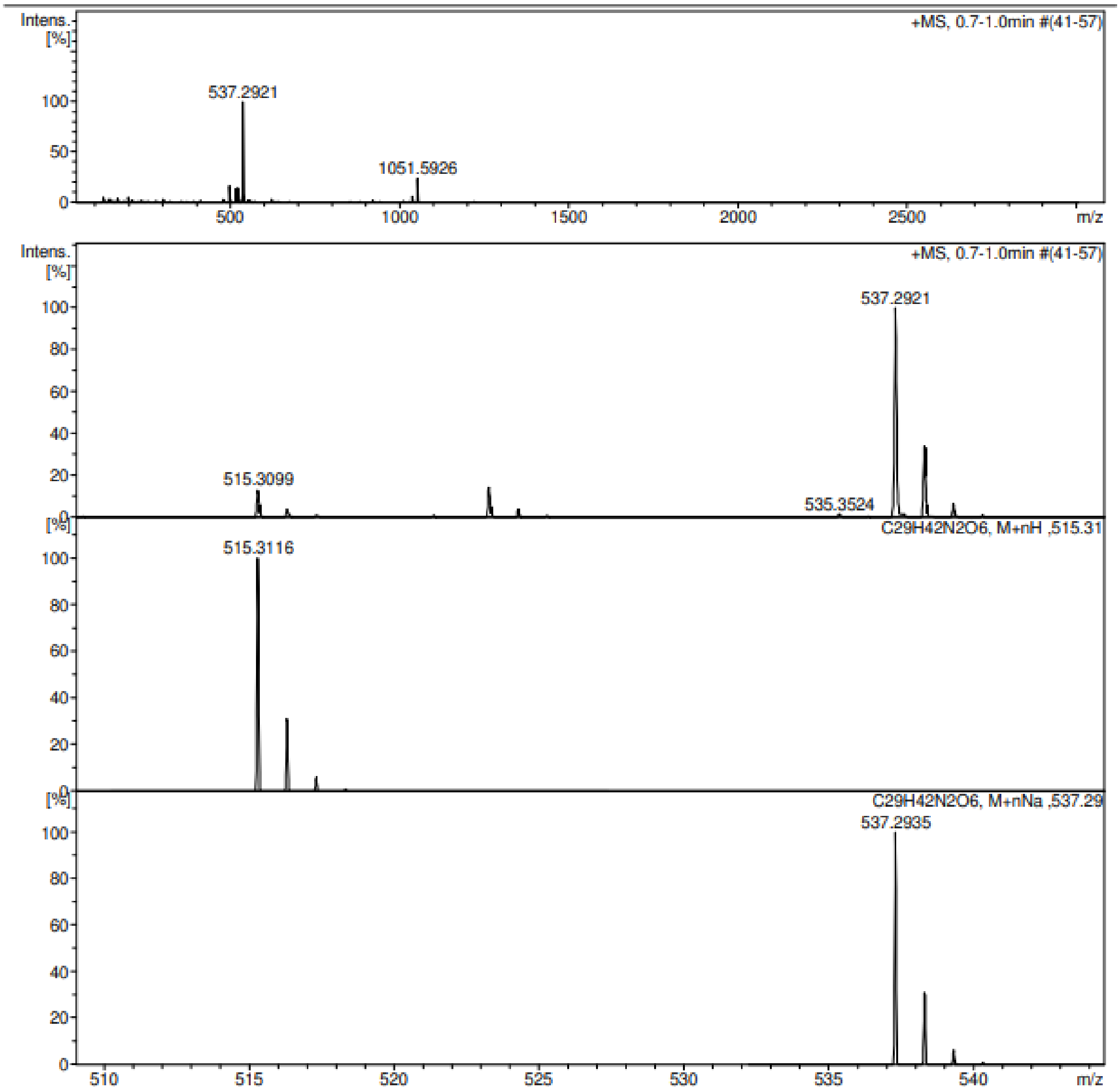

Bruker Compass DataAnalysis 4.0 printed: 18.12.2024 17:35:21 Page 1 of 1

HRMS spectrum of 3,5-Diethyl-1-{3-[(1*S*,2*S*,4a*S*,10a*R*)-2-hydroxy-7-methoxy-2-methyl-1,2,3,4,4a,9,10,10a-octahydrophenanthren-1-yl]propanoyl}-4,5-dihydro-1*H*-pyrazol-5-ol **(3o)**

Display Report

Analysis Info

Analysis Name	D:\Data\Kolotyrkina\2023\Ilovaisky\1109010.d	Acquisition Date	09.11.2023 17:09:29
Method	tune_low.m	Operator	BDAL@DE
Sample Name	/ILOV Merk5111	Instrument / Ser#	micrOTOF 10248
Comment	C26H38N2O4 mH443.2904 calibrant added CH3OH		

Acquisition Parameter

Source Type	ESI	Ion Polarity	Positive	Set Nebulizer	0.4 Bar
Focus	Not active			Set Dry Heater	180 °C
Scan Begin	50 m/z	Set Capillary	4500 V	Set Dry Gas	4.0 l/min
Scan End	3000 m/z	Set End Plate Offset	-500 V	Set Divert Valve	Waste

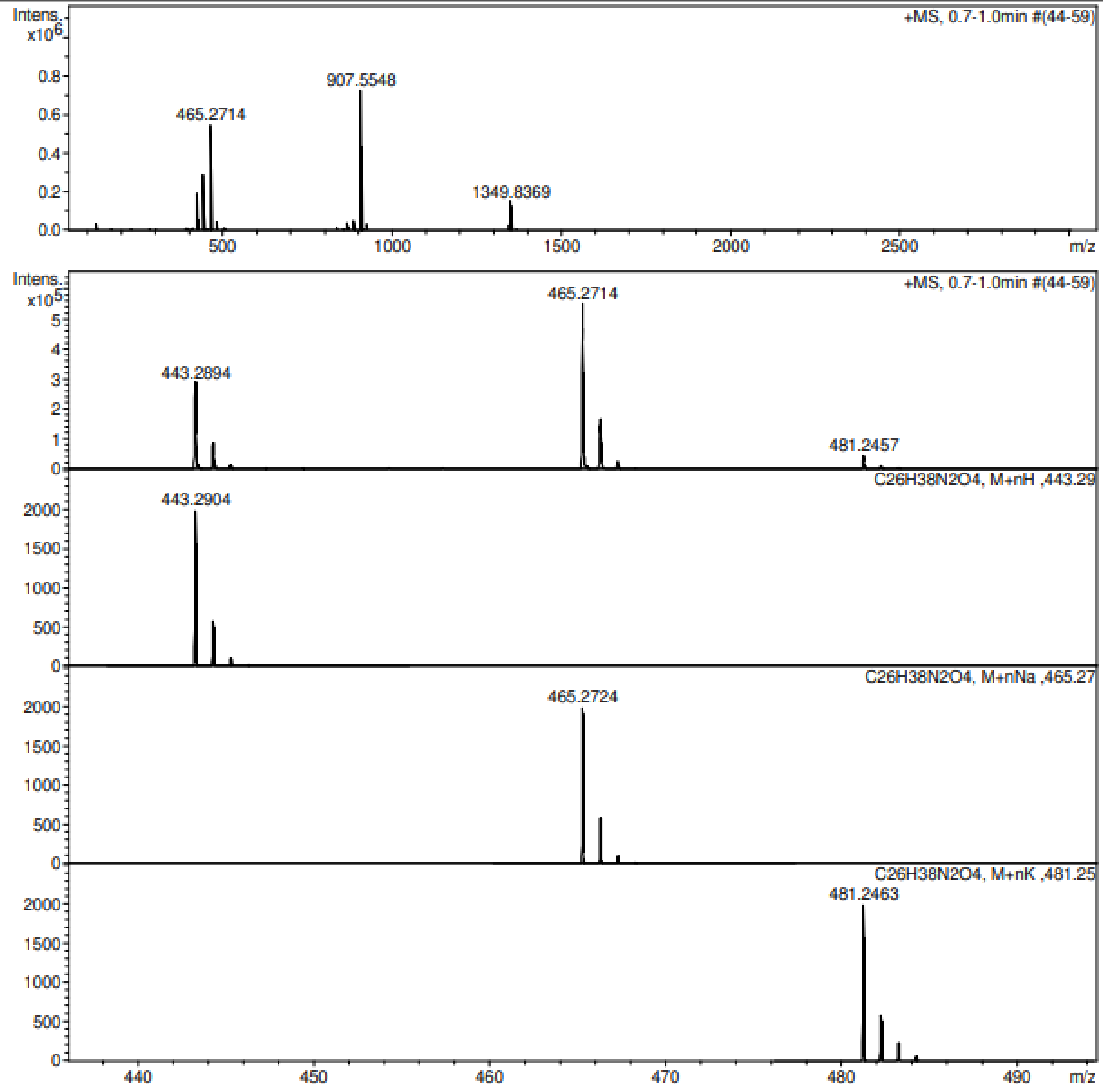

HRMS spectrum of 3,5-Diethyl-1-{3-[(1*S*,2*S*,4a*S*,10a*R*)-2-hydroxy-7-methoxy-2-methyl-1,2,3,4,4a,9,10,10a-octahydrophenanthren-1-yl]propanoyl}-4-methyl-4,5-dihydro-1*H*-pyrazol-5-ol (**3p**)

Display Report

Analysis Info

Analysis Name	D:\Data\Kolotyrkina\2024\Ilovaiskii\0207003.d	Acquisition Date	07.02.2024 10:37:05
Method	tune_low.m	Operator	BDAL@DE
Sample Name	/ILOV MERK5221	Instrument / Ser#	micrOTOF 10248
Comment	C27H40N2O4 mH 457.3060 calibrant added CH3CN		

Acquisition Parameter

Source Type	ESI	Ion Polarity	Positive	Set Nebulizer	0.4 Bar
Focus	Not active			Set Dry Heater	180 °C
Scan Begin	50 m/z	Set Capillary	4500 V	Set Dry Gas	4.0 l/min
Scan End	3000 m/z	Set End Plate Offset	-500 V	Set Divert Valve	Waste

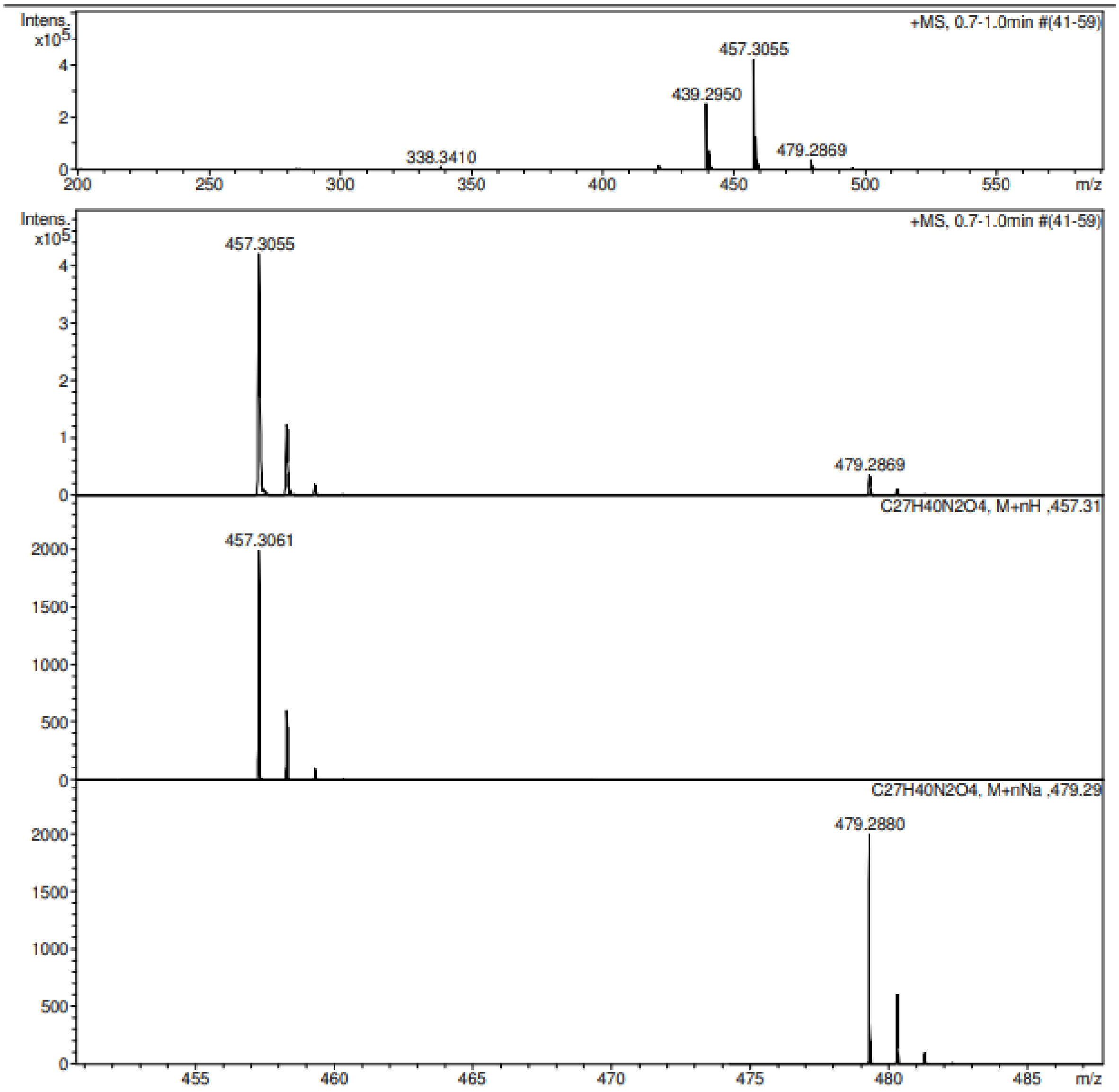

HRMS spectrum of 1-{3-[(1*S*,2*S*,4a*S*,10a*R*)-2-Hydroxy-7-methoxy-2-methyl-1,2,3,4,4a,9,10,10a-octahydrophenanthren-1-yl]propanoyl}-3-methyl-5-phenyl-4,5-dihydro-1*H*-pyrazol-5-ol (**3q**)

Display Report

Analysis Info

Analysis Name	D:\Data\Kolotyrkina\2024\Ilovaiskii\0201041.d	Acquisition Date	01.02.2024 17:49:09
Method	tune_low.m	Operator	BDAL@DE
Sample Name	/ILOV Merk5209	Instrument / Ser#	micrOTOF 10248
Comment	C29H36N2O4 mH477.2747 calibrant added CH3OH		

Acquisition Parameter

Source Type	ESI	Ion Polarity	Positive	Set Nebulizer	0.4 Bar
Focus	Not active			Set Dry Heater	180 °C
Scan Begin	50 m/z	Set Capillary	4500 V	Set Dry Gas	4.0 l/min
Scan End	3000 m/z	Set End Plate Offset	-500 V	Set Divert Valve	Waste

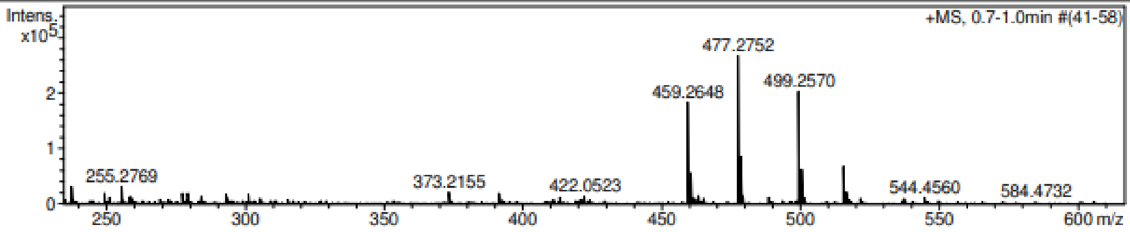

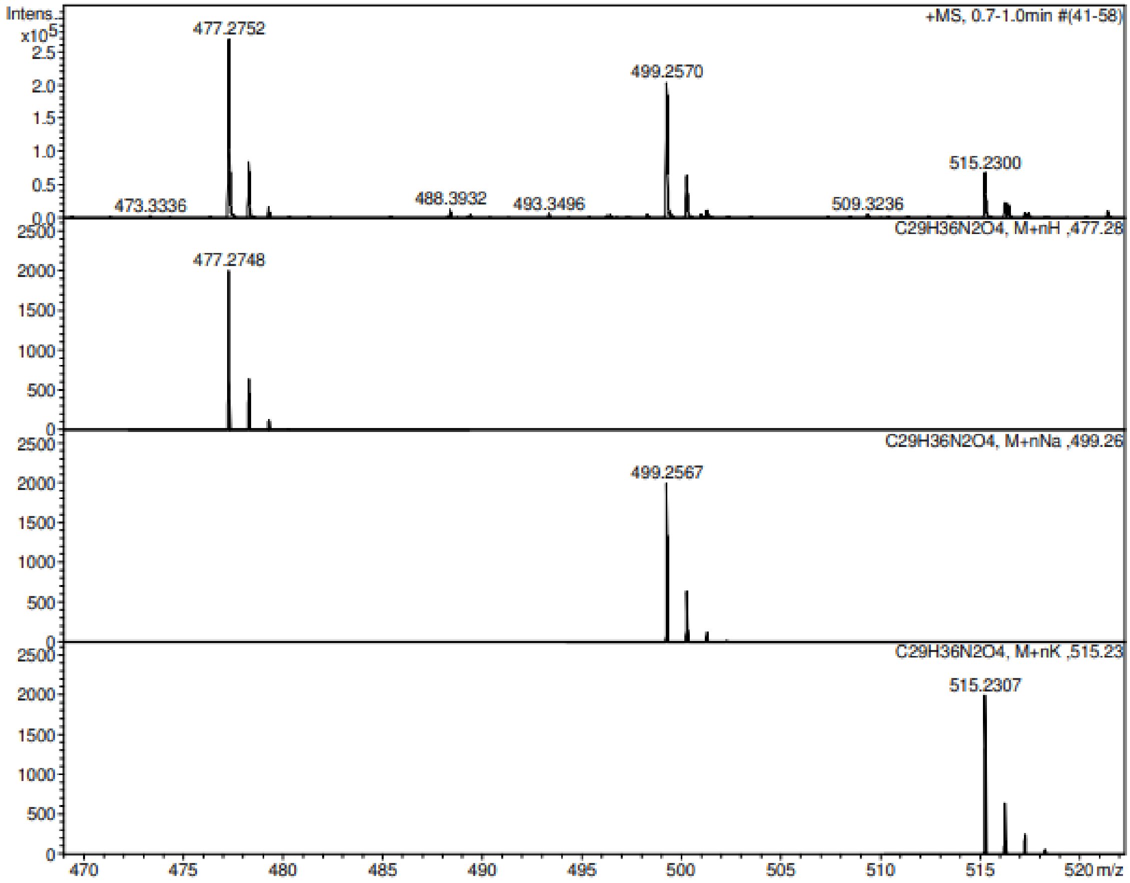

Bruker Compass DataAnalysis 4.0 printed: 01.02.2024 17:52:15 Page 1 of 1

HRMS spectrum of 5-(4-Fluorophenyl)-1-{3-[(1*S*,2*S*,4a*S*,10a*R*)-2-hydroxy-7-methoxy-2-methyl-1,2,3,4,4a,9,10,10a-octahydrophenanthren-1-yl]propanoyl}-3-methyl-4,5-dihydro-1*H*-pyrazol-5-ol (**3r**)

Display Report

Analysis Info

Analysis Name	D:\Data\Kolotyrkina\2023\Ilovaisky\0919004.d	Acquisition Date	19.09.2023 12:39:15
Method	tune_low.m	Operator	BDAL@DE
Sample Name	/ILOV Merk5077	Instrument / Ser#	micrOTOF 10248
Comment	C29H35FN2O4 mH 495.2653 calibrant added CH3CN		

Acquisition Parameter

Source Type	ESI	Ion Polarity	Positive	Set Nebulizer	0.4 Bar
Focus	Not active			Set Dry Heater	180 °C
Scan Begin	50 m/z	Set Capillary	4500 V	Set Dry Gas	4.0 l/min
Scan End	3000 m/z	Set End Plate Offset	-500 V	Set Divert Valve	Waste

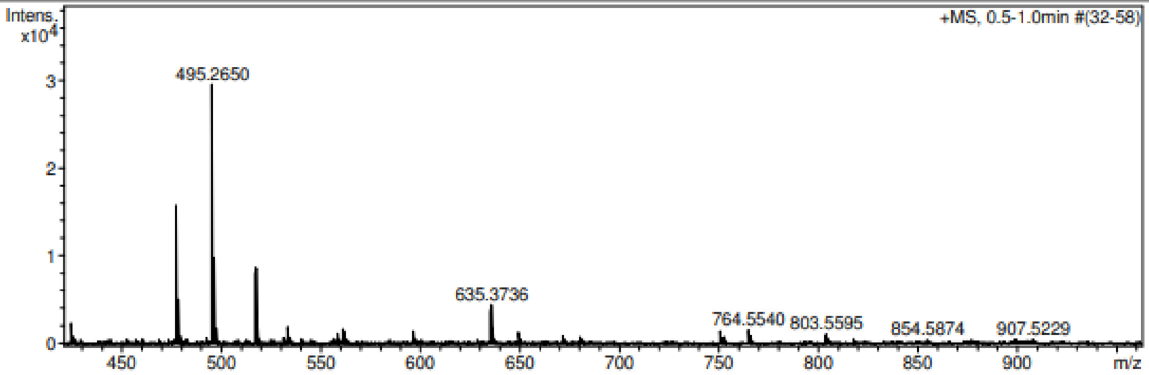

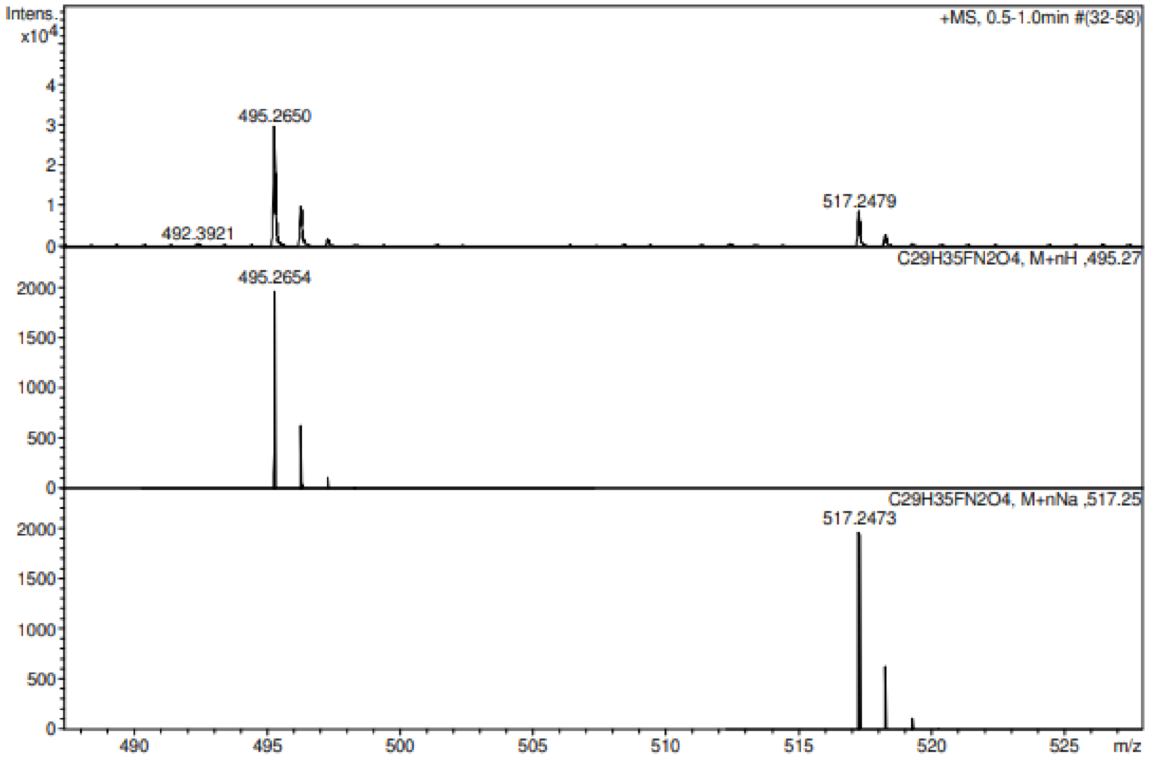

HRMS spectrum of 5-(4-Bromophenyl)-1-{3-[(1*S*,2*S*,4a*S*,10a*R*)-2-hydroxy-7-methoxy-2-methyl-1,2,3,4,4a,9,10,10a-octahydrophenanthren-1-yl]propanoyl}-3-methyl-4,5-dihydro-1*H*-pyrazol-5-ol (**3s**)

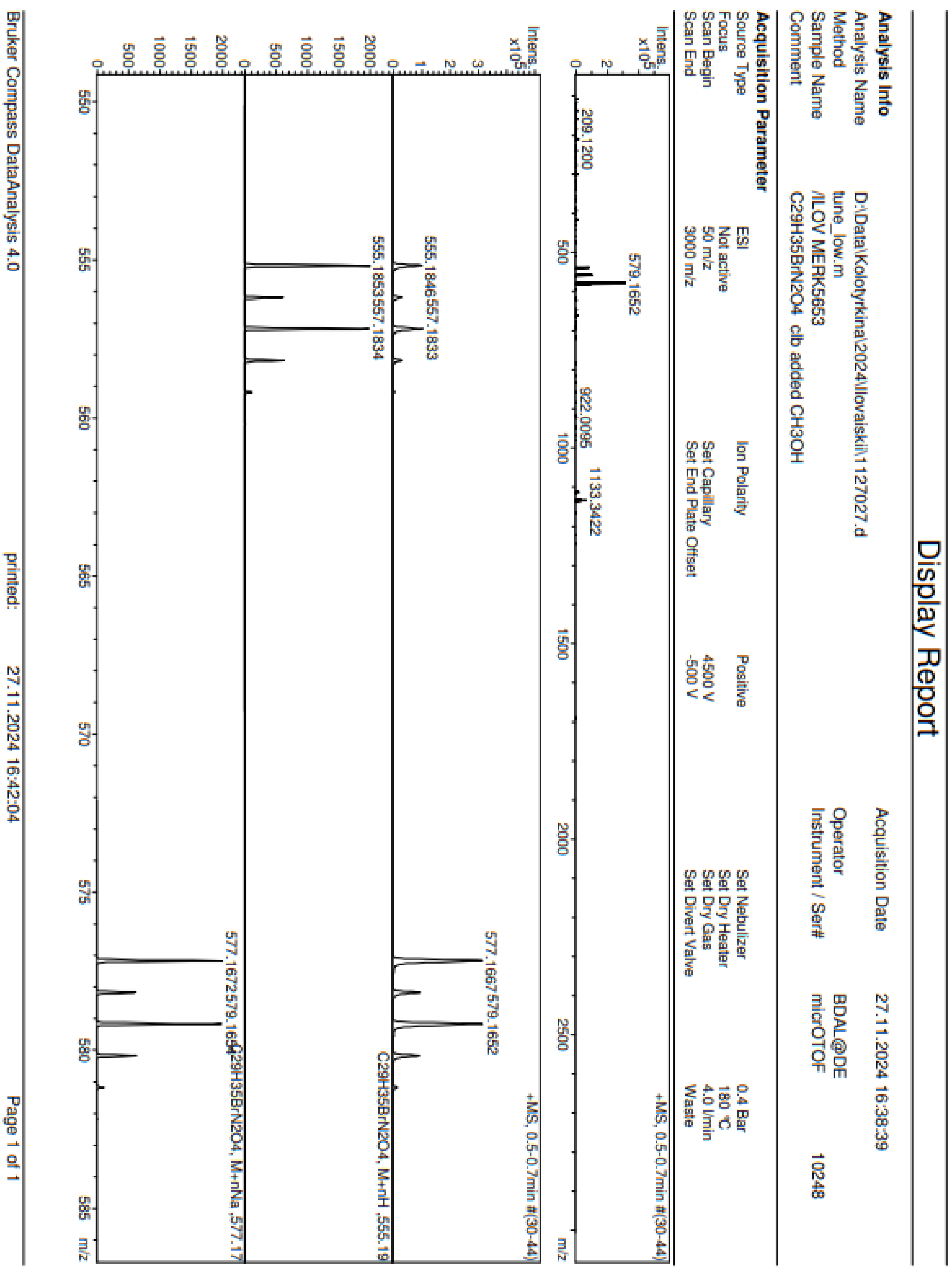

HRMS spectrum of 1-{3-[(1*S*,2*S*,4a*S*,10a*R*)-2-Hydroxy-7-methoxy-2-methyl-1,2,3,4,4a,9,10,10a-octahydrophenanthren-1-yl]propanoyl}-3-methyl-5-(trifluoromethyl)-4,5-dihydro-1*H*-pyrazol-5-ol (**3t**)

Display Report

Analysis Info

Analysis Name	D:\Data\Chizhov\Terentiev\Ilovaiskii\merk5014_&clb.d	Acquisition Date	04.08.2023 12:47:10
Method	tune_wide.m	Operator	BDAL@DE
Sample Name	/ILOV merk5014	Instrument / Ser#	micrOTOF 10248
Comment	CH3CN 100 %, dil. 200 calibrant added		

Acquisition Parameter

Source Type	ESI	Ion Polarity	Positive	Set Nebulizer	0.4 Bar
Focus	Not active			Set Dry Heater	180 °C
Scan Begin	50 m/z	Set Capillary	4500 V	Set Dry Gas	4.0 l/min
Scan End	3000 m/z	Set End Plate Offset	-500 V	Set Divert Valve	Waste

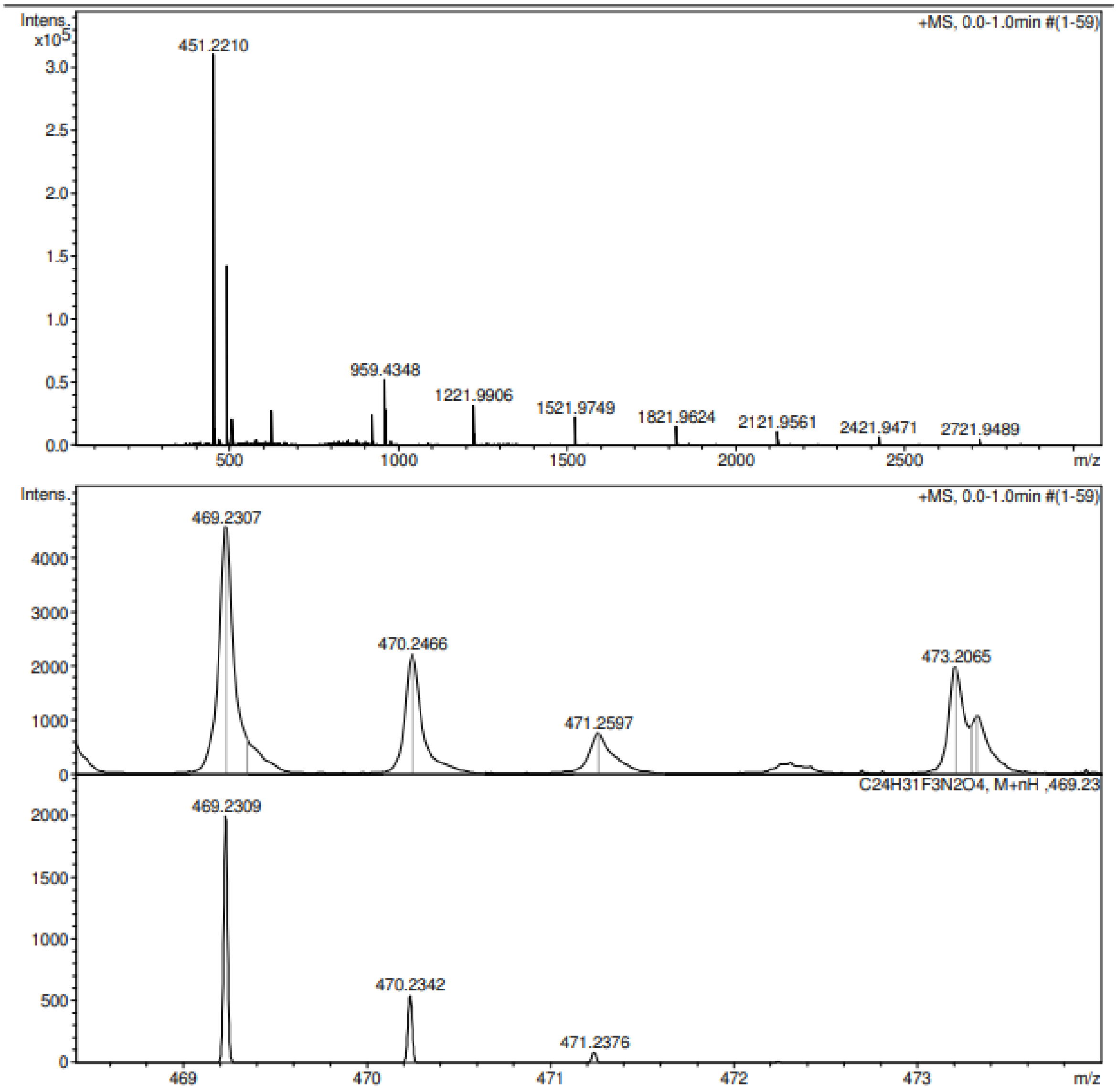

Supplement: Supplementary file 1 [file biomedicines-13-03057-s001.zip › biomedicines-3983383-supplementary.pdf]
